# Regio‐ and Stereoselective Thianthrenation of Olefins To Access Versatile Alkenyl Electrophiles

**DOI:** 10.1002/anie.201914215

**Published:** 2020-02-03

**Authors:** Junting Chen, Jiakun Li, Matthew B. Plutschack, Florian Berger, Tobias Ritter

**Affiliations:** ^1^ Max-Planck-Institut für Kohlenforschung Kaiser-Wilhelm-Platz 1 45470 Mülheim an der Ruhr Germany

**Keywords:** alkenes, alkenyl electrophiles, C−H functionalization, cross-coupling, regioselectivity

## Abstract

Herein, we report a regioselective alkenyl electrophile synthesis from unactivated olefins that is based on a direct and regioselective C−H thianthrenation reaction. The selectivity is proposed to arise from an unusual inverse‐electron‐demand hetero‐Diels–Alder reaction. The alkenyl sulfonium salts can serve as electrophiles in palladium‐ and ruthenium‐catalyzed cross‐coupling reactions to make alkenyl C−C, C−Cl, C−Br, and C−SCF_3_ bonds with stereoretention.

## Graphical abstract

**The regioselective synthesis** of sulfonium‐based alkenyl electrophiles was achieved directly through C−H functionalization of simple olefins. The alkenyl sulfonium salts participate in palladium‐catalyzed cross‐coupling and ruthenium‐catalyzed (pseudo)halogenation reactions.

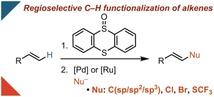

Olefins are among the most useful building blocks in organic synthesis, but the direct synthesis of alkenyl electrophiles from olefins is an unsolved problem.^1^ While olefins display C(sp^2^)−H bonds like arenes, their reactivity is distinct from arenes when treated with electrophiles: arenes undergo electrophilic C(sp^2^)−H substitution, while olefins typically undergo addition reactions such as dihalogenation to generate dihaloalkanes.^2^ Despite the synthetic utility of alkenyl electrophiles, a general direct and regioselective functionalization of olefins by C(sp^2^)−H substitution to access them is not yet known.^3^ Herein, we report a regio‐ and stereoselective method that affords alkenyl electrophiles directly from unactivated alkenes by C(sp^2^)−H substitution (Scheme 1). The high stereoselectivity may be the consequence of an unusual inverse‐electron‐demand hetero‐Diels–Alder reaction with a previously unused dicationic aromatic thianthrene dication. The resulting alkenyl sulfonium salts are versatile electrophiles for palladium‐ and ruthenium‐catalyzed cross‐coupling reactions.

**Scheme 1:**
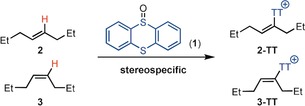
Stereoselective C(sp^2^)−H thianthrenation of olefins. TT=thianthrenium.

Alkenyl halides, especially alkenyl bromides and iodides, are widely used alkenyl electrophiles for cross‐coupling and other reactions.^4^ Their syntheses often require multistep sequences, for instance, dibromination of alkenes followed by the elimination of HBr under harsh conditions,^5^ or the prior synthesis of alkenyl nucleophiles, such as alkenyl boronates,^6^ alkenyl silanes,^7^ or other metal species,^8^ followed by treatment with electrophilic halogen sources. Methods starting from alkynes can afford alkenyl halides in one step, although they are less widely used considering the smaller availability of alkynes when compared to alkenes.^8^ Other frequently used methods such as the Takai and Wittig olefination reactions often give *E*/*Z* mixtures starting from aldehydes or ketones.^9^ It is desirable to access alkenyl (pseudo)halides directly from widely available alkenes, but functionalizations of alkenes commonly deliver hydrofunctionalized or vicinally difunctionalized alkanes.^10^ Modern olefin metathesis catalysts can sometimes access the *Z* isomers of alkenyl halides through cross‐metathesis,3e and also *E* alkenyl halides in some cases when bulky substituents are present in the starting material.^11^ Alkenyl nucleophiles are available directly from the C(sp^2^)−H functionalization of alkenes to give alkenyl silane^12^ and boronate^13^ species, for example. Michael acceptors such as nitroolefins^14^ and alkenyl nitriles^15^ can also be synthesized from alkenes. Procter and co‐workers disclosed the synthesis of alkenyl sulfoniums via an interrupted Pummerer approach, but the reaction is limited to styrene‐like substrates.^16^ The Carreira group reported a directed C(sp^2^)−H functionalization to make alkenyl iodides that bear a picolinamide directing group.^17^ The direct synthesis of versatile alkenyl electrophiles from simple olefins by C(sp^2^)−H functionalization, however, is as of yet unknown.^12^, ^13^, ^14^, ^15^, ^16^, ^18^ Here we fill this gap, and showcase the utility of the alkenyl sulfonium electrophiles in several cross‐coupling reactions. The chemistry differs conceptually from prior art because of the unusual mechanism of olefin functionalization, which sets the chemistry apart from arene thianthrenation and other syntheses of alkenyl sulfonium species.

We have recently published a site‐selective aromatic C(sp^2^)−H functionalization reaction of in situ activated thianthrene‐*S*‐oxides with trifluoroacetic anhydride.19a The formed aryl thianthrenium salts were used as aryl electrophiles to form challenging bonds such as aryl C−SCF_3_,19a C−CF_3_,19b C−N,19c C−O,19d and C−F19e, 19f bonds. An extension of aromatic substitution chemistry to olefins is generally not successful because olefins typically react with electrophiles by addition. Thianthrene radical cation addition to olefins has been explored.^20^ In contrast to this addition chemistry and our previous arene substitution, we disclose here data that supports a distinct mechanism for alkene substitution that allows for the selective synthesis of alkenyl sulfonium salts from thianthrene‐*S*‐oxide.

The reaction is straightforward to execute: Alkene (1.00 equiv) and thianthrene‐*S*‐oxide (**1**; 1.03 equiv) are dissolved in anhydrous acetonitrile under ambient atmosphere, followed by sequential addition of trifluoroacetic anhydride and trifluoromethanesulfonic acid with cooling. The reaction is complete within less than two hours. The products can be purified by column chromatography (Table 1).

**Table 1:** Regioselective C(sp^2^)−H thianthrenation of unactivated alkenes.^[a]^ 
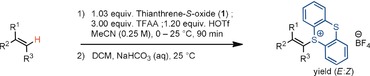

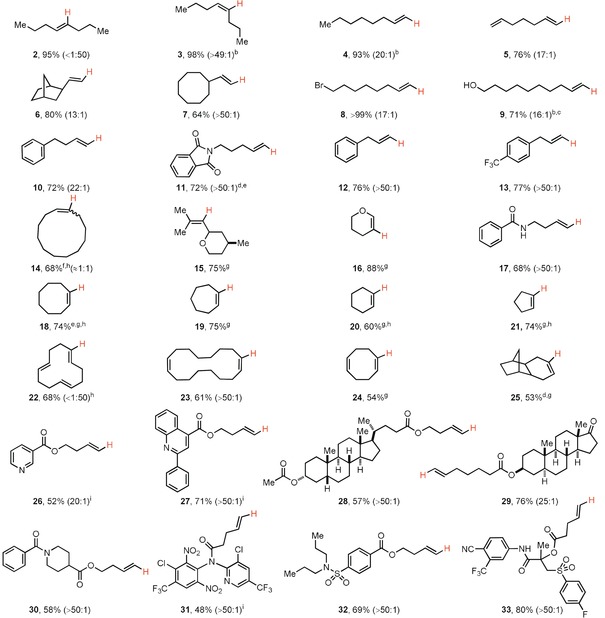

[a] On 0.500 mmol scale; **1** (1.03 equiv), TFAA (3.00 equiv), HOTf (1.20 equiv). Yields of isolated products. *E*/*Z* ratios determined by ^1^H NMR analysis. [b] 2.40 equiv of HOTf were used to obtain a better *E*/*Z* ratio. [c] The alcohol was partially acylated during the reaction (see the Supporting Information). [d] 1.20 equiv HBF_4_⋅Et_2_O instead of HOTf was used to obtain a better *E*/*Z* ratio. [e] The reaction was performed on 3.00 mmol scale. [f] Cyclododecene starting material used as mixture of isomers (*E*/*Z*≈1:2; see the Supporting Information). [g] *E*/*Z* ratio not applicable. [h] 1.20 equiv HBF_4_⋅Et_2_O instead of HOTf were used to obtain higher yields. [i] 2.40 equiv of HOTf were used to obtain higher yields. TFAA=trifluoroacetic anhydride, HOTf=trifluoromethanesulfonic acid.

For all α‐olefins, the *E*‐alkenyl sulfonium salts were obtained as major products, often without detectable *Z* isomers, when analyzed by NMR spectroscopy. Functionalizations of internal alkenes proceeded with retention of the double bond geometry for both *E* (**2**, **22**) and *Z* (**3**, **23**) olefins. Steric bulk in the allylic position does not interfere with productive functionalization (**6**, **7**). Functional groups such as imide (**11**), primary alkyl bromide (**8**), alcohol (**9**), ether (**15**, **16**), amide (**17**), ester (**26**–**33**), ketone (**29**), piperidine (**30**), nitro (**31**), sulfonamide (**32**), sulfone (**33**), and nitrile (**33**) moieties are all tolerated. Electron‐rich arenes can undergo aromatic substitution,19c but electron‐poor (**11**, **13**), electron‐neutral (**12**), and six‐membered heterocyclic arenes (**26**, **27**, **31**) are tolerated. Even bis‐benzylic and allylic C(sp^3^)−H bonds (**12**, **13**) are tolerated without double bond isomerization or allylic functionalization.13b, 18, 21 Internal olefins that are electronically biased such as enol ethers (**16**) are functionalized regioselectively, as are all α‐olefins, but the regioselectivity diminishes for other 1,2‐disubstituted olefins.^22^ Trisubstituted olefins (**15**) on the other hand are functionalized regioselectively. Cyclic olefins (**18**, **19**, **20**, **21**) participate well. Selective monofunctionalization is observed for dienes (**5**, **23**, **24**) and a triene (**22**), most likely because of the introduction of a cationic substituent in an electrophilic pathway.

To rationalize the selectivity, especially the unusually high selectivity with respect to the double bond geometry such as in **2** and **3** (Scheme 2), we attempted to gain further insight into the reaction mechanism. Both the cycloadducts **2‐INT** and **3‐INT** as well as the high selectivity are consistent with an unusual [4+2] cycloaddition between the olefin and the dicationic thianthrenium dication (see the Supporting Information). To substantiate the viability of the unusual thianthrenium dication as a reaction partner, we observed it by cyclic voltammetry and recorded its UV/Vis spectrum in an electrical cell at constant potential (see the Supporting Information). We cannot rule out an addition of the thianthrene radical cation to the olefin, followed by single electron oxidation and subsequent cyclization to the bicyclic adducts, but oxidation and cyclization would need to occur faster than bond rotation around the C−C single bond to account for the observed selectivity. In addition, reactions of thianthrene radical cations with olefins can afford bis‐adducts, with two thianthrene radical cations reacting with one olefin.^20^ Attempts to thermally induce a cycloreversion of the cycloadduct **2‐INT** were not successful, possibly because of its two formal positive charges that also prevented experiments such as flash‐vacuum pyrolysis. When the work‐up of the reaction (aqueous bicarbonate) was omitted, we were able to isolate and fully characterize the two diastereomeric intermediates **2‐INT** and **3‐INT** from *E*‐ and *Z*‐4‐octene, respectively. Treatment of either bicycle with mild base, such as bicarbonate, resulted in clean and high‐yielding conversion into the corresponding alkenyl sulfonium salts **2‐TT** and **3‐TT**, respectively, with complete selectivity of olefin geometry. Stereoelectronic considerations dictate that the elimination cannot proceed by an E2 mechanism because of the geometry of the bicycles **2‐INT** and **3‐INT**, which prevents an antiperiplanar arrangement of leaving group and proton. An E1 mechanism cannot be excluded but is unlikely because of the nearly complete degree of fidelity of double bond geometry. Consistent with all data is an E1cB_irr_ mechanism where rate‐determining deprotonation is followed by a rapid elimination.^23^

**Scheme 2:**
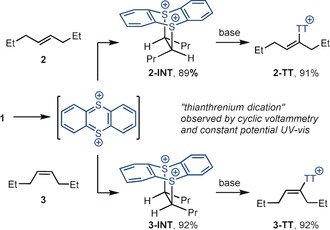
Mechanism of the regioselective thianthrenation of alkenyl C(sp^2^)−H bonds.

As their aromatic counterparts,^19^ the alkenyl thianthrenium salts serve as excellent electrophiles in follow‐up transformations and thus differ from other alkenyl sulfonium salts that have narrower reactivity (Table 2). Palladium‐catalyzed cross‐coupling reactions generally proceed with retention of the double bond geometry (**34**, **35**, **36**, and the Supporting Information). The alkenyl thianthrene salts also serve as suitable electrophiles for ruthenium‐based catalysis, which enable the synthesis of alkenyl halides (**37**, **38**) and pseudohalides (**39**), also stereoselectively: For example, chlorination in the presence of [Cp*Ru(MeCN)_3_]PF_6_ as the catalyst was performed successfully on gram scale, and an alkenyl trifluoromethyl thioether, which is difficult to access otherwise, was readily obtained.^24^ Although Ru^II^ catalysts are often proposed to undergo slow oxidative addition to alkenyl halides,^25^ [Cp*Ru(MeCN)_3_]PF_6_ showed robust catalytic reactivity in reactions with alkenyl thianthrenium salts as electrophiles. Retention of double bond geometry was observed for all cross‐coupling reactions, also those of trisubstituted olefins (see the Supporting Information).

**Table 2:** Derivatizations of alkenyl thianthrenium salts. 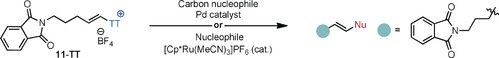

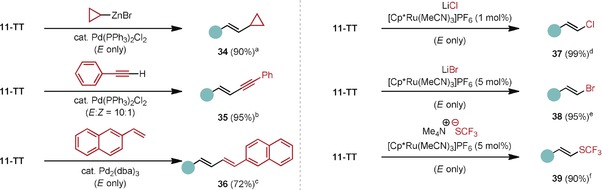

[a] CyclopropylZnBr (3.0 equiv), Pd(PPh_3_)_2_Cl_2_ (20 mol %) in THF (0.1 m). [b] Phenylacetylene (2.0 equiv), *N*‐methylmorpholine (2.0 equiv), CuI (25 mol %), Pd(PPh_3_)_2_Cl_2_ (10 mol %) in THF (0.1 m). [c] 2‐Vinylnaphthalene (2.0 equiv), K_2_CO_3_ (2.5 equiv), Pd_2_(dba)_3_ (10 mol %) in DMF (0.1 m). [d] LiCl (1.5 equiv), [Cp*Ru(MeCN)_3_]PF_6_ (1 mol %) in THF (0.1 m). [e] LiBr (1.5 equiv), [Cp*Ru(MeCN)_3_]PF_6_ (5 mol %) in THF (0.1 m). [f] [Me_4_N^+^][^−^SCF_3_] (1.2 equiv), [Cp*Ru(MeCN)_3_]PF_6_ (5 mol %) in THF (0.1 m).

In conclusion, we have developed a synthesis of alkenyl electrophiles, directly from unactivated olefins, that proceeds regio‐ and stereoselectively for a large variety of olefin classes. Preliminary mechanistic studies suggest that thianthrenation could proceed via an unusual inverse‐electron‐demand cycloaddition reaction. The alkenyl sulfonium salts, which may contain a large range of functional groups, are suitable electrophiles for Pd‐catalyzed C−C cross‐coupling reactions and Ru‐catalyzed C−X bond formation.

## Conflict of interest

T.R. and F.B. may benefit from royalty payments regarding sales of thianthrene‐related compounds.

## Supporting information

As a service to our authors and readers, this journal provides supporting information supplied by the authors. Such materials are peer reviewed and may be re‐organized for online delivery, but are not copy‐edited or typeset. Technical support issues arising from supporting information (other than missing files) should be addressed to the authors.

Supplementary
