# Supplementary material for: Regio‐ and Stereoselective Thianthrenation of Olefins To Access Versatile Alkenyl Electrophiles
Source: Angew Chem Int Ed Engl. 2020 Feb 3;59(14):5616–20. doi: 10.1002/anie.201914215 (PMC7154751; doi:10.1002/anie.201914215)

Angewandte Chemie
Eine Zeitschrift der Gesellschaft Deutscher Chemiker

Supporting Information

Regio- and Stereoselective Thianthrenation of Olefins To Access Versatile Alkenyl Electrophiles

*Junting Chen, Jiakun Li, Matthew B. Plutschack, Florian Berger, and Tobias Ritter**

anie_201914215_sm_miscellaneous_information.pdf

TABLE OF CONTENTS

TABLE OF CONTENTS 1

MATERIALS AND METHODS 11

Solvents 11

Chromatography 11

Spectroscopy and Instruments 11

Starting materials 11

EXPERIMENTAL DATA 12

Thianthrenation of alkenes 12

General procedure 12

trans-4-Octene-derived thianthrenium salt **2-TT** 12

cis-4-Octene-derived thianthrenium salt **3-TT** 13

1-Octene-derived thianthrenium salt **4-TT** 14

1,6-Heptadiene-derived thianthrenium salt **5-TT** 16

(-)-2-Vinylnorbornane-derived thianthrenium salt **6-TT** 17

Vinylcyclooctane-derived thianthrenium salt **7-TT** 18

8-Brom-1-octene-derived thianthrenium salt **8-TT** 19

10-Undecen-1-ol-derived thianthrenium salts **9-TT(OTFA)** and **9-TT(OH)** 19

4-Phenyl-1-butene-derived thianthrenium salt **10-TT** 21

Pent-4-en-1-yl-phthalimide-derived thianthrenium salt **11-TT** 22

Allylbenzol-derived thianthrenium salt **12-TT** 23

1-Allyl-4-(trifluormethyl)-benzol-derived thianthrenium salt **13-TT** 24

Cyclododecene-derived thianthrenium salt **14-TT** 25

(+)-Rosenoxide-derived thianthrenium salt **15-TT** 26

3,4-Dihydro-2*H*-pyrane-derived thianthrenium salt **16-TT** 27

N-(But-3-en-1-yl)benzamide-derived thianthrenium salt **17-TT** 27

Cyclooctene-derived thianthrenium salt **18-TT** 28

Cycloheptene-derived thianthrenium salt **19-TT** 29

Cyclohexene-derived thianthrenium salt **20-TT** 30

Cyclopentene-derived thianthrenium salt **21-TT** 31
(*E,E,E*)-1,5,9-Cyclododecatriene-derived thianthrenium salt **22-TT** 32
1,9-Cyclohexadecadiene-derived thianthrenium salt **23-TT** 32
1,5-Cyclooctadiene-derived thianthrenium salt **24-TT** 33
Tricyclo[6.2.1.02,7]undeca-4-ene-derived thianthrenium salt **25-TT** 34
Nicotinic acid-derived thianthrenium salt **26-TT** 35
Cinchophene-derived thianthrenium salt **27-TT** 36
Lithocholic acid-derived thianthrenium salt **28-TT** 37
Epiandrosterone-derived thianthrenium salt **29-TT** 38
Piperidine-derived thianthrenium salt **30-TT** 39
Fluazinam-derived thianthrenium salt **31-TT** 40
Probenecid-derived thianthrenium salt **32-TT** 41
Bicalutamide-derived thianthrenium salt **33-TT** 42
Evaluation on different *S*-oxide 43
Functionalization of alkenyl thianthrenium salts 43
Cyclopropyl-pent-4-en-1-yl-phthalimide (**34**) 43
Phenylacetylenyl-pent-4-en-1-yl-phthalimide (**35**) 44
Vinylnaphthalenyl-pent-4-en-1-yl-phthalimide (**36**) 45
Chloro-pent-4-en-1-yl-phthalimide (**37**) 45
Bromo-pent-4-en-1-yl-phthalimide (**38**) 47
Trifluoromethylsulfuryl-pent-4-en-1-yl-phthalimide (**39**) 47
Phenylacetylenyl-4-octene (**40**) 48
Synthesis of alkene starting materials 49
Nicotinic acid-derived alkene **26** 49
Cinchophene-derived alkene **27** 50
Lithocholic acid-derived alkene **28** 51
Epiandrosterone-derived alkene **29** 52
Piperidine-derived alkene **30** 53
Fluazinam-derived alkene **31** 53

Probenecid-derived alkene **32** 54

Bicalutamide-derived alkene **33** 55

Synthesis cyclo-adduct: thianthrenium dication 56

trans-4-Octene-derived thianthrenium dication **2-INT** 56

cis-4-Octene-derived thianthrenium dication **3-INT** 56

Reactivity studies on cycloadducts: thianthrenium dications 57

Studies on the formation of **2-TT** from trans-4-octene by NMR 57

Synthesis of **2-TT** from **2-INT** 59

Synthesis of **3-TT** from **3-INT** 60

Formation and stability of trans-4-octene adduct 61

Attempt at retrocyclization and trapping with o-xylene 62

Attempt at retrocyclization by vacuum pyrolysis 62

Studies on thianthrenium dication formation in solution 63

Cyclic Voltammetry 63

Constant Potential UV-vis Spectroscopy 64

Density Functional Theory calculations 65

Methods 65

DFT evaluation on disulfonium species 66

Discussion on six-membered and bicyclo[2.2.0] disulfonium species 68

Discussion on elimination mechanism 68

References 69

SPECTROSCOPIC DATA 70

trans-4-Octene-derived thianthrenium salt **2-TT** 70

^{1}H NMR of trans-4-octene-derived thianthrenium salt **2-TT** 70

^{13}C NMR of trans-4-octene-derived thianthrenium salt **2-TT** 71

^{19}F NMR of trans-4-octene-derived thianthrenium salt **2-TT** 72

cis-4-Octene-derived thianthrenium salt **3-TT** 73

^{1}H NMR of cis-4-octene-derived thianthrenium salt **3-TT (*E*)** 73

^{13}C NMR of cis-4-octene-derived thianthrenium salt **3-TT** 74

^{19}F NMR of cis-4-octene-derived thianthrenium salt **3-TT** 75

1-Octene-derived thianthrenium salt **4-TT** 76

^{1}H NMR of 1-octene-derived thianthrenium salt **4-TT (*E*)** 76

^{13}C NMR of 1-octene-derived thianthrenium salt **4-TT** 77

^{19}F NMR of 1-octene-derived thianthrenium salt **4-TT** 78

1,6-Heptadiene-derived thianthrenium salt **5-TT** 79

^{1}H NMR of 1,6-heptadiene-derived thianthrenium salt **5-TT (*E*)** 79

^{13}C NMR of 1,6-heptadiene-derived thianthrenium salt **5-TT** 80

^{19}F NMR of 1,6-heptadiene-derived thianthrenium salt **5-TT** 81

(-)-2-Vinylnorbornane-derived thianthrenium salt **6-TT** 82

^{1}H NMR of (-)-2-vinylnorbornane-derived thianthrenium salt **6-TT (*E*)** 82

^{13}C NMR of (-)-2-vinylnorbornane-derived thianthrenium salt **6-TT** 83

^{19}F NMR of (-)-2-vinylnorbornane-derived thianthrenium salt **6-TT** 84

Vinylcyclooctane-derived thianthrenium salt **7-TT** 85

^{1}H NMR of vinylcyclooctane-derived thianthrenium salt **7-TT** 85

^{13}C NMR of vinylcyclooctane-derived thianthrenium salt **7-TT** 86

^{19}F NMR of vinylcyclooctane-derived thianthrenium salt **7-TT** 87

8-Brom-1-octene-derived thianthrenium salt **8-TT** 88

^{1}H NMR of 8-brom-1-octene-derived thianthrenium salt **8-TT (*E*)** 88

^{13}C NMR of 8-brom-1-octene-derived thianthrenium salt **8-TT** 89

^{19}F NMR of 8-brom-1-octene-derived thianthrenium salt **8-TT** 90

10-Undecen-1-ol-derived thianthrenium salts **9-TT(OTFA)** 91

^{1}H NMR of 10-undecen-1-ol-derived thianthrenium salts **9-TT(OTFA) (*E*)** 91

^{13}C NMR of 10-undecen-1-ol-derived thianthrenium salts **9-TT(OTFA)** 92

^{19}F NMR of 10-undecen-1-ol-derived thianthrenium salts **9-TT(OTFA)** 93

10-Undecen-1-ol-derived thianthrenium salts **9-TT(OH)** 94

^{1}H NMR of 10-undecen-1-ol-derived thianthrenium salts **9-TT(OH)** 94

^{13}C NMR of 10-undecen-1-ol-derived thianthrenium salts **9-TT(OH)** 95

^{19}F NMR of 10-undecen-1-ol-derived thianthrenium salts **9-TT(OH)** 96

4-Phenyl-1-butene-derived thianthrenium salt **10-TT** ... 97

^{1}H NMR of 4-phenyl-1-butene-derived thianthrenium salt **10-TT** ... 97

^{13}C NMR of 4-phenyl-1-butene-derived thianthrenium salt **10-TT** ... 98

^{19}F NMR of 4-phenyl-1-butene-derived thianthrenium salt **10-TT** ... 99

Pent-4-en-1-yl-phthalimide-derived thianthrenium salt **11-TT** ... 100

^{1}H NMR of pent-4-en-1-yl-phthalimide-derived thianthrenium salt **11-TT** ... 100

^{13}C NMR of pent-4-en-1-yl-phthalimide-derived thianthrenium salt **11-TT** ... 101

^{19}F NMR of pent-4-en-1-yl-phthalimide-derived thianthrenium salt **11-TT** ... 102

Allylbenzol-derived thianthrenium salt **12-TT** ... 103

^{1}H NMR of allylbenzol-derived thianthrenium salt **12-TT** ... 103

^{13}C NMR of allylbenzol-derived thianthrenium salt **12-TT** ... 104

^{19}F NMR of allylbenzol-derived thianthrenium salt **12-TT** ... 105

1-Allyl-4-(trifluormethyl)-benzol-derived thianthrenium salt **13-TT** ... 106

^{1}H NMR of 1-allyl-4-(trifluormethyl)-benzol-derived thianthrenium salt **13-TT** ... 106

^{13}C NMR of 1-allyl-4-(trifluormethyl)-benzol-derived thianthrenium salt **13-TT** ... 107

^{19}F NMR of 1-allyl-4-(trifluormethyl)-benzol-derived thianthrenium salt **13-TT** ... 108

Cyclododecene-derived thianthrenium salt **14-TT** ... 109

^{1}H NMR of cyclododecene-derived thianthrenium salt **14-TT** ... 109

^{13}C NMR of cyclododecene-derived thianthrenium salt **14-TT** ... 110

^{19}F NMR of cyclododecene-derived thianthrenium salt **14-TT** ... 111

(+)-Rosenoxide-derived thianthrenium salt **15-TT** ... 112

^{1}H NMR of (+)-rosenoxide-derived thianthrenium salt **15-TT** ... 112

^{13}C NMR of (+)-rosenoxide-derived thianthrenium salt **15-TT** ... 113

^{19}F NMR of (+)-rosenoxide-derived thianthrenium salt **15-TT** ... 114

3,4-Dihydro-2*H*-pyrane-derived thianthrenium salt **16-TT** ... 115

^{1}H NMR of 3,4-dihydro-2*H*-pyrane-derived thianthrenium salt **16-TT** ... 115

^{13}C NMR of 3,4-dihydro-2*H*-pyrane-derived thianthrenium salt **16-TT** ... 116

^{19}F NMR of 3,4-dihydro-2*H*-pyrane-derived thianthrenium salt **16-TT** ... 117

N-(But-3-en-1-yl)benzamide-derived thianthrenium salt **17-TT** ... 118

^{1}H NMR of *N*-(but-3-en-1-yl)benzamide-derived thianthrenium salt **17-TT** 118

^{13}C NMR of *N*-(but-3-en-1-yl)benzamide-derived thianthrenium salt **17-TT** 119

^{19}F NMR of *N*-(but-3-en-1-yl)benzamide-derived thianthrenium salt **17-TT** 120

Cyclooctene-derived thianthrenium salt **18-TT** 121

^{1}H NMR of cyclooctene-derived thianthrenium salt **18-TT** 121

^{13}C NMR of cyclooctene-derived thianthrenium salt **18-TT** 122

^{19}F NMR of cyclooctene-derived thianthrenium salt **18-TT** 123

Cycloheptene-derived thianthrenium salt **19-TT** 124

^{1}H NMR of cycloheptene-derived thianthrenium salt **19-TT** 124

^{13}C NMR of cycloheptene-derived thianthrenium salt **19-TT** 125

^{19}F NMR of cycloheptene-derived thianthrenium salt **19-TT** 126

Cyclohexene-derived thianthrenium salt **20-TT** 127

^{1}H NMR of cyclohexene-derived thianthrenium salt **20-TT** 127

^{13}C NMR of cyclohexene-derived thianthrenium salt **20-TT** 128

^{19}F NMR of cyclohexene-derived thianthrenium salt **20-TT** 129

Cyclopentene-derived thianthrenium salt **21-TT** 130

^{1}H NMR of cyclopentene-derived thianthrenium salt **21-TT** 130

^{13}C NMR of cyclopentene-derived thianthrenium salt **21-TT** 131

^{19}F NMR of cyclopentene-derived thianthrenium salt **21-TT** 132

(*E,E,E*)-1,5,9-Cyclododecatriene-derived thianthrenium salt **22-TT** 133

^{1}H NMR of (*E,E,E*)-1,5,9-cyclododecatriene-derived thianthrenium salt **22-TT** 133

^{13}C NMR of (*E,E,E*)-1,5,9-cyclododecatriene-derived thianthrenium salt **22-TT** 134

^{19}F NMR of (*E,E,E*)-1,5,9-cyclododecatriene-derived thianthrenium salt **22-TT** 135

1,9-Cyclohexadecadiene-derived thianthrenium salt **23-TT** 136

^{1}H NMR of 1,9-cyclohexadecadiene-derived thianthrenium salt **23-TT** 136

^{13}C NMR of 1,9-cyclohexadecadiene-derived thianthrenium salt **23-TT** 137

^{19}F NMR of 1,9-cyclohexadecadiene-derived thianthrenium salt **23-TT** 138

1,5-Cyclooctadiene-derived thianthrenium salt **24-TT** 139

^{1}H NMR of 1,5-cyclooctadiene-derived thianthrenium salt **24-TT** 139

^{13}C NMR of 1,5-cyclooctadiene-derived thianthrenium salt **24-TT** 140

^{19}F NMR of 1,5-cyclooctadiene-derived thianthrenium salt **24-TT** 141

Tricyclo[6.2.1.02,7]undeca-4-ene-derived thianthrenium salt **25-TT** 142

^{1}H NMR of tricyclo[6.2.1.02,7]undeca-4-ene-derived thianthrenium salt **25-TT** 142

^{13}C NMR of tricyclo[6.2.1.02,7]undeca-4-ene-derived thianthrenium salt **25-TT** 143

^{19}F NMR of tricyclo[6.2.1.02,7]undeca-4-ene-derived thianthrenium salt **25-TT** 144

Nicotinic acid-derived thianthrenium salt **26-TT** 145

^{1}H NMR of nicotinic acid-derived thianthrenium salt **26-TT(*E*)** 145

^{13}C NMR of nicotinic acid-derived thianthrenium salt **26-TT** 146

^{19}F NMR of nicotinic acid-derived thianthrenium salt **26-TT** 147

Cinchophene-derived thianthrenium salt **27-TT** 148

^{1}H NMR of cinchophene-derived thianthrenium salt **27-TT** 148

^{13}C NMR of cinchophene-derived thianthrenium salt **27-TT** 149

^{19}F NMR of cinchophene-derived thianthrenium salt **27-TT** 150

Lithocholic acid-derived thianthrenium salt **28-TT** 151

^{1}H NMR of lithocholic acid-derived thianthrenium salt **28-TT** 151

^{13}C NMR of lithocholic acid-derived thianthrenium salt **28-TT** 152

^{19}F NMR of lithocholic acid-derived thianthrenium salt **28-TT** 153

Epiandrosterone-derived thianthrenium salt **29-TT** 154

^{1}H NMR of epiandrosterone-derived thianthrenium salt **29-TT** 154

^{13}C NMR of epiandrosterone-derived thianthrenium salt **29-TT** 155

^{19}F NMR of epiandrosterone-derived thianthrenium salt **29-TT** 156

Piperidine-derived thianthrenium salt **30-TT** 157

^{1}H NMR of piperidine-derived thianthrenium salt **30-TT** 157

^{13}C NMR of piperidine-derived thianthrenium salt **30-TT** 158

^{19}F NMR of piperidine-derived thianthrenium salt **30-TT** 158

Fluazinam-derived thianthrenium salt **31-TT** 160

^{1}H NMR of Fluazinam-derived thianthrenium salt **31-TT** 160

^{13}C NMR of Fluazinam-derived thianthrenium salt **31-TT** 161

^{19}F NMR of Fluazinam-derived thianthrenium salt **31-TT** 162

Probenecid-derived thianthrenium salt **32-TT** 163

^{1}H NMR of Probenecid-derived thianthrenium salt **32-TT** 163

^{13}C NMR of Probenecid-derived thianthrenium salt **32-TT** 164

^{19}F NMR of Probenecid-derived thianthrenium salt **32-TT** 164

Bicalutamide-derived thianthrenium salt **33-TT** 166

^{1}H NMR of Bicalutamide-derived thianthrenium salt **33-TT** 166

^{13}C NMR of Bicalutamide-derived thianthrenium salt **33-TT** 167

^{19}F NMR of Bicalutamide-derived thianthrenium salt **33-TT** 167

Cyclopropyl-pent-4-en-1-yl-phthalimide (**34**) 169

^{1}H NMR of cyclopropyl-pent-4-en-1-yl-phthalimide (**34**) 169

^{13}C NMR of cyclopropyl-pent-4-en-1-yl-phthalimide (**34**) 170

Phenylacetylenyl-pent-4-en-1-yl-phthalimide (**35**) 171

^{1}H NMR of phenylacetylenyl-pent-4-en-1-yl-phthalimide (**35**) 171

^{13}C NMR of phenylacetylenyl-pent-4-en-1-yl-phthalimide (**35**) 172

Vinylnaphthalenyl-pent-4-en-1-yl-phthalimide (**36**) 173

^{1}H NMR of vinylnaphthalenyl-pent-4-en-1-yl-phthalimide (**36**) 173

^{13}C NMR of vinylnaphthalenyl-pent-4-en-1-yl-phthalimide (**36**) 174

Chloro-pent-4-en-1-yl-phthalimide (**37**) 175

^{1}H NMR of chloro-pent-4-en-1-yl-phthalimide (**37**) 175

^{13}C NMR of chloro-pent-4-en-1-yl-phthalimide (**37**) 176

Bromo-pent-4-en-1-yl-phthalimide (**38**) 177

^{1}H NMR of bromo-pent-4-en-1-yl-phthalimide (**38**) 177

^{13}C NMR of bromo-pent-4-en-1-yl-phthalimide (**38**) 178

Trifluoromethylsulfuryl-pent-4-en-1-yl-phthalimide (**39**) 179

^{1}H NMR of trifluoromethylsulfuryl-pent-4-en-1-yl-phthalimide (**39**) 179

^{13}C NMR of trifluoromethylsulfuryl-pent-4-en-1-yl-phthalimide (**39**) 180

^{19}F NMR of trifluoromethylsulfuryl-pent-4-en-1-yl-phthalimide (**39**) 181

Phenylacetylenyl-4-octene (**40-*Z***) 182

^{1}H NMR of phenylacetylenyl-4-octene (**40-*Z*)** 182

^{13}C NMR of phenylacetylenyl-4-octene (**40-*Z***) 183

Phenylacetylenyl-4-octene (**40-*E***) 184

^{1}H NMR of phenylacetylenyl-4-octene (**40-*E***) 184

^{13}C NMR of phenylacetylenyl-4-octene (**40-*E***) 185

Nicotinic acid-derived alkene **26** 186

^{1}H NMR of Nicotinic acid-derived alkene **26** 186

^{13}C NMR of Nicotinic acid-derived alkene **26** 187

Cinchophene-derived alkene **27** 188

^{1}H NMR of cinchophene-derived alkene **27** 188

^{13}C NMR of cinchophene-derived alkene **27** 189

Lithocholic acid-derived alkene **28** 190

^{1}H NMR of lithocholic acid-derived alkene **28** 190

^{13}C NMR of lithocholic acid-derived alkene **28** 191

Epiandrosterone-derived alkene **29** 192

^{1}H NMR of epiandrosterone-derived alkene **29** 192

^{13}C NMR of epiandrosterone-derived alkene **29** 193

Piperidine-derived alkene **30** 196

^{1}H NMR of piperidine-derived alkene **30** 196

^{13}C NMR of piperidine-derived alkene **30** 197

Flazinam-derived alkene **31** 197

^{1}H NMR of Fluazinam-derived alkene **31** 198

^{13}C NMR of Fluazinam-derived alkene **31** 199

^{19}F NMR of Fluazinam-derived alkene **31** 200

Probenecid-derived alkene **32** 201

^{1}H NMR of Probenecid-derived alkene **32** 201

^{13}C NMR of Probenecid-derived alkene **32** 202

Bicalutamide-derived alkene **33** 203

^{1}H NMR of Bicalutamide-derived alkene **33** 203

^{13}C NMR of Bicalutamide-derived alkene **33** 204

^{19}F NMR of Bicalutamide-derived alkene **33** 205

trans-4-Octene-derived thianthrenium dication **2-INT** 206

^{1}H NMR of trans-4-octene-derived thianthrenium dication **2-INT** 206

^{13}C NMR of trans-4-octene-derived thianthrenium dication **2-INT** 207

^{19}F NMR of trans-4-octene-derived thianthrenium dication **2-INT** 208

cis-4-Octene-derived thianthrenium dication **3-INT** 209

^{1}H NMR of cis-4-octene-derived thianthrenium dication **3-INT** 209

^{13}C NMR of cis-4-octene-derived thianthrenium dication **3-INT** 210

^{19}F NMR of cis-4-octene-derived thianthrenium dication **3-INT** 211

MATERIALS AND METHODS

All reactions were carried out under ambient atmosphere unless otherwise stated. High-resolution mass spectra were obtained using *Q Exactive Plus* from *Thermo*. Concentration under reduced pressure was performed by rotary evaporation at 25–35 °C at an appropriate pressure. Purified compounds were further dried under vacuum (10^{-6}–10^{-3} bar).

Solvents

Anhydrous acetonitrile and tetrahydrofuran were obtained from Phoenix Solvent Drying Systems. All deuterated solvents were purchased from Euriso-Top.

Chromatography

Thin layer chromatography (TLC) was performed using EMD TLC plates pre-coated with 250 μm thickness silica gel 60 F_{254} plates and visualized by irradiation UV light or by placing the TLC plate into an iodine chamber for a few minutes. Flash chromatography was performed using silica gel (40–63 µm particle size) purchased from Geduran.

Spectroscopy and Instruments

NMR spectra were recorded on a *Bruker Ascend™ 500* spectrometer operating at 500 MHz, 471 MHz, and 126 MHz, for ^{1}H, ^{19}F, and ^{13}C acquisitions, respectively. Chemical shifts are reported in ppm with the solvent residual peak as the internal standard. For ^{1}H NMR: $CDCl_3$, δ 7.26; CD_2Cl_2, δ 5.32; For ^{13}C NMR: $CDCl_3$, δ 77.16; CD_2Cl_2, δ 53.84. ^{19}F NMR spectra were referenced using a unified chemical shift scale based on the ^{1}H resonance of tetramethylsilane (1% v/v solution in the respective solvent). Data is reported as follows: s = singlet, d = doublet, t = triplet, q = quartet, quin = quintet, sext = sextet, sept = septet, m = multiplet, bs = broad singlet; coupling constants in Hz; integration. The *E*/*Z* ratio was determined by ^{1}H NMR, quantified by peak integrations of the characteristic *E*/*Z* alkenyl protons.

Starting materials

All substrates were used as received from commercial suppliers, unless otherwise stated. Chemicals were purchased from *Sigma-Aldrich*, *Chempur*, *TCI*, or *Alfa Aesar*. Tiny amount of trifluoroacetate counterion residues in the alkenyl thianthrenium salts do not interfere the following up transformations.

EXPERIMENTAL DATA

Thianthrenation of alkenes

General procedure

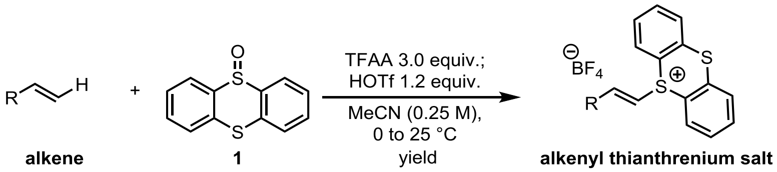

Under ambient atmosphere, a 20 mL borosilicate vial equipped with a magnetic stir bar was charged with alkene (0.500 mmol, 1.00 equiv.), thianthrene S-oxide (**1**) (120 mg, 0.517 mmol, 1.03 equiv.), and MeCN (2.0 mL, c = 0.25 M). After cooling to 0°C, trifluoroacetic anhydride (0.21 mL, 0.31 g, 1.5 mmol, 3.0 equiv.) was added dropwise within 30 seconds, followed by dropwise addition of HOTf (52 µL, 88 mg, 0.59 mmol, 1.2 equiv.) within 10 seconds. After stirring the lilac mixture at 0 °C for 60 min followed by stirring at 25 °C for 30 min, the resulting light pink mixture was concentrated under reduced pressure and subsequently diluted with CH_2Cl_2 (10 mL). The CH_2Cl_2 solution was poured onto a saturated aqueous $NaHCO_3$ solution (ca. 20 mL). The combined mixture was poured into a separatory funnel, and the layers were separated. The CH_2Cl_2 layer was collected, and the aqueous layer was further extracted with CH_2Cl_2 (2 × ca. 10 mL). The combined CH_2Cl_2 solution was washed with aqueous $NaBF_4$ solution (2 × ca. 20 mL, 5 % w/w). The CH_2Cl_2 layer was dried over Na_2SO_4, filtered, and the solvent was removed under reduced pressure. The residue was purified by chromatography on silica gel eluting with CH_2Cl_2/*i*-PrOH to afford the alkenyl thianthrenium salt.

trans-4-Octene-derived thianthrenium salt 2-TT

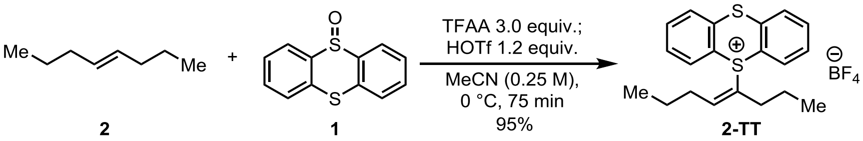

Under ambient atmosphere, a 20 mL borosilicate vial equipped with a magnetic stir bar was charged with trans-4-octene (78.5 µL, 56.1 mg, 0.500 mmol, 1.00 equiv.), thianthrene S-oxide (**1**) (120 mg, 0.517 mmol, 1.03 equiv.), and MeCN (2.0 mL, c = 0.25 M). After cooling to 0°C, trifluoroacetic anhydride (0.21 mL, 0.31 g, 1.5 mmol, 3.0 equiv.) was added dropwise within 30 seconds, followed by dropwise addition of HOTf (52 µL, 88 mg, 0.59 mmol, 1.2 equiv.) within 10 seconds. After stirring the lilac mixture at 0 °C for 75 min, the resulting light pink mixture was concentrated under reduced pressure and subsequently diluted with CH_2Cl_2 (10 mL). The CH_2Cl_2 solution was poured onto a saturated aqueous $NaHCO_3$ solution (ca. 20 mL). The combined mixture was poured into a separatory funnel, and the layers were separated. The CH_2Cl_2 layer was collected, and the aqueous layer was further extracted with CH_2Cl_2 (2 × ca. 10 mL). The combined CH_2Cl_2 solution was washed with aqueous $NaBF_4$ solution (2 × ca. 20 mL, 5 % w/w). The CH_2Cl_2 layer was dried

over Na_2SO_4, filtered, and the solvent was removed under reduced pressure. The residue was purified by chromatography on silica gel eluting with CH_2Cl_2/*i*-PrOH (100:1, v/v). The product-containing fractions were collected and concentrated under reduced pressure. The residue was further dried in vacuo to afford **2-TT** (*E*/*Z* < 50/1, 197.5 mg, 477 µmol, 95 %) as a colorless solid.

R*f* = 0.46 (CH_2Cl_2/MeOH, 15:1, v/v).

NMR Spectroscopy:

^{1}H NMR (500 MHz, CD_2Cl_2, 298 K, δ): 8.01 (dd, *J* = 8.0, 1.3 Hz, 2H), 7.84 – 7.76 (m, 4H), 7.69 (ddd, *J* = 8.1, 7.1, 1.7 Hz, 2H), 6.42 (tt, *J* = 7.6, 1.4 Hz, 1H), 2.72 – 2.65 (m, 2H), 2.19 (m, 2H), 1.55 (h, *J* = 7.3 Hz, 2H), 1.35 (h, *J* = 7.3 Hz, 2H), 1.02 (t, *J* = 7.3 Hz, 3H), 0.76 (t, *J* = 7.3 Hz, 3H).

^{13}C NMR {^{1}H} (126 MHz, CD_2Cl_2, 298 K, δ): 149.4, 136.7, 135.1, 133.7, 131.4, 130.7, 130.5, 117.6, 35.2, 32.9, 22.7, 22.3, 13.9, 13.6.

^{19}F NMR (471 MHz, CD_2Cl_2, 298 K, δ): –152.29 (bs), –152.34 (bs).

HRMS-ESI (m/z) calc'd. for $C_{20}H_{23}S_2^+$ $[M]^+$, 327.12357; found, 327.12306; deviation: +1.56 ppm.

cis-4-Octene-derived thianthrenium salt 3-TT

With 1.20 equiv. HOTf:

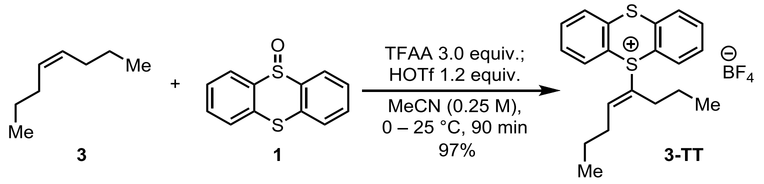

3 **1** **3-TT**

Under ambient atmosphere, a 20 mL borosilicate vial equipped with a magnetic stir bar was charged with cis-4-octene (77.8 µL, 56.1 mg, 0.500 mmol, 1.00 equiv.), thianthrene S-oxide (**1**) (120 mg, 0.517 mmol, 1.03 equiv.), and MeCN (2.0 mL, c = 0.25 M). After cooling to 0°C, trifluoroacetic anhydride (0.21 mL, 0.31 g, 1.5 mmol, 3.0 equiv.) was added dropwise within 30 seconds, followed by dropwise addition of HOTf (52 µL, 88 mg, 0.59 mmol, 1.2 equiv.) within 10 seconds. After stirring the lilac mixture at 0 °C for 60 min followed by stirring at 25 °C for 30 min, the resulting purple mixture was concentrated under reduced pressure and subsequently diluted with CH_2Cl_2 (10 mL). The CH_2Cl_2 solution was poured onto a saturated aqueous $NaHCO_3$ solution (ca. 20 mL). The combined mixture was poured into a separatory funnel, and the layers were separated. The CH_2Cl_2 layer was collected, and the aqueous layer was further extracted with CH_2Cl_2 (2 × ca. 10 mL). The combined CH_2Cl_2 solution was washed with aqueous $NaBF_4$ solution (2 × ca. 20 mL, 5 % w/w). The CH_2Cl_2 layer was dried over Na_2SO_4, filtered, and the solvent was removed under reduced pressure. The residue was purified by chromatography on silica gel eluting with CH_2Cl_2/*i*-PrOH (100:1, v/v). The product-containing fractions were collected and concentrated under reduced pressure. The residue was further dried in vacuo to afford **3-TT** (*E*/*Z* ≅ 17/1, 200.6 mg, 484 µmol, 97 %) as a colorless solid. **3-TT** was further purified by chromatography on silica gel eluting with CH_2Cl_2/*i*-PrOH (100:1, v/v), and fractions containing **3-TT(*E*)** were collected and concentrated under reduced pressure. The residue was further dried

in vacuo.

With 2.40 equiv. HOTf:

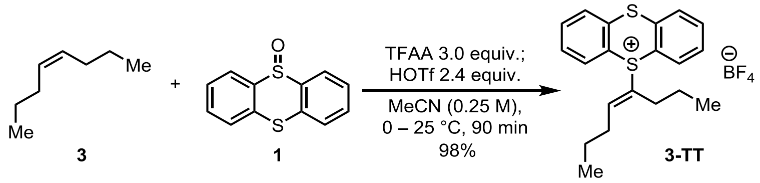

Under ambient atmosphere, a 20 mL borosilicate vial equipped with a magnetic stir bar was charged with cis-4-octene (77.8 µL, 56.1 mg, 0.500 mmol, 1.00 equiv.), thianthrene S-oxide (**1**) (120 mg, 0.517 mmol, 1.03 equiv.), and MeCN (2.0 mL, c = 0.25 M). After cooling to 0°C, trifluoroacetic anhydride (0.21 mL, 0.31 g, 1.5 mmol, 3.0 equiv.) was added dropwise within 30 seconds, followed by dropwise addition of HOTf (104 µL, 1766 mg, 1.18 mmol, 2.40 equiv.) within 10 seconds. After stirring the lilac mixture at 0 °C for 60 min followed by stirring at 25 °C for 30 min, the resulting purple mixture was concentrated under reduced pressure and subsequently diluted with CH_2Cl_2 (10 mL). The CH_2Cl_2 solution was poured onto a saturated aqueous $NaHCO_3$ solution (ca. 20 mL). The combined mixture was poured into a separatory funnel, and the layers were separated. The CH_2Cl_2 layer was collected, and the aqueous layer was further extracted with CH_2Cl_2 (2 × ca. 10 mL). The combined CH_2Cl_2 solution was washed with aqueous $NaBF_4$ solution (2 × ca. 20 mL, 5 % w/w). The CH_2Cl_2 layer was dried over Na_2SO_4, filtered, and the solvent was removed under reduced pressure. The residue was purified by chromatography on silica gel eluting with CH_2Cl_2/*i*-PrOH (100:1, v/v). The product-containing fractions were collected and concentrated under reduced pressure. The residue was further dried in vacuo to afford **3-TT** (*E*/*Z* > 49/1, 204.5 mg, 0.488 µmol, 98 %) as a colorless solid.

R*f* = 0.46 (CH_2Cl_2/MeOH, 15:1, v/v).

NMR Spectroscopy:

^{1}H NMR (500 MHz, $CDCl_3$, 298 K, δ): 8.46 (d, *J* = 7.7 Hz, 2H), 7.86 – 7.66 (m, 6H), 5.51 (t, *J* = 7.6 Hz, 1H), 2.13 (m, 4H), 1.30 (m, 4H), 0.81 (t, *J* =7.4 Hz, 6H).

^{13}C NMR {^{1}H} (126 MHz, CD_2Cl_2, 298 K, δ): 142.7, 137.4, 135.5, 135.3, 130.8, 130.6, 123.6, 117.0, 31.9, 30.2, 22.1, 21.9, 13.8, 13.7.

^{19}F NMR (471 MHz, CD_2Cl_2, 298 K, δ): –151.69 (bs), –151.74 (bs).

HRMS-ESI (m/z) calc'd. for $C_{20}H_{23}S_2^+$ $[M]^+$, 327.12357; found, 327.12346; deviation: +0.34 ppm.

1-Octene-derived thianthrenium salt 4-TT

With 1.20 equiv. HOTf:

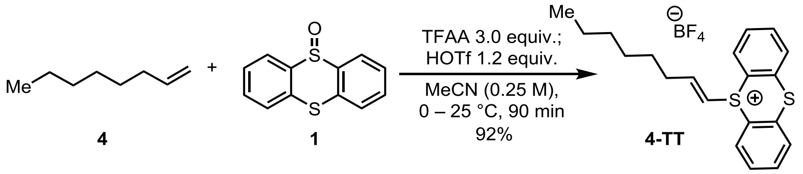

Under ambient atmosphere, a 20 mL borosilicate vial equipped with a magnetic stir bar was charged with 1-octene (78.5 µL, 56.1 mg, 0.500 mmol, 1.00 equiv.), thianthrene S-oxide (**1**) (120 mg, 0.517 mmol, 1.03 equiv.), and MeCN (2.0 mL, c = 0.25 M). After cooling to 0°C, trifluoroacetic anhydride (0.21 -mL, 0.31 g, 1.5 mmol, 3.0 equiv.) was added dropwise within 30 seconds, followed by dropwise addition of HOTf (52 µL, 88 mg, 0.59 mmol, 1.2 equiv.) within 10 seconds. After stirring the lilac mixture at 0 °C for 60 min followed by stirring at 25 °C for 30 min, the resulting purple mixture was concentrated under reduced pressure and subsequently diluted with CH_2Cl_2 (10 mL). The CH_2Cl_2 solution was poured onto a saturated aqueous $NaHCO_3$ solution (ca. 20 mL). The combined mixture was poured into a separatory funnel, and the layers were separated. The CH_2Cl_2 layer was collected, and the aqueous layer was further extracted with CH_2Cl_2 (2 × ca. 10 mL). The combined CH_2Cl_2 solution was washed with aqueous $NaBF_4$ solution (2 × ca. 20 mL, 5 % w/w). The CH_2Cl_2 layer was dried over Na_2SO_4, filtered, and the solvent was removed under reduced pressure. The residue was purified by chromatography on silica gel eluting with CH_2Cl_2/*i*-PrOH (100:1, v/v). The product-containing fractions were collected and concentrated under reduced pressure. The residue was further dried in vacuo to afford **4-TT** (*E*/*Z* ≅ 13/1, 191.2 mg, 462 µmol, 92 %) as a colorless sticky oil. **4-TT** was further purified by chromatography on silica gel eluting with CH_2Cl_2/*i*-PrOH (100:1, v/v), and fractions containing **4-TT(*E*)** were collected and concentrated under reduced pressure. The residue was further dried in vacuo.

With 2.40 equiv. HOTf:

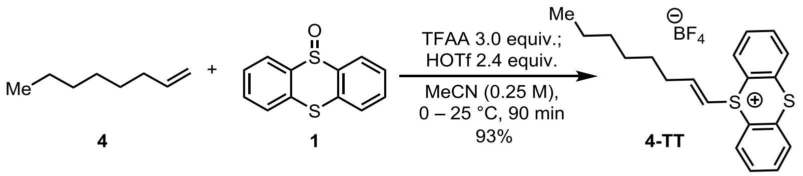

Under ambient atmosphere, a 20 mL borosilicate vial equipped with a magnetic stir bar was charged with 1-octene (78.5 µL, 56.1 mg, 0.500 mmol, 1.00 equiv.), thianthrene S-oxide (**1**) (120 mg, 0.517 mmol, 1.03 equiv.), and MeCN (2.0 mL, c = 0.25 M). After cooling to 0°C, trifluoroacetic anhydride (0.21 -mL, 0.31 g, 1.5 mmol, 3.0 equiv.) was added dropwise within 30 seconds, followed by dropwise addition of HOTf (104 µL, 1766 mg, 1.18 mmol, 2.40 equiv.) within 10 seconds. After stirring the lilac mixture at 0 °C for 60 min followed by stirring at 25 °C for 30 min, the resulting purple mixture was concentrated under reduced pressure and subsequently diluted with CH_2Cl_2 (10 mL). The CH_2Cl_2 solution was cooled to 0 °C for 5 min and poured onto a saturated aqueous $NaHCO_3$ solution (ca. 20 mL) which was prechilled to 0 °C for 5 min. The combined mixture was stirred at 0 °C for 5 min and poured into a separatory funnel, and the layers were separated. The CH_2Cl_2 layer was collected, and the aqueous layer was further extracted with CH_2Cl_2 (2 × ca. 10 mL). The combined CH_2Cl_2 solution was washed with aqueous $NaBF_4$ solution (2 × ca. 20 mL, 5 % w/w). The CH_2Cl_2 layer was dried over Na_2SO_4, filtered, and the solvent was removed under reduced pressure. The residue was purified by chromatography on silica gel eluting with CH_2Cl_2/*i*-PrOH (100:1, v/v). The product-containing fractions were collected and concentrated under reduced pressure. The residue was further dried in vacuo to afford **4-TT** (*E*/*Z* ≅ 20/1, 192.3 mg, 464 µmol, 93 %) as a colorless sticky oil.

R*f* = 0.46 (CH_2Cl_2/MeOH, 15:1, v/v).

NMR Spectroscopy:

^{1}H NMR (500 MHz, CD_2Cl_2, 298 K, δ): 8.24 (d, *J* = 7.9 Hz, 2H), 7.89 (d, *J* = 8.0 Hz, 2H), 7.78 (t, *J* = 7.8 Hz, 2H), 7.71 (t, *J* = 7.7 Hz, 2H), 7.10 (dt, *J* = 14.9, 7.5 Hz, 1H), 6.55 (dt, *J* = 14.8, 1.5 Hz, 1H), 2.27 (m, 2H), 1.43 – 1.35 (m, 2H), 1.31 – 1.12 (m, 6H), 0.87 – 0.76 (m, 3H).

^{13}C NMR {^{1}H} (126 MHz, CD_2Cl_2, 298 K, δ): 157.4, 136.3, 135.1, 133.7, 130.8, 130.7, 120.8, 109.8, 33.8, 31.8, 29.1, 27.7, 22.9, 14.2.

^{19}F NMR (471 MHz, CD_2Cl_2, 298 K, δ): –151.05 (bs), –151.11 (bs).

HRMS-ESI (m/z) calc'd. for $C_{20}H_{23}S_2^+$ $[M]^+$, 327.12357; found, 327.12302; deviation: +1.68 ppm.

1,6-Heptadiene-derived thianthrenium salt 5-TT

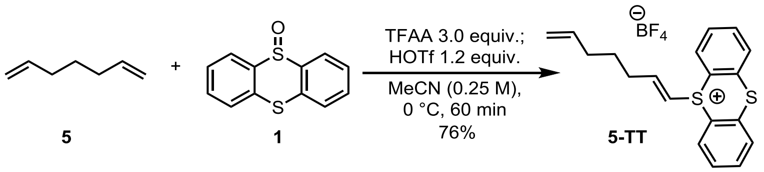

Under ambient atmosphere, a 20 mL borosilicate vial equipped with a magnetic stir bar was charged with 1,6-heptadiene (67.3 µL, 48.1 mg, 0.500 mmol, 1.00 equiv.), thianthrene S-oxide (**1**) (120 mg, 0.517 mmol, 1.03 equiv.), and MeCN (2.0 mL, c = 0.25 M). After cooling to 0°C, trifluoroacetic anhydride (0.21 mL, 0.31 g, 1.5 mmol, 3.0 equiv.) was added dropwise within 30 seconds, followed by dropwise addition of HOTf (52 µL, 88 mg, 0.59 mmol, 1.2 equiv.) within 10 seconds. After stirring the lilac mixture at 0 °C for 60 min, the resulting yellow mixture was concentrated under reduced pressure and subsequently diluted with CH_2Cl_2 (10 mL). The CH_2Cl_2 solution was poured onto a saturated aqueous $NaHCO_3$ solution (ca. 20 mL). The combined mixture was poured into a separatory funnel, and the layers were separated. The CH_2Cl_2 layer was collected, and the aqueous layer was further extracted with CH_2Cl_2 (2 × ca. 10 mL). The combined CH_2Cl_2 solution was washed with aqueous $NaBF_4$ solution (2 × ca. 20 mL, 5 % w/w). The CH_2Cl_2 layer was dried over Na_2SO_4, filtered, and the solvent was removed under reduced pressure. The residue was purified by chromatography on silica gel eluting with CH_2Cl_2/*i*-PrOH (100:1, v/v). The product-containing fractions were collected and concentrated under reduced pressure. The residue was further dried in vacuo to afford **5-TT** (*E*/*Z* ≅ 17/1, 151.6 mg, 381 µmol, 76 %) as a colorless sticky oil. **5-TT** was further purified by chromatography on silica gel eluting with CH_2Cl_2/*i*-PrOH (100:1, v/v), and fractions containing **5-TT(*E*)** were collected and concentrated under reduced pressure. The residue was further dried in vacuo.

R*f* = 0.46 (CH_2Cl_2/MeOH, 15:1, v/v).

NMR Spectroscopy:

^{1}H NMR (500 MHz, CD_2Cl_2, 298 K, δ): 8.25 (d, *J* = 7.9 Hz, 2H), 7.89 (d, *J* = 7.9 Hz, 2H), 7.78 (t, *J* = 7.5 Hz, 2H), 7.71 (t, *J* = 7.7 Hz, 2H), 7.10 (dt, *J* = 14.9, 7.0 Hz, 1H), 6.55 (dt, *J* = 14.8, 1.5 Hz, 1H), 5.71 (m, 1H), 4.94 – 4.91 (m, 1H), 4.90 – 4.92 (m, 1H), 2.30 – 2.23 (m, 2H), 2.01 (q, *J* = 7.2

Hz, 2H), 1.50 (p, J = 7.4 Hz, 2H).

^{13}C NMR {^{1}H} (126 MHz, CD_2Cl_2, 298 K, δ): 156.9, 138.0, 136.3, 135.1, 133.8, 130.8, 130.7, 120.7, 115.8, 110.1, 33.3, 33.1, 26.9.

^{19}F NMR (471 MHz, CD_2Cl_2, 298 K, δ): –150.98 (bs), –151.04 (bs).

HRMS-ESI (m/z) calc'd. for $C_{19}H_{19}S_2^+$ $[M]^+$, 311.09227; found, 311.09192; deviation: +1.13 ppm.

(-)-2-Vinylnorbornane-derived thianthrenium salt 6-TT

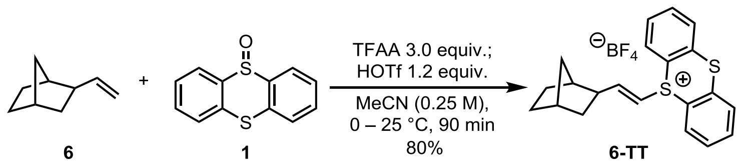

Under ambient atmosphere, a 20 mL borosilicate vial equipped with a magnetic stir bar was charged with (-)-2-vinylnorbornane (60.1 mg, 0.500 mmol, 1.00 equiv.), thianthrene S-oxide (**1**) (120 mg, 0.517 mmol, 1.03 equiv.), and MeCN (2.0 mL, c = 0.25 M). After cooling to 0°C, trifluoroacetic anhydride (0.21 mL, 0.31 g, 1.5 mmol, 3.0 equiv.) was added dropwise within 30 seconds, followed by dropwise addition of HOTf (52 µL, 88 mg, 0.59 mmol, 1.2 equiv.) within 10 seconds. After stirring the lilac mixture at 0 °C for 60 min followed by stirring at 25 °C for 30 min, the resulting purple mixture was concentrated under reduced pressure and subsequently diluted with CH_2Cl_2 (10 mL). The CH_2Cl_2 solution was poured onto a saturated aqueous $NaHCO_3$ solution (ca. 20 mL). The combined mixture was poured into a separatory funnel, and the layers were separated. The CH_2Cl_2 layer was collected, and the aqueous layer was further extracted with CH_2Cl_2 (2 × ca. 10 mL). The combined CH_2Cl_2 solution was washed with aqueous $NaBF_4$ solution (2 × ca. 20 mL, 5 % w/w). The CH_2Cl_2 layer was dried over Na_2SO_4, filtered, and the solvent was removed under reduced pressure. The residue was purified by chromatography on silica gel eluting with CH_2Cl_2/*i*-PrOH (100:1, v/v). The product-containing fractions were collected and concentrated under reduced pressure. The residue was further dried in vacuo to afford **6-TT** (*E*/*Z* ≅ 13/1, 170.1 mg, 401 µmol, 80 %) as a colorless solid. **6-TT** was further purified by chromatography on silica gel eluting with CH_2Cl_2/*i*-PrOH (100:1, v/v), and fractions containing **6-TT(*E*)** were collected and concentrated under reduced pressure. The residue was further dried in vacuo.

R_f = 0.46 (CH_2Cl_2/MeOH, 15:1, v/v).

NMR Spectroscopy:

^{1}H NMR (500 MHz, CD_2Cl_2, 298 K, δ): 8.23 (dt, J = 7.9, 1.3 Hz, 2H), 7.89 (dt, J = 7.8, 1.3 Hz, 2H), 7.77 (tt, J = 7.8, 1.4 Hz, 2H), 7.70 (tt, J = 7.6, 1.6 Hz, 2H), 6.98 (dd, J = 14.7, 8.5 Hz, 1H), 6.49 (dd, J = 14.7, 1.1 Hz, 1H), 2.33 – 2.25 (m, 2H), 2.14 – 2.09 (m, 1H), 1.53 – 1.44 (m, 3H), 1.35 – 1.27 (m, 2H), 1.22 – 1.12 (m, 3H).

^{13}C NMR {^{1}H} (126 MHz, CD_2Cl_2, 298 K, δ): 161.1, 136.3 (d, J = 2.8 Hz), 135.0, 133.7 (d, J = 3.7 Hz), 130.83, 130.75, 121.1 (d, J = 3.6 Hz), 107.7, 46.6, 42.6, 37.2, 37.0, 36.2, 29.9, 29.0.

^{19}F NMR (471 MHz, CD_2Cl_2, 298 K, δ): –151.00 (bs), –151.05 (bs).

HRMS-ESI (m/z) calc'd. for $C_{21}H_{21}S_2^+$ $[M]^+$, 337.10792; found, 337.10729; deviation: +1.87 ppm.

Vinylcyclooctane-derived thianthrenium salt 7-TT

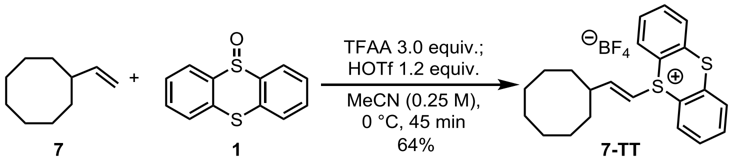

Under ambient atmosphere, a 20 mL borosilicate vial equipped with a magnetic stir bar was charged with vinylcyclooctane (69.1 mg, 0.500 mmol, 1.00 equiv.), thianthrene S-oxide (**1**) (120 mg, 0.517 mmol, 1.03 equiv.), and MeCN (2.0 mL, c = 0.25 M). After cooling to 0°C, trifluoroacetic anhydride (0.21 mL, 0.31 g, 1.5 mmol, 3.0 equiv.) was added dropwise within 30 seconds, followed by dropwise addition of HOTf (52 µL, 88 mg, 0.59 mmol, 1.2 equiv.) within 10 seconds. After stirring the lilac mixture at 0 °C for 45 min, the resulting purple mixture was concentrated under reduced pressure and subsequently diluted with CH_2Cl_2 (10 mL). The CH_2Cl_2 solution was poured onto a saturated aqueous $NaHCO_3$ solution (ca. 20 mL). The combined mixture was poured into a separatory funnel, and the layers were separated. The CH_2Cl_2 layer was collected, and the aqueous layer was further extracted with CH_2Cl_2 (2 × ca. 10 mL). The combined CH_2Cl_2 solution was washed with aqueous $NaBF_4$ solution (2 × ca. 20 mL, 5 % w/w). The CH_2Cl_2 layer was dried over Na_2SO_4, filtered, and the solvent was removed under reduced pressure. The residue was purified by chromatography on silica gel eluting with CH_2Cl_2/*i*-PrOH (100:1, v/v). The product-containing fractions were collected and concentrated under reduced pressure. The residue was further dried in vacuo to afford **7-TT** (*E*/*Z* > 50/1, 141.1 mg, 321 µmol, 64 %) as a colorless solid.

R*f* = 0.46 (CH_2Cl_2/MeOH, 15:1, v/v).

NMR Spectroscopy:

^{1}H NMR (500 MHz, CD_2Cl_2, 298 K, δ): 8.24 (dd, *J* = 7.9, 1.4 Hz, 2H), 7.89 (dd, *J* = 7.9, 1.4 Hz, 2H), 7.78 (td, *J* = 7.7, 1.4 Hz, 2H), 7.70 (td, *J* = 7.7, 1.3 Hz, 2H), 7.01 (dd, *J* = 14.9, 7.6 Hz, 1H), 6.47 (dd, *J* = 14.8, 1.3 Hz, 1H), 2.50 – 2.43 (m, 1H), 1.68 – 1.56 (m, 5H), 1.55 – 1.39 (m, 9H).

^{13}C NMR {^{1}H} (126 MHz, CD_2Cl_2, 298 K, δ): 162.4, 136.4, 135.0, 133.8, 130.9, 130.8, 121.0, 107.9, 42.8, 30.6, 27.3, 26.3, 25.2.

^{19}F NMR (471 MHz, CD_2Cl_2, 298 K, δ): –149.16 (bs), –149.22 (bs).

HRMS-ESI (m/z) calc'd. for $C_{22}H_{25}S_2^+$ $[M]^+$, 353.13922; found, 353.13868; deviation: +1.53 ppm.

8-Brom-1-octene-derived thianthrenium salt 8-TT

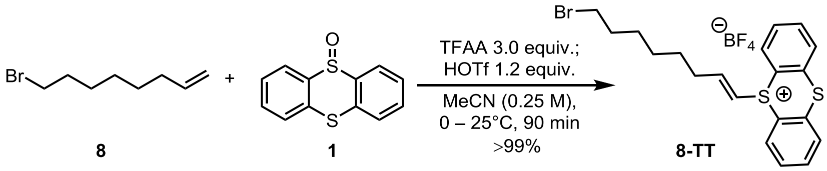

Under ambient atmosphere, a 20 mL borosilicate vial equipped with a magnetic stir bar was charged with 8-brom-1-octene (95.6 mg, 0.500 mmol, 1.00 equiv.), thianthrene S-oxide (**1**) (120 mg, 0.517 mmol, 1.03 equiv.), and MeCN (2.0 mL, c = 0.25 M). After cooling to 0°C, trifluoroacetic anhydride (0.21 mL, 0.31 g, 1.5 mmol, 3.0 equiv.) was added dropwise within 30 seconds, followed by dropwise addition of HOTf (52 µL, 88 mg, 0.59 mmol, 1.2 equiv.) within 10 seconds. After stirring the lilac mixture at 0 °C for 60 min followed by stirring at 25 °C for 30 min, the resulting purple mixture was concentrated under reduced pressure and subsequently diluted with CH_2Cl_2 (10 mL). The CH_2Cl_2 solution was poured onto a saturated aqueous $NaHCO_3$ solution (ca. 20 mL). The combined mixture was poured into a separatory funnel, and the layers were separated. The CH_2Cl_2 layer was collected, and the aqueous layer was further extracted with CH_2Cl_2 (2 × ca. 10 mL). The combined CH_2Cl_2 solution was washed with aqueous $NaBF_4$ solution (2 × ca. 20 mL, 5 % w/w). The CH_2Cl_2 layer was dried over Na_2SO_4, filtered, and the solvent was removed under reduced pressure. The residue was purified by chromatography on silica gel eluting with CH_2Cl_2/*i*-PrOH (100:1, v/v). The product-containing fractions were collected and concentrated under reduced pressure. The residue was further dried in vacuo to afford **8-TT** (*E*/*Z* ≅ 17/1, 245.8 mg, 500 µmol, >99 %) as a beige sticky oil. **8-TT** was further purified by chromatography on silica gel eluting with CH_2Cl_2/*i*-PrOH (100:1, v/v), and fractions containing **8-TT(*E*)** were collected and concentrated under reduced pressure. The residue was further dried in vacuo.

R*f* = 0.46 (CH_2Cl_2/MeOH, 15:1, v/v).

NMR Spectroscopy:

^{1}H NMR (500 MHz, CD_2Cl_2, 298 K, δ) 8.28 (dt, *J* = 7.9, 1.4 Hz, 2H), 7.93 (dt, *J* = 7.9, 1.4 Hz, 2H), 7.82 (ddt, *J* = 9.3, 7.8, 1.5 Hz, 2H), 7.74 (tt, *J* = 7.6, 1.4 Hz, 2H), 7.19 – 7.08 (m, 1H), 6.60 (dq, *J* = 14.8, 1.5 Hz, 1H), 3.40 (td, *J* = 6.8, 1.3 Hz, 2H), 2.35 – 2.28 (m, 2H), 1.88 – 1.78 (m, 2H), 1.51 – 1.44 (m, 2H), 1.44 – 1.36 (m, 2H), 1.33 – 1.26 (m, 2H).

^{13}C NMR {^{1}H} (126 MHz, CD_2Cl_2, 298 K, δ): 157.1, 136.4, 135.1, 133.8, 130.8, 130.7, 120.8, 110.0, 34.6, 33.6, 33.0, 28.5, 28.1, 27.5.

^{19}F NMR (471 MHz, CD_2Cl_2, 298 K, δ): –151.05 (bs), –151.11 (bs).

HRMS-ESI (m/z) calc'd. for $C_{20}H_{22}BrS_2^+$ $[M]^+$, 405.03409; found, 405.03362; deviation: +1.17 ppm.

10-Undecen-1-ol-derived thianthrenium salts 9-TT(OTFA) and 9-TT(OH)

With 1.20 equiv. HOTf:

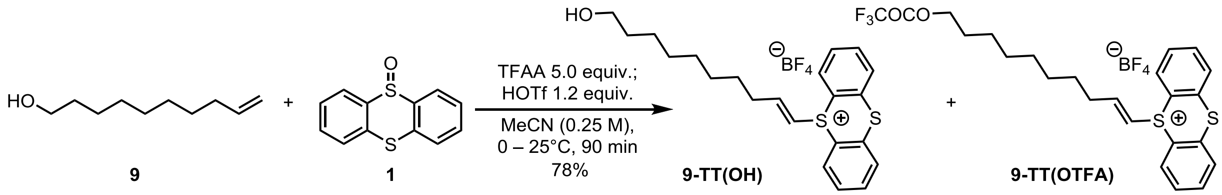

Under ambient atmosphere, a 20 mL borosilicate vial equipped with a magnetic stir bar was charged with 10-undecen-1-ol (78.1 mg, 0.500 mmol, 1.00 equiv.), thianthrene S-oxide (**1**) (120 mg, 0.517 mmol, 1.03 equiv.), and MeCN (2.0 mL, c = 0.25 M). After cooling to 0°C, trifluoroacetic anhydride (0.35 mL, 0.31 g, 1.5 mmol, 5.0 equiv.) was added dropwise within 30 seconds, followed by dropwise addition of HOTf (52 µL, 88 mg, 0.59 mmol, 1.2 equiv.) within 10 seconds. After stirring the lilac mixture at 0 °C for 60 min followed by stirring at 25 °C for 30 min, the resulting purple mixture was concentrated under reduced pressure and subsequently diluted with CH_2Cl_2 (10 mL). The CH_2Cl_2 solution was poured onto a saturated aqueous $NaHCO_3$ solution (ca. 20 mL). The combined mixture was poured into a separatory funnel, and the layers were separated. The CH_2Cl_2 layer was collected, and the aqueous layer was further extracted with CH_2Cl_2 (2 × ca. 10 mL). The combined CH_2Cl_2 solution was washed with aqueous $NaBF_4$ solution (2 × ca. 20 mL, 5 % w/w). The CH_2Cl_2 layer was dried over Na_2SO_4, filtered, and the solvent was removed under reduced pressure. The residue was purified by chromatography on silica gel eluting with CH_2Cl_2/*i*-PrOH (100:1, v/v). The product-containing fractions were collected and concentrated under reduced pressure. The residue was further dried in vacuo to afford **9-TT(OTFA)** (*E*/*Z* ≅ 14/1, 130.0 mg, 229 µmol, 46 %) as a colorless sticky oil and **9-TT(OH)** (*E*/*Z* ≅ 10/1, 75.4 mg, 160 µmol, 32 %) as a colorless sticky oil. **9-TT(OTFA)** was further purified by chromatography on silica gel eluting with CH_2Cl_2/*i*-PrOH (100:1, v/v), and fractions containing **9-TT(OTFA)(*E*)** were collected and concentrated under reduced pressure. The residue was further dried in vacuo.

With 2.40 equiv. HOTf:

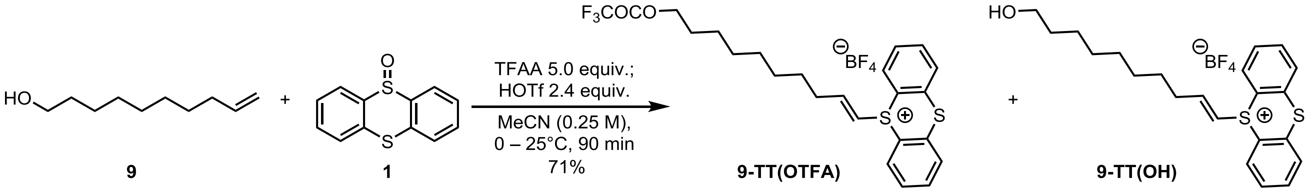

Under ambient atmosphere, a 20 mL borosilicate vial equipped with a magnetic stir bar was charged with 10-undecen-1-ol (78.1 mg, 0.500 mmol, 1.00 equiv.), thianthrene S-oxide (**1**) (120 mg, 0.517 mmol, 1.03 equiv.), and MeCN (2.0 mL, c = 0.25 M). After cooling to 0°C, trifluoroacetic anhydride (0.35 mL, 0.31 g, 1.5 mmol, 5.0 equiv.) was added dropwise within 30 seconds, followed by dropwise addition of HOTf (104 µL, 176 mg, 1.18 mmol, 2.40 equiv.) within 10 seconds. After stirring the lilac mixture at 0 °C for 60 min followed by stirring at 25 °C for 30 min, the resulting light purple mixture was concentrated under reduced pressure and subsequently diluted with CH_2Cl_2 (10 mL). The CH_2Cl_2 solution was cooled to 0 °C for 5 min and poured onto a saturated aqueous $NaHCO_3$ solution (ca. 20 mL) which was prechilled to 0 °C for 5 min. The combined mixture was stirred at 0 °C for 5 min and poured into a separatory funnel, and the layers were separated. The

CH_2Cl_2 layer was collected, and the aqueous layer was further extracted with CH_2Cl_2 (2 × ca. 10 mL). The combined CH_2Cl_2 solution was washed with aqueous $NaBF_4$ solution (2 × ca. 20 mL, 5 % w/w).The CH_2Cl_2 layer was dried over Na_2SO_4, filtered, and the solvent was removed under reduced pressure. The residue was purified by chromatography on silica gel eluting with CH_2Cl_2/*i*-PrOH (100:1 to 50:1, v/v). The product-containing fractions were collected and concentrated under reduced pressure. The residue was further dried in vacuo to afford **9-TT(OTFA)** (*E*/*Z* ≅ 31/1, 150.9 mg, 272 µmol, 54 %) as a light yellow sticky oil and **9-TT(OH)** (*E*/*Z* ≅ 16/1, 39.2 mg, 86 µmol, 17 %) as a light yellow sticky oil.

R*f* = 0.46 (CH_2Cl_2/MeOH, 15:1, v/v) for **9-TT(OTFA)**.

R*f* = 0.32 (CH_2Cl_2/MeOH, 15:1, v/v). for **9-TT(OH)**

NMR Spectroscopy for 9-TT(OTFA):

^{1}H NMR (500 MHz, CD_2Cl_2, 298 K, δ) 8.17 (d, *J* = 7.9 Hz, 2H), 7.80 (d, *J* = 7.9 Hz, 2H), 7.69 (tt, *J* = 7.8, 1.5 Hz, 2H), 7.65 – 7.60 (m, 2H), 7.08 – 6.99 (m, 1H), 6.47 (dt, *J* = 14.8, 1.6 Hz, 1H), 4.24 (t, *J* = 6.7 Hz, 2H), 2.23 – 2.13 (m, 2H), 1.69 – 1.56 (m, 2H), 1.33 (p, *J* = 7.1 Hz, 2H), 1.28 – 1.22 (m, 2H), 1.21 – 1.10 (m, 6H).

^{13}C NMR {^{1}H} (126 MHz, CD_2Cl_2, 298 K, δ): 158.6 (2C), 137.7, 136.0, 135.1, 132.3, 132.1, 122.2, 116.5 (q, J = 284.5 Hz), 111.3, 70.4, 35.2, 30.8, 30.7, 30.7, 29.9, 29.1, 27.1.

^{19}F NMR (471 MHz, CD_2Cl_2, 298 K, δ): –73.62 (s), –148.95 (bs), –149.01 (bs).

HRMS-ESI (m/z) 9-TT(OTFA) calc'd. for $C_{24}H_{26}F_3O_2S_2^+$ [M]$^+$, 467.13208; found, 467.13153; deviation: +1.19 ppm.

NMR Spectroscopy for 9-TT(OH):

^{1}H NMR (500 MHz, CD_2Cl_2, 298 K, δ) 10.19 (dd, *J* = 8.0, 1.4 Hz, 2H), 9.86 (dd, *J* = 7.9, 1.3 Hz, 2H), 9.75 (td, *J* = 7.7, 1.4 Hz, 2H), 9.65 (td, *J* = 7.7, 1.3 Hz, 2H), 9.06 (dt, *J* = 14.7, 6.9 Hz, 1H), 8.52 (dt, *J* = 14.8, 1.5 Hz, 1H), 6.28 (t, *J* = 6.7 Hz, 2H), 4.27 – 4.09 (m, 2H), 3.66 (dq, *J* = 8.4, 6.7 Hz, 2H), 3.36 (t, *J* = 7.5 Hz, 2H), 3.32 – 3.15 (m, 8H).

^{13}C NMR {^{1}H} (126 MHz, CD_2Cl_2, 298 K, δ): 157.3, 136.3, 135.1, 133.6, 130.8, 130.7, 120.7, 109.8, 63.1, 33.7, 33.2, 29.6, 29.5, 29.2, 27.7, 26.1.

^{19}F NMR (471 MHz, CD_2Cl_2, 298 K, δ): –150.67 (bs), –150.72 (bs).

HRMS-ESI (m/z) 9-TT(OH) calc'd. for $C_{22}H_{27}OS_2^+$ [M]$^+$, 371.14978; found, 371.14910; deviation: +1.85 ppm.

4-Phenyl-1-butene-derived thianthrenium salt 10-TT

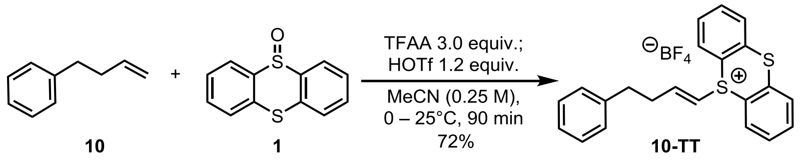

Under ambient atmosphere, a 20 mL borosilicate vial equipped with a magnetic stir bar was charged with 4-phenyl-1-butene (75.1 µL, 66.1 mg, 0.500 mmol, 1.00 equiv.), thianthrene S-oxide (**1**) (120 mg, 0.517 mmol, 1.03 equiv.), and MeCN (2.0 mL, c = 0.25 M). After cooling to 0°C, trifluoroacetic anhydride (0.21 mL, 0.31 g, 1.5 mmol, 3.0 equiv.) was added dropwise within 30 seconds, followed by dropwise addition of HOTf (52 µL, 88 mg, 0.59 mmol, 1.2 equiv.) within 10 seconds. After stirring the lilac mixture at 0 °C for 60 min followed by stirring at 25 °C for 30 min, the resulting purple mixture was concentrated under reduced pressure and subsequently diluted with CH_2Cl_2 (10 mL). The CH_2Cl_2 solution was poured onto a saturated aqueous $NaHCO_3$ solution (ca. 20 mL). The combined mixture was poured into a separatory funnel, and the layers were separated. The CH_2Cl_2 layer was collected, and the aqueous layer was further extracted with CH_2Cl_2 (2 × ca. 10 mL). The combined CH_2Cl_2 solution was washed with aqueous $NaBF_4$ solution (2 × ca. 20 mL, 5 % w/w). The CH_2Cl_2 layer was dried over Na_2SO_4, filtered, and the solvent was removed under reduced pressure. The residue was purified by chromatography on silica gel eluting with CH_2Cl_2/*i*-PrOH (100:1, v/v). The product-containing fractions were collected and concentrated under reduced pressure. The residue was further dried in vacuo to afford **10-TT** (*E*/*Z* ≅ 22/1, 156.0 mg, 359 µmol, 72 %) as a colorless solid.

R*f* = 0.46 (CH_2Cl_2/MeOH, 15:1, v/v).

NMR Spectroscopy:

^{1}H NMR (500 MHz, CD_2Cl_2, 298 K, δ) 8.16 (d, *J* = 7.9 Hz, 2H), 7.85 (d, *J* = 7.8 Hz, 2H), 7.76 (td, *J* = 7.7, 1.4 Hz, 2H), 7.69 (td, *J* = 7.7, 1.4 Hz, 2H), 7.17 (dd, *J* = 8.0, 6.4 Hz, 2H), 7.14 – 7.06 (m, 2H), 7.05 – 7.02 (m, 2H), 6.41 (d, *J* = 14.8 Hz, 1H), 2.76 (t, *J* = 7.3 Hz, 2H), 2.61 (q, *J* = 7.2 Hz, 2H).

^{13}C NMR {^{1}H} (126 MHz, $CDCl_3$, 298 K, δ): 156.3, 139.8, 135.7, 134.6, 133.9, 130.6, 130.4, 128.84, 128.79, 126.7, 121.1, 110.5, 35.2, 33.6.

^{19}F NMR (471 MHz, $CDCl_3$, 298 K, δ): –150.60(bs), –150.65 (bs).

HRMS-ESI (m/z) calc'd. for $C_{22}H_{19}S_2^+$ $[M]^+$, 347.09227; found, 347.09167; deviation: +1.73 ppm.

Pent-4-en-1-yl-phthalimide-derived thianthrenium salt 11-TT

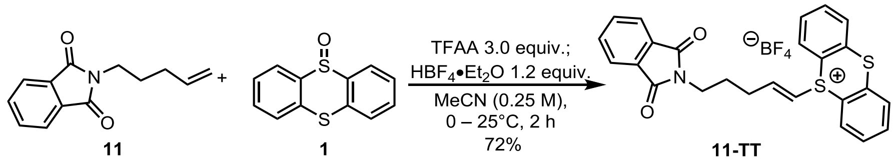

Under ambient atmosphere, a 20 mL borosilicate vial equipped with a magnetic stir bar was charged with pent-4-en-1-yl-phthalimide[1] (645 mg, 3.00 mmol, 1.00 equiv.), thianthrene S-oxide (**1**) (696 mg, 3.00 mmol, 1.00 equiv.), and MeCN (12 mL, c = 0.25 M). After cooling to 0°C, trifluoroacetic anhydride (1.26 mL, 1.86 g, 9.00 mmol, 3.00 equiv.) was added dropwise within 30 seconds, followed by dropwise addition of HBF_4•OEt_2 (522 µL, 3.60 mmol, 1.2 equiv.) within 10 seconds. After stirring the lilac mixture at 0 °C for 60 min followed by stirring at 25 °C for 60 min, the resulting purple mixture was concentrated under reduced pressure and

subsequently diluted with CH_2Cl_2 (10 mL). The CH_2Cl_2 solution was poured onto a saturated aqueous $NaHCO_3$ solution (ca. 20 mL). The combined mixture was poured into a separatory funnel, and the layers were separated. The CH_2Cl_2 layer was collected, and the aqueous layer was further extracted with CH_2Cl_2 (2 × ca. 10 mL). The combined CH_2Cl_2 solution was washed with aqueous $NaBF_4$ solution (2 × ca. 20 mL, 5 % w/w). The CH_2Cl_2 layer was dried over Na_2SO_4, filtered, and the solvent was removed under reduced pressure. The residue was purified by chromatography on silica gel eluting with CH_2Cl_2/*i*-PrOH (100:1, v/v). The product-containing fractions were collected and concentrated under reduced pressure. The residue was further dried in vacuo to afford **11-TT** (*E*/*Z* > 50/1, 1.12 g, 2.17 mmol, 72 %) as a colorless solid.

R*f* = 0.40 (CH_2Cl_2/MeOH, 15:1, v/v).

NMR Spectroscopy:

^{1}H NMR (500 MHz, CD_2Cl_2, 298 K, δ 8.24 (dd, *J* = 7.9, 1.3 Hz, 2H), 7.89 (dd, *J* = 7.9, 1.3 Hz, 2H), 7.82 – 7.74 (m, 4H), 7.76 – 7.67 (m, 4H), 7.06 (ddd, *J* = 14.0, 7.3, 6.3 Hz, 1H), 6.61 (dd, *J* = 14.8, 1.4 Hz, 1H), 3.60 (t, *J* = 6.8 Hz, 2H), 2.33 (q, *J* = 8.1, 7.5 Hz, 2H), 1.80 (p, *J* = 7.2 Hz, 2H).

^{13}C NMR {^{1}H} (126 MHz, CD_2Cl_2, 298 K, δ): 168.7, 155.2, 136.5, 135.1, 134.6, 133.9, 132.5, 130.9, 130.8, 123.6, 120.6, 110.9, 37.2, 30.9, 26.8.

^{19}F NMR (471 MHz, CD_2Cl_2, 298 K, δ): –151.07(bs), –151.12 (bs).

HRMS-ESI (m/z) calc'd. for $C_{25}H_{20}NO_2S_2^+$ $[M]^+$, 430.09299 found, 430.09273; deviation: +0.63 ppm.

Allylbenzol-derived thianthrenium salt 12-TT

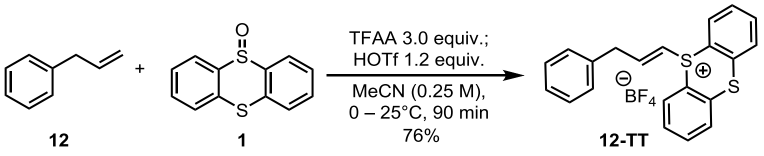

Under ambient atmosphere, a 20 mL borosilicate vial equipped with a magnetic stir bar was charged with allylbenzol (66.4 µL, 59.1 mg, 0.500 mmol, 1.00 equiv.), thianthrene S-oxide (**1**) (120 mg, 0.517 mmol, 1.03 equiv.), and MeCN (2.0 mL, c = 0.25 M). After cooling to 0°C, trifluoroacetic anhydride (0.21 mL, 0.31 g, 1.5 mmol, 3.0 equiv.) was added dropwise within 30 seconds, followed by dropwise addition of HOTf (52 µL, 88 mg, 0.59 mmol, 1.2 equiv.) within 10 seconds. After stirring the lilac mixture at 0 °C for 60 min followed by stirring at 25 °C for 30 min, the resulting purple mixture was concentrated under reduced pressure and subsequently diluted with CH_2Cl_2 (10 mL). The CH_2Cl_2 solution was poured onto a saturated aqueous $NaHCO_3$ solution (ca. 20 mL). The combined mixture was poured into a separatory funnel, and the layers were separated. The CH_2Cl_2 layer was collected, and the aqueous layer was further extracted with CH_2Cl_2 (2 × ca. 10 mL). The combined CH_2Cl_2 solution was washed with aqueous $NaBF_4$ solution (2 × ca. 20 mL, 5 % w/w). The CH_2Cl_2 layer was dried over Na_2SO_4, filtered, and the solvent was removed under reduced pressure. The residue was purified by chromatography on silica gel eluting with CH_2Cl_2/*i*-PrOH (100:1, v/v). The product-containing fractions were collected and concentrated under reduced pressure. The residue was further dried in vacuo to afford **12-TT** (*E*/*Z* > 50/1, 158.9 mg, 378 µmol, 76 %) as a colorless solid.

R*f* = 0.46 (CH_2Cl_2/MeOH, 15:1, v/v).

NMR Spectroscopy:

> **^{1}H NMR** (500 MHz, CD_2Cl_2, 298 K, δ) 8.22 (dd, *J* = 7.9, 1.4 Hz, 2H), 7.88 (dt, *J* = 8.0, 1.4 Hz, 2H), 7.77 (tt, *J* = 7.8, 1.4 Hz, 2H), 7.69 (td, *J* = 7.7, 1.3 Hz, 2H), 7.31 – 7.26 (m, 2H), 7.26 – 7.21 (m, 1H), 7.18 (dt, *J* = 14.8, 6.7 Hz, 1H), 7.11 – 7.08 (m, 2H), 6.50 (dt, *J* = 14.8, 1.6 Hz, 1H), 3.60 (dd, *J* = 6.7, 1.6 Hz, 2H).
>
> **^{13}C NMR {^{1}H}** (126 MHz, CD_2Cl_2, 298 K, δ): 154.8, 136.5, 135.8, 135.1, 133.9, 130.9, 130.8, 129.5, 129.3, 127.8, 120.5, 111.2, 39.6.
>
> **^{19}F NMR** (471 MHz, CD_2Cl_2, 298 K, δ): –150.87 (bs), –150.92 (bs).

HRMS-ESI (m/z) calc'd. for $C_{21}H_{17}S_2^+$ $[M]^+$, 333.07662; found, 333.07610; deviation: +1.56 ppm.

1-Allyl-4-(trifluormethyl)-benzol-derived thianthrenium salt 13-TT

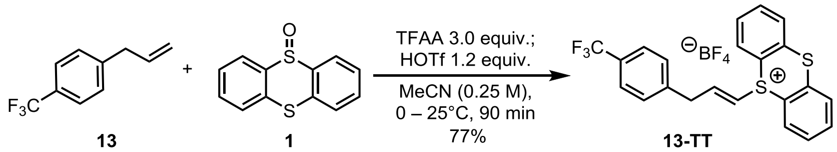

Under ambient atmosphere, a 20 mL borosilicate vial equipped with a magnetic stir bar was charged with 1-allyl-4-(trifluormethyl)-benzol (83.9 µL, 93.1 mg, 0.500 mmol, 1.00 equiv.), thianthrene S-oxide (**1**) (120 mg, 0.517 mmol, 1.03 equiv.), and MeCN (2.0 mL, c = 0.25 M). After cooling to 0°C, trifluoroacetic anhydride (0.21 mL, 0.31 g, 1.5 mmol, 3.0 equiv.) was added dropwise within 30 seconds, followed by dropwise addition of HOTf (52 µL, 88 mg, 0.59 mmol, 1.2 equiv.) within 10 seconds. After stirring the lilac mixture at 0 °C for 60 min followed by stirring at 25 °C for 30 min, the resulting purple mixture was concentrated under reduced pressure and subsequently diluted with CH_2Cl_2 (10 mL). The CH_2Cl_2 solution was poured onto a saturated aqueous $NaHCO_3$ solution (ca. 20 mL). The combined mixture was poured into a separatory funnel, and the layers were separated. The CH_2Cl_2 layer was collected, and the aqueous layer was further extracted with CH_2Cl_2 (2 × ca. 10 mL). The combined CH_2Cl_2 solution was washed with aqueous $NaBF_4$ solution (2 × ca. 20 mL, 5 % w/w). The CH_2Cl_2 layer was dried over Na_2SO_4, filtered, and the solvent was removed under reduced pressure. The residue was purified by chromatography on silica gel eluting with CH_2Cl_2/*i*-PrOH (100:1, v/v). The product-containing fractions were collected and concentrated under reduced pressure. The residue was further dried in vacuo to afford **13-TT** (*E*/*Z* > 50/1, 187.0 mg, 383 µmol, 77 %) as a colorless solid.

R*f* = 0.46 (CH_2Cl_2/MeOH, 15:1, v/v).

NMR Spectroscopy:

> **^{1}H NMR** (500 MHz, CD_2Cl_2, 298 K, δ) 8.21 (dd, *J* = 8.0, 1.4 Hz, 2H), 7.88 (dd, *J* = 8.0, 1.3 Hz, 2H), 7.77 (td, *J* = 7.7, 1.3 Hz, 2H), 7.67 (td, *J* = 7.7, 1.4 Hz, 2H), 7.52 (d, *J* = 8.0 Hz, 2H), 7.28 (d, *J* = 8.0 Hz, 2H), 7.15 (dt, *J* = 14.8, 6.9 Hz, 1H), 6.56 (dt, *J* = 14.8, 1.5 Hz, 1H), 3.67 (dd, *J* = 6.8, 1.5

Hz, 2H).

^{13}C NMR {^{1}H} (126 MHz, CD_2Cl_2, 298 K, δ): 153.1, 140.3, 136.4, 135.2, 133.7, 130.9, 130.6, 129.8, 129.5, 126.2 (q, J = 3.8 Hz), 124.6 (q, J = 271.8 Hz), 120.1, 112.1, 39.0.

^{19}F NMR (471 MHz, CD_2Cl_2, 298 K, δ): –62.73, –150.27 (bs), –150.37 (bs).

HRMS-ESI (m/z) calc'd. for $C_{12}H_{16}F_3S_2^+$ $[M]^+$, 401.06400; found, 401.06332; deviation: +1.71 ppm.

Cyclododecene-derived thianthrenium salt 14-TT

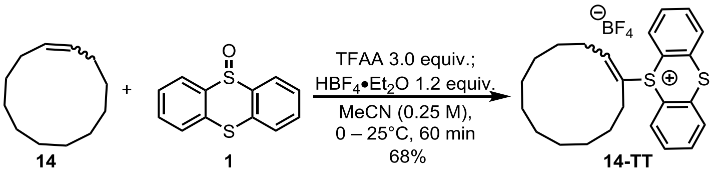

Under ambient atmosphere, a 20 mL borosilicate vial equipped with a magnetic stir bar was charged with cyclododecene (E/Z ≅ 1/2, 96.7 µL, 83.2 mg, 0.500 mmol, 1.00 equiv.), thianthrene S-oxide (**1**) (120 mg, 0.517 mmol, 1.03 equiv.), and MeCN (2.0 mL, c = 0.25 M). After cooling to 0°C, trifluoroacetic anhydride (0.21 mL, 0.31 g, 1.5 mmol, 3.0 equiv.) was added dropwise within 30 seconds, followed by dropwise addition of $HBF_4{\cdot}OEt_2$ (87 µL, 0.60 mmol, 1.2 equiv.) within 10 seconds. After stirring the lilac mixture at 0 °C for 60 min, the resulting pink mixture was concentrated under reduced pressure and subsequently diluted with CH_2Cl_2 (10 mL). The CH_2Cl_2 solution was poured onto a saturated aqueous $NaHCO_3$ solution (ca. 20 mL). The combined mixture was poured into a separatory funnel, and the layers were separated. The CH_2Cl_2 layer was collected, and the aqueous layer was further extracted with CH_2Cl_2 (2 × ca. 10 mL). The combined CH_2Cl_2 solution was washed with aqueous $NaBF_4$ solution (2 × ca. 20 mL, 5 % w/w). The CH_2Cl_2 layer was dried over Na_2SO_4, filtered, and the solvent was removed under reduced pressure. The residue was purified by chromatography on silica gel eluting with CH_2Cl_2/*i*-PrOH (100:1, v/v). The product-containing fractions were collected and concentrated under reduced pressure. The residue was further dried in vacuo to afford **14-TT** (*E*/*Z* ≅ 1/1, 159.0 mg, 339 µmol, 68 %) as a colorless solid.

R*f* = 0.46 (CH_2Cl_2/MeOH, 15:1, v/v).

NMR Spectroscopy:

^{1}H NMR (500 MHz, CD_2Cl_2, 298 K, δ) 8.22 (dd, J = 7.9, 1.4 Hz, 1H), 8.11 (dd, J = 8.1, 1.4 Hz, 1H), 7.92 (dd, J = 7.9, 1.4 Hz, 1H), 7.84 (td, J = 7.8, 1.4 Hz, 2H), 7.80 (td, J = 7.7, 1.4 Hz, 1H), 7.76 (td, J = 7.6, 1.4 Hz,1H), 7.74 – 7.67 (m, 1H), 6.49 (t, J = 7.7 Hz, 0.4H), 5.39 (t, J = 8.2 Hz, 0.5H), 2.67 – 2.59 (m, 1H), 2.39 – 2.32 (m, 1H), 2.22 (t, J = 7.2 Hz, 1H), 2.20 – 2.16 (m, 1H), 1.77 – 1.70 (m, 1H), 1.58 – 1.49 (m, 2H), 1.43 – 1.27 (m, 8H), 1.28 – 1.21 (m, 2H), 1.21 – 1.13 (m, 2H), 1.13 – 1.06 (m, 1H).

^{13}C NMR {^{1}H} (126 MHz, CD_2Cl_2, 298 K, δ): 150.4, 142.3, 137.8, 137.6, 135.5, 135.5, 135.3, 134.5, 131.2, 131.0, 130.8, 130.7, 130.6, 124.4, 117.9, 117.1, 34.8, 30.4, 27.4, 26.4, 26.1, 26.0, 25.9, 25.69, 25.67, 25.2, 25.1, 24.9, 24.8, 24.7, 24.23, 24.16, 22.9, 22.4.

^{19}F NMR (471 MHz, CD_2Cl_2, 298 K, δ): –152.16 (bs), –152.21 (bs).

HRMS-ESI (m/z) calc'd. for $C_{24}H_{29}S_2^+$ $[M]^+$, 381.17052; found, 381.17032; deviation: +0.53 ppm.

(+)-Rosenoxide-derived thianthrenium salt 15-TT

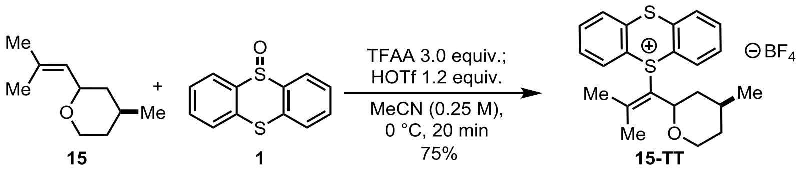

Under ambient atmosphere, a 20 mL borosilicate vial equipped with a magnetic stir bar was charged with (+)-rosenoxide (88.6 µL, 77.1 mg, 0.500 mmol, 1.00 equiv.), thianthrene S-oxide (**1**) (120 mg, 0.517 mmol, 1.03 equiv.), and MeCN (2.0 mL, c = 0.25 M). After cooling to 0°C, trifluoroacetic anhydride (0.21 mL, 0.31 g, 1.5 mmol, 3.0 equiv.) was added dropwise within 30 seconds, followed by dropwise addition of HOTf (52 µL, 88 mg, 0.59 mmol, 1.2 equiv.) within 10 seconds. After stirring the lilac mixture at 0 °C for 20 min, the resulting yellow mixture was concentrated under reduced pressure and subsequently diluted with CH_2Cl_2 (10 mL). The CH_2Cl_2 solution was poured onto a saturated aqueous $NaHCO_3$ solution (ca. 20 mL). The combined mixture was poured into a separatory funnel, and the layers were separated. The CH_2Cl_2 layer was collected, and the aqueous layer was further extracted with CH_2Cl_2 (2 × ca. 10 mL). The combined CH_2Cl_2 solution was washed with aqueous $NaBF_4$ solution (2 × ca. 20 mL, 5 % w/w). The CH_2Cl_2 layer was dried over Na_2SO_4, filtered, and the solvent was removed under reduced pressure. The residue was purified by chromatography on silica gel eluting with CH_2Cl_2/*i*-PrOH (100:1, v/v). The product-containing fractions were collected and concentrated under reduced pressure. The residue was further dried in vacuo to afford **15-TT** (170.0 mg, 373 µmol, 75 %) as a colorless solid.

R*f* = 0.46 (CH_2Cl_2/MeOH, 15:1, v/v).

NMR Spectroscopy:

^{1}H NMR (500 MHz, $CDCl_3$, 298 K, δ) 8.00 – 7.95 (m, 1H), 7.87 – 7.82 (m, 1H), 7.82 – 7.79 (m, 1H), 7.76 – 7.71 (m, 2H), 7.69 – 7.64 (m, 3H), 4.85 (dd, *J* = 11.6, 2.4 Hz, 1H), 3.99 (ddd, *J* = 11.6, 4.6, 1.5 Hz, 1H), 3.56 (ddd, *J* = 12.5, 11.5, 2.3 Hz, 1H), 2.45 (s, 3H), 2.10 (d, *J* = 13.0 Hz, 1H), 1.82 – 1.77 (m, 1H), 1.76 (s, 3H), 1.58 – 1.52 (m, 1H), 1.18 – 1.08 (m, 1H), 1.00 (dt, *J* = 12.9, 11.6 Hz, 1H), 0.93 (d, *J* = 6.5 Hz, 3H).

^{13}C NMR {^{1}H} (126 MHz, CD_2Cl_2, 298 K, δ): 133.1 (d, *J* = 5.7 Hz), 131.3(d, *J* = 6.7 Hz), 130.2 (d, *J* = 11.5 Hz), 129.6 (d, *J* = 17.2 Hz), 128.9, 128.6, 123.7, 118.9, 75.1, 68.5, 38.8, 33.6, 29.7, 26.7, 24.5, 21.9.

^{19}F NMR (471 MHz, CD_2Cl_2, 298 K, δ): –152.96 (bs), –153.02 (bs).

HRMS-ESI (m/z) calc'd. for $C_{22}H_{25}OS_2^+$ $[M]^+$, 369.13413; found, 369.13367; deviation: +1.26 ppm.

3,4-Dihydro-2*H*-pyrane-derived thianthrenium salt 16-TT

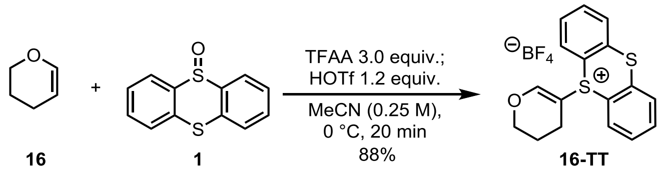

Under ambient atmosphere, a 20 mL borosilicate vial equipped with a magnetic stir bar was charged with 3,4-dihydro-2*H*-pyrane (45.7 µL, 42.1 mg, 0.500 mmol, 1.00 equiv.), thianthrene S-oxide (**1**) (120 mg, 0.517 mmol, 1.03 equiv.), and MeCN (2.0 mL, c = 0.25 M). After cooling to 0°C, trifluoroacetic anhydride (0.21 mL, 0.31 g, 1.5 mmol, 3.0 equiv.) was added dropwise within 30 seconds, followed by dropwise addition of HOTf (52 µL, 88 mg, 0.59 mmol, 1.2 equiv.) within 10 seconds. After stirring the lilac mixture at 0 °C for 20 min, the resulting yellow mixture was concentrated under reduced pressure and subsequently diluted with CH_2Cl_2 (10 mL). The CH_2Cl_2 solution was poured onto a saturated aqueous $NaHCO_3$ solution (ca. 20 mL). The combined mixture was poured into a separatory funnel, and the layers were separated. The CH_2Cl_2 layer was collected, and the aqueous layer was further extracted with CH_2Cl_2 (2 × ca. 10 mL). The combined CH_2Cl_2 solution was washed with aqueous $NaBF_4$ solution (2 × ca. 20 mL, 5 % w/w). The CH_2Cl_2 layer was dried over Na_2SO_4, filtered, and the solvent was removed under reduced pressure. The residue was purified by chromatography on silica gel eluting with CH_2Cl_2/*i*-PrOH (100:1, v/v). The product-containing fractions were collected and concentrated under reduced pressure. The residue was further dried in vacuo to afford **10-TT** (169.0 mg, 438 µmol, 88 %) as a colorless solid.

R*f* = 0.46 (CH_2Cl_2/MeOH, 15:1, v/v).

NMR Spectroscopy:

^{1}H NMR (500 MHz, CD_2Cl_2, 298 K, δ) 8.07 (dd, *J* = 7.9, 1.2 Hz, 2H), 7.79 – 7.73 (m, 5H), 7.69 (ddd, *J* = 8.6, 6.6, 2.0 Hz, 2H), 4.18 – 4.14 (t, *J* = 5.9 Hz, 2H), 2.21 (t, *J* = 6.3 Hz, 2H), 1.95 (p, *J* = 6.1 Hz, 2H).

^{13}C NMR {^{1}H} (126 MHz, CD_2Cl_2, 298 K, δ): 159.6, 136.2, 134.6, 133.0, 130.6, 130.0, 118.6, 103.0, 68.3, 21.8, 20.2.

^{19}F NMR (471 MHz, CD_2Cl_2, 298 K, δ): –151.57 (bs), –151.59 (bs).

HRMS-ESI (m/z) calc'd. for $C_{22}H_{25}OS_2^+$ $[M]^+$, 369.13413; found, 369.13432; deviation: –0.50 ppm.

N-(But-3-en-1-yl)benzamide-derived thianthrenium salt 17-TT

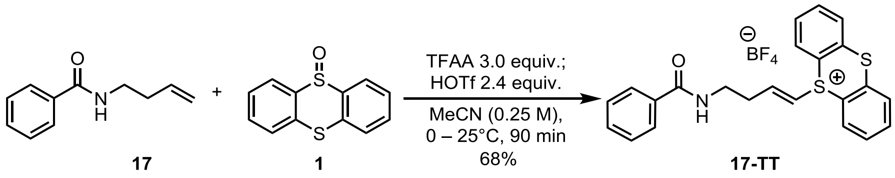

Under ambient atmosphere, a 20 mL borosilicate vial equipped with a magnetic stir bar was charged with *N*-(but-3-en-1-yl)benzamide[2] (109 mg, 0.500 mmol, 1.00 equiv.), thianthrene S-oxide (**1**) (120 mg, 0.517 mmol,

1.03 equiv.), and MeCN (2.0 mL, c = 0.25 M). After cooling to 0°C, HOTf (52 µL, 88 mg, 0.59 mmol, 1.2 equiv.) within 10 seconds, followed by dropwise addition of trifluoroacetic anhydride (0.21 mL, 0.31 g, 1.5 mmol, 3.0 equiv.) was added dropwise within 30 seconds. After stirring the lilac mixture at 0 °C for 60 min followed by stirring at 25 °C for 30 min, the resulting purple mixture was concentrated under reduced pressure and subsequently diluted with CH_2Cl_2 (10 mL). The CH_2Cl_2 solution was poured onto a saturated aqueous $NaHCO_3$ solution (ca. 20 mL). The combined mixture was poured into a separatory funnel, and the layers were separated. The CH_2Cl_2 layer was collected, and the aqueous layer was further extracted with CH_2Cl_2 (2 × ca. 10 mL). The combined CH_2Cl_2 solution was washed with aqueous $NaBF_4$ solution (2 × ca. 20 mL, 5 % w/w). The CH_2Cl_2 layer was dried over Na_2SO_4, filtered, and the solvent was removed under reduced pressure. The residue was purified by chromatography on silica gel eluting with CH_2Cl_2/*i*-PrOH (70:1 to 30:1, v/v). The product-containing fractions were collected and concentrated under reduced pressure. The residue was further dried in vacuo to afford **10-TT** (*E*/*Z* > 50/1, 163.0 mg, 342 µmol, 68 %) as a beige solid.

R*f* = 0.21 (CH_2Cl_2/MeOH, 15:1, v/v).

NMR Spectroscopy:

^{1}H NMR (500 MHz, CD_2Cl_2, 298 K, δ) 8.09 (dd, *J* = 8.0, 1.4 Hz, 2H), 7.76 (dd, *J* = 7.9, 1.4 Hz, 2H), 7.66 (t, *J* = 7.8 Hz, 4H), 7.56 (td, *J* = 7.7, 1.4 Hz, 2H), 7.44 (td, *J* = 7.4, 1.4 Hz, 1H), 7.35 – 7.31 (m, 2H), 7.31 – 7.26 (m, 1H), 7.26 – 7.20 (m, 1H), 6.64 (dd, *J* = 14.8, 1.4 Hz, 1H), 3.55 (q, *J* = 6.1 Hz, 2H), 2.61 (q, *J* = 6.5 Hz, 2H).

^{13}C NMR {^{1}H} (126 MHz, CD_2Cl_2, 298 K, δ): 167.7, 155.9, 136.0, 134.9, 134.5, 133.3, 131.8, 130.7, 130.5, 128.9, 127.5, 120.8, 111.2, 37.9, 34.2.

^{19}F NMR (471 MHz, $CDCl_3$, 298 K, δ): –149.98 (bs), –150.03 (bs).

HRMS-ESI (m/z) calc'd. for $C_{23}H_{20}NOS_2^+$ $[M]^+$, 390.09808; found, 390.09744; deviation: +1.65 ppm.

Cyclooctene-derived thianthrenium salt 18-TT

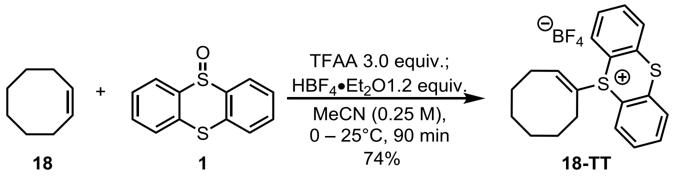

Under ambient atmosphere, a 20 mL borosilicate vial equipped with a magnetic stir bar was charged with cyclooctene (389 µL, 331 mg, 3.00 mmol, 1.00 equiv.), thianthrene S-oxide (**1**) (696 mg, 3.00 mmol, 1.00 equiv.), and MeCN (12 mL, c = 0.25 M). After cooling to 0°C, trifluoroacetic anhydride (1.26 mL, 1.86 g, 9.00 mmol, 3.00 equiv.) was added dropwise within 30 seconds, followed by dropwise addition of HBF_4•OEt_2 (522 µL, 4.20 mmol, 1.20 equiv.) within 10 seconds. After stirring the lilac mixture at 0 °C for 60 min followed by stirring at 25 °C for 30 min, the resulting purple mixture was concentrated under reduced pressure and subsequently diluted with CH_2Cl_2 (20 mL). The CH_2Cl_2 solution was poured onto a saturated aqueous $NaHCO_3$ solution (ca. 20 mL). The combined mixture was poured into a separatory funnel, and the layers

were separated. The CH_2Cl_2 layer was collected, and the aqueous layer was further extracted with CH_2Cl_2 (2 × ca. 10 mL). The combined CH_2Cl_2 solution was washed with aqueous $NaBF_4$ solution (2 × ca. 20 mL, 5 % w/w). The CH_2Cl_2 layer was dried over Na_2SO_4, filtered, and the solvent was removed under reduced pressure. The residue was purified by chromatography on silica gel eluting with CH_2Cl_2/*i*-PrOH (100:1, v/v). The product-containing fractions were collected and concentrated under reduced pressure. The residue was further dried in vacuo to afford **18-TT** (920 mg, 2.23 mmol, 74 %) as a colorless solid.

R*f* = 0.46 (CH_2Cl_2/MeOH, 15:1, v/v).

NMR Spectroscopy:

^{1}H NMR (500 MHz, CD_2Cl_2, 298 K, δ) 8.22 (dd, *J* = 7.9, 1.5 Hz, 2H), 7.89 (dd, *J* = 8.1, 1.6 Hz, 2H), 7.84 (td, *J* = 7.6, 1.5 Hz, 2H), 7.75 (td, *J* = 7.7, 1.6 Hz, 2H), 5.76 (dd, *J* = 9.1, 7.8 Hz, 1H), 2.50 (t, *J* = 6.3 Hz, 2H), 2.29 – 2.22 (m, 2H), 1.57 (p, *J* = 6.1 Hz, 2H), 1.43 – 1.33 (m, 4H), 1.03 (p, *J* = 5.9 Hz, 2H).

^{13}C NMR {^{1}H} (126 MHz, CD_2Cl_2, 298 K, δ): 143.3, 137.4, 135.6 (d, *J* = 1.7 Hz), 135.3, 130.72, 130.67, 123.8, 116.8 (d, *J* = 2.4 Hz), 29.5, 28.6, 28.4, 28.3, 26.3, 25.6.

^{19}F NMR (471 MHz, CD_2Cl_2, 298 K, δ): –152.30 (bs), –152.34 (bs).

HRMS-ESI (m/z) calc'd. for $C_{20}H_{21}S_2^+$ $[M]^+$, 325.10792; found, 325.10728; deviation: +1.97 ppm.

Cycloheptene-derived thianthrenium salt 19-TT

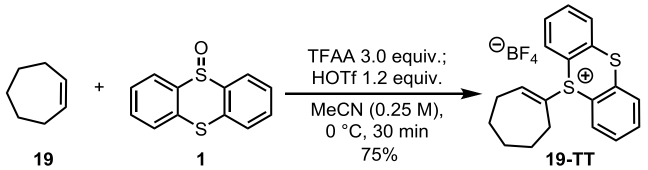

Under ambient atmosphere, a 20 mL borosilicate vial equipped with a magnetic stir bar was charged with cycloheptene (58.6 µL,486.1 mg, 0.500 mmol, 1.00 equiv.), thianthrene S-oxide (**1**) (120 mg, 0.517 mmol, 1.03 equiv.), and MeCN (2.0 mL, c = 0.25 M). After cooling to 0°C, trifluoroacetic anhydride (0.21 mL, 0.31 g, 1.5 mmol, 3.0 equiv.) was added dropwise within 30 seconds, followed by dropwise addition of HOTf (52 µL, 88 mg, 0.59 mmol, 1.2 equiv.) within 10 seconds. After stirring the lilac mixture at 0 °C for 30 min, the resulting pink mixture was concentrated under reduced pressure and subsequently diluted with CH_2Cl_2 (10 mL). The CH_2Cl_2 solution was poured onto a saturated aqueous $NaHCO_3$ solution (ca. 20 mL). The combined mixture was poured into a separatory funnel, and the layers were separated. The CH_2Cl_2 layer was collected, and the aqueous layer was further extracted with CH_2Cl_2 (2 × ca. 10 mL). The combined CH_2Cl_2 solution was washed with aqueous $NaBF_4$ solution (2 × ca. 20 mL, 5 % w/w). The CH_2Cl_2 layer was dried over Na_2SO_4, filtered, and the solvent was removed under reduced pressure. The residue was purified by chromatography on silica gel eluting with CH_2Cl_2/*i*-PrOH (100:1, v/v). The product-containing fractions were collected and concentrated under reduced pressure. The residue was further dried in vacuo to afford **19-TT** (149.3 mg, 375 µmol, 75 %) as a colorless solid.

R*f* = 0.46 (CH_2Cl_2/MeOH, 15:1, v/v).

NMR Spectroscopy:

^{1}H NMR (500 MHz, CD_2Cl_2, 298 K, δ) 8.20 (d, *J* = 8.0 Hz, 2H), 7.89 (d, *J* = 7.9 Hz, 2H), 7.83 (t, *J* = 7.7 Hz, 2H), 7.74 (t, *J* = 7.6 Hz, 2H), 5.92 (t, *J* = 6.6 Hz, 1H), 2.28 (dq, *J* = 11.4, 5.9 Hz, 4H), 1.67 (dp, *J* = 11.6, 5.1, 4.7 Hz, 2H), 1.49 (p, *J* = 5.8 Hz, 2H), 1.34 (p, *J* = 5.8 Hz, 2H).

^{13}C NMR {^{1}H} (126 MHz, CD_2Cl_2, 298 K, δ): 145.1, 137.1 (d, *J* = 2.1 Hz), 135.4 (d, *J* = 3.7 Hz), 135.3, 130.8 (d, *J* = 2.0 Hz), 130.6, 124.6, 117.6 (d, *J* = 2.2 Hz), 31.0, 30.6, 29.9, 26.1, 25.4.

^{19}F NMR (471 MHz, CD_2Cl_2, 298 K, δ): –151.78 (bs), –151.83 (bs).

HRMS-ESI (m/z) calc'd. for $C_{19}H_{19}S_2^+$ $[M]^+$, 311.09227; found, 311.09166; deviation: +1.96 ppm.

Cyclohexene-derived thianthrenium salt 20-TT

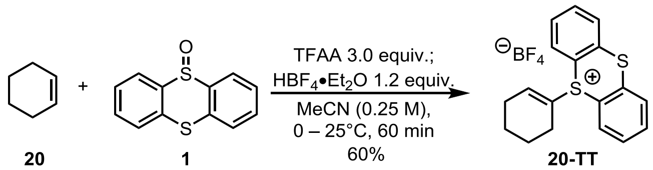

Under ambient atmosphere, a 20 mL borosilicate vial equipped with a magnetic stir bar was charged with cyclohexene (50.6 µL, 41.1 mg, 0.500 mmol, 1.00 equiv.), thianthrene S-oxide (**1**) (120 mg, 0.517 mmol, 1.03 equiv.), and MeCN (2.0 mL, c = 0.25 M). After cooling to 0°C, trifluoroacetic anhydride (0.21 mL, 0.31 g, 1.5 mmol, 3.0 equiv.) was added dropwise within 30 seconds, followed by dropwise addition of HBF_4•OEt_2 (87 µL, 0.59 mmol, 1.2 equiv.) within 10 seconds. After stirring the lilac mixture at 0 °C for 45 min followed by stirring at 25 °C for 15 min, the resulting purple mixture was concentrated under reduced pressure and subsequently diluted with CH_2Cl_2 (10 mL). The CH_2Cl_2 solution was poured onto a saturated aqueous $NaHCO_3$ solution (ca. 20 mL). The combined mixture was poured into a separatory funnel, and the layers were separated. The CH_2Cl_2 layer was collected, and the aqueous layer was further extracted with CH_2Cl_2 (2 × ca. 10 mL). The combined CH_2Cl_2 solution was washed with aqueous $NaBF_4$ solution (2 × ca. 20 mL, 5 % w/w). The CH_2Cl_2 layer was dried over Na_2SO_4, filtered, and the solvent was removed under reduced pressure. The residue was purified by chromatography on silica gel eluting with CH_2Cl_2/*i*-PrOH (100:1, v/v). The product-containing fractions were collected and concentrated under reduced pressure. The residue was further dried in vacuo to afford **20-TT** (115.5 mg, 301 µmol, 60 %) as a colorless solid.

R*f* = 0.46 (CH_2Cl_2/MeOH, 15:1, v/v).

NMR Spectroscopy:

^{1}H NMR (500 MHz, CD_2Cl_2, 298 K, δ) 8.19 (d, *J* = 7.9 Hz, 2H), 7.88 (d, *J* = 7.9 Hz, 2H), 7.82 (t, *J* = 7.7 Hz, 2H), 7.75 – 7.70 (m, 2H), 6.05 (d, *J* = 4.1 Hz, 1H), 2.23 – 2.17 (m, 2H), 1.99 – 1.93 (m, 2H), 1.73 – 1.66 (m, 2H), 1.59 – 1.52 (m, 2H).

^{13}C NMR {^{1}H} (126 MHz, CD_2Cl_2, 298 K, δ): 142.0, 137.1, 135.4, 135.2, 130.7, 130.5, 123.8,

117.1, 27.7, 25.9, 23.0, 20.8.

^{19}F NMR (471 MHz, CD_2Cl_2, 298 K, δ): –149.84 (bs), –149.92 (bs).

HRMS-ESI (m/z) calc'd. for $C_{18}H_{17}S_2^+$ [M]$^+$, 297.07662; found, 297.07647; deviation: +0.51 ppm.

Cyclopentene-derived thianthrenium salt 21-TT

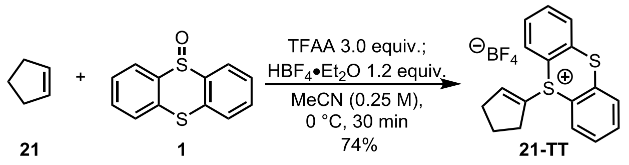

Under ambient atmosphere, a 20 mL borosilicate vial equipped with a magnetic stir bar was charged with cyclopentene (45.8 µL, 34.1 mg, 0.500 mmol, 1.00 equiv.), thianthrene S-oxide (**1**) (120 mg, 0.517 mmol, 1.03 equiv.), and MeCN (2.0 mL, c = 0.25 M). After cooling to 0°C, trifluoroacetic anhydride (0.21 mL, 0.31 g, 1.5 mmol, 3.0 equiv.) was added dropwise within 30 seconds, followed by dropwise addition of HBF_4•OEt_2 (87 µL, 0.60 mmol, 1.2 equiv.) within 10 seconds. After stirring the lilac mixture at 0 °C for 30 min, the resulting light purple mixture was concentrated under reduced pressure and subsequently diluted with CH_2Cl_2 (10 mL). The CH_2Cl_2 solution was poured onto a saturated aqueous $NaHCO_3$ solution (ca. 20 mL). The combined mixture was poured into a separatory funnel, and the layers were separated. The CH_2Cl_2 layer was collected, and the aqueous layer was further extracted with CH_2Cl_2 (2 × ca. 10 mL). The combined CH_2Cl_2 solution was washed with aqueous $NaBF_4$ solution (2 × ca. 20 mL, 5 % w/w). The CH_2Cl_2 layer was dried over Na_2SO_4, filtered, and the solvent was removed under reduced pressure. The residue was purified by chromatography on silica gel eluting with CH_2Cl_2/*i*-PrOH (100:1, v/v). The product-containing fractions were collected and concentrated under reduced pressure. The residue was further dried in vacuo to afford **21-TT** (136.6 mg, 369 µmol, 74 %) as a colorless solid.

R*f* = 0.46 (CH_2Cl_2/MeOH, 15:1, v/v).

NMR Spectroscopy:

^{1}H NMR (500 MHz, CD_2Cl_2, 298 K, δ): 8.22 (dd, *J* = 7.9, 1.4 Hz, 2H), 7.87 (dd, *J* = 8.0, 1.4 Hz, 2H), 7.82 (td, *J* = 7.6, 1.4 Hz, 2H), 7.72 (td, *J* = 7.7, 1.4 Hz, 2H), 6.27 (p, *J* = 2.2 Hz, 1H), 2.55 – 2.50 (m, 2H), 2.38 – 2.33 (m, 2H), 2.03 (p, *J* = 7.6 Hz, 2H).

^{13}C NMR {^{1}H} (126 MHz, CD_2Cl_2, 298 K, δ): 148.5, 136.9, 135.3, 134.4, 130.6, 130.5, 124.7, 117.7, 34.0, 32.9, 23.8.

^{19}F NMR (471 MHz, CD_2Cl_2, 298 K, δ): –151.52 (bs), –151.59 (bs).

HRMS-ESI (m/z) calc'd. for $C_{17}H_{15}S_2^+$ [M]$^+$, 283.06097; found, 283.06045; deviation: +1.84 ppm.

(*E,E,E*)-1,5,9-Cyclododecatriene-derived thianthrenium salt 22-TT

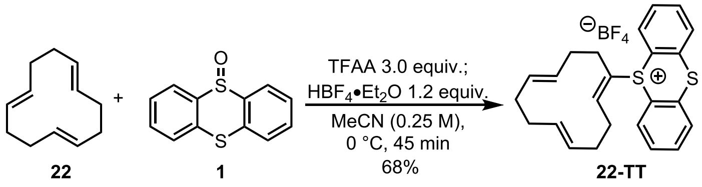

Under ambient atmosphere, a 20 mL borosilicate vial equipped with a magnetic stir bar was charged with (*E,E,E*)-1,5,9-cyclododecatriene (91.2 µL, 81.1 mg, 0.500 mmol, 1.00 equiv.), thianthrene S-oxide (**1**) (120 mg, 0.517 mmol, 1.03 equiv.), and MeCN (2.0 mL, c = 0.25 M). After cooling to 0°C, trifluoroacetic anhydride (0.21 mL, 0.31 g, 1.5 mmol, 3.0 equiv.) was added dropwise within 30 seconds, followed by dropwise addition of $HBF_4·OEt_2$ (87 µL, 0.60 mmol, 1.2 equiv.) within 10 seconds. After stirring the lilac mixture at 0 °C for 45 min, the resulting purple mixture was concentrated under reduced pressure and subsequently diluted with CH_2Cl_2 (10 mL). The CH_2Cl_2 solution was poured onto a saturated aqueous $NaHCO_3$ solution (ca. 20 mL). The combined mixture was poured into a separatory funnel, and the layers were separated. The CH_2Cl_2 layer was collected, and the aqueous layer was further extracted with CH_2Cl_2 (2 × ca. 10 mL). The combined CH_2Cl_2 solution was washed with aqueous $NaBF_4$ solution (2 × ca. 20 mL, 5 % w/w). The CH_2Cl_2 layer was dried over Na_2SO_4, filtered, and the solvent was removed under reduced pressure. The residue was purified by chromatography on silica gel eluting with CH_2Cl_2/*i*-PrOH (100:1, v/v). The product-containing fractions were collected and concentrated under reduced pressure. The residue was further dried in vacuo to afford **22-TT** (157.4 mg, 339 µmol, 68 %) as a colorless solid.

R*f* = 0.46 (CH_2Cl_2/MeOH, 15:1, v/v).

NMR Spectroscopy:

^{1}H NMR (500 MHz, CD_2Cl_2, 298 K, δ): 8.06 – 8.02 (m, 2H), 7.83 – 7.77 (m, 4H), 7.70 (m, *J* = 8.1, 6.4, 2.4 Hz, 2H), 6.03 (t, *J* = 7.4 Hz, 1H), 5.31 – 5.25 (m, 1H), 5.21 – 5.10 (m, 1H), 4.94 – 4.84 (m, 1H), 4.71 – 4.59 (m, 1H), 2.84 (td, *J* = 7.2, 5.4 Hz, 2H), 2.46 – 2.40 (m, 2H), 2.35 (m, *J* = 6.6 Hz, 2H), 2.11 – 2.04 (m, 2H), 2.01 (m, *J* = 7.1, 5.9, 3.3 Hz, 2H), 1.68 (m, *J* = 6.7 Hz, 2H).

^{13}C NMR {^{1}H} (126 MHz, CD_2Cl_2, 298 K, δ): 154.1, 137.0, 135.2, 134.4, 134.2, 134.0, 130.6, 130.4, 130.0, 129.6, 128.6, 117.3, 35.1, 32.6, 32.1, 32.0, 31.9, 30.3.

^{19}F NMR (471 MHz, CD_2Cl_2, 298 K, δ): –152.13 (bs), –152.18 (bs).

HRMS-ESI (m/z) calc'd. for $C_{24}H_{25}S_2^+$ $[M]^+$, 377.13922; found, 377.13857; deviation: +1.72 ppm.

1,9-Cyclohexadecadiene-derived thianthrenium salt 23-TT

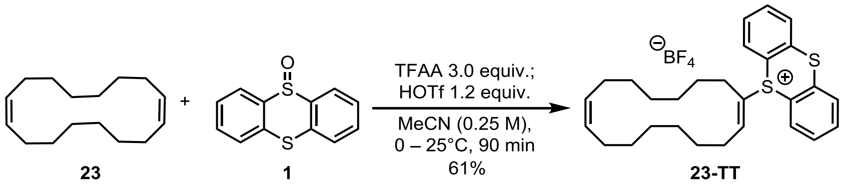

Under ambient atmosphere, a 20 mL borosilicate vial equipped with a magnetic stir bar was charged with 1,9-cyclohexadecadiene (110 mg, 0.500 mmol, 1.00 equiv.), thianthrene S-oxide (**1**) (120 mg, 0.517 mmol, 1.03 equiv.), and MeCN (2.0 mL, c = 0.25 M). After cooling to 0°C, trifluoroacetic anhydride (0.21 mL, 0.31 g, 1.5 mmol, 3.0 equiv.) was added dropwise within 30 seconds, followed by dropwise addition of HOTf (52 µL, 88 mg, 0.59 mmol, 1.2 equiv.) within 10 seconds. After stirring the lilac mixture at 0 °C for 60 min followed by stirring at 25 °C for 30 min, the resulting purple mixture was concentrated under reduced pressure and subsequently diluted with CH_2Cl_2 (10 mL). The CH_2Cl_2 solution was poured onto a saturated aqueous $NaHCO_3$ solution (ca. 20 mL). The combined mixture was poured into a separatory funnel, and the layers were separated. The CH_2Cl_2 layer was collected, and the aqueous layer was further extracted with CH_2Cl_2 (2 × ca. 10 mL). The combined CH_2Cl_2 solution was washed with aqueous $NaBF_4$ solution (2 × ca. 20 mL, 5 % w/w). The CH_2Cl_2 layer was dried over Na_2SO_4, filtered, and the solvent was removed under reduced pressure. The residue was purified by chromatography on silica gel eluting with CH_2Cl_2/*i*-PrOH (100:1, v/v). The product-containing fractions were collected and concentrated under reduced pressure. The residue was further dried in vacuo to afford **23-TT** (159.0 mg, 304 µmol, 61 %) as a colorless solid.

R*f* = 0.46 (CH_2Cl_2/MeOH, 15:1, v/v).

NMR Spectroscopy:

^{1}H NMR (500 MHz, CD_2Cl_2, 298 K, δ): 8.22 (d, *J* = 7.9 Hz, 2H), 7.89 – 7.80 (m, 4H), 7.72 (t, *J* = 7.6 Hz, 2H), 5.51 (t, *J* = 8.3 Hz, 1H), 5.31 – 5.24 (m, 2H), 2.25 – 2.15 (m, 2H), 2.08 (q, *J* = 8.0 Hz, 2H), 1.97 (m, 4H), 1.35 – 1.10 (m, 16H).

^{13}C NMR {^{1}H} (126 MHz, CD_2Cl_2, 298 K, δ): 142.2, 137.3, 135.4, 135.3, 130.7, 130.5, 130.4, 130.1, 123.7, 116.9, 29.3, 29.1, 28.9, 28.5, 28.3, 28.08, 28.03, 28.01, 27.97, 27.7, 27.1, 26.9.

^{19}F NMR (471 MHz, CD_2Cl_2, 298 K, δ): –151.37 (bs), –151.42 (bs).

HRMS-ESI (m/z) calc'd. for $C_{28}H_{35}S_2^+$ $[M]^+$, 435.21747; found, 435.21674; deviation: +1.68 ppm.

1,5-Cyclooctadiene-derived thianthrenium salt 24-TT

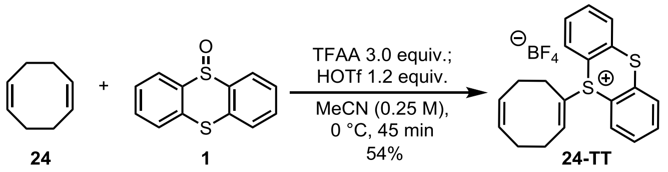

Under ambient atmosphere, a 20 mL borosilicate vial equipped with a magnetic stir bar was charged with 1,5-cyclooctadiene (61.3 µL, 54.1 mg, 0.500 mmol, 1.00 equiv.), thianthrene S-oxide (**1**) (120 mg, 0.517 mmol, 1.03 equiv.), and MeCN (2.0 mL, c = 0.25 M). After cooling to 0°C, trifluoroacetic anhydride (0.21 mL, 0.31 g, 1.5 mmol, 3.0 equiv.) was added dropwise within 30 seconds, followed by dropwise addition of HOTf (52 µL, 88 mg, 0.59 mmol, 1.2 equiv.) within 10 seconds. After stirring the lilac mixture at 0 °C for 45 min, the resulting purple mixture was concentrated under reduced pressure and subsequently diluted with CH_2Cl_2 (10 mL). The CH_2Cl_2 solution was poured onto a saturated aqueous $NaHCO_3$ solution (ca. 20 mL). The

combined mixture was poured into a separatory funnel, and the layers were separated. The CH_2Cl_2 layer was collected, and the aqueous layer was further extracted with CH_2Cl_2 (2 × ca. 10 mL). The combined CH_2Cl_2 solution was washed with aqueous $NaBF_4$ solution (2 × ca. 20 mL, 5 % w/w). The CH_2Cl_2 layer was dried over Na_2SO_4, filtered, and the solvent was removed under reduced pressure. The residue was purified by chromatography on silica gel eluting with CH_2Cl_2/*i*-PrOH (100:1, v/v). The product-containing fractions were collected and concentrated under reduced pressure. The residue was further dried in vacuo to afford **24-TT** (110.0 mg, 268 µmol, 54 %) as a colorless solid.

R*f* = 0.46 (CH_2Cl_2/MeOH, 15:1, v/v).

NMR Spectroscopy:

^{1}H NMR (500 MHz, $CDCl_3$, 298 K, δ): 8.37 – 8.31 (m, 2H), 7.81 – 7.75 (m, 4H), 7.70 (ddd, *J* = 8.6, 6.8, 2.1 Hz, 2H), 5.84 (t, *J* = 6.5 Hz, 1H), 5.53 – 5.44 (m, 1H), 5.32 – 5.24 (m, 1H), 2.64 (t, *J* = 6.9 Hz, 2H), 2.49 (q, *J* = 6.6 Hz, 2H), 2.33 (q, *J* = 6.9 Hz, 2H), 2.02 (q, *J* = 6.8 Hz, 2H).

^{13}C NMR {^{1}H} (126 MHz, $CDCl_3$, 298 K, δ): 142.2, 136.8, 135.6, 135.1, 130.5, 130.2, 129.0, 127.3, 124.1, 117.5, 29.6, 28.9, 27.1, 26.1.

^{19}F NMR (471 MHz, $CDCl_3$, 298 K, δ): –151.67 (bs), –151.72 (bs).

HRMS-ESI (m/z) calc'd. for $C_{20}H_{19}S_2^+$ $[M]^+$, 323.09227; found, 323.09220; deviation: +0.22 ppm.

Tricyclo[6.2.1.02,7]undeca-4-ene-derived thianthrenium salt 25-TT

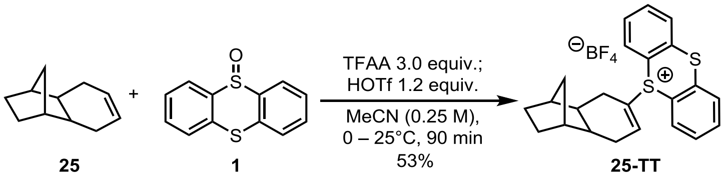

Under ambient atmosphere, a 20 mL borosilicate vial equipped with a magnetic stir bar was charged with tricyclo[6.2.1.02,7]undeca-4-ene (74.1 mg, 0.500 mmol, 1.00 equiv.), thianthrene S-oxide (**1**) (120 mg, 0.517 mmol, 1.03 equiv.), and MeCN (2.0 mL, c = 0.25 M). After cooling to 0°C, trifluoroacetic anhydride (0.21 mL, 0.31 g, 1.5 mmol, 3.0 equiv.) was added dropwise within 30 seconds, followed by dropwise addition of HBF_4•OEt_2 (87 µL, 0.59 mmol, 1.2 equiv.) within 10 seconds. After stirring the lilac mixture at 0 °C for 60 min followed by stirring at 25 °C for 30 min, the resulting purple mixture was concentrated under reduced pressure and subsequently diluted with CH_2Cl_2 (10 mL). The CH_2Cl_2 solution was poured onto a saturated aqueous $NaHCO_3$ solution (ca. 20 mL). The combined mixture was poured into a separatory funnel, and the layers were separated. The CH_2Cl_2 layer was collected, and the aqueous layer was further extracted with CH_2Cl_2 (2 × ca. 10 mL). The combined CH_2Cl_2 solution was washed with aqueous $NaBF_4$ solution (2 × ca. 20 mL, 5 % w/w). The CH_2Cl_2 layer was dried over Na_2SO_4, filtered, and the solvent was removed under reduced pressure. The residue was purified by chromatography on silica gel eluting with CH_2Cl_2/*i*-PrOH (100:1, v/v). The product-containing fractions were collected and concentrated under reduced pressure. The residue was further dried in vacuo to afford **25-TT** (119.1 mg, 265 µmol, 53 %) as a colorless solid.

R*f* = 0.46 (CH_2Cl_2/MeOH, 15:1, v/v).

NMR Spectroscopy:

^{1}H NMR (500 MHz, CD_2Cl_2, 298 K, δ): 8.19 (dd, *J* = 8.0, 1.4 Hz, 2H), 7.87 (ddd, *J* = 8.0, 3.8, 1.4 Hz, 2H), 7.81 (tdd, *J* = 7.8, 3.1, 1.4 Hz, 2H), 7.73 (tdd, *J* = 7.5, 3.0, 1.4 Hz, 2H), 6.01 (dd, *J* = 7.6, 2.4 Hz, 1H), 2.49 – 2.42 (m, 1H), 2.25 – 2.17 (m, 1H), 1.95 – 1.90 (m, 1H), 1.89 – 1.83 (m, 1H), 1.66 – 1.54 (m, 4H), 1.51 – 1.40 (m, 3H), 1.18 – 1.06 (m, 2H), 1.05 – 0.98 (m, 1H).

^{13}C NMR {^{1}H} (126 MHz, CD_2Cl_2, 298 K, δ): 142.9, 136.9, 135.4, 135.3, 135.2, 134.9, 130.8, 130.7, 130.5, 121.3, 117.5, 117.3, 44.4, 43.6, 43.3, 42.6, 33.6, 31.1, 29.7, 29.6, 29.5.

^{19}F NMR (471 MHz, CD_2Cl_2, 298 K, δ): –151.72 (bs), –151.78 (bs).

HRMS-ESI (m/z) calc'd. for $C_{23}H_{23}S_2^+$ $[M]^+$, 363.12357; found, 363.12354; deviation: +0.08 ppm.

Nicotinic acid-derived thianthrenium salt 26-TT

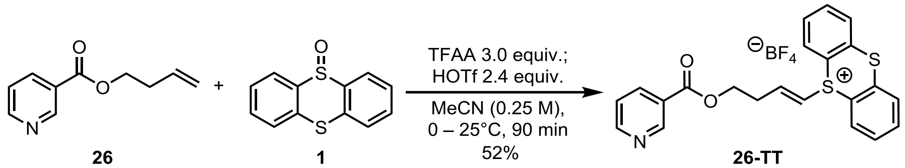

Under ambient atmosphere, a 20 mL borosilicate vial equipped with a magnetic stir bar was charged with nicotinic acid-derived alkene **26** (88.5 mg, 0.500 mmol, 1.00 equiv.), thianthrene S-oxide (**1**) (120 mg, 0.517 mmol, 1.03 equiv.), and MeCN (2.0 mL, c = 0.25 M). After cooling to 0°C, HOTf (52 µL, 88 mg, 0.59 mmol, 1.2 equiv.) was added in one portion. Subsequently, trifluoroacetic anhydride (0.21 mL, 0.31 g, 1.5 mmol, 3.0 equiv.) was added dropwise within 30 seconds, followed by dropwise addition of HOTf (52 µL, 88 mg, 0.59 mmol, 1.2 equiv.) within 10 seconds. After stirring the lilac mixture at 0 °C for 60 min followed by stirring at 25 °C for 30 min, the resulting purple mixture was concentrated under reduced pressure and subsequently diluted with CH_2Cl_2 (10 mL). The CH_2Cl_2 solution was poured onto a saturated aqueous $NaHCO_3$ solution (ca. 20 mL). The combined mixture was poured into a separatory funnel, and the layers were separated. The CH_2Cl_2 layer was collected, and the aqueous layer was further extracted with CH_2Cl_2 (2 × ca. 10 mL). The combined CH_2Cl_2 solution was washed with aqueous $NaBF_4$ solution (2 × ca. 20 mL, 5 % w/w). The CH_2Cl_2 layer was dried over Na_2SO_4, filtered, and the solvent was removed under reduced pressure. The residue was purified by chromatography on silica gel eluting with CH_2Cl_2/*i*-PrOH (100:1, v/v). The product-containing fractions were collected and concentrated under reduced pressure. The residue was further dried in vacuo to afford **26-TT** (*E*/*Z* ≅ 20/1, 124.5 mg, 260 µmol, 52 %) as a colorless solid. **26-TT** was further purified by chromatography on silica gel eluting with CH_2Cl_2/*i*-PrOH (100:1, v/v), and fractions containing **26-TT(*E*)** were collected and concentrated under reduced pressure. The residue was further dried in vacuo.

R*f* = 0.30 (CH_2Cl_2/MeOH, 15:1, v/v).

NMR Spectroscopy:

^{1}H NMR (500 MHz, CD_2Cl_2, 298 K, δ): 8.98 (d, *J* = 2.2 Hz, 1H), 8.77 (dd, *J* = 4.9, 1.7 Hz, 1H), 8.24 (dd, *J* = 7.9, 1.5 Hz, 2H), 8.15 (dt, *J* = 7.9, 2.0 Hz, 1H), 7.82 (dd, *J* = 7.9, 1.4 Hz, 2H), 7.75 (td, *J* = 7.7, 1.4 Hz, 2H), 7.68 (td, *J* = 7.7, 1.4 Hz, 2H), 7.37 (ddd, *J* = 7.9, 4.8, 0.9 Hz, 1H), 7.10 (dt, *J* = 14.9, 7.0 Hz, 1H), 6.68 (dt, *J* = 14.8, 1.4 Hz, 1H), 4.43 (t, *J* = 6.0 Hz, 2H), 2.79 –2.74 (m, 2H).

^{13}C NMR {^{1}H} (126 MHz, CD_2Cl_2, 298 K, δ): 165.23, 153.9, 151.6, 150.9, 137.5, 136.2, 135.2, 133.9, 130.8, 130.7, 120.0, 112.7, 62.4, 32.9.

^{19}F NMR (471 MHz, CD_2Cl_2, 298 K, δ): –150.60 (bs), –150.66 (bs).

HRMS-ESI (m/z) calc'd. for $C_{22}H_{18}NO_2S_2^+$ $[M]^+$, 392.07734; found, 392.07726; deviation: +0.23 ppm.

Cinchophene-derived thianthrenium salt 27-TT

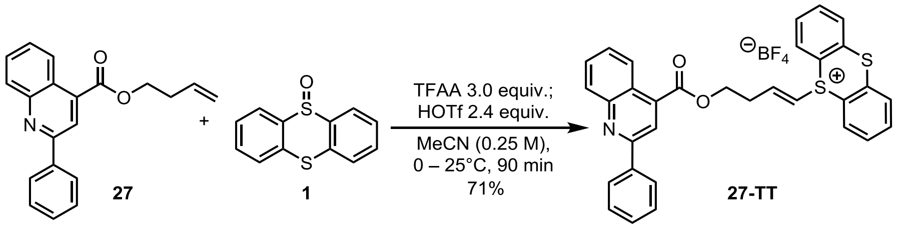

Under ambient atmosphere, a 20 mL borosilicate vial equipped with a magnetic stir bar was charged with Cinchophene-derived alkene **27** (152 mg, 0.500 mmol, 1.00 equiv.), thianthrene S-oxide (**1**) (120 mg, 0.517 mmol, 1.03 equiv.), and MeCN (2.0 mL, c = 0.25 M). After cooling to 0°C, HOTf (52 µL, 88 mg, 0.59 mmol, 1.2 equiv.) was added in one portion. Subsequently, trifluoroacetic anhydride (0.21 mL, 0.31 g, 1.5 mmol, 3.0 equiv.) was added dropwise within 30 seconds, followed by dropwise addition of HOTf (52 µL, 88 mg, 0.59 mmol, 1.2 equiv.) within 10 seconds. After stirring the lilac mixture at 0 °C for 60 min followed by stirring at 25 °C for 30 min, the resulting purple mixture was concentrated under reduced pressure and subsequently diluted with CH_2Cl_2 (10 mL). The CH_2Cl_2 solution was poured onto a saturated aqueous $NaHCO_3$ solution (ca. 20 mL). The combined mixture was poured into a separatory funnel, and the layers were separated. The CH_2Cl_2 layer was collected, and the aqueous layer was further extracted with CH_2Cl_2 (2 × ca. 10 mL). The combined CH_2Cl_2 solution was washed with aqueous $NaBF_4$ solution (2 × ca. 20 mL, 5 % w/w). The CH_2Cl_2 layer was dried over Na_2SO_4, filtered, and the solvent was removed under reduced pressure. The residue was purified by chromatography on silica gel eluting with CH_2Cl_2/*i*-PrOH (100:1, v/v). The product-containing fractions were collected and concentrated under reduced pressure. The residue was further dried in vacuo to afford **27-TT** (*E*/*Z* > 50/1, 214.1 mg, 354 µmol, 71 %) as a colorless solid.

R*f* = 0.42 (CH_2Cl_2/MeOH, 15:1, v/v).

NMR Spectroscopy:

^{1}H NMR (500 MHz, CD_2Cl_2, 298 K, δ): 8.58 (ddd, *J* = 8.6, 1.4, 0.6 Hz, 1H), 8.32 – 8.17 (m, 4H), 8.08 (dt, *J* = 7.6, 1.0 Hz, 2H), 7.81 (ddd, *J* = 8.4, 6.9, 1.4 Hz, 1H), 7.69 – 7.56 (m, 4H), 7.54 – 7.45 (m, 4H), 7.41 (ddd, *J* = 7.9, 5.9, 3.0 Hz, 2H), 7.18 (dt, *J* = 14.4, 7.0 Hz, 1H), 6.74 (dt, *J* = 14.8, 1.3 Hz, 1H), 4.61 (t, *J* = 6.0 Hz, 2H), 2.97 – 2.83 (m, 2H).

^{13}C NMR {^{1}H} (126 MHz, CD_2Cl_2, 298 K, δ): 166.2, 156.9, 151.8, 149.6, 138.9, 136.0, 135.6, 134.9, 133.7, 130.70, 130.66, 130.55, 130.47, 130.45, 129.6, 128.5, 128.0, 125.7, 124.3, 120.4, 120.0, 112.7, 62.85, 33.07.

^{19}F NMR (471 MHz, CD_2Cl_2, 298 K, δ): –150.54 (bs), –150.59 (bs).

HRMS-ESI (m/z) calc'd. for $C_{32}H_{24}NO_2S_2^+$ $[M]^+$, 518.12429; found, 518.12406; deviation: +0.46 ppm.

Lithocholic acid-derived thianthrenium salt 28-TT

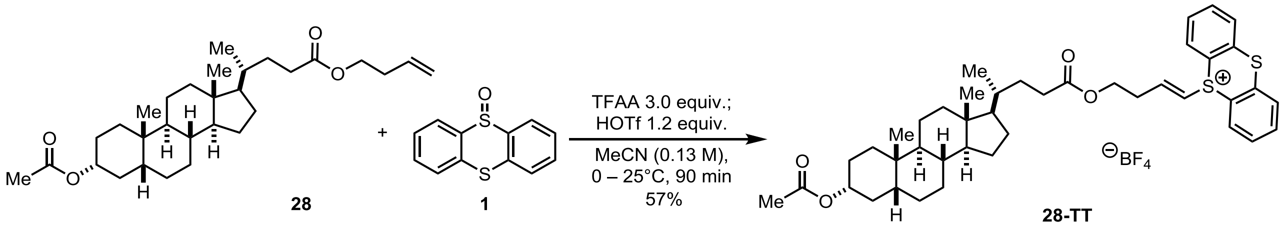

Under ambient atmosphere, a 20 mL borosilicate vial equipped with a magnetic stir bar was charged with Lithocholic acid-derived alkene **28** (118 mg, 0.250 mmol, 1.00 equiv.), thianthrene S-oxide (**1**) (60 mg, 0.258 mmol, 1.03 equiv.), and MeCN (2.0 mL, c = 0.13 M). After cooling to 0°C, trifluoroacetic anhydride (0.11 mL, 0.16 g, 0.75 mmol, 3.0 equiv.) was added dropwise within 30 seconds, followed by dropwise addition of HOTf (26 µL, 44 mg, 0.30 mmol, 1.2 equiv.) within 10 seconds. After stirring the lilac mixture at 0 °C for 60 min followed by stirring at 25 °C for 30 min, the resulting purple mixture was concentrated under reduced pressure and subsequently diluted with CH_2Cl_2 (10 mL). The CH_2Cl_2 solution was poured onto a saturated aqueous $NaHCO_3$ solution (ca. 20 mL). The combined mixture was poured into a separatory funnel, and the layers were separated. The CH_2Cl_2 layer was collected, and the aqueous layer was further extracted with CH_2Cl_2 (2 × ca. 10 mL). The combined CH_2Cl_2 solution was washed with aqueous $NaBF_4$ solution (2 × ca. 20 mL, 5 % w/w). The CH_2Cl_2 layer was dried over Na_2SO_4, filtered, and the solvent was removed under reduced pressure. The residue was purified by chromatography on silica gel eluting with CH_2Cl_2/*i*-PrOH (100:1, v/v). The product-containing fractions were collected and concentrated under reduced pressure. The residue was further dried in vacuo to afford **28-TT** (*E*/*Z* > 50/1, 109.7 mg, 142 µmol, 57 %) as a colorless solid.

R*f* = 0.46 (CH_2Cl_2/MeOH, 15:1, v/v).

NMR Spectroscopy:

^{1}H NMR (500 MHz, CD_2Cl_2, 298 K, δ): 8.25 (dd, *J* = 7.9, 1.2 Hz, 2H), 7.88 (dd, *J* = 7.9, 1.2 Hz, 2H), 7.78 (td, *J* = 7.7, 1.3 Hz, 2H), 7.70 (td, *J* = 7.7, 1.2 Hz, 2H), 7.03 (dt, *J* = 14.9, 6.8 Hz, 1H), 6.63 (dt, *J* = 14.9, 1.3 Hz, 1H), 4.66 (ddt, *J* = 16.1, 11.0, 4.7 Hz, 1H), 4.10 (t, *J* = 6.2 Hz, 2H), 2.59 (q, *J* = 6.3 Hz, 2H), 2.24 – 2.16 (m, 1H), 2.11 – 2.03 (m, 1H), 1.97 (s, 3H), 1.96 – 1.92 (m, 1H), 1.89 – 1.73 (m, 5H), 1.68 – 1.47 (m, 4H), 1.46 – 1.30 (m, 7H), 1.20 (m, 5H), 1.11 – 1.00 (m, 5H), 0.92 (s, 3H), 0.84 (d, *J* = 6.5 Hz, 3H), 0.63 (s, 3H).

^{13}C NMR {^{1}H} (126 MHz, CD_2Cl_2, 298 K, δ): 174.18, 170.82, 152.38, 136.38, 135.16, 133.99,

130.83, 130.79, 120.40, 112.11, 74.7, 61.3, 57.0, 56.5, 43.2, 42.5, 40.9, 40.7, 36.3, 35.9, 35.5, 35.1, 33.0, 32.8, 31.4, 31.3, 28.7, 27.6, 27.2, 26.9, 24.7, 23.6, 21.8, 21.3, 18.6, 12.3.

^{19}F NMR (471 MHz, CD_2Cl_2, 298 K, δ): –150.72 (bs), –150.77 (bs).

HRMS-ESI (m/z) calc'd. for $C_{42}H_{55}O_4S_2^+$ $[M]^+$, 687.35363; found, 687.35379; deviation: –0.23 ppm.

Epiandrosterone-derived thianthrenium salt 29-TT

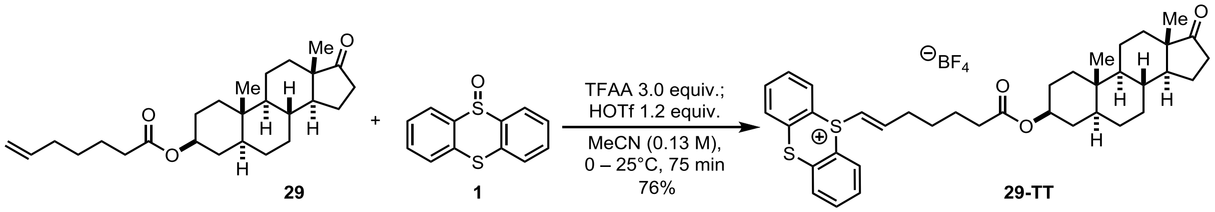

Under ambient atmosphere, a 20 mL borosilicate vial equipped with a magnetic stir bar was charged with Epiandrosterone-derived alkene **29** (100 mg, 0.250 mmol, 1.00 equiv.), thianthrene S-oxide (**1**) (60 mg, 0.258 mmol, 1.03 equiv.), and MeCN (2.0 mL, c = 0.13 M). After cooling to 0°C, trifluoroacetic anhydride (0.11 mL, 0.16 g, 0.75 mmol, 3.0 equiv.) was added dropwise within 30 seconds, followed by dropwise addition of HOTf (26 µL, 44 mg, 0.30 mmol, 1.2 equiv.) within 10 seconds. After stirring the lilac mixture at 0 °C for 60 min followed by stirring at 25 °C for 15 min, the resulting pink mixture was concentrated under reduced pressure and subsequently diluted with CH_2Cl_2 (10 mL). The CH_2Cl_2 solution was poured onto a saturated aqueous $NaHCO_3$ solution (ca. 20 mL). The combined mixture was poured into a separatory funnel, and the layers were separated. The CH_2Cl_2 layer was collected, and the aqueous layer was further extracted with CH_2Cl_2 (2 × ca. 10 mL). The combined CH_2Cl_2 solution was washed with aqueous $NaBF_4$ solution (2 × ca. 20 mL, 5 % w/w). The CH_2Cl_2 layer was dried over Na_2SO_4, filtered, and the solvent was removed under reduced pressure. The residue was purified by chromatography on silica gel eluting with CH_2Cl_2/*i*-PrOH (100:1, v/v). The product-containing fractions were collected and concentrated under reduced pressure. The residue was further dried in vacuo to afford **29-TT** (*E*/*Z* ≅ 25/1, 134.2 mg, 191 µmol, 76 %) as a colorless solid.

R*f* = 0.46 (CH_2Cl_2/MeOH, 15:1, v/v).

NMR Spectroscopy:

^{1}H NMR (500 MHz, CD_2Cl_2, 298 K, δ): 8.23 (dd, *J* = 7.9, 1.3 Hz, 2H), 7.90 (dd, *J* = 7.9, 1.3 Hz, 2H), 7.79 (td, *J* = 7.7, 1.4 Hz, 2H), 7.69 (td, *J* = 7.7, 1.3 Hz, 2H), 7.07 (dt, *J* = 14.9, 6.9 Hz, 1H), 6.58 (dt, *J* = 14.8, 1.5 Hz, 1H), 4.61 (tt, *J* = 11.4, 4.8 Hz, 1H), 2.37 (dd, *J* = 18.9, 9.0 Hz, 1H), 2.29 (qd, *J* = 7.0, 1.6 Hz, 2H), 2.20 (t, *J* = 7.2 Hz, 2H), 2.00 (dt, *J* = 19.0, 9.0 Hz, 1H), 1.94 – 1.87 (m, 1H), 1.81 – 1.69 (m, 4H), 1.65 – 1.59 (m, 1H), 1.57 – 1.41 (m, 7H), 1.37 – 1.15 (m, 7H), 1.05 – 0.92 (m, 2H), 0.83 (s, 3H), 0.82 (s, 3H), 0.70 (ddd, *J* = 12.2, 10.4, 4.1 Hz, 1H).

^{13}C NMR {^{1}H} (126 MHz, CD_2Cl_2, 298 K, δ): 221.0, 172.2, 156.6, 136.3, 135.0, 133.7, 130.8, 130.7, 120.7, 110.2, 73.8, 54.8, 51.8, 48.1, 45.2, 37.1, 36.2, 36.1, 35.5, 34.4, 34.4, 33.4, 32.1,

31.3, 28.8, 27.9, 27.1, 24.7, 22.2, 20.9, 14.1, 12.4.

^{19}F NMR (471 MHz, CD_2Cl_2, 298 K, δ): –150.87 (bs), –150.93 (bs).

HRMS-ESI (m/z) calc'd. for $C_{38}H_{27}O_3S_2^+$ $[M]^+$, 615.29611; found, 615.29608; deviation: +0.06 ppm.

Piperidine-derived thianthrenium salt 30-TT

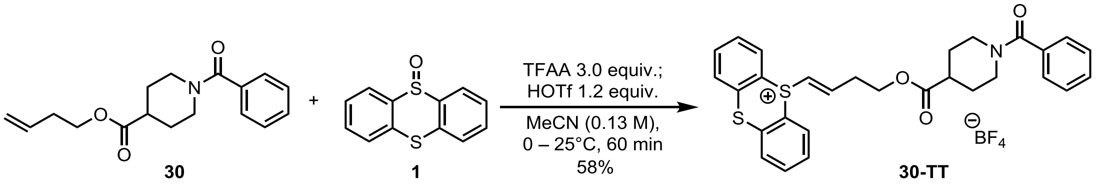

Under ambient atmosphere, a 20 mL borosilicate vial equipped with a magnetic stir bar was charged with piperidine-derived alkene **30** (144 mg, 0.500 mmol, 1.00 equiv.), thianthrene S-oxide (**1**) (120 mg, 0.517 mmol, 1.03 equiv.), and MeCN (2.0 mL, c = 0.25 M). After cooling to 0°C, HOTf (52 µL, 88 mg, 0.59 mmol, 1.2 equiv.) was added in one portion. Subsequently, trifluoroacetic anhydride (0.21 mL, 0.31 g, 1.5 mmol, 3.0 equiv.) was added dropwise within 30 seconds, followed by dropwise addition of HOTf (52 µL, 88 mg, 0.59 mmol, 1.2 equiv.) within 10 seconds. After stirring the lilac mixture at 0 °C for 30 min followed by stirring at 25 °C for 30 min, the resulting lilac mixture was concentrated under reduced pressure and subsequently diluted with CH_2Cl_2 (10 mL). The CH_2Cl_2 solution was poured onto a saturated aqueous $NaHCO_3$ solution (ca. 20 mL). The combined mixture was poured into a separatory funnel, and the layers were separated. The CH_2Cl_2 layer was collected, and the aqueous layer was further extracted with CH_2Cl_2 (2 × ca. 10 mL). The combined CH_2Cl_2 solution was washed with aqueous $NaBF_4$ solution (2 × ca. 20 mL, 5 % w/w). The CH_2Cl_2 layer was dried over Na_2SO_4, filtered, and the solvent was removed under reduced pressure. The residue was purified by chromatography on silica gel eluting with CH_2Cl_2/*i*-PrOH (100:1 to 70:1, v/v). The product-containing fractions were collected and concentrated under reduced pressure. The residue was further dried in vacuo to afford **30-TT** (*E*/*Z* > 50/1, 171.5 mg, 291 µmol, 58 %) as a colorless solid.

R*f* = 0.36 (CH_2Cl_2/MeOH, 20:1, v/v).

NMR Spectroscopy:

^{1}H NMR (600 MHz, CD_3Cl, 298 K, δ): 8.38 (dd, *J* = 7.9, 1.4 Hz, 2H), 7.85 – 7.79 (m, 2H), 7.72 (t, *J* = 7.4 Hz, 2H), 7.67 (td, *J* = 7.6, 1.4 Hz, 2H), 7.44 – 7.41 (m, 3H), 7.40 – 7.37 (m, 2H), 7.30 (dt, *J* = 14.2, 6.8 Hz, 1H), 6.67 (dt, *J* = 14.8, 1.4 Hz, 1H), 4.44 (bs, 1 H), 4.18 (t, *J* = 5.9 Hz, 2H), 3.78 – 3.58 (bs, 1 H), 2.96 (2bs, 1H), 2.65 – 2.59 (m, 2H), 2.57 – 2.48 (m, 1H), 1.95 – 1.67 (2bs, 3H), 1.54 – 1.43 (m, 2H).

^{13}C NMR {^{1}H} (151 MHz, CD_2Cl_2, 298 K, δ): 173.8, 170.4, 152.3, 135.9, 135.5, 134.5, 134.1, 130.4, 130.0, 129.7, 128.6, 126.9, 120.1, 111.5, 61.1, 46.9 (bs), 41.4 (bs), 40.7, 32.4, 28.3 (bs), 27.8 (bs).

^{19}F NMR (471 MHz, CD_2Cl_2, 298 K, δ): –150.24 (bs), –150.59 (bs).

HRMS-ESI (m/z) calc'd. for $C_{29}H_{28}O_3S_2^+$ $[M]^+$, 502.15051; found, 502.15051; deviation: +0.92 ppm.

Fluazinam-derived thianthrenium salt 31-TT

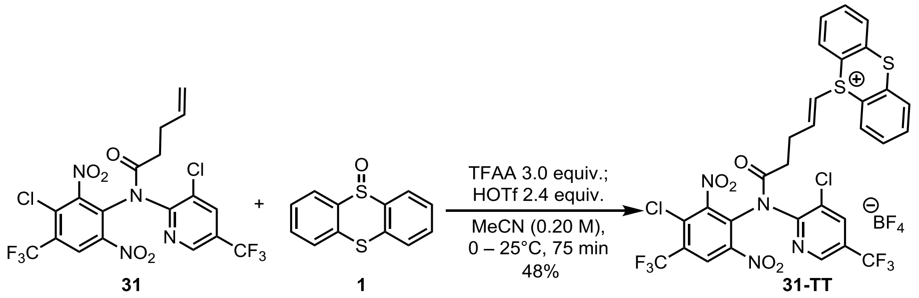

Under ambient atmosphere, a 20 mL borosilicate vial equipped with a magnetic stir bar was charged with Fluazinam-derived alkene **31** (109 mg, 0.200 mmol, 1.00 equiv.), thianthrene S-oxide (**1**) (48 mg, 0.206 mmol, 1.03 equiv.), and MeCN (1.0 mL, c = 0.20 M). After cooling to 0°C, HOTf (20 µL, 35 mg, 0.24 mmol, 1.2 equiv.) within 10 seconds was added dropwise within 30 seconds, followed by dropwise addition of trifluoroacetic anhydride (84 µL, 64 mg, 0.60 mmol, 3.0 equiv.) and HOTf (20 µL, 35 mg, 0.24 mmol, 1.2 equiv.). After stirring the lilac mixture at 0 °C for 45 min followed by stirring at 25 °C for 30 min, the resulting purple mixture was concentrated under reduced pressure and subsequently diluted with CH_2Cl_2 (10 mL). The CH_2Cl_2 solution was poured onto a saturated aqueous $NaHCO_3$ solution (ca. 20 mL). The combined mixture was poured into a separatory funnel, and the layers were separated. The CH_2Cl_2 layer was collected, and the aqueous layer was further extracted with CH_2Cl_2 (2 × ca. 10 mL). The combined CH_2Cl_2 solution was washed with aqueous $NaBF_4$ solution (2 × ca. 20 mL, 5 % w/w). The CH_2Cl_2 layer was dried over Na_2SO_4, filtered, and the solvent was removed under reduced pressure. The residue was purified by chromatography on silica gel eluting with CH_2Cl_2/*i*-PrOH (100:1, v/v). The product-containing fractions were collected and concentrated under reduced pressure. The residue was further dried in vacuo to afford **31-TT** (*E*/*Z* > 50/1, 81.3 mg, 95.8 µmol, 48 %) as a yellow solid.

R*f* = 0.46 (CH_2Cl_2/MeOH, 20:1, v/v).

NMR Spectroscopy:

^{1}H NMR (600 MHz, CD_3Cl, 298 K, δ): 8.74 (s, 1H), 8.40 (s, 1H), 8.23 (d, *J* = 7.9 Hz, 1H), 8.20 (d, *J* = 7.9 Hz, 1H), 7.98 (s, 1H), 7.84 – 7.78 (m, 2H), 7.74 – 7.70 (m, 2H), 7.69 – 7.63 (m, 2H), 7.14 – 7.06 (m, 1H), 6.55 (d, *J* = 14.6 Hz, 1H), 2.91 – 2.79 (m, 2H), 2.78 – 2.70 (m, 1H), 2.60 – 2.51 (m, 1H).

^{13}C NMR {^{1}H} (151 MHz, CD_3Cl, 268 K, δ): 170.3, 154.3, 151.0, 150.6, 147.2, 143.6 (d, *J* = 4.0 Hz), 136.7, 135.7, 135.6, 134.4, 134.3, 134.0, 133.5, 132.5(d, *J* = 34.6 Hz), 130.5, 130.4, 130.25, 130.23, 129.4, 129.3, 129.1, 127.2, 126.7 (q, *J* = 34.1 Hz), 122.1 (d, *J* = 273.2 Hz), 120.6 (d, *J* = 274.9 Hz), 120.2, 119.8, 111.0, 32.6, 27.3.

^{19}F NMR (471 MHz, CD_3Cl, 298 K, δ): –62.29 (s), –63.24 (s), –150.63 (bs), –150.69 (bs).

HRMS-ESI (m/z) calc'd. for $C_{30}H_{17}Cl_2F_6N_4O_5S_2^+$ [M]$^+$, 760.99162; found, 760.99226; deviation: –0.48 ppm.

Probenecid-derived thianthrenium salt 32-TT

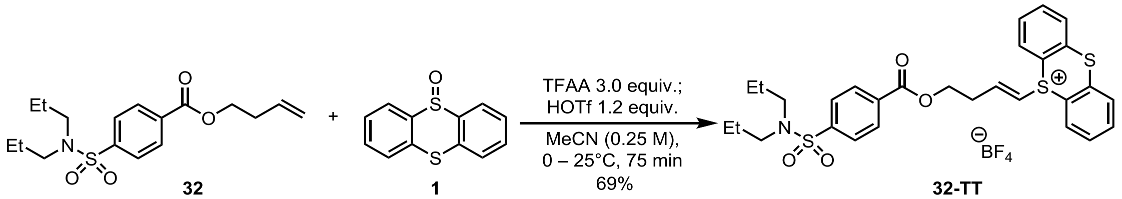

Under ambient atmosphere, a 20 mL borosilicate vial equipped with a magnetic stir bar was charged with Probenecid-derived alkene **32** (170 mg, 0.500 mmol, 1.00 equiv.), thianthrene S-oxide (**1**) (120 mg, 0.517 mmol, 1.03 equiv.), and MeCN (2.0 mL, c = 0.25 M). After cooling to 0°C, trifluoroacetic anhydride (0.21 mL, 0.31 g, 1.5 mmol, 3.0 equiv.) was added dropwise within 30 seconds, followed by dropwise addition of HOTf (52 µL, 88 mg, 0.59 mmol, 1.2 equiv.) within 10 seconds. After stirring the lilac mixture at 0 °C for 45 min followed by stirring at 25 °C for 30 min, the resulting purple mixture was concentrated under reduced pressure and subsequently diluted with CH_2Cl_2 (10 mL). The CH_2Cl_2 solution was poured onto a saturated aqueous $NaHCO_3$ solution (ca. 20 mL). The combined mixture was poured into a separatory funnel, and the layers were separated. The CH_2Cl_2 layer was collected, and the aqueous layer was further extracted with CH_2Cl_2 (2 × ca. 10 mL). The combined CH_2Cl_2 solution was washed with aqueous $NaBF_4$ solution (2 × ca. 20 mL, 5 % w/w). The CH_2Cl_2 layer was dried over Na_2SO_4, filtered, and the solvent was removed under reduced pressure. The residue was purified by chromatography on silica gel eluting with CH_2Cl_2/*i*-PrOH (90:1 to 60:1, v/v). The product-containing fractions were collected and concentrated under reduced pressure. The residue was further dried in vacuo to afford **32-TT** (*E*/*Z* > 50/1, 220.0 mg, 343 µmol, 69 %) as a colorless solid.

R*f* = 0.46 (CH_2Cl_2/MeOH, 20:1, v/v).

NMR Spectroscopy:

^{1}H NMR (500 MHz, CD_3Cl, 298 K, δ): 8.36 – 8.31 (m, 2H), 7.99 (d, *J* = 8.2 Hz, 2H), 7.79 (d, *J* = 8.1 Hz, 2H), 7.76 – 7.68 (m, 4H), 7.64 (td, *J* = 7.5, 1.7 Hz, 2H), 7.29 (dt, *J* = 14.6, 7.2 Hz, 1H), 6.70 (d, *J* = 14.7 Hz, 1H), 4.44 (t, *J* = 6.0 Hz, 2H), 3.15 – 3.06 (m, 4H), 2.76 (q, *J* = 6.3 Hz, 2H), 1.55 (h, *J* = 7.4 Hz, 4H), 0.87 (t, *J* = 7.3 Hz, 6H).

^{13}C NMR {^{1}H} (126 MHz, CD_3Cl, 298 K, δ): 164.8, 151.6, 144.5, 135.5, 134.5, 134.0, 132.8, 130.4, 130.2, 130.0, 127.1, 119.9, 112.0, 62.0, 50.0, 32.4, 22.0, 11.2.

^{19}F NMR (471 MHz, CD_3Cl, 298 K, δ): –150.25 (bs), –150.30 (bs).

HRMS-ESI (m/z) calc'd. for $C_{29}H_{32}NO_4S_3^+$ [M]$^+$, 554.14880; found, 554.14880; deviation: 0.00 ppm.

Bicalutamide-derived thianthrenium salt 33-TT

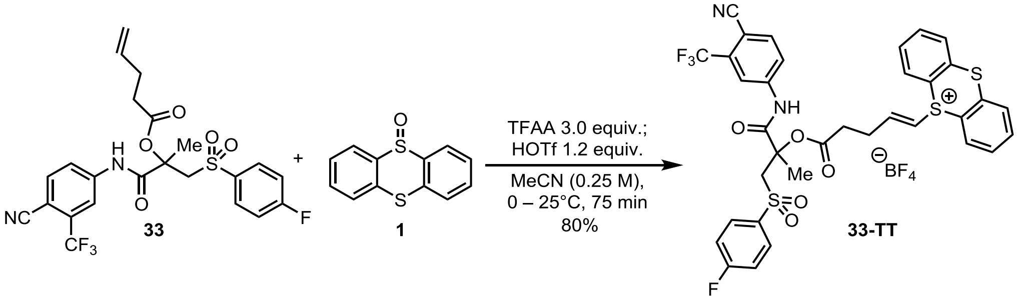

Under ambient atmosphere, a 20 mL borosilicate vial equipped with a magnetic stir bar was charged with Bicalutamide-derived alkene **33** (128 mg, 0.250 mmol, 1.00 equiv.), thianthrene S-oxide (**1**) (60 mg, 0.258 mmol, 1.03 equiv.), and MeCN (2.0 mL, c = 0.13 M). After cooling to 0°C, trifluoroacetic anhydride (0.11 mL, 0.16 g, 0.75 mmol, 3.0 equiv.) was added dropwise within 30 seconds, followed by dropwise addition of HOTf (26 µL, 44 mg, 0.30 mmol, 1.2 equiv.) within 10 seconds. After stirring the lilac mixture at 0 °C for 60 min followed by stirring at 25 °C for 15 min, the resulting pink mixture was concentrated under reduced pressure and subsequently diluted with CH_2Cl_2 (10 mL). The CH_2Cl_2 solution was poured onto a saturated aqueous $NaHCO_3$ solution (ca. 20 mL). The combined mixture was poured into a separatory funnel, and the layers were separated. The CH_2Cl_2 layer was collected, and the aqueous layer was further extracted with CH_2Cl_2 (2 × ca. 10 mL). The combined CH_2Cl_2 solution was washed with aqueous $NaBF_4$ solution (2 × ca. 20 mL, 5 % w/w). The CH_2Cl_2 layer was dried over Na_2SO_4, filtered, and the solvent was removed under reduced pressure. The residue was purified by chromatography on silica gel eluting with CH_2Cl_2/*i*-PrOH (80:1 to 50:1, v/v). The product-containing fractions were collected and concentrated under reduced pressure. The residue was further dried in vacuo to afford **33-TT** (*E*/*Z* > 50/1, 163.2 mg, 200 µmol, 80 %) as a colorless solid.

R*f* = 0.46 (CH_2Cl_2/MeOH, 20:1, v/v).

NMR Spectroscopy:

^{1}H NMR (500 MHz, CD_3Cl, 298 K, δ): 9.34 (s, 1H), 8.24 (d, *J* = 2.2 Hz, 1H), 8.16 (d, *J* = 7.9 Hz, 1H), 8.11 (d, *J* = 7.9 Hz, 1H), 7.91 (dd, *J* = 8.6, 2.1 Hz, 1H), 7.88 – 7.79 (m, 4H), 7.77 – 7.68 (m, 2H), 7.67 – 7.56 (m, 3H), 7.28 (dt, *J* = 14.3, 6.8 Hz, 1H), 7.12 (t, *J* = 8.5 Hz, 2H), 6.74 (d, *J* = 14.7 Hz, 1H), 4.17 (d, *J* = 14.5 Hz, 1H), 4.01 (d, *J* = 14.5 Hz, 1H), 3.02 – 2.93 (m, 1H), 2.91 – 2.82 (m, 1H), 2.78 – 2.55 (m, 2H), 1.70 (s, 3H).

^{13}C NMR {^{1}H} (126 MHz, CD_3Cl, 298 K, δ): 171.0, 169.3, 165.8 (d, *J* = 257.4 Hz), 155.8, 141.9, 135.4, 135.4, 135.3, 135.2, 134.32, 134.25, 133.2, 132.9, 132.7, 132.5, 131.3, 131.2, 130.3, 130.2, 130.1, 130.0, 123.1, 122.2 (q, *J* = 274.1 Hz), 120.2 (d, *J* = 8.7 Hz), 118.1, 116.4 (d, *J* = 22.6 Hz), 115.7, 110.2, 103.9, 79.7, 57.4, 53.4, 31.5, 27.7, 23.9.

^{19}F NMR (471 MHz, CD_3Cl, 298 K, δ): –62.00, –102.47 (m), –148.12 (bs), –148.17 (bs).

HRMS-ESI (m/z) calc'd. for $C_{35}H_{27}N_2O_5S_2F_4^+$ $[M]^+$, 727.10128; found, 727.10209; deviation: –1.11 ppm.

Evaluation on different *S*-oxide

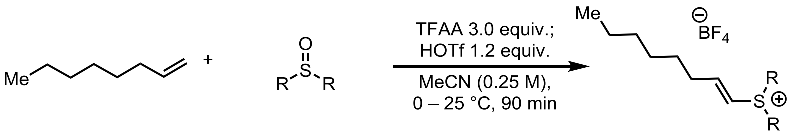

Under ambient atmosphere, a 20 mL borosilicate vial equipped with a magnetic stir bar was charged with 1-octene (78.5 µL, 56.1 mg, 0.500 mmol, 1.00 equiv.), *S*-oxide (0.517 mmol, 1.03 equiv.), and MeCN (2.0 mL, c = 0.25 M). After cooling to 0°C, trifluoroacetic anhydride (0.21 -mL, 0.31 g, 1.5 mmol, 3.0 equiv.) was added dropwise within 30 seconds, followed by dropwise addition of HOTf (52 µL, 88 mg, 0.59 mmol, 1.2 equiv.) within 10 seconds. After stirring the lilac mixture at 0 °C for 60 min followed by stirring at 25 °C for 30 min, the resulting purple mixture was concentrated under reduced pressure and subsequently diluted with CH_2Cl_2 (10 mL). The CH_2Cl_2 solution was poured onto a saturated aqueous $NaHCO_3$ solution (ca. 20 mL). The combined mixture was poured into a separatory funnel, and the layers were separated. The CH_2Cl_2 layer was collected, and the aqueous layer was further extracted with CH_2Cl_2 (2 × ca. 10 mL). The combined CH_2Cl_2 solution was washed with aqueous $NaBF_4$ solution (2 × ca. 20 mL, 5 % w/w). The CH_2Cl_2 layer was dried over Na_2SO_4, filtered, and the solvent was removed under reduced pressure. The residue was checked by TLC and LCMS or purified by chromatography on silica gel to obtain the yield for alkenyl sulfonium salts.

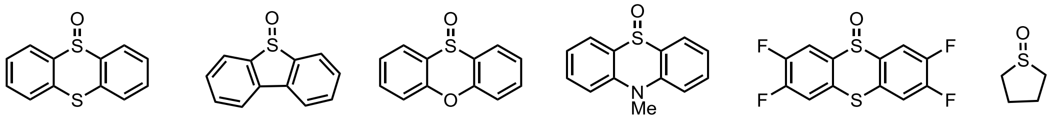

1 **1-2** **1-3** **1-4** **1-5** **1-6**

Entry	yield
**1**	92%[a]
**1-2**	<10%[b]
**1-3**	<10%[b]
**1-4**	0[b]
**1-5**	70%[a]
**1-6**	trace[b]

Table S1: S-oxide screening (a: isolated yields; b: based on LCMS analysis).

Functionalization of alkenyl thianthrenium salts

Cyclopropyl-pent-4-en-1-yl-phthalimide (34)

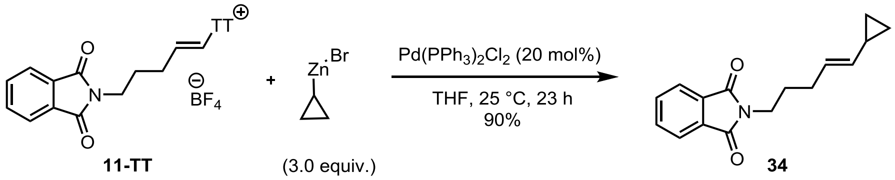

11-TT (3.0 equiv.) **34**

To a 4-mL borosilicate vial equipped with a stir bar was added $Pd(PPh_3)_2Cl_2$ (28 mg, 0.040 mmol, 20 mol%),

and alkenyl thianthrenium salt **11-TT** (104 mg, 0.200 mmol, 1.00 equiv.). The vial was transferred into a N_2-filled glovebox. After addition of cyclopropylzinc bromide solution (1.2 mL, 0.50 M in THF, 0.60 mmol, 3.0 equiv.) and THF (0.80 mL, c = 0.10 M), the vial was capped, transferred out of the glovebox and placed on a stirring plate. After being stirred at 25 °C for 23 h, the reaction mixture was diluted with CH_2Cl_2 (2 mL), and the resulting mixture was filtered through a short pad of Celite® using CH_2Cl_2 (5 mL) as eluent. The filtrate was collected and concentrated by rotary evaporation. The residue was purified by chromatography on silica gel eluting with hexanes/ethyl acetate (40:1 to 10:1, v/v) to afford **34** (46.0 mg, 0.180 mmol, 90%) as a colorless solid.

R*f* = 0.30 (hexanes/ethylacetate, 10:1, v/v).

NMR Spectroscopy:

^{1}H NMR (500 MHz, $CDCl_3$, 298 K, δ): 7.83 (dd, *J* = 5.4, 3.1 Hz, 2H), 7.70 (dd, *J* = 5.5, 3.0 Hz, 2H), 5.47 (dt, *J* = 15.3, 6.7 Hz, 1H), 4.98 (dd, *J* = 15.3, 8.6 Hz, 1H), 3.68 (t, *J* = 7.3 Hz, 2H), 2.10 – 2.00 (m, 2H), 1.74 (p, *J* = 7.4 Hz, 2H), 1.35 – 1.24 (m, 1H), 0.65 – 0.56 (m, 2H), 0.33 – 0.23 (m, 2H).

^{13}C NMR {^{1}H} (126 MHz, $CDCl_3$, 298 K, δ): 168.4, 134.82, 133.8, 132.2, 126.3, 123.1, 37.7, 29.8, 28.2, 13.5, 6.3.

HRMS-ESI (m/z) calc'd. for $C_{16}H_{17}NO_2Na^+$ $[M+Na]^+$, 278.11514; found, 278.11500; deviation: +0.53 ppm.

Phenylacetylenyl-pent-4-en-1-yl-phthalimide (35)

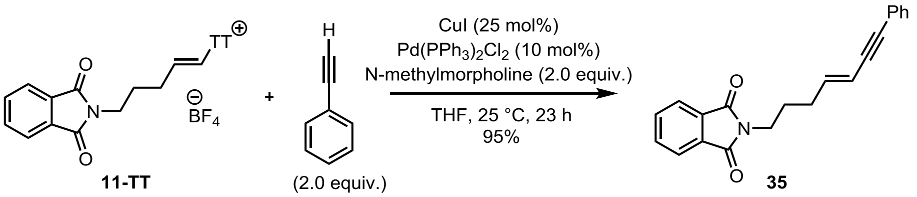

11-TT (2.0 equiv.) **35**

To a 4-mL borosilicate vial equipped with a stir bar was added $Pd(PPh_3)_2Cl_2$ (7.0 mg, 0.010 mmol, 10 mol%), CuI (4.8 mg, 0.025 mmol, 25 mol%), and alkenyl thianthrenium salt **11-TT** (51.7 mg, 0.100 mmol, 1.00 equiv.). The vial was transferred into a N_2-filled glovebox. After addition of THF (1.0 mL, c = 0.10 M), phenylacetylene (22.0 μL, 20.5 mg, 0.200mmol, 2.00 equiv.), and N-methylmorpholine (22.0 μL, 20.3 mg, 0.200 mmol, 2.00 equiv.), the vial was capped, transferred out of the glovebox, and placed on a stirring plate. After being stirred at 25 °C for 23 h, the reaction mixture was diluted with CH_2Cl_2 (2 mL), and the resulting mixture was filtered through a short pad of Celite® using CH_2Cl_2 (5 mL) as eluent. The filtrate was collected and concentrated by rotary evaporation. The residue was purified by chromatography on silica gel eluting with hexanes/ethyl acetate (40:1 to 10:1, v/v) to afford **35** (30.0 mg, 0.095 mmol, 95%, *E*/*Z* ≅ 10/1) as a yellow solid.

R*f* = 0.26 (hexanes/ethylacetate, 10:1, v/v).

NMR Spectroscopy:

^{1}H NMR (500 MHz, $CDCl_3$, 298 K, δ): 7.70 (dd, *J* = 5.5, 3.1 Hz, 2H), 7.56 (dd, *J* = 5.4, 3.0 Hz, 2H), 7.28 – 7.24 (m, 2H), 7.14 (m, 3H), 6.08 (dt, *J* = 15.7, 7.0 Hz, 1H), 5.60 (dt, *J* = 15.8, 1.6 Hz, 1H),

3.58 (t, J = 7.1 Hz, 2H), 2.15 – 2.05 (m, 2H), 1.69 (p, J = 7.3 Hz, 2H).

^{13}C NMR {^{1}H} (126 MHz, $CDCl_3$, 298 K, δ): 168.3, 142.9, 133.9, 132.1, 131.4, 128.2, 127.9, 123.4, 123.2, 110.6, 88.3, 87.9, 37.4, 30.5, 27.

HRMS-ESI (m/z) calc'd. for $C_{21}H_{17}NO_2Na^+$ $[M+Na]^+$, 338.11514; found, 338.11551; deviation: –1.07 ppm.

Vinylnaphthalenyl-pent-4-en-1-yl-phthalimide (36)

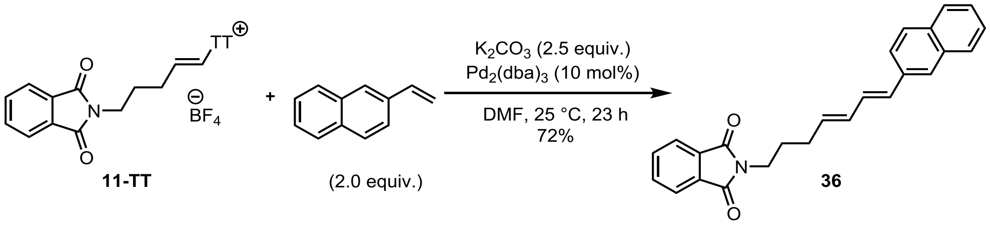

11-TT (2.0 equiv.) **36**

To a 4-mL borosilicate vial equipped with a stir bar was added $Pd_2(dba)_3$ (14 mg, 0.010 mmol, 10 mol%), 2-vinylnaphthalene (30.8 mg, 0.200 mmol, 2.00 equiv.), K_2CO_3 (34.6 mg, 0.250 mmol, 2.50 equiv.), and alkenyl thianthrenium salt **11-TT** (51.7 mg, 0.100 mmol, 1.00 equiv.). The vial was transferred into a N_2-filled glovebox. After addition of THF (1.0 mL, c = 0.10 M), the vial was capped, transferred out of the glovebox, and placed on a stirring plate. After being stirred at 25 °C for 23 h, the reaction mixture was diluted with CH_2Cl_2 (2 mL), and the resulting mixture was filtered through a short pad of Celite® using CH_2Cl_2 (5 mL) as eluent. The filtrate was collected and concentrated by rotary evaporation. The residue was purified by chromatography on silica gel eluting with hexanes/ethyl acetate (40:1 to 10:1, v/v) to afford **36** (26.4 mg, 0.072 mmol, 72%) as a colorless solid.

R*f* = 0.25 (hexanes/ethylacetate, 10:1, v/v).

NMR Spectroscopy:

^{1}H NMR (500 MHz, CD_2Cl_2, 298 K, δ): 7.84 – 7.81 (m, 2H), 7.78 (t, J = 8.5 Hz, 3H), 7.72 – 7.69 (m, 3H), 7.59 (dd, J = 8.7, 1.7 Hz, 1H), 7.47 – 7.40 (m, 2H), 6.86 (dd, J = 15.7, 10.4 Hz, 1H), 6.60 (d, J = 15.6 Hz, 1H), 6.31 (dd, J = 15.2, 10.4 Hz, 1H), 5.89 (dt, J = 14.6, 7.0 Hz, 1H), 3.71 (t, J = 7.2 Hz, 2H), 2.24 (q, J = 7.3 Hz, 2H), 1.84 (p, J = 7.3 Hz, 2H).

^{13}C NMR {^{1}H} (126 MHz, $CDCl_3$, 298 K, δ): 168.6, 135.2, 134.2, 134.1, 133.9, 133.0, 132.3, 131.6, 130.8, 129.6, 128.3, 128.1, 127.8, 126.4, 126.2, 125.9, 123.6, 123.4, 37.8, 30.4, 28.1.

HRMS-ESI (m/z) calc'd. for $C_{25}H_{22}NO_2$ $[M+H]^+$, 368.16450; found, 368.16436; deviation: +0.39 ppm.

Chloro-pent-4-en-1-yl-phthalimide (37)

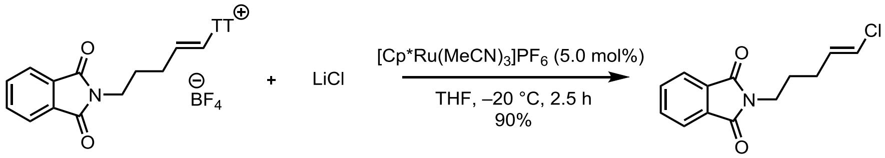

11-TT (1.5 equiv.) **37**

To a 4-mL borosilicate vial equipped with a stir bar was added LiCl (12.7 mg, 0.300 mmol, 1.50 equiv.) and alkenyl thianthrenium salt **11-TT** (104 mg, 0.200 mmol, 1.00 equiv.). The vial was transferred into a N_2-filled glovebox. After addition of [Cp*Ru(MeCN)$_3$]PF$_6$ (5.0 mg, 10 μmol, 5.0 mol%) and THF (2.0 mL, c = 0.10 M, THF was precooled to –20 °C), the vial was capped and placed in a cooling well (temperature at round –20 °C) which was pre-cooled in a dry ice-acetonitrile cooling bath. After being stirred at –20 °C for 2.5 h, the vial was transferred out of the glovebox, and the reaction mixture was concentrated by rotary evaporation. The residue was purified by chromatography on silica gel eluting with hexanes/ethyl acetate (40:1 to 10:1, v/v) to afford **37** (45.6 mg, 0.181 mmol, 90%) as a colorless solid.

Gram scale reaction

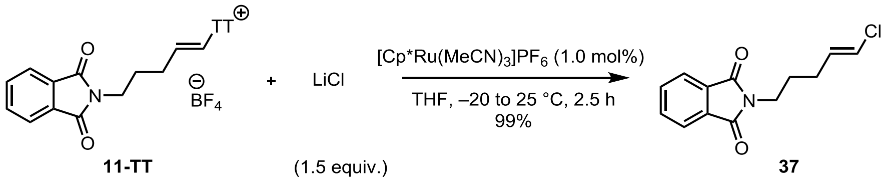

To a 100-mL round-bottomed flask equipped with a stir bar was added LiCl (127 mg, 3.00 mmol, 1.50 equiv.) and alkenyl thianthrenium salt **11-TT** (1.04 g, 2.00 mmol, 1.00 equiv.). The flask was transferred into a N_2-filled glovebox. After addition of [Cp*Ru(MeCN)$_3$]PF$_6$ (11 mg, 5.0 μmol, 1.0 mol%) and THF (20 mL, c = 0.10 M, THF was precooled to –20 °C), the flask was capped with a rubber septum and placed in a cooling well (temperature at round –20 °C) which was pre-cooled in a dry ice-acetonitrile cooling bath. After being stirred at –20 °C for 2 h, the flask was transferred out of the glovebox, and the reaction mixture was stirred at 25 °C for 30 min further and subsequently concentrated by rotary evaporation. The residue was purified by chromatography on silica gel eluting with hexanes/ethyl acetate (60:1 to 10:1, v/v) to afford **37** (495 mg, 1.99 mmol, 99%) as a colorless solid.

R*f* = 0.32 (hexanes/ethylacetate, 10:1, v/v).

NMR Spectroscopy:

^{1}H NMR (500 MHz, $CDCl_3$, 298 K, δ): 7.84 (dd, *J* = 5.4, 3.1 Hz, 2H), 7.71 (dd, *J* = 5.5, 3.0 Hz, 2H), 6.00 (dt, *J* = 13.2, 1.4 Hz, 1H), 5.89 (dt, *J* = 13.5, 7.2 Hz, 1H), 3.69 (t, *J* = 7.1 Hz, 2H), 2.12 (qd, *J* = 7.3, 1.4 Hz, 2H), 1.78 (p, *J* = 7.3 Hz, 2H).

^{13}C NMR {^{1}H} (126 MHz, $CDCl_3$, 298 K, δ): 168.6, 134.2, 132.5, 132.3, 123.8, 118.2, 37.5, 28.5, 27.9.

HRMS-ESI (m/z) calc'd. for $C_{13}H_{13}NO_2Cl$ $[M+H]^+$, 250.06293; found, 250.06264; deviation: +1.17 ppm.

Bromo-pent-4-en-1-yl-phthalimide (38)

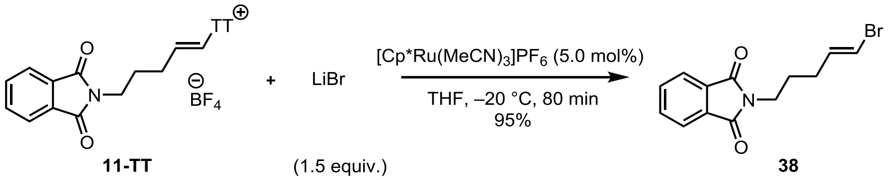

To a 4-mL borosilicate vial equipped with a stir bar was added LiBr (26.1 mg, 0.300 mmol, 1.50 equiv.) and alkenyl thianthrenium salt **11-TT** (104 mg, 0.200 mmol, 1.00 equiv.). The vial was transferred into a N_2-filled glovebox. After addition of $[Cp^*Ru(MeCN)_3]PF_6$ (5.0 mg, 10 µmol, 5.0 mol%) and THF (2.0 mL, c = 0.10 M, THF was precooled to –20 °C), the vial was capped and placed in a cooling well (temperature at round –20 °C) which was pre-cooled in a dry ice-acetonitrile cooling bath. After being stirred at –20 °C for 80 min, the vial was transferred out of the glovebox, and the reaction mixture was concentrated by rotary evaporation. The residue was purified by chromatography on silica gel eluting with hexanes/ethyl acetate (40:1 to 10:1, v/v) to afford **38** (55.7 mg, 0.190 mmol, 95%) as a colorless solid.

R*f* = 0.32 (hexanes/ethylacetate, 10:1, v/v).

NMR Spectroscopy:

^{1}H NMR (500 MHz, $CDCl_3$, 298 K, δ): 7.84 (dd, *J* = 5.4, 3.0 Hz, 2H), 7.71 (dd, *J* = 5.5, 3.0 Hz, 2H), 6.17 (dt, *J* = 13.8, 6.9 Hz, 1H), 6.09 (dd, *J* = 13.5, 1.1 Hz, 1H), 3.70 (t, *J* = 7.0 Hz, 2H), 2.14 – 2.07 (m, 2H), 1.80 (p, *J* = 7.2 Hz, 2H).

^{13}C NMR {^{1}H} (126 MHz, $CDCl_3$, 298 K, δ): 168.1, 136.2, 133.7, 131.8, 123.0, 105.1, 36.9, 30.0, 27.1.

HRMS-ESI (m/z) calc'd. for $C_{13}H_{13}NO_2Br$ $[M+H]^+$, 294.01242; found, 294.01217; deviation: +0.88 ppm.

Trifluoromethylsulfuryl-pent-4-en-1-yl-phthalimide (39)

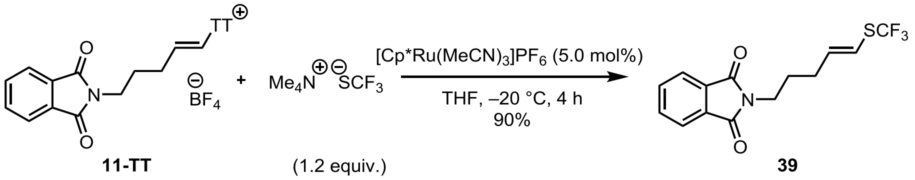

To a 4-mL borosilicate vial equipped with a stir bar was added alkenyl thianthrenium salt **11-TT** (51.7 mg, 0.100 mmol, 1.00 equiv.). The vial was transferred into a N_2-filled glovebox. After addition of tetramethyl ammonium trifluoromethylsulfide[3] (21.0 mg, 0.120 mmol, 1.20 equiv.), $[Cp^*Ru(MeCN)_3]PF_6$ (2.5 mg, 5.0 µmol, 5.0 mol%), and THF (1.0 mL, c = 0.10 M, THF was precooled to –20 °C), the vial was capped and placed in a cooling well (temperature at round –20 °C) which was pre-cooled in a dry ice-acetonitrile cooling bath. After being stirred at –20 °C for 4 h, the vial was transferred out of the glovebox, and the reaction mixture was concentrated by rotary evaporation. The residue was purified by chromatography on silica gel eluting with hexanes/ethyl acetate (40:1 to 10:1, v/v) to afford **39**(28.3 mg, 89.8 µmol, 90%) as a colorless solid.

R*f* = 0.32 (hexanes/ethylacetate, 10:1, v/v).

NMR Spectroscopy:

^{1}H NMR (500 MHz, $CDCl_3$, 298 K, δ): 7.85 (dd, *J* = 5.4, 3.1 Hz, 2H), 7.72 (dd, *J* = 5.4, 3.1 Hz, 2H), 6.23 (dt, *J* = 13.9, 6.8 Hz, 1H), 6.13 (d, *J* = 14.9 Hz, 1H), 3.71 (t, *J* = 7.1 Hz, 2H), 2.25 (q, *J* = 7.2 Hz, 2H), 1.83 (p, *J* = 7.3 Hz, 2H).

^{13}C NMR {^{1}H} (126 MHz, $CDCl_3$, 298 K, δ): 168.3, 144.1, 134.0, 132.0, 129.6 (q, *J* = 307.4 Hz), 123.3, 112.9 (q, *J* = 2.9 Hz), 37.2, 30.4, 27.3.

^{19}F NMR (471 MHz, CD_2Cl_2, 298 K, δ): –43.16 (s).

HRMS-ESI (m/z) calc'd. for $C_{14}H_{12}F_3NO_2SNa$ $[M+Na]^+$, 338.04330; found, 338.04296; deviation: +1.02 ppm.

Phenylacetylenyl-4-octene (40)

For tri-substituted thianthrenium salt **2-TT**

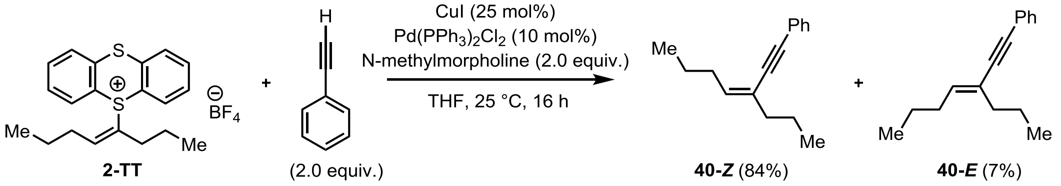

To a 4-mL borosilicate vial equipped with a stir bar was added $Pd(PPh_3)_2Cl_2$ (14 mg, 20 μmol, 10 mol%), CuI (9.6 mg, 50 μmol, 25 mol%), and alkenyl thianthrenium salt **2-TT** (82.8 mg, 0.200 mmol, 1.00 equiv.). The vial was transferred into a N_2-filled glovebox. After addition of THF (2.0 mL, c = 0.10 M), phenylacetylene (43.9 μL, 40.9 mg, 0.400mmol, 2.00 equiv.), and N-methylmorpholine (44.0 μL, 40.5 mg, 0.400 mmol, 2.00 equiv.), the vial was capped, transferred out of the glovebox, and placed on a stirring plate. After being stirred at 25 °C for 16 h, the reaction mixture was diluted with CH_2Cl_2 (2 mL), and the resulting mixture was filtered through a short pad of Celite® using CH_2Cl_2 (5 mL) as eluent. The filtrate was collected and concentrated by rotary evaporation. The residue was purified by chromatography on silica gel eluting with pentane to afford **40-*Z*** (35.5 mg, 0.167 mmol, 84%) as a colorless oil and **40-*E*** (3.0 mg, 14 μmol, 7%) as a colorless oil.

For tri-substituted thianthrenium salt **3-TT**

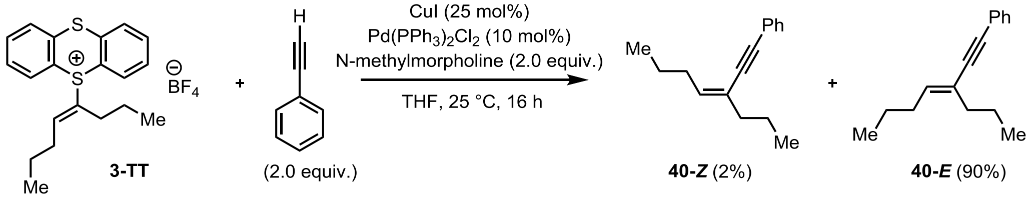

To a 4-mL borosilicate vial equipped with a stir bar was added $Pd(PPh_3)_2Cl_2$ (14 mg, 20 μmol, 10 mol%), CuI (9.6 mg, 50 μmol, 25 mol%), and alkenyl thianthrenium salt **3-TT** (82.8 mg, 0.200 mmol, 1.00 equiv.). The vial was transferred into a N_2-filled glovebox. After addition of THF (2.0 mL, c = 0.10 M), phenylacetylene (43.9 μL, 40.9 mg, 0.400mmol, 2.00 equiv.), and N-methylmorpholine (44.0 μL, 40.5 mg, 0.400 mmol, 2.00 equiv.), the

vial was capped, transferred out of the glovebox, and placed on a stirring plate. After being stirred at 25 °C for 16 h, the reaction mixture was diluted with CH_2Cl_2 (2 mL), and the resulting mixture was filtered through a short pad of Celite® using CH_2Cl_2 (5 mL) as eluent. The filtrate was collected and concentrated by rotary evaporation. The residue was purified by chromatography on silica gel eluting with pentane to afford **40-*Z*** (0.7 mg, 3.3 μmol, 2%) as a colorless oil and **40-*E*** (38.0 mg, 0.179 mmol, 90%) as a colorless oil.

Sonogashira couplings with alkenyl thianthrenium salts are as stereoselective as coupling reactions with alkenyl bromides.[4]

40-*Z*

R*f* = 0.76 (hexanes).

NMR Spectroscopy:

^{1}H NMR (500 MHz, $CDCl_3$, 298 K, δ): 7.37 – 7.31 (m, 2H), 7.25 – 7.14 (m, 3H), 5.90 (tt, *J* = 7.5, 1.1 Hz, 1H), 2.12 (t, *J* = 7.4, 1.0 Hz, 2H), 2.05 (q, *J* = 7.4 Hz, 2H), 1.59 – 1.47 (m, 2H), 1.41 – 1.29 (m, 2H), 0.88 (t, *J* = 7.4 Hz, 3H), 0.86 (t, *J* = 7.4 Hz, 3H).

^{13}C NMR {^{1}H} (126 MHz, $CDCl_3$, 298 K, δ): 138.5, 131.4, 128.2, 127.6, 123.9, 123.1, 91.9, 86.5, 32.7, 30.5, 22.6, 21.7, 13.9, 13.8.

GCMS-EI (m/z) calc'd. for $C_{16}H_{20}$ [M], 212.15595; found, 212.15579; deviation: +0.76 ppm.

40-*E*

R*f* = 0.70 (hexanes).

NMR Spectroscopy:

^{1}H NMR (500 MHz, $CDCl_3$, 298 K, δ): 7.49 – 7.41 (m, 2H), 7.32 (tdt, *J* = 7.0, 5.4, 1.7 Hz, 3H), 5.75 (tt, *J* = 7.4, 1.3 Hz, 1H), 2.35 (q, *J* = 7.3 Hz, 2H), 2.23 – 2.15 (m, 2H), 1.68 – 1.57 (m, 2H), 1.53 – 1.43 (m, 2H), 0.97 (t, *J* = 7.4 Hz, 3H), 0.95 (t, *J* = 7.4 Hz,3H).

^{13}C NMR {^{1}H} (126 MHz, $CDCl_3$, 298 K, δ): 138.0, 131.4, 128.3, 127.8, 124.0, 123.1, 93.2, 88.5, 39.2, 32.7, 22.5, 21.8, 13.9, 13.5.

GCMS-EI (m/z) calc'd. for $C_{16}H_{20}$ [M], 212.15595; found, 212.15577; deviation: +0.85 ppm.

Synthesis of alkene starting materials

Nicotinic acid-derived alkene 26

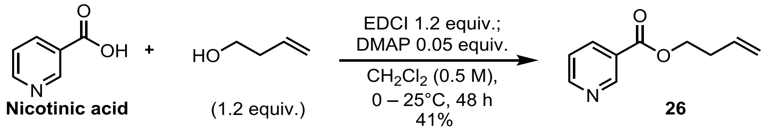

Under ambient atmosphere, a 25 mL round-bottom flask equipped with a magnetic stir bar was charged with 3-buten-1-ol (516 μL, 433 mg, 6.00 mmol, 1.20 equiv.), EDCI•HCl (1.15 g, 6.00 mmol, 1.20 equiv.),

triethylamine(1.05 mL, 759 mg, 7.50 mmol, 1.50 equiv.), and DMAP (61 mg, 0.50 mmol, 0.10 equiv.) in anhydrous CH_2Cl_2 (10 mL, 0.50 M). After the reaction mixture was cooled to 0 °C, Nicotinic acid (615 mg, 5.00 mmol, 1.00 equiv.) was added at 0 °C in 5 portions within 2 min. The reaction mixture was stirred at 0 °C for 30 min and then at 25 °C for 48 h further. The resulting mixture was subsequently diluted with 20 mL CH_2Cl_2, washed by 1 M HCl (2 × ca. 20 mL), saturated aqueous $NaHCO_3$ (2 × ca. 20 mL), and brine (1 × ca. 20 mL). The organic layer was dried over Na_2SO_4, filtered, and the solvent was removed under reduced pressure. The residue was further dried in vacuo to afford **26** (362 mg, 2.04 mmol, 41 %) as a colorless oil.

R*f* = 0.25 (hexanes/ethyl acetate, 10:1, v/v).

NMR Spectroscopy:

^{1}H NMR (500 MHz, $CDCl_3$, 298 K, δ): 9.21 (d, *J* = 2.3 Hz, 1H), 8.76 (dt, *J* = 5.0, 2.0 Hz, 1H), 8.27 (dt, *J* = 7.9, 2.0 Hz, 1H), 7.40 – 7.35 (m, 1H), 5.84 (ddt, *J* = 19.4, 10.3, 6.8 Hz, 1H), 5.16 (dq, *J* = 17.1, 1.7 Hz, 1H), 5.10 (dp, *J* = 10.2, 1.3 Hz, 1H), 4.40 (td, *J* = 6.7, 2.4 Hz, 2H), 2.52 (qt, *J* = 6.7, 1.4 Hz, 2H).

^{13}C NMR {^{1}H} (126 MHz, $CDCl_3$, 298 K, δ): 164.9, 153.1, 150.6, 136.7, 133.4, 125.9, 123.0, 117.3, 64.1, 32.8.

HRMS-ESI (m/z) calc'd. for $C_{10}H_{12}NO_2^+$ $[M+H]^+$, 178.08625; found, 178.08609; deviation: +0.92 ppm.

Cinchophene-derived alkene 27

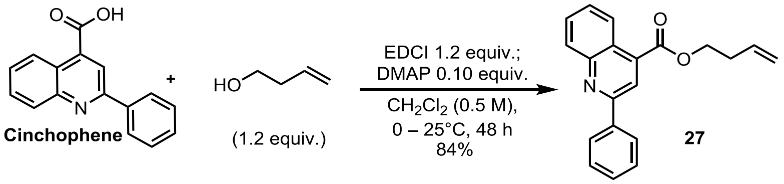

Under ambient atmosphere, a 25 mL round-bottom flask equipped with a magnetic stir bar was charged with 3-buten-1-ol (310 µL, 260 mg, 3.60 mmol, 1.20 equiv.), EDCI•HCl (690 mg, 3.60 mmol, 1.20 equiv.), triethylamine(630 µL, 455 mg, 4.50 mmol, 1.50 equiv.), and DMAP (37 mg, 0.30 mmol, 0.10 equiv.) in anhydrous CH_2Cl_2 (10 ml, 0.30 M). After the reaction mixture was cooled to 0 °C, Cinchophene (748 mg, 3.00 mmol, 1.00 equiv.) was added at 0 °C in 5 portions within 2 min. The reaction mixture was stirred at 0 °C for 30 min and then at 25 °C for 48 h further. The resulting mixture was subsequently diluted with 20 mL CH_2Cl_2, washed by 1 M HCl (2 × ca. 20 mL), saturated aqueous $NaHCO_3$ (2 × ca. 20 mL), and brine (1 × ca. 20 mL). The organic layer was dried over Na_2SO_4, filtered, and the solvent was removed under reduced pressure. The residue was purified by chromatography on silica gel eluting with hexanes/ethyl acetate (20:1, v/v). The product-containing fractions were collected and concentrated under reduced pressure. The residue was further dried in vacuo to afford **26** (362.0 mg, 2.04 mmol, 84 %) as a yellow oil.

R*f* = 0.45 (hexanes/ethyl acetate, 10:1, v/v).

NMR Spectroscopy:

^{1}H NMR (500 MHz, $CDCl_3$, 298 K, δ): 8.74 (dd, *J* = 8.6, 1.4 Hz, 1H), 8.40 (s, 1H), 8.26 – 8.18 (m, 3H), 7.77 (ddd, *J* = 8.4, 6.8, 1.4 Hz, 1H), 7.62 (ddd, *J* = 8.3, 6.8, 1.3 Hz, 1H), 7.56 (dd, *J* = 8.3, 6.8 Hz, 2H), 7.52 – 7.47 (m, 1H), 5.94 (ddt, *J* = 17.1, 10.3, 6.7 Hz, 1H), 5.25 (dq, *J* = 17.1, 1.6 Hz, 1H), 5.19 (dq, *J* = 10.2, 1.4 Hz, 1H), 4.55 (t, *J* = 6.6 Hz, 2H), 2.64 (qt, *J* = 6.7, 1.4 Hz, 2H).

^{13}C NMR {^{1}H} (126 MHz, $CDCl_3$, 298 K, δ): 166.4, 156.7, 149.2, 138.8, 135.9, 133.8, 130.3, 129.9, 129.7, 128.9, 127.7, 127.4, 125.4, 124.0, 120.2, 117.8, 64.8, 33.2.

HRMS-ESI (m/z) calc'd. for $C_{20}H_{18}NO_2^+$ $[M+H]^+$, 304.13320; found, 304.13268; deviation: +1.72 ppm.

Lithocholic acid-derived alkene 28

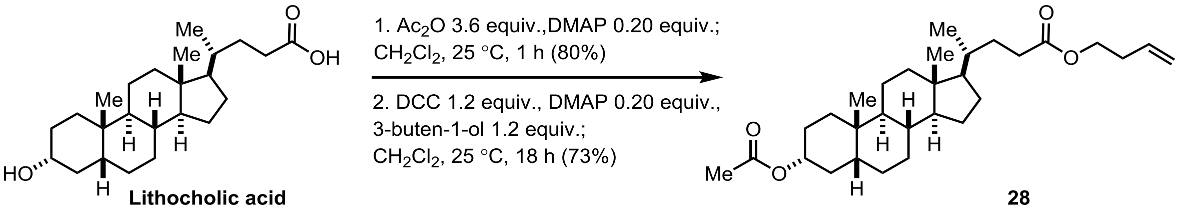

Under ambient atmosphere, a 50 mL round-bottom flask equipped with a magnetic stir bar was charged with Lithocholic acid (1.00 g, 2.66 mmol, 1.00 equiv.), and DMAP (65 mg, 0.53 mmol, 0.20 equiv.) in anhydrous CH_2Cl_2 (30 mL, 0.10 M). After the reaction mixture was cooled to 0 °C, acetic anhydride (907 µL, 980 mg, 9.58 mmol, 3.60 equiv.) was added dropwise at 0 °C over a period of 2 min. The reaction mixture was stirred at 0 °C for 5 min and then at 25 °C for 1 h further. The resulting mixture was subsequently diluted with 20 mL CH_2Cl_2, washed by 1 M HCl (2 × ca. 20 mL), saturated aqueous $NaHCO_3$ (2 × ca. 20 mL), and brine (1 × ca. 20 mL). The organic layer was dried over Na_2SO_4, filtered, and the solvent was removed under reduced pressure. The residue was purified by chromatography on silica gel eluting with hexanes/ethyl acetate (5:1, v/v). The acylated product was collected and dried in vacuo to afford **28-OAc** (886.6 mg, 2.12 mmol, 80%) as a colorless solid.

R*f* = 0.52 (hexanes/ethyl acetate, 5:1, v/v).

Under ambient atmosphere, a 25 mL round-bottom flask equipped with a magnetic stir bar was charged with acylated Lithocholic acid **28-OAc** (418 mg, 1.00 mmol, 1.00 equiv.), 3-buten-1-ol (103 µL, 86.7 mg, 1.20 mmol, 1.20 equiv.), DCC (248 mg, 1.20 mmol, 1.20 equiv.), and DMAP (24 mg, 0.20 mmol, 0.20 equiv.) in anhydrous CH_2Cl_2 (10 mL, 0.10 M). The reaction mixture was stirred at 25 °C for 18 h. The resulting mixture was subsequently diluted with 20 mL CH_2Cl_2, washed by 1 M HCl (2 × ca. 20 mL), saturated aqueous $NaHCO_3$ (2 × ca. 20 mL), and brine (1 × ca. 20 mL). The organic layer was dried over Na_2SO_4, filtered, and the solvent was removed under reduced pressure. The residue was purified by chromatography on silica gel eluting with hexanes/ethyl acetate (20:1, v/v). The product-containing fractions were collected and concentrated under reduced pressure. The residue was further dried in vacuo to afford **28** (343.4 mg, 0.727 mmol, 73 %) as a colorless solid.

R*f* = 0.45 (hexanes/ethyl acetate, 10:1, v/v).

NMR Spectroscopy:

^{1}H NMR (500 MHz, $CDCl_3$, 298 K, δ): 5.78 (ddt, *J* = 17.0, 10.2, 6.7 Hz, 1H), 5.10 (dt, *J* = 17.2, 1.7 Hz, 1H), 5.06 (dd, *J* = 10.3, 1.6 Hz, 1H), 4.71 (tt, *J* = 11.4, 4.7 Hz, 1H), 4.11 (t, *J* = 6.8 Hz, 2H), 2.37 (qt, *J* = 6.4, 1.3 Hz, 2H), 2.32 (dt, *J* = 10.0, 5.2 Hz, 1H), 2.20 (ddd, *J* = 15.7, 9.7, 6.6 Hz, 1H), 2.02 (s, 3H), 1.95 (dt, *J* = 12.4, 3.1 Hz, 1H), 1.88 – 1.74 (m, 5H), 1.72 – 1.63 (m, 1H), 1.60 – 1.50 (m, 2H), 1.47 – 1.34 (m, 7H), 1.33 – 1.19 (m, 4H), 1.18 – 0.98 (m, 6H), 0.91 (s, 3H), 0.90 (d, *J* = 6.5 Hz, 3H), 0.63 (s, 3H).

^{13}C NMR {^{1}H} (126 MHz, $CDCl_3$, 298 K, δ): 174.5, 170.8, 134.3, 117.4, 74.6, 63.5, 56.7, 56.3, 43.0, 42.1, 40.6, 40.4, 36.0, 35.6, 35.3, 34.8, 33.3, 32.5, 31.5, 31.2, 28.4, 27.3, 26.9, 26.6, 24.4, 23.6, 21.7, 21.1, 18.5, 12.3.

HRMS-ESI (m/z) calc'd. for $C_{30}H_{48}O_4Na^+$ $[M+Na]^+$, 495.34447; found, 495.34362; deviation: +1.73 ppm.

Epiandrosterone-derived alkene 29

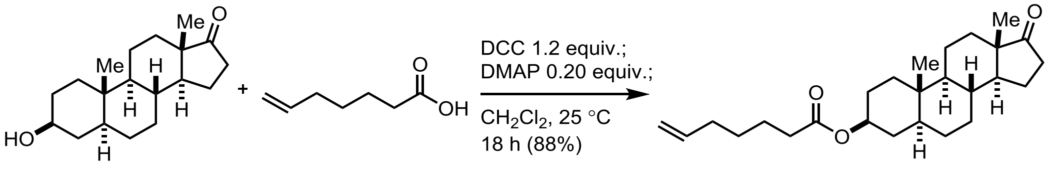

Under ambient atmosphere, a 25 mL round-bottom flask equipped with a magnetic stir bar was charged with Epiandrosteron (580 mg, 2.00 mmol, 1.00 equiv.), 6-heptenoic acid (325 µL, 308 mg, 2.40 mmol, 1.20 equiv.), DCC (495 mg, 2.40 mmol, 1.20 equiv.), and DMAP (48 mg, 0.40 mmol, 0.20 equiv.) in anhydrous CH_2Cl_2 (10 mL, 0.20 M). The reaction mixture was stirred at 25 °C for 18 h. The resulting mixture was subsequently diluted with 20 mL CH_2Cl_2, washed by 1 M HCl (2 × ca. 20 mL), saturated aqueous $NaHCO_3$ (2 × ca. 20 mL), and brine (1 × ca. 20 mL). The organic layer was dried over Na_2SO_4, filtered, and the solvent was removed under reduced pressure. The residue was purified by chromatography on silica gel eluting with hexanes/ethyl acetate (20:1, v/v). The product-containing fractions were collected and concentrated under reduced pressure. The residue was further dried in vacuo to afford **29** (400.3 mg, 1.76 mmol, 88 %) as a colorless solid.

R*f* = 0.32 (hexanes/ethyl acetate, 10:1, v/v).

NMR Spectroscopy:

^{1}H NMR (500 MHz, $CDCl_3$, 298 K, δ): 5.84 – 5.73 (m, 1H), 5.00 (dq, *J* = 17.2, 1.8 Hz, 1H), 4.94 (dt, *J* = 10.3, 1.9 Hz, 1H), 4.69 (tdd, *J* = 11.5, 5.7, 4.0 Hz, 1H), 2.42 (dd, *J* = 19.3, 8.9 Hz, 1H), 2.26 (td, *J* = 7.4, 1.7 Hz, 2H), 2.05 (ddd, *J* = 14.5, 9.0, 6.9 Hz, 3H), 1.92 (ddd, *J* = 14.1, 8.5, 5.9 Hz, 1H), 1.77 (m, 4H), 1.62 (m, 4H), 1.50 (m, 3H), 1.45 – 1.36 (m, 2H), 1.36 – 1.16 (m, 7H), 1.07 – 0.93 (m, 2H), 0.85 (d, *J* = 2.5 Hz, 6H), 0.71 (ddd, *J* = 12.5, 10.3, 3.9 Hz, 1H).

^{13}C NMR {^{1}H} (126 MHz, $CDCl_3$, 298 K, δ): 221.4, 173.5, 138.7, 114.9, 73.5, 54.5, 51.6, 48.0, 44.9, 36.9, 36.1, 35.9, 35.3, 34.8, 34.2, 33.6, 31.8, 31.0, 28.6, 28.5, 27.7, 24.7, 22.0, 20.7, 14.0, 12.4.

HRMS-ESI (m/z) calc'd. for $C_{26}H_{40}O_3Na^+$ $[M+Na]^+$, 423.28696; found, 423.28656; deviation: +0.95 ppm.

Piperidine-derived alkene 30

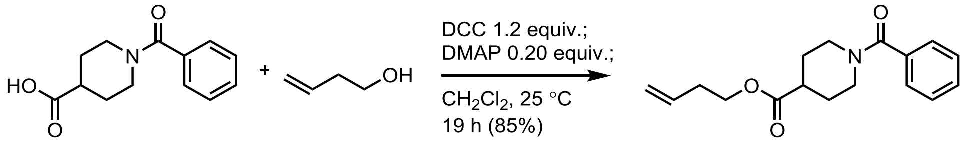

30

Under ambient atmosphere, a 25 mL round-bottom flask equipped with a magnetic stir bar was charged with 1-benzoylpiperidine-4-carboxylic acid (466 mg, 2.00 mmol, 1.00 equiv.), 3-buten-1-ol (103 μL, 86.7 mg, 1.20 mmol, 1.20 equiv.), DCC (495 mg, 2.40 mmol, 1.20 equiv.), and DMAP (48 mg, 0.40 mmol, 0.20 equiv.) in anhydrous CH_2Cl_2 (20 mL, 0.10 M). The reaction mixture was stirred at 25 °C for 19 h. The resulting mixture was subsequently diluted with 20 mL CH_2Cl_2, washed by 1 M HCl (2 × ca. 20 mL), saturated aqueous $NaHCO_3$ (2 × ca. 20 mL), and brine (1 × ca. 20 mL). The organic layer was dried over Na_2SO_4, filtered, and the solvent was removed under reduced pressure. The residue was purified by chromatography on silica gel eluting with hexanes/ethyl acetate (8:1, v/v). The product-containing fractions were collected and concentrated under reduced pressure. The residue was further dried in vacuo to afford **30** (489.0 mg, 1.70 mmol, 85 %) as a colorless liquid.

R*f* = 0.30 (hexanes/ethyl acetate, 5:1, v/v).

NMR Spectroscopy:

^{1}H NMR (600 MHz, DMSO-d_6, 298 K, δ): 7.45 – 7.41 (m, 3H), 7.39 – 7.34 (m, 2H), 5.83 – 5.67 (m, 1H), 5.12 – 5.08 (m, 1H), 5.06 – 5.03 (m, 1H), 4.32 (bs, 1H), 3.53 (bs, 1H), 3.34 (s, 1H), 3.08 (bs, 1H), 2.96 (bs, 1H), 2.63 (tt, *J* = 10.9, 4.0 Hz, 1H), 2.51 – 2.47 (m, 1H), 2.33 (dtt, *J* = 6.6, 5.4, 1.3 Hz, 2H), 1.91 (bs, 1H), 1.77 (bs, 1H), 1.51 (bs, 2H).

^{13}C NMR {^{1}H} (151 MHz, DMSO-d_6, 298 K, δ): 173.1, 168.5, 135.7, 133.9, 128.8, 127.8, 126.1, 116.6, 62.3, 45.7 (bs), 40.1 (bs), 39.5, 32.1, 27.6 (bs), 27.0 (bs).

HRMS-ESI (m/z) calc'd. for $C_{17}H_{21}NO_3Na^+$ $[M+Na]^+$, 310.14136; found, 310.14128; deviation: +0.27 ppm.

Fluazinam-derived alkene 31

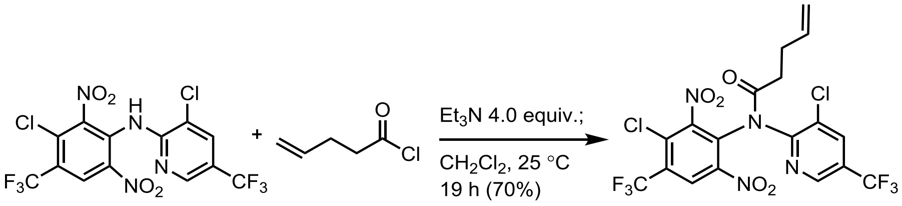

Fluazinam **31**

Under ambient atmosphere, a 25 mL round-bottom flask equipped with a magnetic stir bar was charged with Fluazinam (465 mg, 1.00 mmol, 1.00 equiv.) in anhydrous CH_2Cl_2 (10 mL, 0.10 M). After the reaction mixture was stirred at 0 °C for 5 min, triethylamine (557 μL, 405 mg, 4.00 mmol, 4.00 equiv.) was added, followed by

dropwise addition of 4-pentenoyl chloride (226 µL, 237 mg, 2.00 mmol, 2.00 equiv.). The reaction mixture was continuously stirred 0 °C for 30 min followed by at 25 °C for 18 h. The resulting mixture was subsequently diluted with 20 mL CH_2Cl_2, washed by 1 M HCl (2 × ca. 20 mL), saturated aqueous $NaHCO_3$ (2 × ca. 20 mL), and brine (1 × ca. 20 mL). The organic layer was dried over Na_2SO_4, filtered, and the solvent was removed under reduced pressure. The residue was purified by chromatography on silica gel eluting with hexanes/ethyl acetate (20:1, v/v). The product-containing fractions were collected and concentrated under reduced pressure. The residue was further dried in vacuo to afford **31** (380.1 mg, 696 µmol, 70 %) as a yellow solid.

R*f* = 0.52 (hexanes/ethyl acetate, 20:1, v/v).

NMR Spectroscopy:

^{1}H NMR (500 MHz, $CDCl_3$, 298 K, δ): 8.62 (s, 1H), 8.45 (s, 1H), 8.05 (s, 1H), 5.88 – 5.71 (m, 1H), 5.09 (d, *J* = 17.2 Hz, 1H), 5.02 (d, *J* = 10.2 Hz, 1H), 2.67 – 2.36 (m, 4H).

^{13}C NMR {^{1}H} (126 MHz, $CDCl_3$, 298 K, δ): 173.5, 151.4, 151.1, 147.0 (bs), 143.6, 143.5, 137.0, 136.1, 132.6 – 131.7 (m), 130.9 (bs), 129.9, 129.6 (bs), 126.9 (q, *J* = 34.2 Hz), 125.0, 124.9, 122.1 (q, *J* = 273.1 Hz), 120.6 (q, *J* = 275.4 Hz), 116.2, 34.6, 28.3.

^{19}F NMR (471 MHz, CD_2Cl_2, 298 K, δ): –62.28 (s), –63.06 (s).

HRMS-ESI (m/z) calc'd. for $C_{18}H_{10}Cl_2F_6N_4O_5Na^+$ $[M+Na]^+$, 568.98247; found, 568.98249; deviation: –0.04 ppm.

Probenecid-derived alkene 32

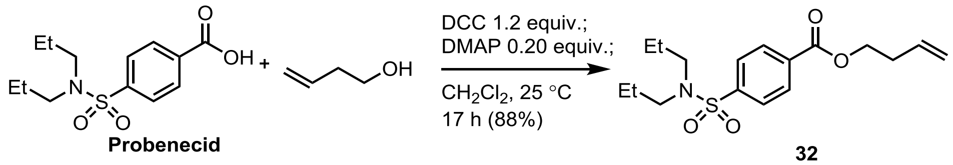

32

Under ambient atmosphere, a 25 mL round-bottom flask equipped with a magnetic stir bar was charged with Probenecid (571 mg, 2.00 mmol, 1.00 equiv.), 3-buten-1-ol (103 µL, 86.7 mg, 1.20 mmol, 1.20 equiv.), DCC (495 mg, 2.40 mmol, 1.20 equiv.), and DMAP (48 mg, 0.40 mmol, 0.20 equiv.) in anhydrous CH_2Cl_2 (20 mL, 0.10 M). The reaction mixture was stirred at 25 °C for 17 h. The resulting mixture was subsequently diluted with 20 mL CH_2Cl_2, washed by 1 M HCl (2 × ca. 20 mL), saturated aqueous $NaHCO_3$ (2 × ca. 20 mL), and brine (1 × ca. 20 mL). The organic layer was dried over Na_2SO_4, filtered, and the solvent was removed under reduced pressure. The residue was purified by chromatography on silica gel eluting with hexanes/ethyl acetate (9:1 to 6:1, v/v). The product-containing fractions were collected and concentrated under reduced pressure. The residue was further dried in vacuo to afford **32** (600.0 mg, 1.77 mmol, 88 %) as a colorless liquid.

R*f* = 0.70 (hexanes/ethyl acetate, 5:1, v/v).

NMR Spectroscopy:

^{1}H NMR (500 MHz, $CDCl_3$, 298 K, δ): 8.14 (d, *J* = 8.5 Hz, 2H), 7.86 (d, *J* = 8.5 Hz, 2H), 5.86 (ddt, *J* = 17.1, 10.3, 6.7 Hz, 1H), 5.18 (dq, *J* = 17.2, 1.6 Hz, 1H), 5.12 (dq, *J* = 10.3, 1.4 Hz, 1H), 4.40 (t, *J* = 6.7 Hz, 2H), 3.12 – 3.05 (m, 4H), 2.54 (qt, *J* = 6.7, 1.4 Hz, 2H), 1.59 – 1.48 (m, 4H), 0.86 (t, *J* = 7.4 Hz, 6H).

^{13}C NMR {^{1}H} (126 MHz, $CDCl_3$, 298 K, δ): 165.4, 144.4, 133.9, 133.8, 130.4, 127.2, 117.8, 64.8, 50.2, 33.3, 22.2, 11.4.

HRMS-ESI (m/z) calc'd. for $C_{17}H_{25}NO_4Na^+$ $[M+Na]^+$, 362.13965; found, 362.13965; deviation: 0.00 ppm.

Bicalutamide-derived alkene 33

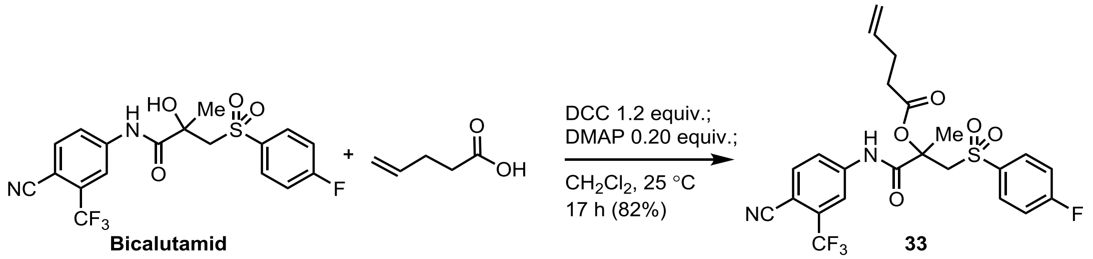

Under ambient atmosphere, a 25 mL round-bottom flask equipped with a magnetic stir bar was charged with Epiandrosteron (430 mg, 1.00 mmol, 1.00 equiv.), 6-pentenoic acid (112 µL, 110 mg, 1.20 mmol, 1.20 equiv.), DCC (248 mg, 1.20 mmol, 1.20 equiv.), and DMAP (24 mg, 0.20 mmol, 0.20 equiv.) in anhydrous CH_2Cl_2 (10 mL, 0.10 M). The reaction mixture was stirred at 25 °C for 17 h. The resulting mixture was subsequently diluted with 20 mL CH_2Cl_2, washed by 1 M HCl (2 × ca. 20 mL), saturated aqueous $NaHCO_3$ (2 × ca. 20 mL), and brine (1 × ca. 20 mL). The organic layer was dried over Na_2SO_4, filtered, and the solvent was removed under reduced pressure. The residue was purified by chromatography on silica gel eluting with hexanes/ethyl acetate (3:1, v/v). The product-containing fractions were collected and concentrated under reduced pressure. The residue was further dried in vacuo to afford **33** (418.0 mg, 816 µmol, 82 %) as a colorless solid.

R*f* = 0.81 (hexanes/ethyl acetate, 1:1, v/v).

NMR Spectroscopy:

^{1}H NMR (500 MHz, $CDCl_3$, 298 K, δ): 8.69 (s, 1H), 8.06 (d, *J* = 2.0 Hz, 1H), 7.93 (dd, *J* = 8.5, 2.1 Hz, 1H), 7.90 – 7.85 (m, 2H), 7.80 (d, *J* = 8.4 Hz, 1H), 7.19 (t, *J* = 8.5 Hz, 2H), 5.89 (ddt, *J* = 16.8, 10.3, 6.3 Hz, 1H), 5.13 (dd, *J* = 17.2, 1.6 Hz, 1H), 5.06 (dd, *J* = 10.2, 1.5 Hz, 1H), 4.27 (d, *J* = 14.2 Hz, 1H), 4.02 (d, *J* = 14.2 Hz, 1H), 2.73 – 2.59 (m, 2H), 2.49 – 2.43 (m, 2H), 1.86 (s, 3H).

^{13}C NMR {^{1}H} (126 MHz, $CDCl_3$, 298 K, δ): 171.3, 169.6, 166.0 (d, *J* = 257.6 Hz), 141.3, 136.4, 135.9 (d, *J* = 3.1 Hz), 135.8, 134.3 – 133.5 (m), 130.9 (d, *J* = 9.6 Hz), 122.9, 122.1 (q, *J* = 274.0 Hz), 118.2 (q, *J* = 4.9 Hz), 116.8 (d, *J* = 22.7 Hz), 116.0, 115.6, 104.9, 80.1, 58.2, 34.0, 28.3, 24.4.

^{19}F NMR (471 MHz, $CDCl_3$, 298 K, δ): –62.13, –102.51 (m).

HRMS-ESI (m/z) calc'd. for $C_{23}H_{20}N_2O_5SF_4Na^+$ $[M+Na]^+$, 535.09213; found, 535.09242; deviation: –0.55 ppm.

Synthesis cyclo-adduct: thianthrenium dication

trans-4-Octene-derived thianthrenium dication 2-INT

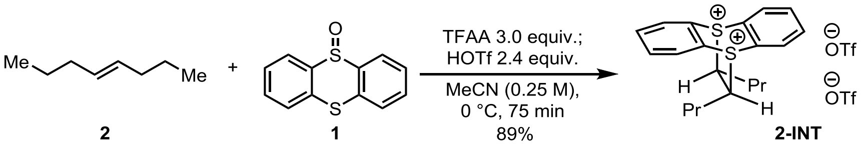

Under ambient atmosphere, a 20 mL borosilicate vial equipped with a magnetic stir bar was charged with trans-4-octene (78.5 µL, 56.1 mg, 0.500 mmol, 1.00 equiv.), thianthrene S-oxide (**1**) (120 mg, 0.517 mmol, 1.03 equiv.), and MeCN (2.0 mL, c = 0.25 M). After cooling to 0°C, trifluoroacetic anhydride (0.21 mL, 0.31 g, 1.5 mmol, 3.0 equiv.) was added dropwise within 30 seconds, followed by dropwise addition of HOTf (104 µL, 176 mg, 1.08 mmol, 2.4 equiv.) within 10 seconds. After stirring the lilac mixture at 0 °C for 75 min, the resulting light pink mixture was concentrated under reduced pressure and subsequently diluted with CH_2Cl_2 (2 mL). The CH_2Cl_2 solution was poured onto a stirring solution of Et_2O (ca. 20 mL), and colorless precipitates formed immediately. The colorless solid was collected on a filter frit and rinsed with Et_2O (3 × ca. 5 mL) The solid was collected and further dried in vacuo to afford **2-INT** (280.0 mg, 447 µmol, 89 %) as a colorless solid.

NMR Spectroscopy:

^{1}H NMR (500 MHz, CD_3CN, 298 K, δ): 8.65 – 8.60 (m, 4H), 8.20 – 8.12 (m, 4H), 4.32 – 4.25 (m, 2H), 1.81 – 1.72 (m, 2H), 1.71 – 1.49 (m, 6H), 0.93 (t, *J* = 7.1 Hz, 6H).

^{13}C NMR {^{1}H} (126 MHz, CD_3CN, 298 K, δ): 137.6 – 137.4 (m, 4C), 125.9, 125.0 – 120.5 (m), 122.8, 60.9, 35.0, 20.9, 13.6.

^{19}F NMR (471 MHz, CD_3CN, 298 K, δ): –79.27 (s).

HRMS-ESI (m/z) calc'd. for $C_{20}H_{24}S_2^{2+}$ $[M]^{2+}$, 164.06542; found, 164.06541; deviation: +0.08 ppm.

cis-4-Octene-derived thianthrenium dication 3-INT

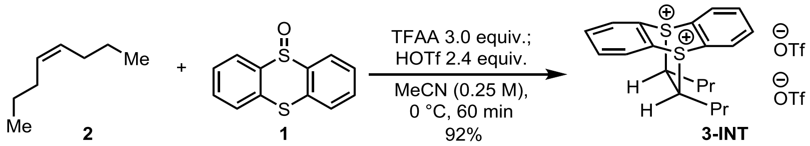

Under ambient atmosphere, a 20 mL borosilicate vial equipped with a magnetic stir bar was charged with cis-4-octene (78.5 µL, 56.1 mg, 0.500 mmol, 1.00 equiv.), thianthrene S-oxide (**1**) (120 mg, 0.517 mmol, 1.03 equiv.), and MeCN (2.0 mL, c = 0.25 M). After cooling to 0°C, trifluoroacetic anhydride (0.21 mL, 0.31 g, 1.5 mmol, 3.0 equiv.) was added dropwise within 30 seconds, followed by dropwise addition of HOTf (104 µL, 176 mg, 1.08 mmol, 2.4 equiv.) within 10 seconds. After stirring the lilac mixture at 0 °C for 60 min, the resulting yellow mixture was concentrated under reduced pressure and subsequently diluted with CH_2Cl_2 (2 mL). The CH_2Cl_2 solution was poured onto a stirring solution of Et_2O (ca. 20 mL), and colorless precipitates

formed immediately. The colorless solid was collected on a filter frit and rinsed with Et_2O (3 × ca. 5 mL) The solid was collected and further dried in vacuo to afford **3-INT** (287.2 mg, 459 µmol, 92 %) as a beige solid.

NMR Spectroscopy:

^{1}H NMR (500 MHz, CD_3CN, 298 K, δ): 8.66 (dd, *J* = 5.8, 3.4 Hz, 2H), 8.60 (dd, *J* = 5.9, 3.4 Hz, 2H), 8.19 (dd, *J* = 5.8, 3.3 Hz, 2H), 8.12 (dd, *J* = 5.9, 3.4 Hz, 2H), 4.81 – 4.68 (m, 2H), 1.98 – 1.90 (m, 2H, overlapped with CD_3CN), 1.89 – 1.77 (m, 2H), 1.71 – 1.60 (m, 2H), 1.43 – 1.23 (m, 2H), 0.97 (t, *J* = 7.2 Hz, 6H).

^{13}C NMR {^{1}H} (151 MHz, CD_3CN, 298 K, δ): 137.4, 137.3, 136.6, 136.5, 125.8, 123.3, 121.5 (q, *J* = 320.6 Hz), 59.2, 30.1, 21.1, 13.3.

^{19}F NMR (471 MHz, CD_3CN, 298 K, δ): –79.26 (s).

HRMS-ESI (m/z) calc'd. for $C_{20}H_{24}S_2^{2+}$ $[M]^{2+}$, 164.06542; found, 164.06525; deviation: +1.05 ppm.

Reactivity studies on cycloadducts: thianthrenium dications

Studies on the formation of 2-TT from trans-4-octene by NMR

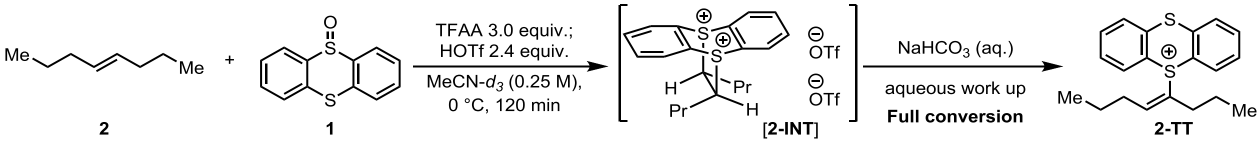

Under ambient atmosphere, a 4 mL borosilicate vial equipped with a magnetic stir bar was charged with trans-4-octene (78.5 µL, 56.1 mg, 0.500 mmol, 1.00 equiv.), thianthrene S-oxide (**1**) (60 mg, 0.26 mmol, 0.52 equiv.), and CD_3CN (1.0 mL, c = 0.50 M). After cooling to 0°C, trifluoroacetic anhydride (0.11 mL, 0.16 g, 0.75 mmol, 3.0 equiv.) was added dropwise within 30 seconds, an aliquot (0.20 mL) was taken out and diluted in 0.5 mL CD_3CN, and ^{1}H-NMR was measured for this sample. HOTf (52 µL, 88 mg, 0.59 mmol, 2.4 equiv.) was added dropwise within 10 seconds, during the course of stirring the lilac mixture at 0 °C for 120 min, an aliquot (0.20 mL) was taken out and diluted in 0.5 mL CD_3CN, and ^{1}H-NMR was measured for the samples at the time of 30", 2'30", 4'30", 7', 10', 15', 20', 30', 40', 60', 90', and 120'. The resulting light pink mixture was concentrated under reduced pressure and ^{1}H-NMR was checked for the residue. Subsequently the residue was diluted with CD_3Cl (2 mL). A saturated aqueous $NaHCO_3$ solution (ca. 5.0 mL) was slowly added into the CD_3Cl solution, the resulting organic phase was separated and measure by ^{1}H-NMR. Full conversion of thianthrenium dication **2-INT** to the alkenyl thianthrenium salt **2-TT** was observed.

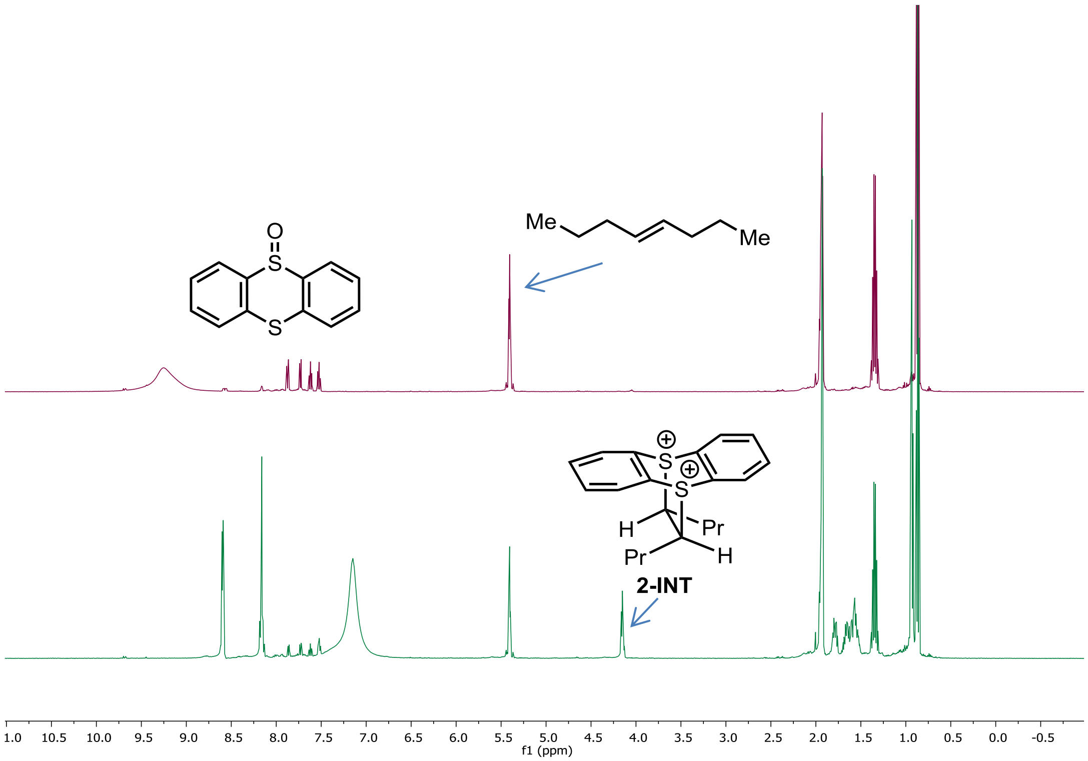

Figure S1: NMR spectra for the reaction of thianthrene-S-oxide, trifluoroacetic anhydride with trans-4-octene before and after addition of triflic acid.

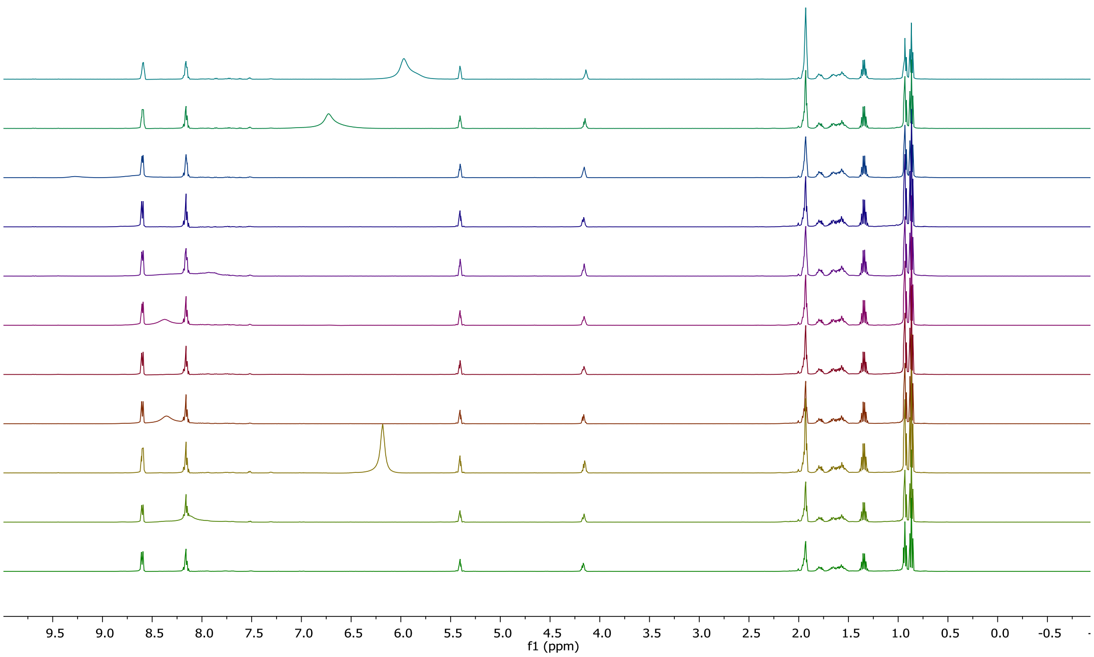

Figure S2: NMR spectra for the reaction of thianthrene-S-oxide, trifluoroacetic anhydride with trans-4-octene after addition of triflic acid between 2’30” and 120’ (from top to bottom: 2’30”, 4’30”, 7’, 10’, 15’, 20’, 30’, 40’, 60’, 90’, and 120’).

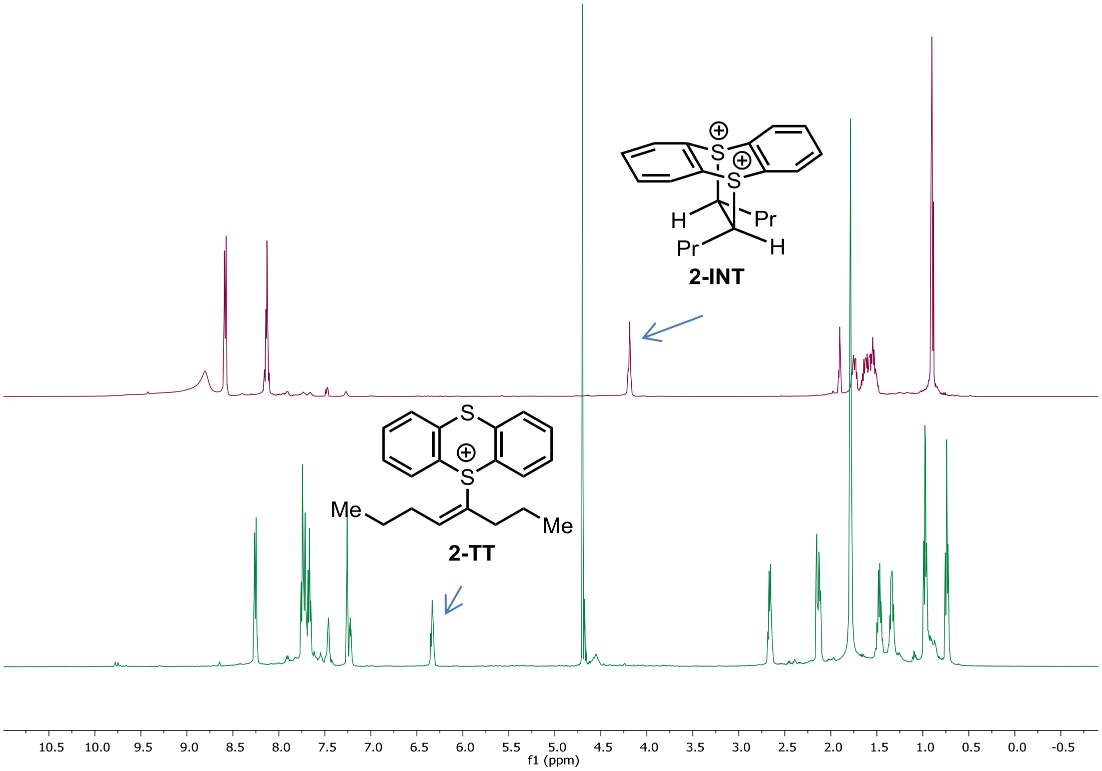

Figure S3: NMR spectra for the reaction for trans-4-octene-derived thianthrenium salt before and after deprotonation (top: concentrated residue; bottom: after deprotonation).

Synthesis of 2-TT from 2-INT

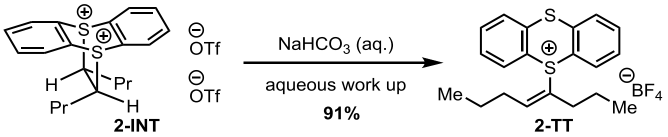

Under ambient atmosphere, a 20 mL borosilicate vial was charged with cis-4-octene-derived thanthrenium dication **2-INT** (100 mg, 160 µmol, 1.00 equiv.). The beige solid was dissolved with CH_2Cl_2 (10 mL) and poured onto a saturated aqueous $NaHCO_3$ solution (ca. 10 mL). The combined mixture was poured into a separatory funnel, and the layers were separated. The CH_2Cl_2 layer was collected, and the aqueous layer was further extracted with CH_2Cl_2 (2 × ca. 10 mL). The combined CH_2Cl_2 solution was washed with aqueous $NaBF_4$ solution (2 × ca. 10 mL, 5 % w/w). The CH_2Cl_2 layer was dried over Na_2SO_4, filtered, and the solvent was removed under reduced pressure. The residue was triturated with Et_2O (3 ×5.0 mL), and Et_2O was removed by pipette. The solid was collected and further dried in vacuo to afford **2-TT** (*E*/*Z* < 50/1, 60.1 mg, 145 µmol, 91 %) as a colorless solid.

Synthesis of 3-TT from 3-INT

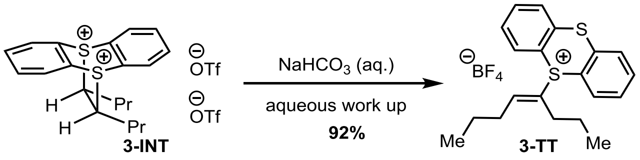

Under ambient atmosphere, a 20 mL borosilicate vial was charged with cis-4-octene-derived thanthrenium dication **3-INT** (100 mg, 160 μmol, 1.00 equiv.). The beige solid was dissolved with CH_2Cl_2 (10 mL) and poured onto a saturated aqueous $NaHCO_3$ solution (ca. 10 mL). The combined mixture was poured into a separatory funnel, and the layers were separated. The CH_2Cl_2 layer was collected, and the aqueous layer was further extracted with CH_2Cl_2 (2 × ca. 10 mL). The combined CH_2Cl_2 solution was washed with aqueous $NaBF_4$ solution (2 × ca. 10 mL, 5 % w/w). The CH_2Cl_2 layer was dried over Na_2SO_4, filtered, and the solvent was removed under reduced pressure. The residue was triturated with Et_2O (3 ×5.0 mL), and Et_2O was removed by pipette. The solid was collected and further dried in vacuo to afford **3-TT** (*E*/*Z* > 50/1, 62.2 mg, 148 μmol, 92 %) as a colorless solid. (When the reaction was executed in D_2O with $NaHCO_3$, only **3-TT** was observed, no H/D scrambling was detected.)

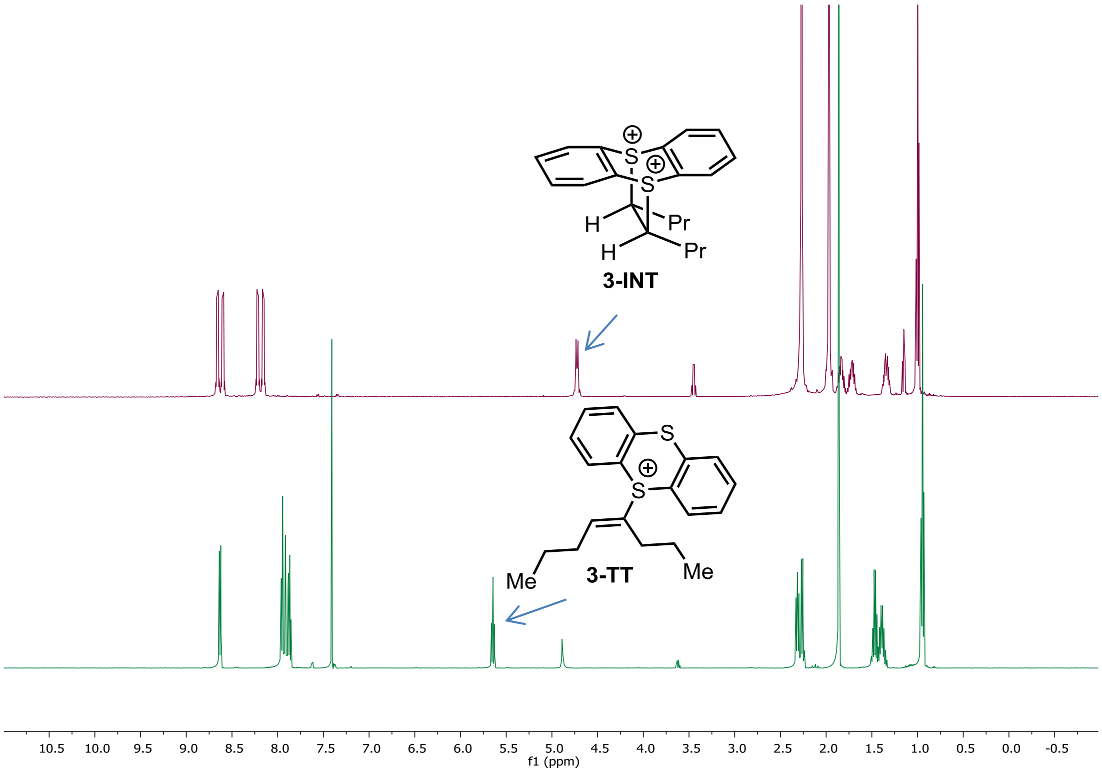

Figure S4: NMR spectra for the reaction cis-4-octene-derived thianthrenium salt before and after deprotonation (top: concentrated residue; bottom: after deprotonation).

Formation and stability of trans-4-octene adduct

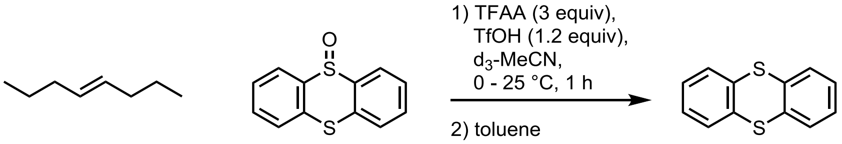

Under an ambient atmosphere, a GC-vial was charged with Teflon coated stir bar, thianthrene-*S*-oxide (**1**) (34 mg, 0.15 mmol, 1.0 equiv), trans-4-octene (17 mg, 0.15 mmol, 1.0 equiv) and CD_3CN (0.5 mL). The reaction mixture was cooled to 0 °C, at which point trifluoroacetic anhydride (0.060 mL, 91 mg, 0.43 mmol, 2.9 equiv) and trifluoromethanesulfonic acid (0.016 mL, 27 mg, 0.18 mmol, 1.2 equiv) were added sequentially. The reaction mixture was warmed to room temperature, transferred to an NMR tube, and analyzed by ^{1}H NMR (Fig. S5, top). At this point, toluene (14 mg, 0.15 mmol, 1.0 equiv) was added to the NMR tube. After 1.5 h, there was no discernable decomposition by ^{1}H NMR (Fig S5, middle), and the NMR tube was heated at 60 °C. After 4 h of heating at 60 °C, ^{1}H NMR analysis showed thianthrene as the primary decomposition product (Fig. S5, bottom). No toluene derived aryl thianthrenium salt could be observed, and the fate of the hydrocarbon motif was not determined.

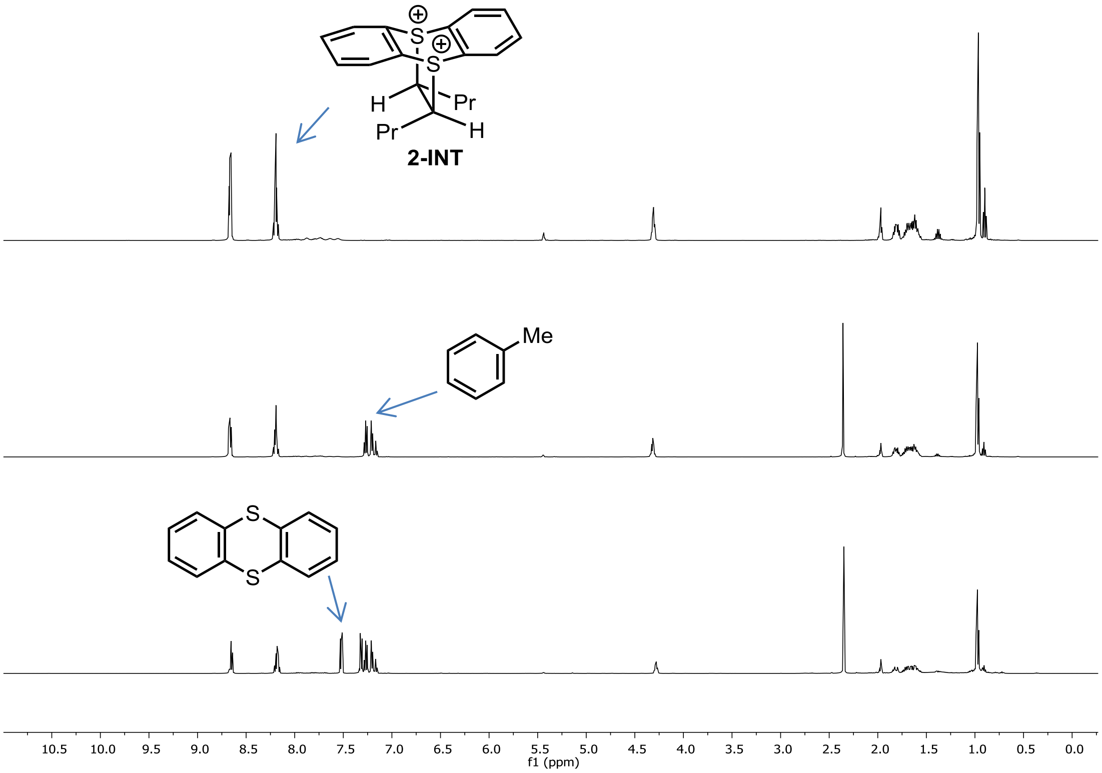

Figure S5: ^{1}H NMR spectra for **2-TT** reaction with toluene during the course of 4 hours.

Attempt at retrocyclization and trapping with o-xylene

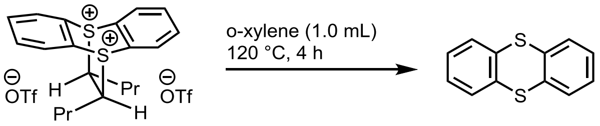

2-INT

An oven-dried 10-mL microwave vial was charged with a teflon-coated stir bar, trans-4-octene cyclo-adduct **2-INT** (25 mg, 0.040 mmol), and sealed with a crimp cap with a septum. Via the septum, the vial was subjected to three vacuum-argon cycles before freshly distilled o-xylene (1.0 mL) was added. The microwave vial was placed in a preheated oil bath (120 °C) and heat for 4 h. An attempt to precipitate aryl thianthrenium salt by addition of diethyl ether (4 mL) failed, so the solvent was removed via rotary evaporator. The resulting solid was analyzed by ^{1}H NMR (Fig. S6).

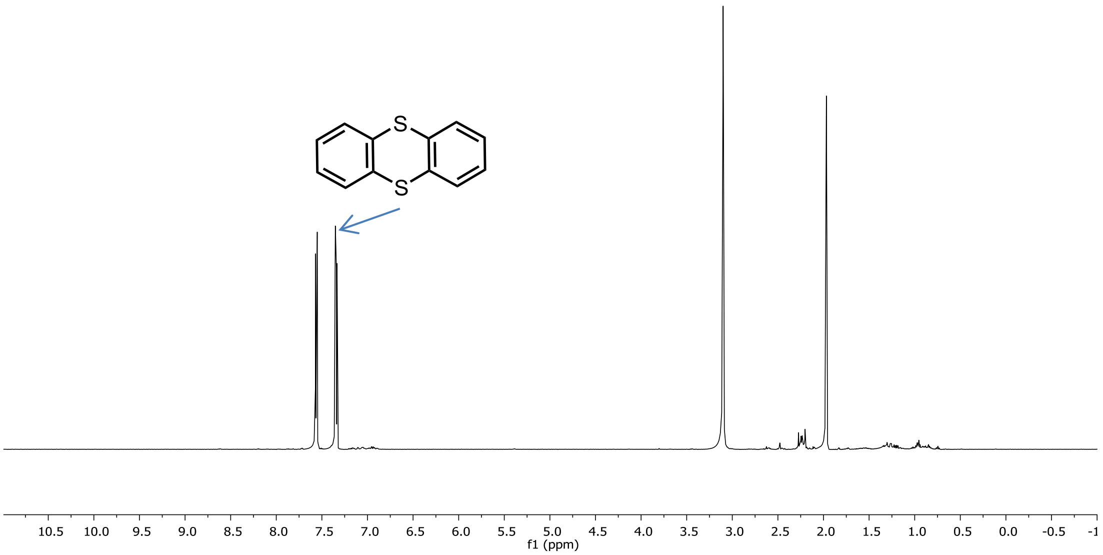

Figure S6: ^{1}H NMR spectra for the resulting solid after treatment with xylene.

Attempt at retrocyclization by vacuum pyrolysis

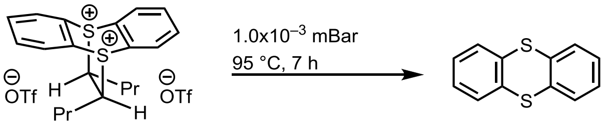

2-INT

A flame dried 10-mL schlenk tube was charged with trans-4-octene cyclo-adduct **2-INT** (25 mg, 0.040 mmol), and subjected to three vacuum-argon cycles. The schlenk tube was brought to a pressure of 1.0x10^{-3} mBar and placed in a preheated oil bath (95 °C). The material was heated for 7 h, at which point the schlenk tube was cooled before being filled with argon. A solution of toluene (4 mg, 0.04 mmol, 1 equiv) in CD_3CN was added to the schlenk tube under argon and then transferred to an NMR tube. The solution was analyzed by ^{1}H NMR (Fig. S7) and showed no evidence for aryl thianthrenium.

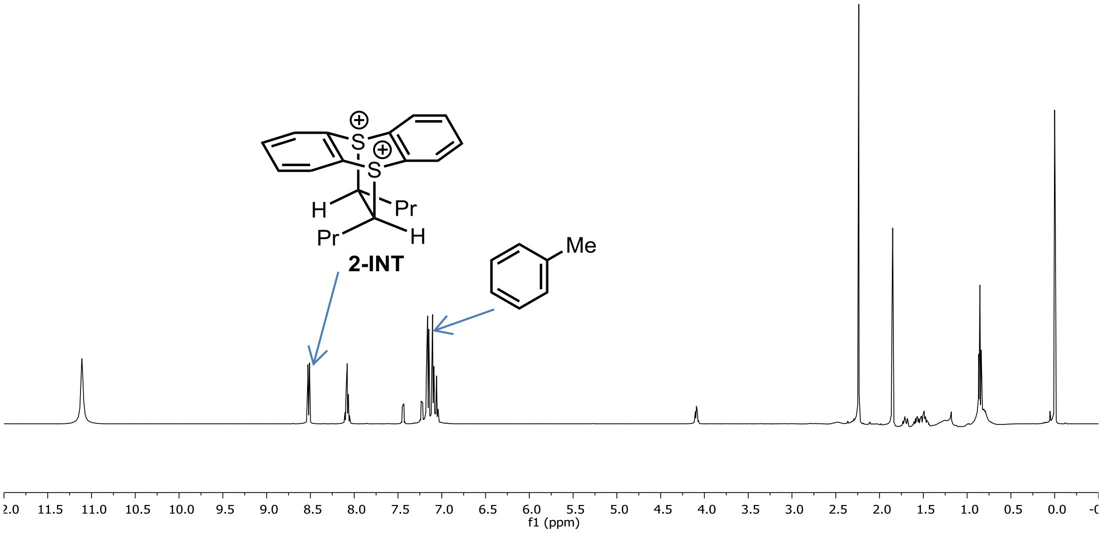

Figure S7: ^{1}H NMR spectra for the resulting mixture after treatment with toluene.

Studies on thianthrenium dication formation in solution

Cyclic Voltammetry

A 50-mL solution stock solution of ferrocene (46 mg, 0.25 mmol), thianthrene (54 mg, 0.25 mmol), and trifluoroacetic anhydride (0.42 mL, 630 mg, 3.0 mmol) in MeCN was prepared using a 50-mL volumetric flask. Sodium triflate (86 mg, 0.50 mmol) was dissolved with 5 mL of the stock solution in a 20-mL vial. A glassy-carbon working electrode (3.0 mm, Part #: MF-2114 from BASi), a platinum wire counter electrode (MW-1032), and a non-aqueous silver/silver Ion (Ag/Ag^{+}) reference electrode were submersed in the solution then the cyclic voltammogram was obtained at 25 °C with an BASi Model E2 Epsilon potentiostat connected to a BASi C3 Cell Stand. Potentials from 0 to 2200 mV were scanned at a sweep rate of 87 $mV{\cdot}s^{-1}$. The resulting cyclic voltammogram is shown in Figure S8. Ferrocene internal standard (Fc/Fc^{+} = 0.37 V); thianthrene to thianthrene radical cation peak potential = 1.21 V; thianthrene radical cation to thianthrene dication = 1.74 V.

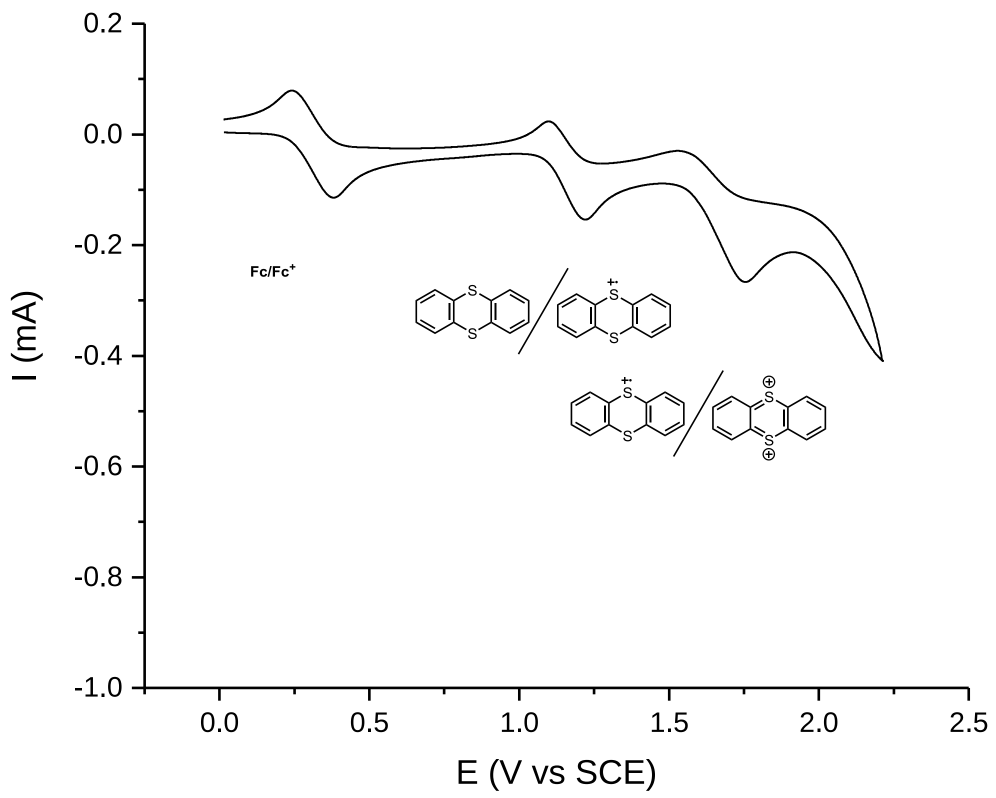

Figure S8: Cyclic voltammogram for the oxidation of thianthrene (5 mM) in acetonitrile containing sodium triflate (0.1 M), trifluoroacetic anhydride (60 mM), and a ferrocene internal standard (5 mM); sweep rate = 87 $mV·s^{-1}$.

Constant Potential UV-vis Spectroscopy

Trifluoroacetic anhydride (0.040 mL, 0.060 g, 0.29 mmol), tetrafluoroboric acid diethyl etherate (0.060 mL, 0.071 g, 0.44 mmol), and thianthrene (4.3 mg, 0.019 mmol, c = 0.80 mM) were dissolved in acetonitrile using a 25-mL volumetric flask. A thin-layer quartz cuvette (Pine Research Instrumentation, part no. RRPG094) was filled with the 0.80 mM thianthrene solution and the honeycomb electrode card (Pine Research Instrumentation, part no. AB01STC1AU) was inserted into the cuvette. The cuvette was fitted into a Cary 50 spectrometer) and the UV-vis absorption spectrum was taken with an applied voltage of 0.0 V (Figure S9, top left). Following the 0.0 V measurement, a voltage of 1.4 V was applied for 10 min using aMetrohm Autolab pgstat302N potentiostat), at which point the UV-vis absorption spectrum was recorded (Figure S9, top right). By visually inspecting the UV-vis cell, the characteristic purple-colored radical cation was observed. Finally, a voltage of 1.8 V was applied for 10 min, and the UV-vis absorption spectrum was recorded (Figure S9, bottom). Visual inspection of the cell showed a colorless solution in the proximity of the electrode with the formation of a purple colored species further away from the electrode (attributed to reduction of dication).

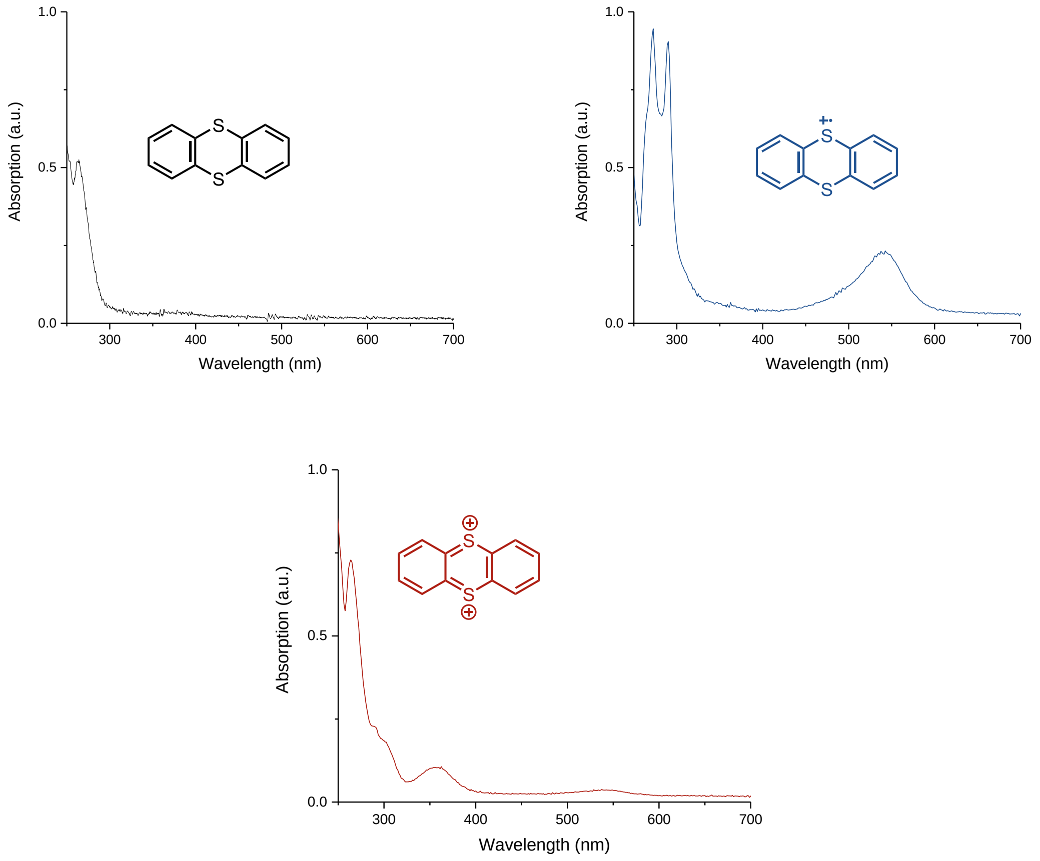

Figure S9: Constant potential UV-vis absorption spectra of thianthrene (0.8 mM) in acetonitrile containing trifluoroacetic anhydride, and tetrafluoroboric acid diethyl etherate with an applied potential of 0.0 V (top left), 1.4 V (top right), and 1.8 V (bottom).

Density Functional Theory calculations

Methods

Density Functional Theory (DFT) calculations were performed on the Max-Planck-Institut für Kohlenforschung computer cluster using the ORCA program package (Version 4.2.x-Stable)[5]. Unless denoted otherwise, structural optimizations were performed with the B3LYP functional[6,7] with D3 dispersion correction[8] and Becke-Johnson damping (BJ)[9] along with RI approximation, utilizing the def2/J auxiliary basis set[10] and the def2-TZVPP basis set[11] on all atoms. The libint2 library was used for the computation of 2-el integrals[12]. Tight SCF convergence and geometry optimization criterions were chosen. Very tight SCF convergence was chosen for cycloadduct **3-INT**. Solvent effects of acetonitrile were taken into account using the conductor-like polarized continuum model (CPCM)[13]. Input files were created using Avogadro 1.2[14]. Geometry optimized structures were visualized using Chemcraft 1.8[15].

DFT evaluation on disulfonium species

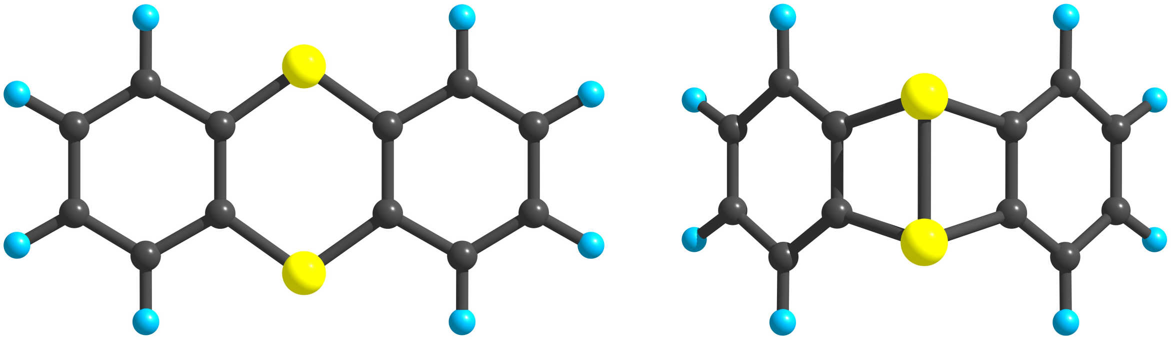

Figure S10: B3LYP geometry optimized structures of the six-membered thianthrene dication (left) at 0.0 kcal·mol^{-1} relative to the bicyclo thianthrene dication (right) at 47.1 kcal·mol^{-1}.

Calculated Coordinates:

six-membered thianthrene dication

S 7.5625537375 2.4130577024 4.4426801167

S 4.8735293639 0.8648595682 5.8074233574

C 6.2348256262 1.3865482314 6.6569321075

C 6.2069640412 1.1363300810 8.0426281381

C 7.2669090254 1.5229167840 8.8186062761

C 8.3890810184 2.1718321425 8.2486339721

C 8.4469364126 2.4308218036 6.9053015386

C 7.3743664749 2.0442899736 6.0783595446

C 6.2011584450 1.8915672762 3.5931928210

C 6.2290033563 2.1418304573 2.2075180314

C 5.1691952127 1.7549818622 1.4314783078

C 4.0471003491 1.1059098013 2.0014217395

C 3.9891817965 0.8470387762 3.3447767297

C 5.0616788032 1.2336929634 4.1717488367

H 5.3482747401 0.6428749166 8.4750643968

H 7.2470356986 1.3322600436 9.8817072773

H 9.2108794480 2.4675309350 8.8842270169

H 9.3018523557 2.9264283478 6.4679499894

H 7.0875506338 2.6355907279 1.7751441750

H 5.1891035232 1.9455797523 0.3683667681

H 3.2253936517 0.8100422898 1.3657888872

H 3.1342262859 0.3515255636 3.7821599718

Starting geometry for bicyclo thianthrene dication

S 6.2162800000 -0.4980700000 5.2120900000

S 7.8293900000 0.4413600000 4.3877900000

C 5.4497400000 0.5528300000 4.0761500000

C 4.1635300000 0.8001200000 3.6689700000

C 3.9984800000 1.7567600000 2.6618900000

C 5.1140900000 2.4158100000 2.1002100000

C 6.4112700000 2.1278400000 2.5371800000

C 6.5301200000 1.1923400000 3.5331700000

C 7.6899000000 1.3251700000 5.8684800000

C 8.4002300000 2.3677400000 6.5632000000

C 7.8922700000 2.6727700000 7.7700500000

C 6.7261700000 2.0097600000 8.3533800000

C 6.0403600000 1.0258300000 7.7452300000

C 6.5439700000 0.6727900000 6.4430400000

H 3.3186600000 0.2789000000 4.1016400000

H 2.9998600000 1.9921800000 2.3002900000

H 4.9611500000 3.1500800000 1.3122500000

H 7.2773200000 2.6144500000 2.1061200000

H 9.2652900000 2.8436700000 6.1259100000

H 8.3612100000 3.4472600000 8.3713900000

H 6.4225200000 2.3451400000 9.3416800000

H 5.1849900000 0.5228100000 8.1710100000

Geometry optimized bicyclo thianthrene dication

S 6.1181909260 -0.4604407592 5.2757902282

S 7.8653523669 0.5499872741 4.3842275195

C 5.3861923815 0.5958247518 4.0214143142

C 4.0980563363 0.8069454824 3.5898393790

C 3.9707388837 1.7376351648 2.5516671545

C 5.0755939706 2.3781908672 1.9894893225

C 6.3820054332 2.1310130654 2.4278880747

C 6.4835866566 1.2309923387 3.4619501701

C 7.6945603806 1.3559305360 5.9811035808

C 8.4025724861 2.3369051064 6.6339503943

C 7.9185307736 2.6647549696 7.9063741328

C 6.8159744751 2.0211238977 8.4700160124

C 6.1224662118 1.0079122073 7.7972655518

C 6.5990187858 0.7176505177 6.5409117149

H 3.2480203567 0.2934638547 4.0127616054

H 2.9845907556 1.9554154882 2.1680137927

H 4.9228681787 3.0800510704 1.1822698634

H 7.2409231317 2.6088224971 1.9815421836

H 9.2687471289 2.8171048542 6.2049686518

H 8.4256147582 3.4332769570 8.4717535911

H 6.4910121841 2.3025593890 9.4609299061

H 5.2821834384 0.4924204695 8.2369928561

Discussion on six-membered and bicyclo[2.2.0] disulfonium species

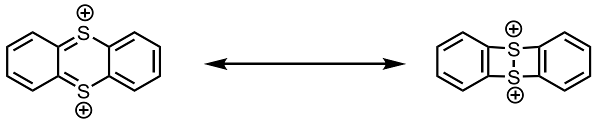

The B3LYP geometry optimized structure of the six-membered thianthrene dication (Figure S10, left) was obtained using thianthrene as a starting geometry with a +2 charge. The B3LYP geometry optimized bicyclo structure (Figure S10, right) was obtained by using the Avogadro geometry optimization extension with thianthrene as a starting geometry, however, with an explicit S–S bond. The Avogadro generated structure was then optimized using the standard computational method. Both structures converged to an energy minimum, however, the Gibb's free energy for the tricyclo structure was calculated to be –1257.81179186 Eh, while the bicyclo structure had an energy –1257.73675187 Eh. Therefore, the six-membered thianthrene dication is expected to be the more stable conformation by 47.1 kcal·mol^{-1}. This six-membered ring dication is also evidenced by the isolation and characterization of the dication of 2,3,7,8-tetramethoxythianthrene.[16] ^{1}H-NMR data suggested from a separate publication also proposes six-membered ring dication.[17]

Discussion on elimination mechanism

The elimination cannot proceed via an E2 elimination mechanism due to stereoelectronic constraints. Also, the product cannot be formed by E1 elimination because the dication is stable in solution in the absence of base (see Reactivity studies on cycloaducts part). The reaction could proceed via a sulfonium ylide intermediate (E1cB). A reversible E1cB mechanism (E1cB$_{rev}$) can be ruled out because we executed an H/D scrambling experiment and did not observe scrambling. Alternatively, the reaction could proceed via an irreversible E1cB$_{irr}$ mechanism, where rate-determining deprotonation is followed by a rapid elimination.[18] All data observed is consistent with an E1cB$_{irr}$ mechanism.

References

1. A. E. Cohrt, T. E. Nielsen, *ACS Comb. Sci.* **2014**, *16*, 71−77.
2. S. Mizuta, S. Verhoog, K. M. Engle, *J. Am. Chem. Soc.* **2013**, *135*, 2505–2508.
3. G. Yin, I. Kalvet, F. Schoenebeck, *Angew. Chem. Int. Ed.* **2015**, *54*, 6809–6813.
4. S. V. F. Hansen, E. Christiansen, C. Urban, B. D. Hudson, C. J. Stocker, *J. Med. Chem.* **2016**, *59*, 2841–2846.
5. F. Neese, *WIREs Comput. Mol. Sci.* **2012**, *2*, 73–78.
6. A. D. Becke, *J. Chem. Phys*. **1993**, *98*, 5648–5652.
7. C. Lee, W. Yang, R. G. Parr, *Physical Review B* **1988**, *37*, 785–789.
8. S. Grimme, J. Antony, S. Ehrlich, H. Krieg, *J. Chem. Phys.* **2010**, *132*, 154104.
9. S. Grimme, S. Ehrlich, L. Goerigk, *J. Comput. Chem.* **2011**, *32*, 1456–1465.
10. F. Weigend, *Physical Chemistry Chemical Physics* **2006**, *8*, 1057–1065.
11. F. Weigend, R. Ahlrichs, *Physical Chemistry Chemical Physics* **2005**, *7*, 3297–3305.
12. E. F. Valeev, Libint: A library for the evaluation of molecular integrals of many-body operators over Gaussian functions, *http://libint.valeyev.net/*
13. V. Barone, M. Cossi, *The Journal of Physical Chemistry A* **1998**, *102*, 1995–2001.
14. M. D. Hanwell, *et al. Journal of Cheminformatics* **2012**, *4*, 17.
15. G. A. Zhurko, Chemcraft - graphical program for visualization of quantum chemistry computations. *https://chemcraftprog.com.*
16. R. S. Glass, W. J. Britt, W. N. Miller, G. S. Wilson, *J. Am. Chem. Soc.* **1973**, *95*, 2375–2376.
17. H. Shine, L. J. T. Hughes, *J. Org. Chem.* **1966**, *31*, 3142–3146.
18. S. Alunni, F. De Angelis, L. Ottavi, M.Papavasileiou, F.Tarantelli, *J. Am. Chem. Soc.* **2005**, *127*, 15151-15160.

SPECTROSCOPIC DATA

trans-4-Octene-derived thianthrenium salt 2-TT

^{1}H NMR of trans-4-octene-derived thianthrenium salt 2-TT

CD_2Cl_2, 298 K

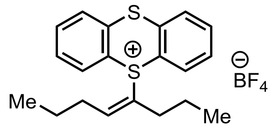

2-TT

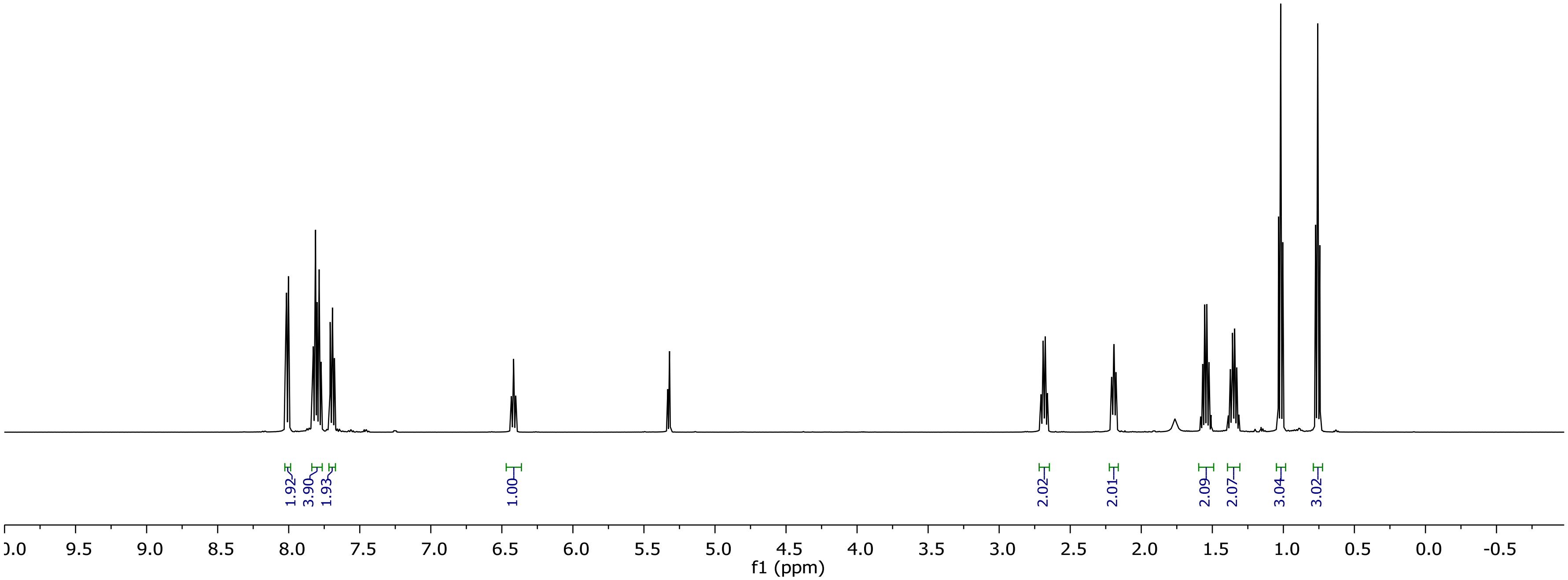

^{13}C NMR of trans-4-octene-derived thianthrenium salt 2-TT

CD_2Cl_2, 298 K

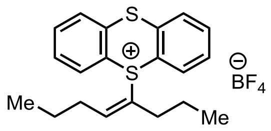

2-TT

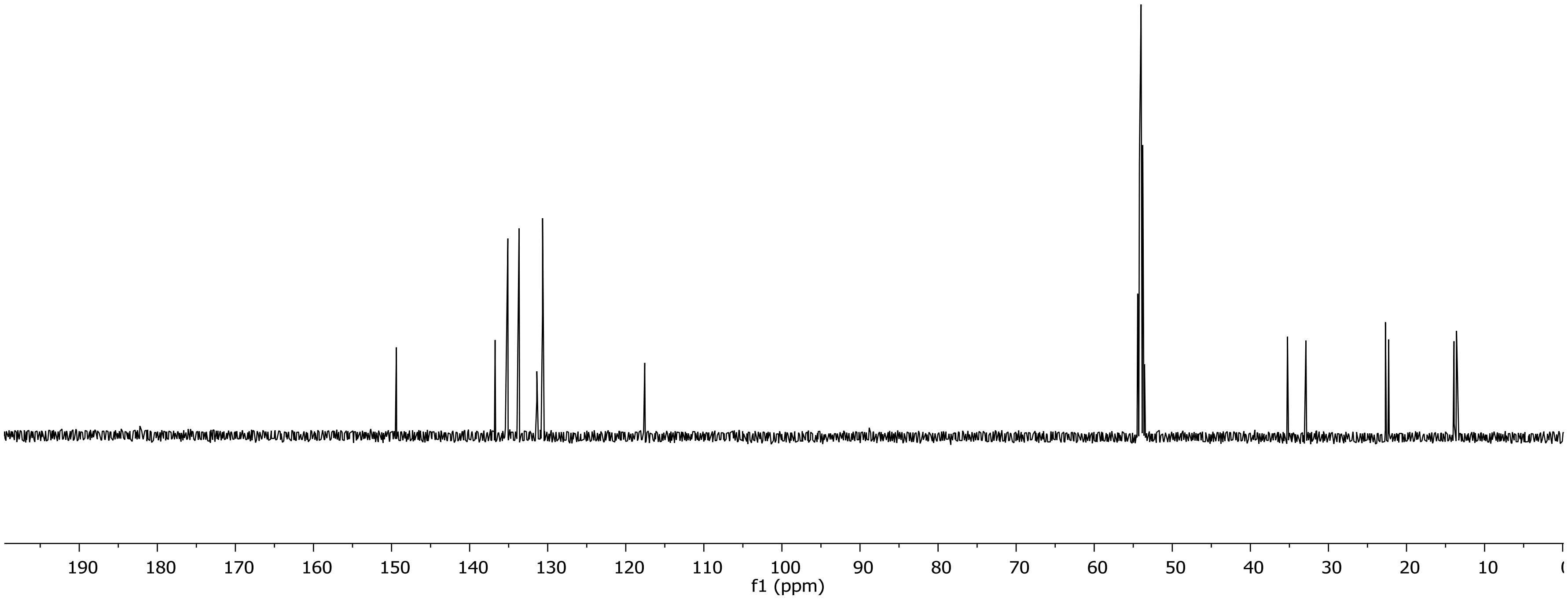

^{19}F NMR of trans-4-octene-derived thianthrenium salt 2-TT

CD_2Cl_2, 298 K

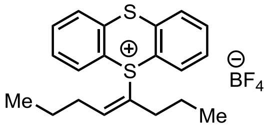

2-TT

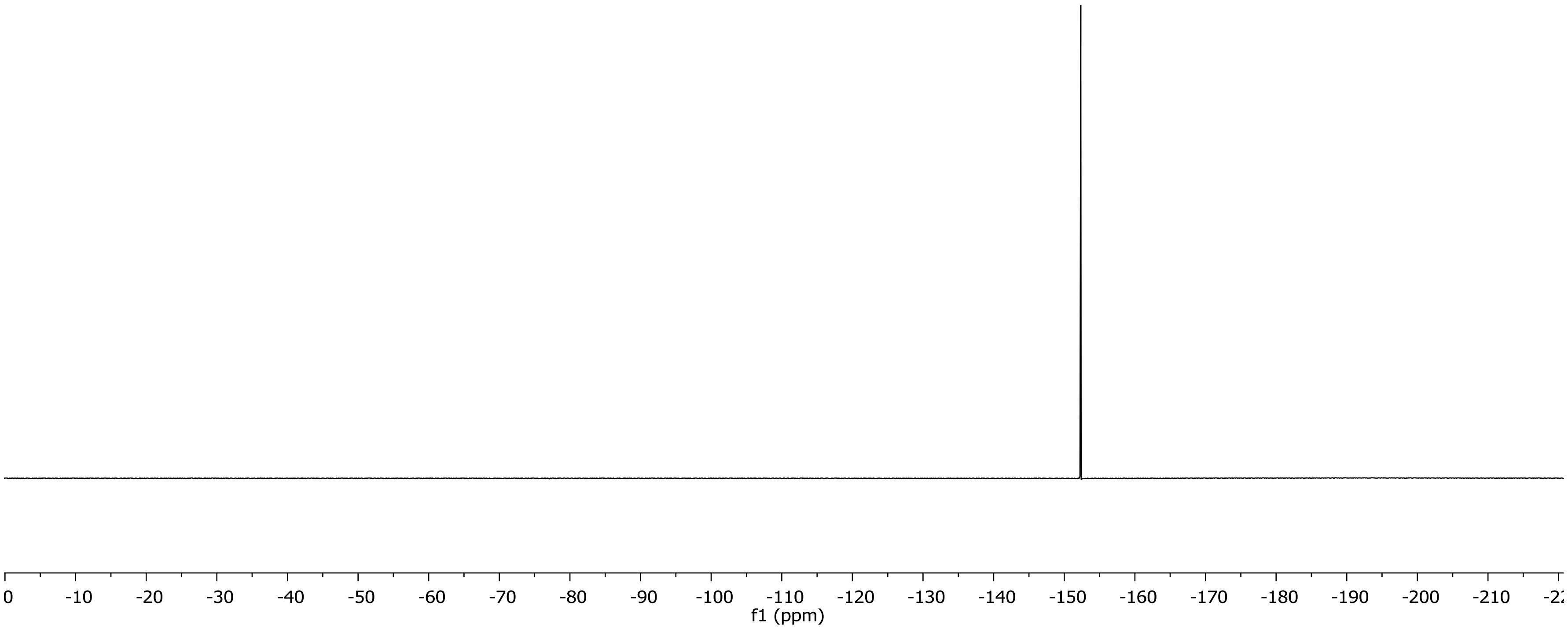

cis-4-Octene-derived thianthrenium salt 3-TT

^{1}H NMR of cis-4-octene-derived thianthrenium salt 3-TT (*E*)

$CDCl_3$, 298 K

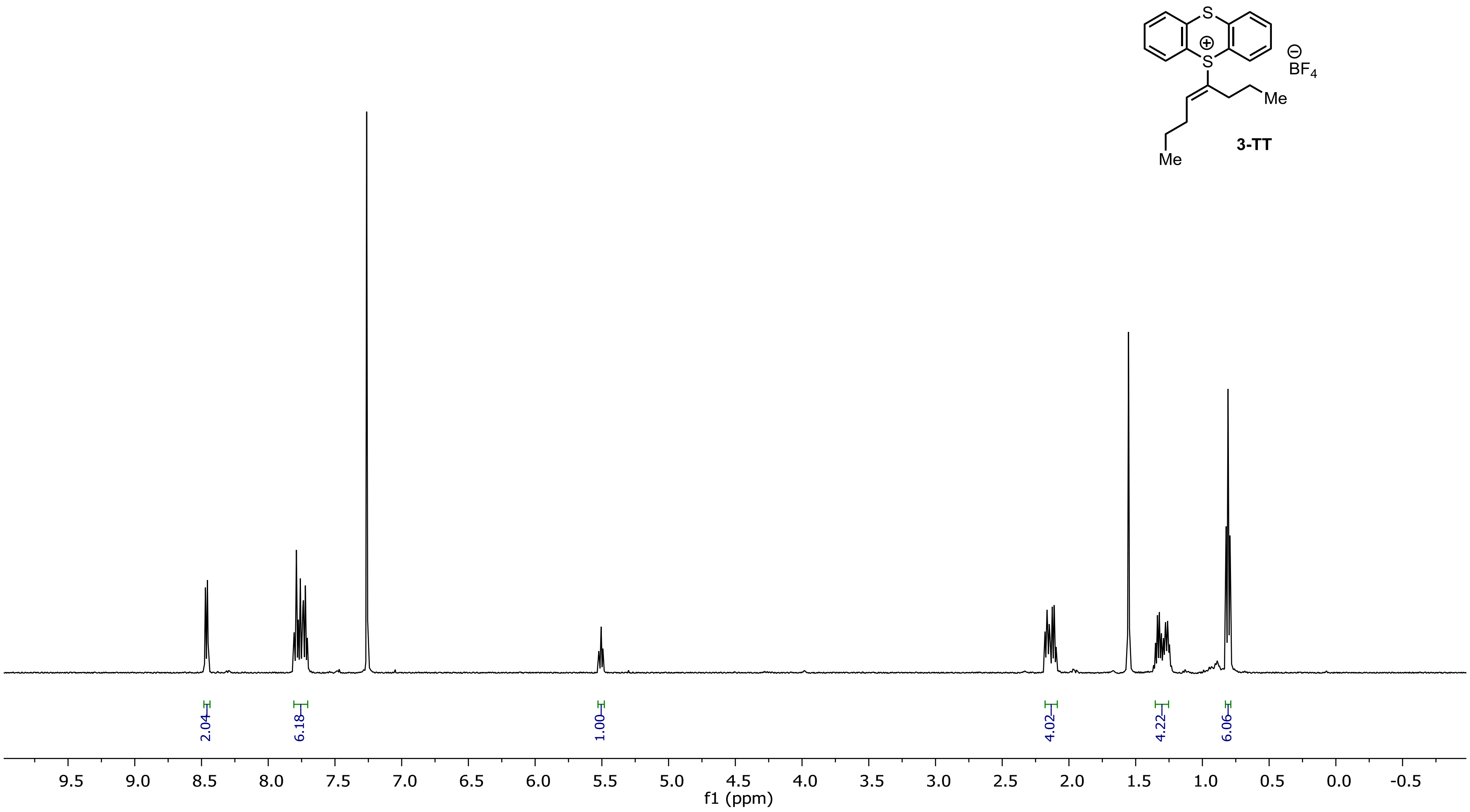

^{13}C NMR of cis-4-octene-derived thianthrenium salt 3-TT

CD_2Cl_2, 298 K

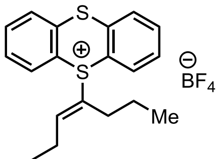

3-TT

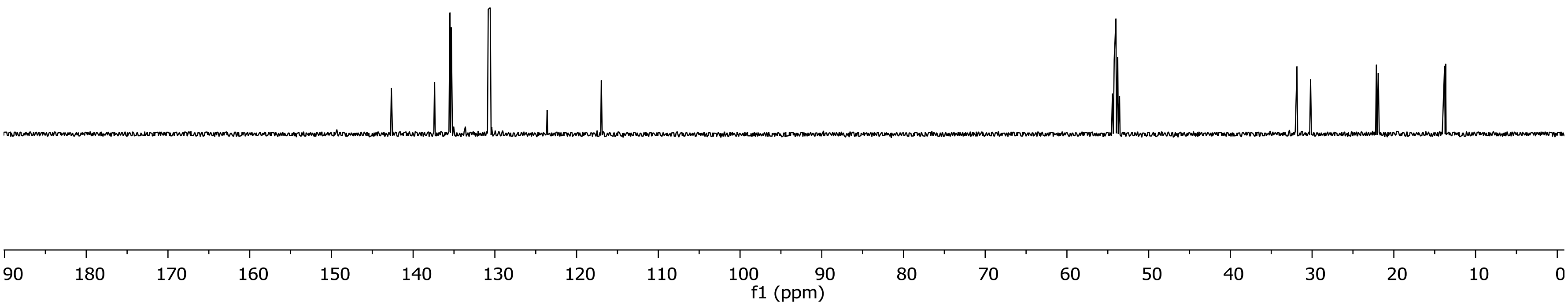

^{19}F NMR of cis-4-octene-derived thianthrenium salt 3-TT

CD_2Cl_2, 298 K

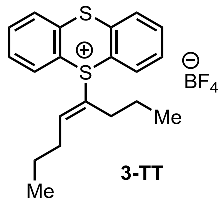

3-TT

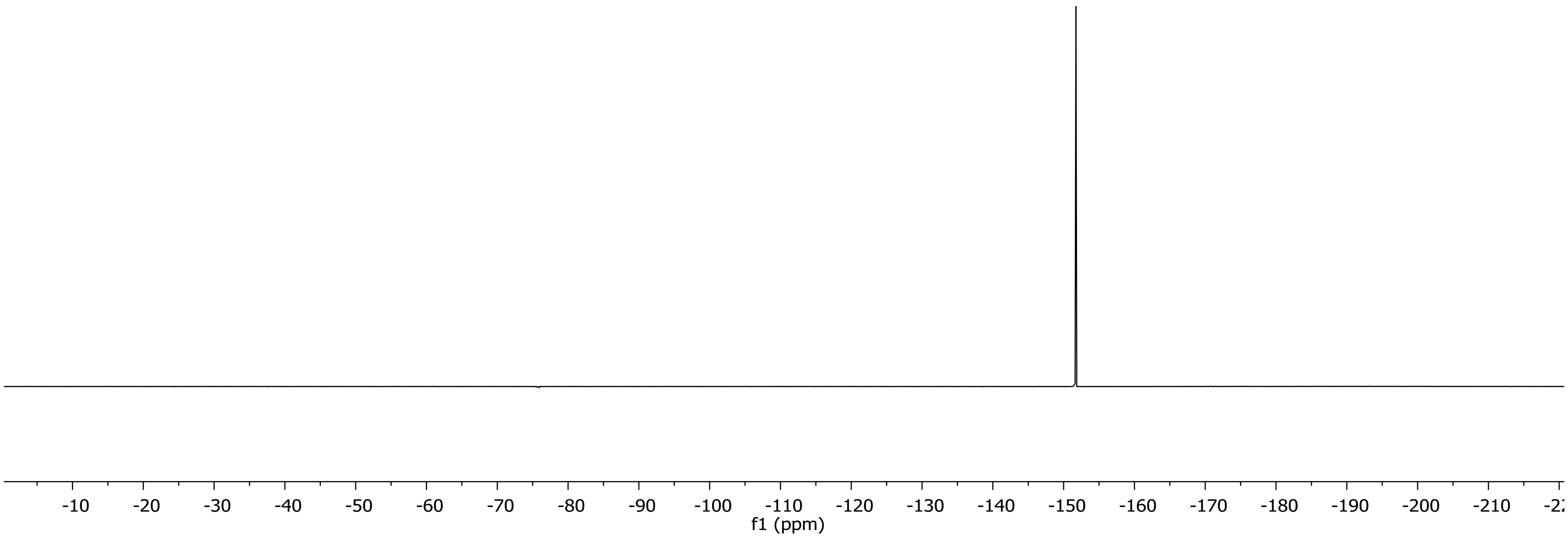

1-Octene-derived thianthrenium salt 4-TT

^{1}H NMR of 1-octene-derived thianthrenium salt 4-TT (*E*)

CD_2Cl_2, 298 K

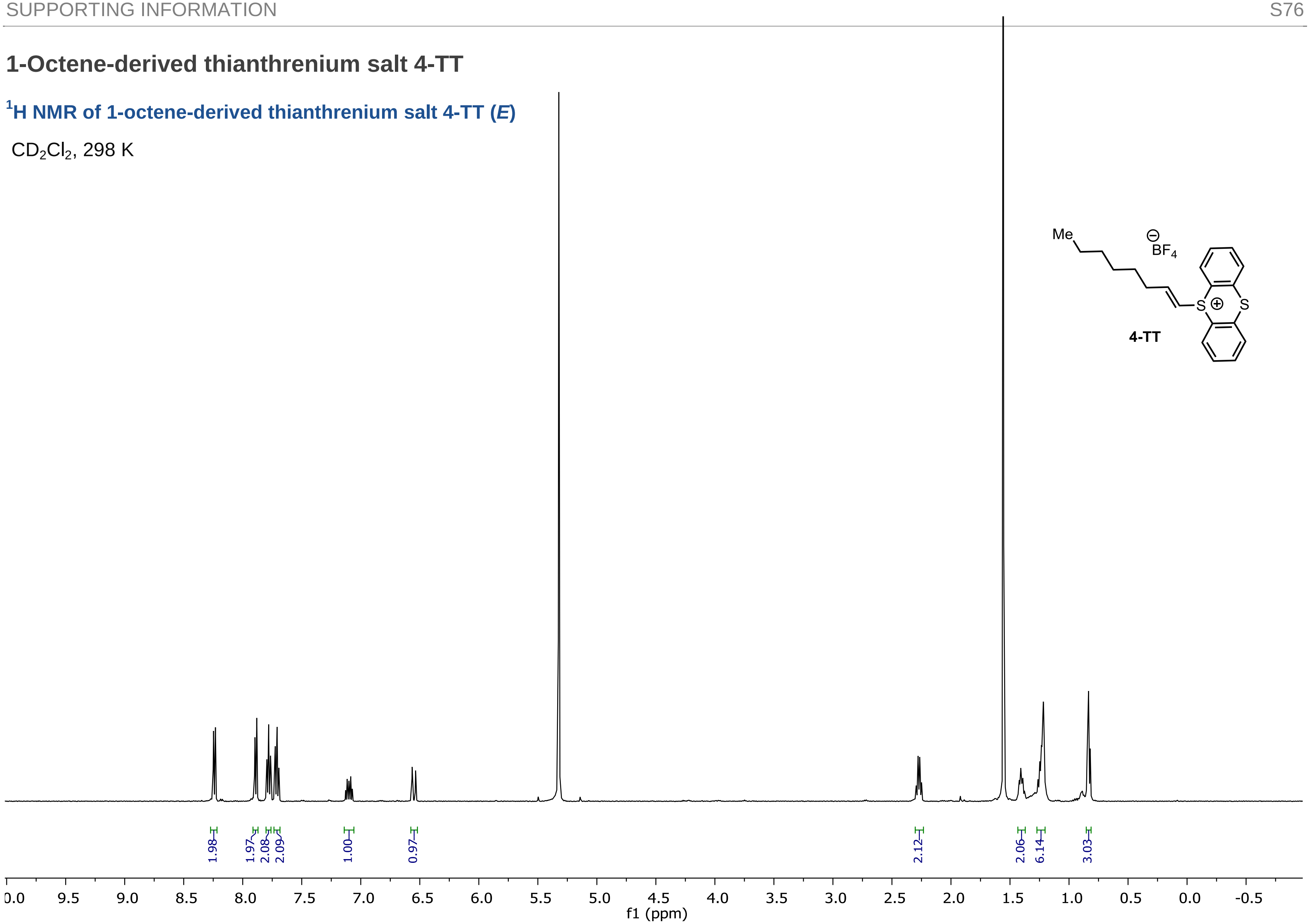

^{13}C NMR of 1-octene-derived thianthrenium salt 4-TT

CD_2Cl_2, 298 K

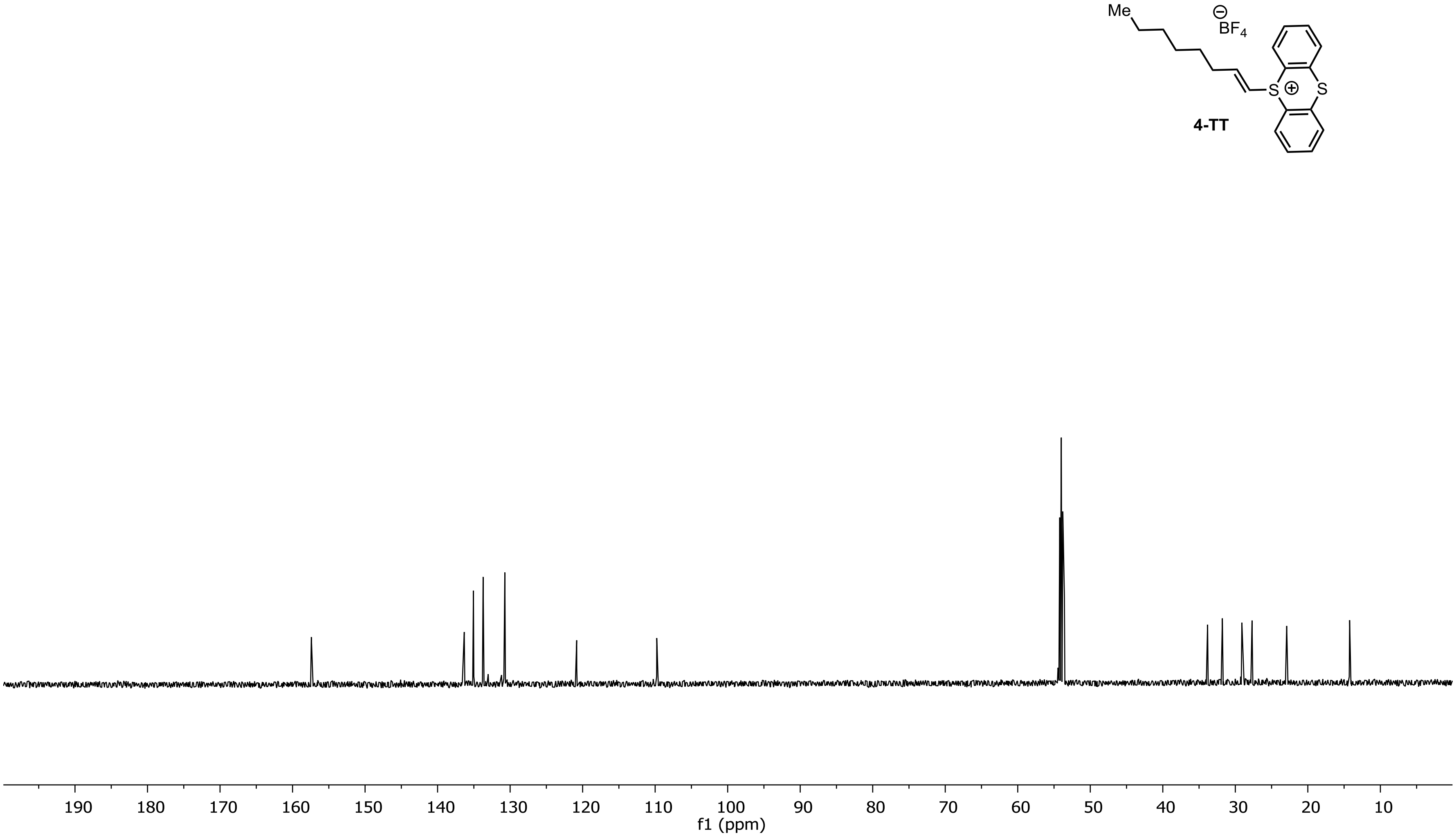

4-TT

^{19}F NMR of 1-octene-derived thianthrenium salt 4-TT

CD_2Cl_2, 298 K

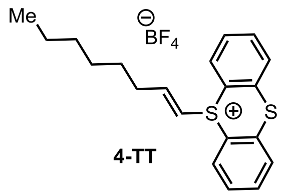

4-TT

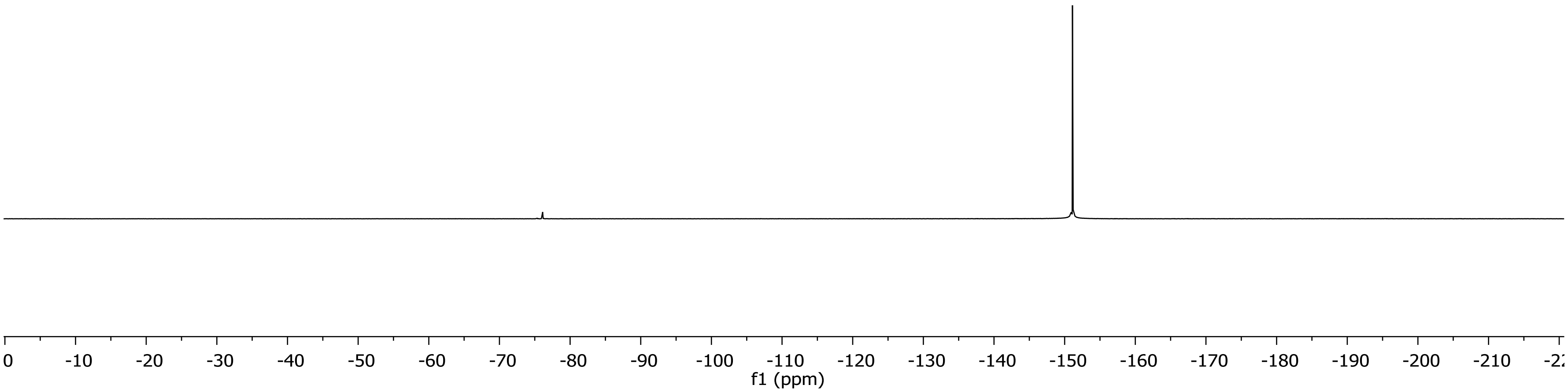

1,6-Heptadiene-derived thianthrenium salt 5-TT

^{1}H NMR of 1,6-heptadiene-derived thianthrenium salt 5-TT (*E*)

CD_2Cl_2, 298 K

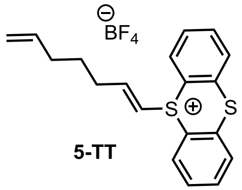

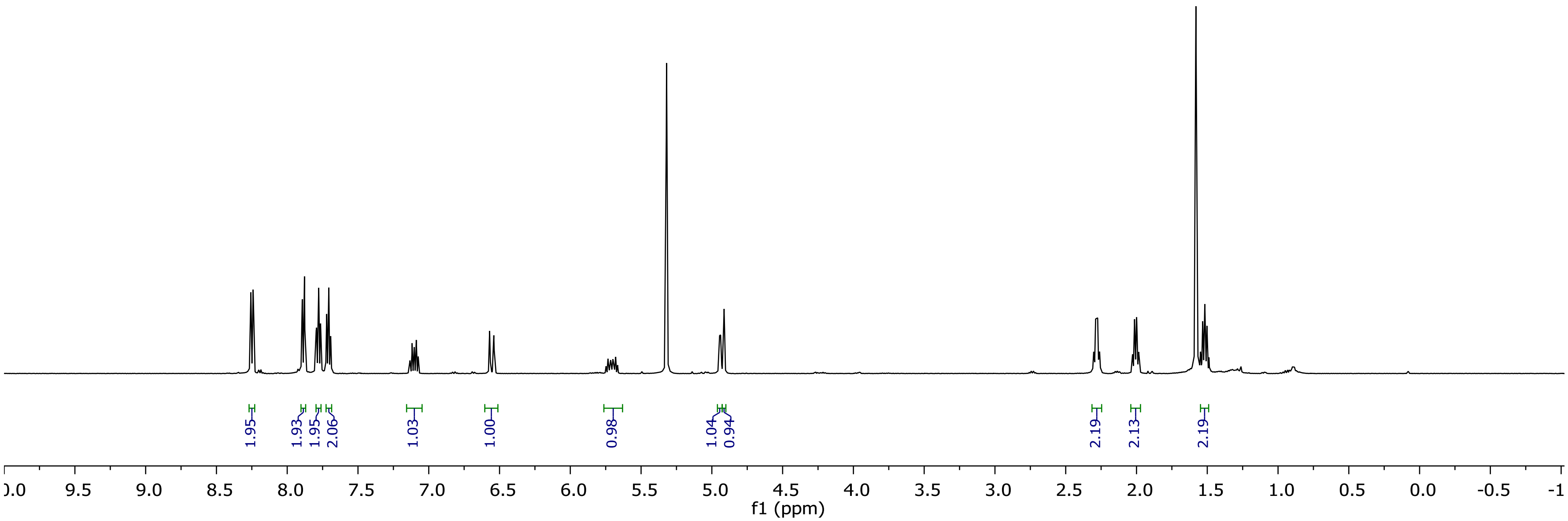

^{13}C NMR of 1,6-heptadiene-derived thianthrenium salt 5-TT

CD_2Cl_2, 298 K

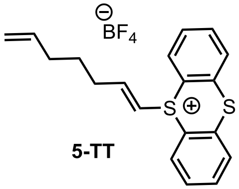

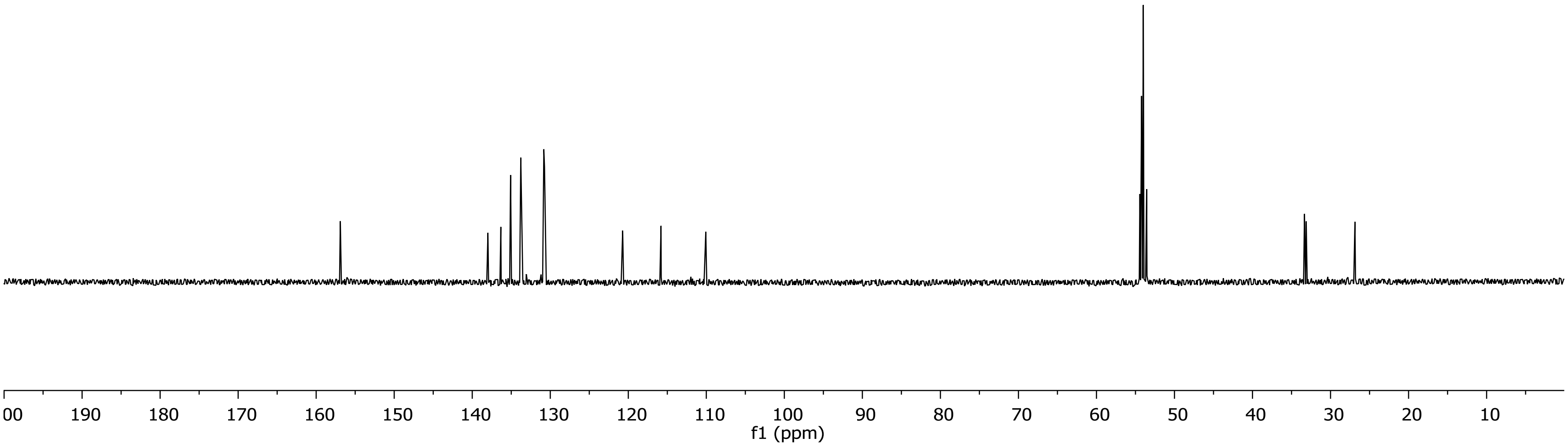

^{19}F NMR of 1,6-heptadiene-derived thianthrenium salt 5-TT

CD_2Cl_2, 298 K

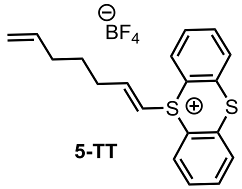

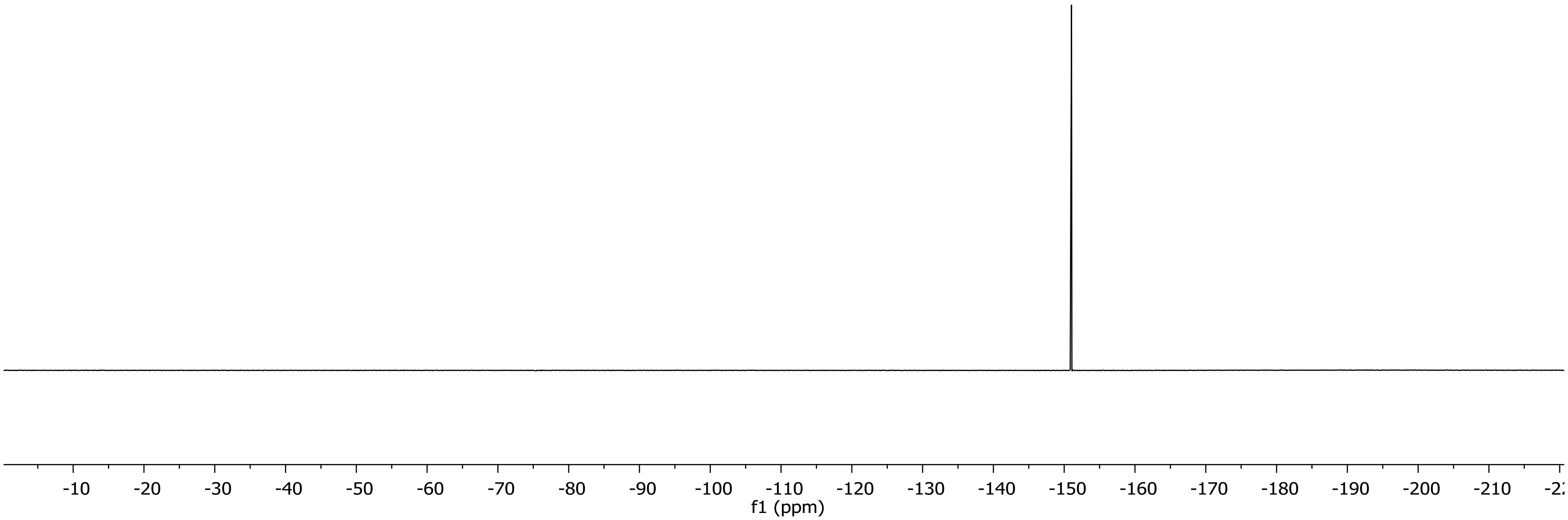

(-)-2-Vinylnorbornane-derived thianthrenium salt 6-TT

^{1}H NMR of (-)-2-vinylnorbornane-derived thianthrenium salt 6-TT (*E*)

CD_2Cl_2, 298 K

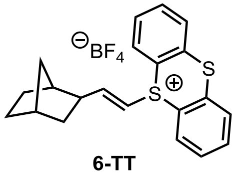

6-TT

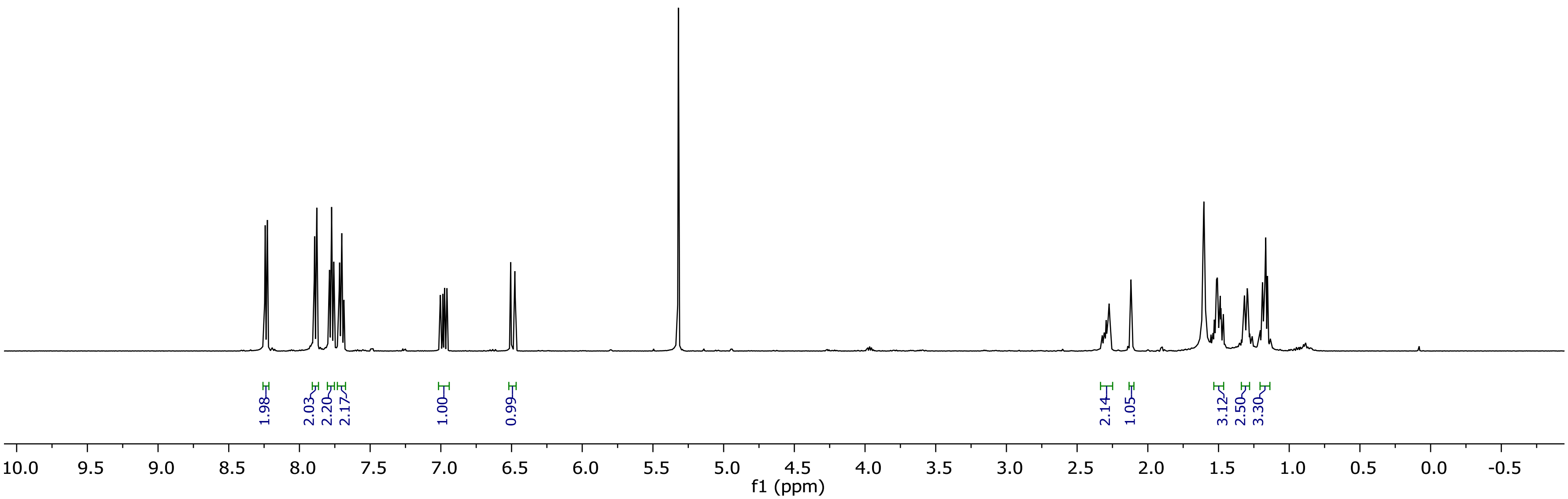

^{13}C NMR of (-)-2-vinylnorbornane-derived thianthrenium salt 6-TT

CD_2Cl_2, 298 K

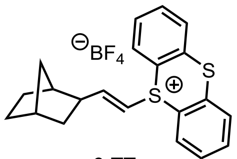

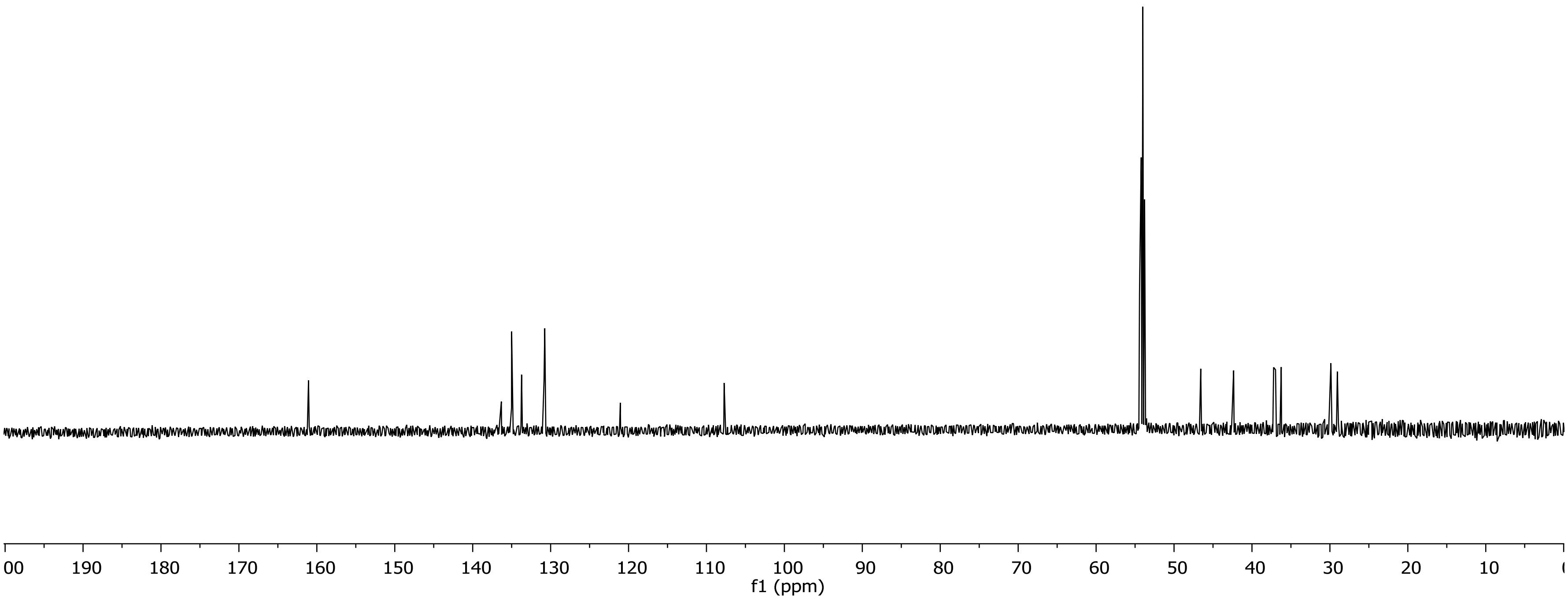

^{19}F NMR of (-)-2-vinylnorbornane-derived thianthrenium salt 6-TT

CD_2Cl_2, 298 K

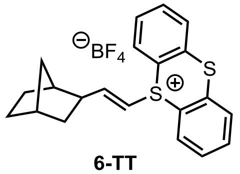

6-TT

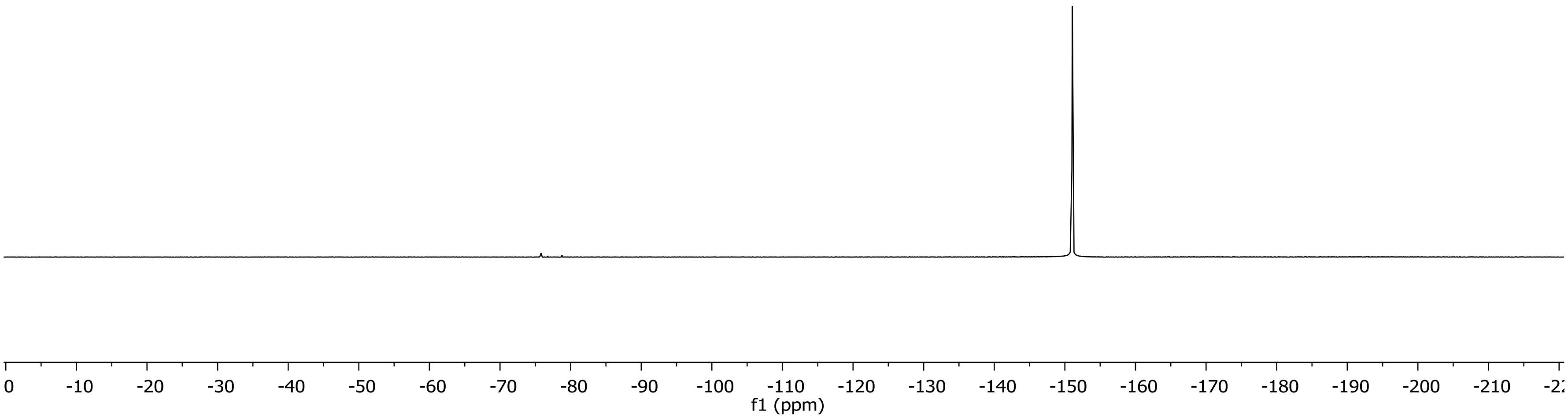

Vinylcyclooctane-derived thianthrenium salt 7-TT

^{1}H NMR of vinylcyclooctane-derived thianthrenium salt 7-TT

CD_2Cl_2, 298 K

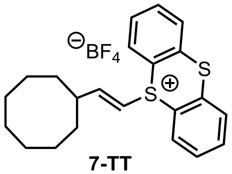

7-TT

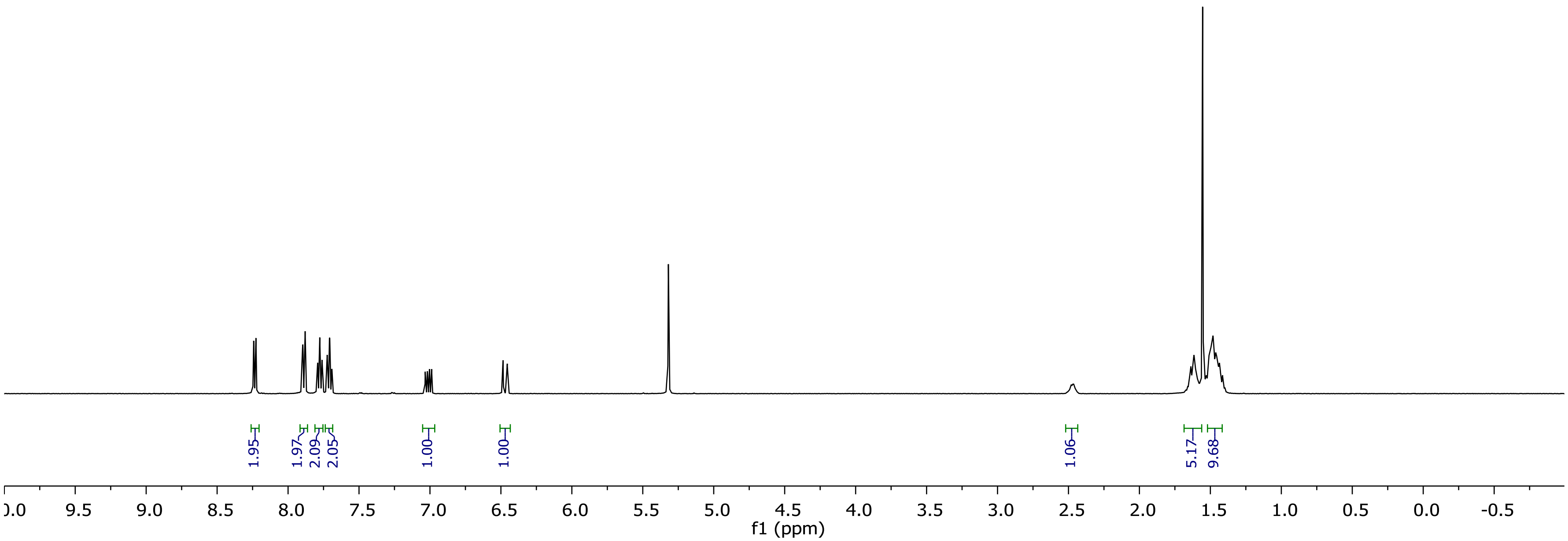

^{13}C NMR of vinylcyclooctane-derived thianthrenium salt 7-TT

CD_2Cl_2, 298 K

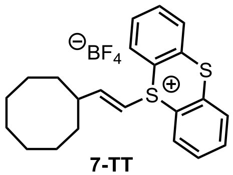

7-TT

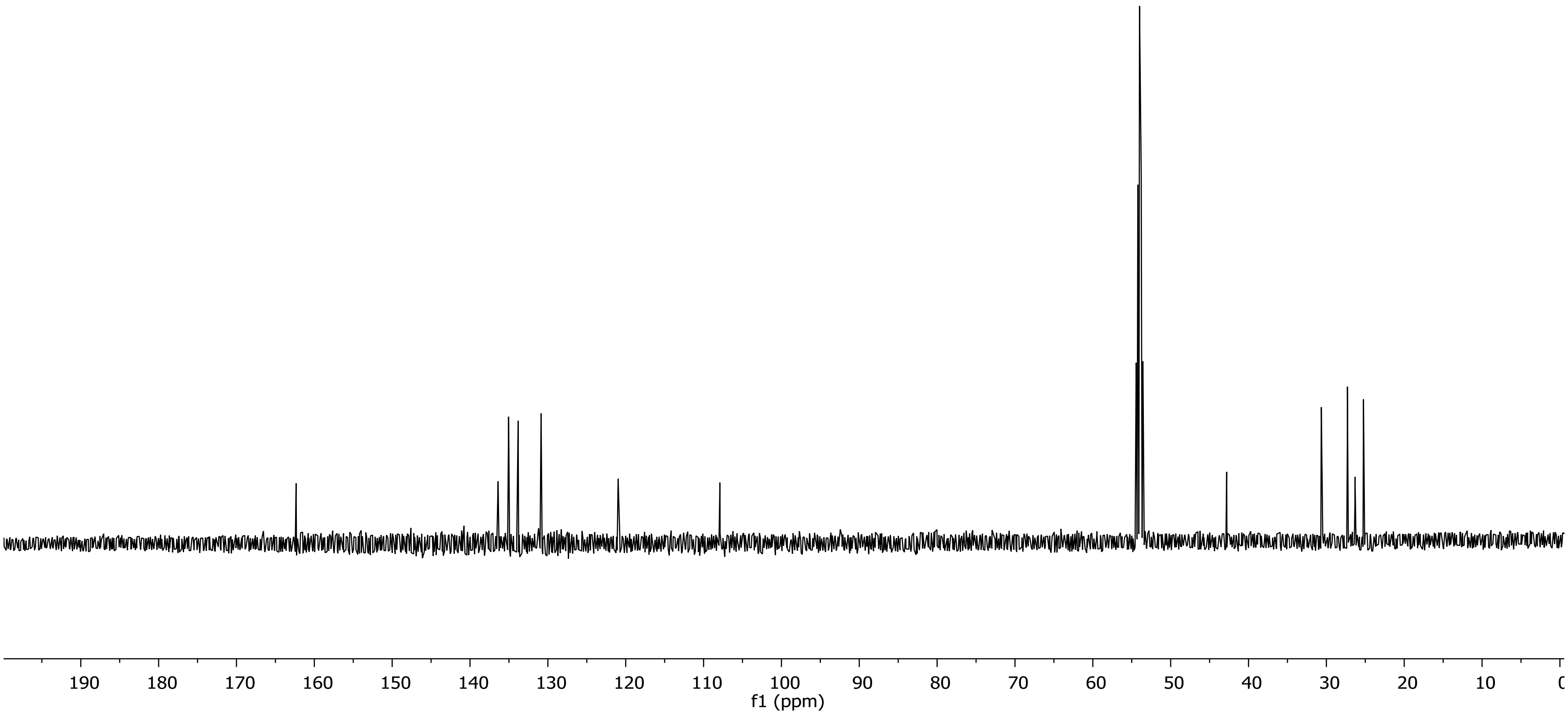

^{19}F NMR of vinylcyclooctane-derived thianthrenium salt 7-TT

CD_2Cl_2, 298 K

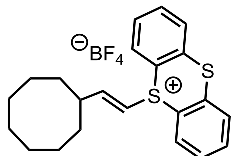

7-TT

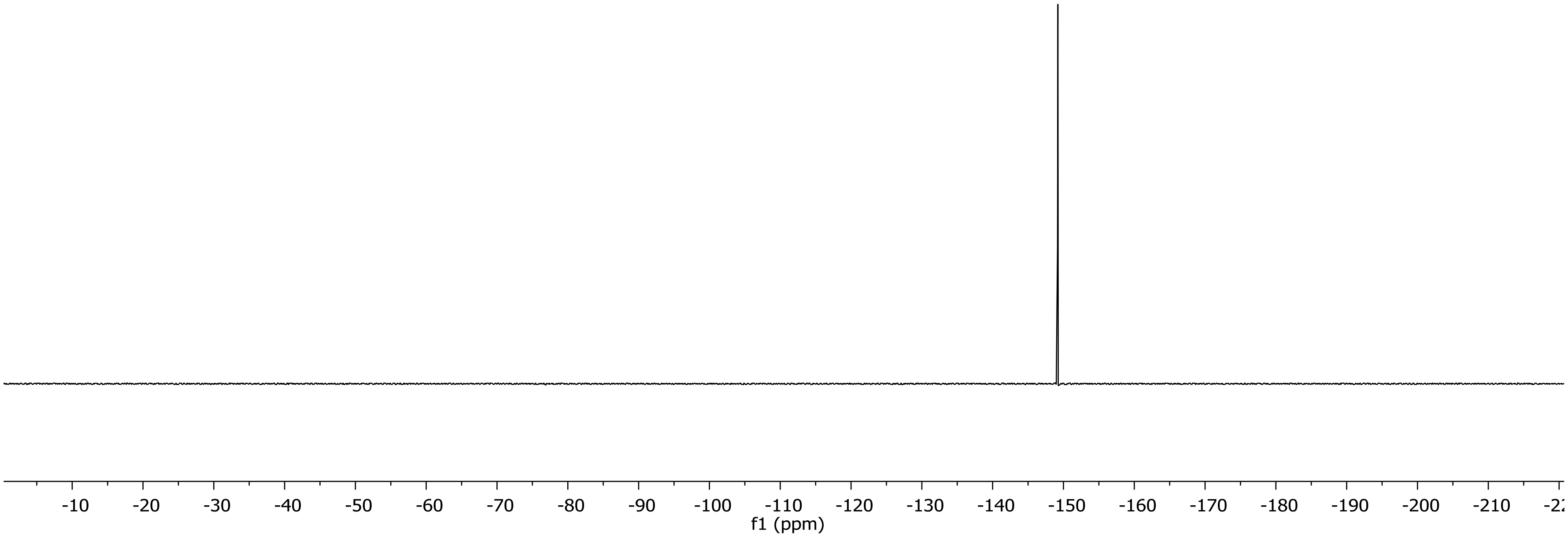

8-Brom-1-octene-derived thianthrenium salt 8-TT

^{1}H NMR of 8-brom-1-octene-derived thianthrenium salt 8-TT (*E*)

CD_2Cl_2, 298 K

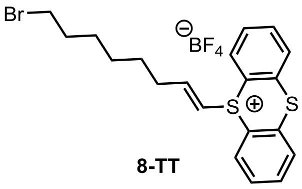

8-TT

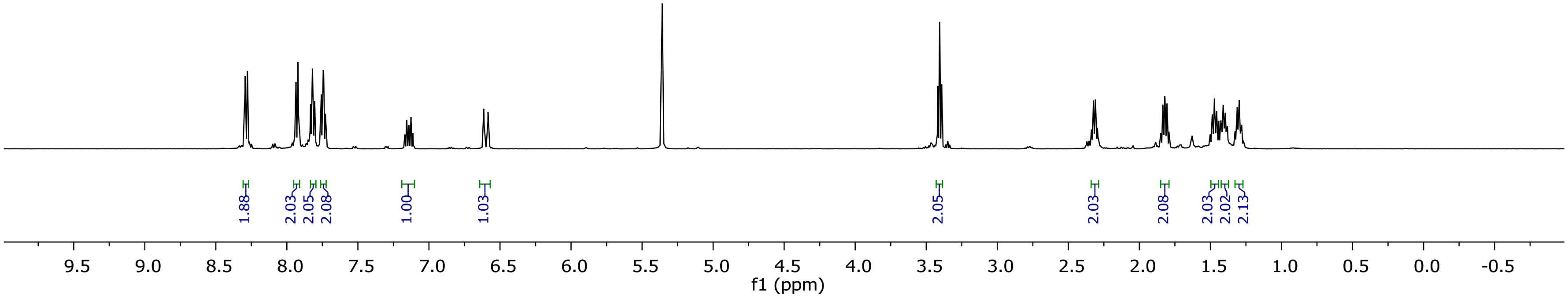

^{13}C NMR of 8-brom-1-octene-derived thianthrenium salt 8-TT

CD_2Cl_2, 298 K

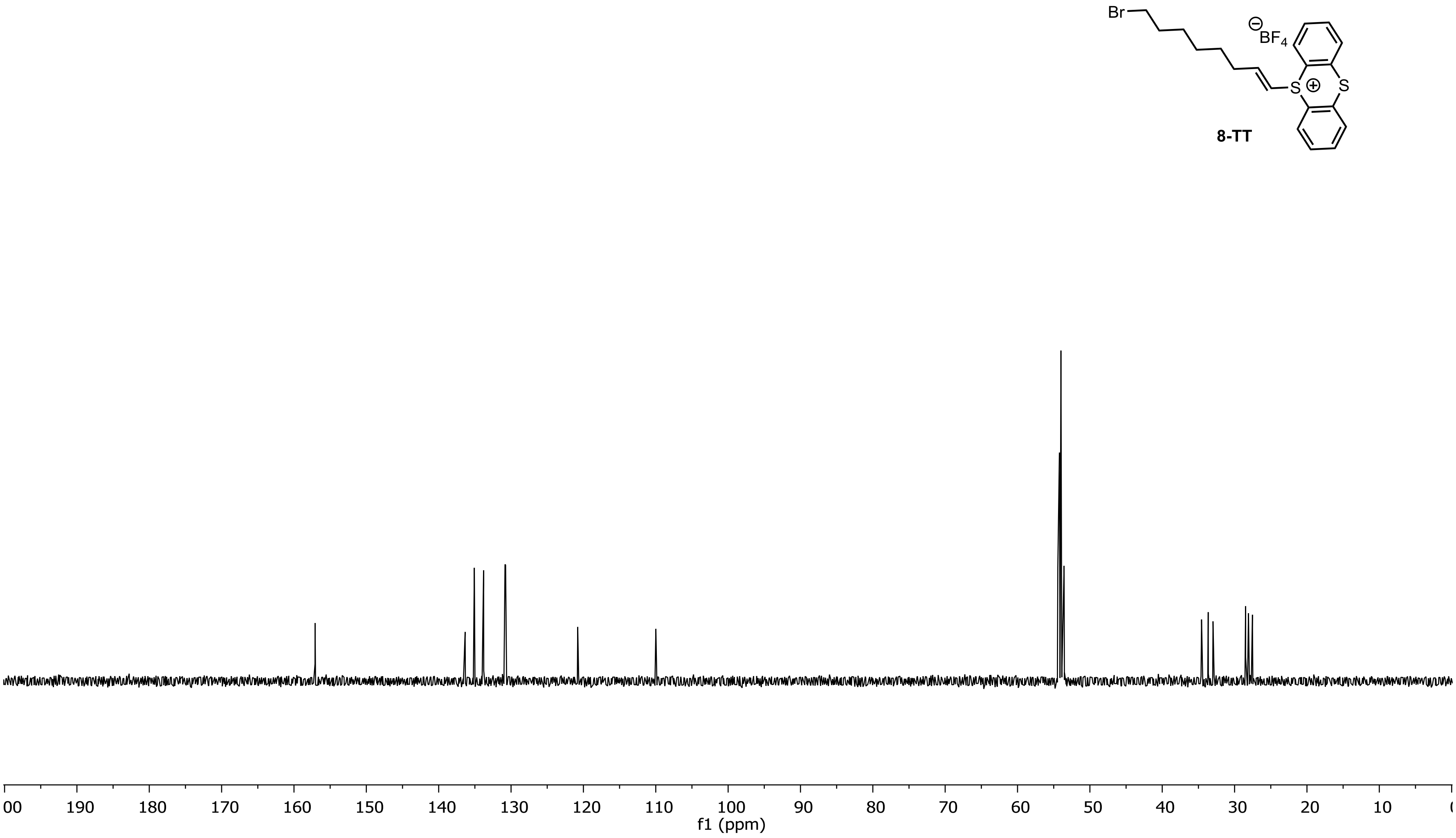

^{19}F NMR of 8-brom-1-octene-derived thianthrenium salt 8-TT

CD_2Cl_2, 298 K

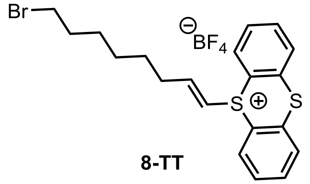

8-TT

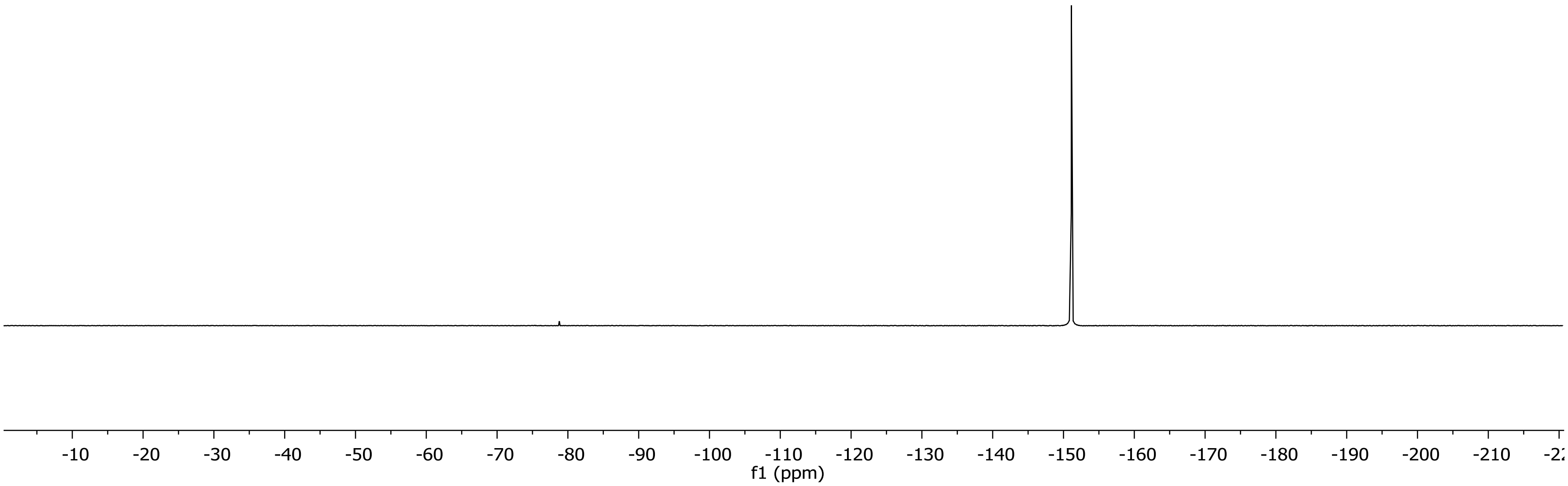

10-Undecen-1-ol-derived thianthrenium salts 9-TT(OTFA)

^{1}H NMR of 10-undecen-1-ol-derived thianthrenium salts 9-TT(OTFA) (*E*)

CD_2Cl_2, 298 K

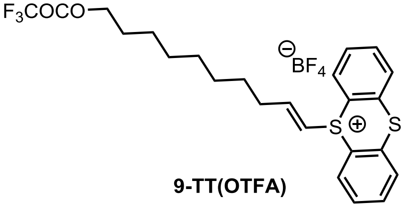

9-TT(OTFA)

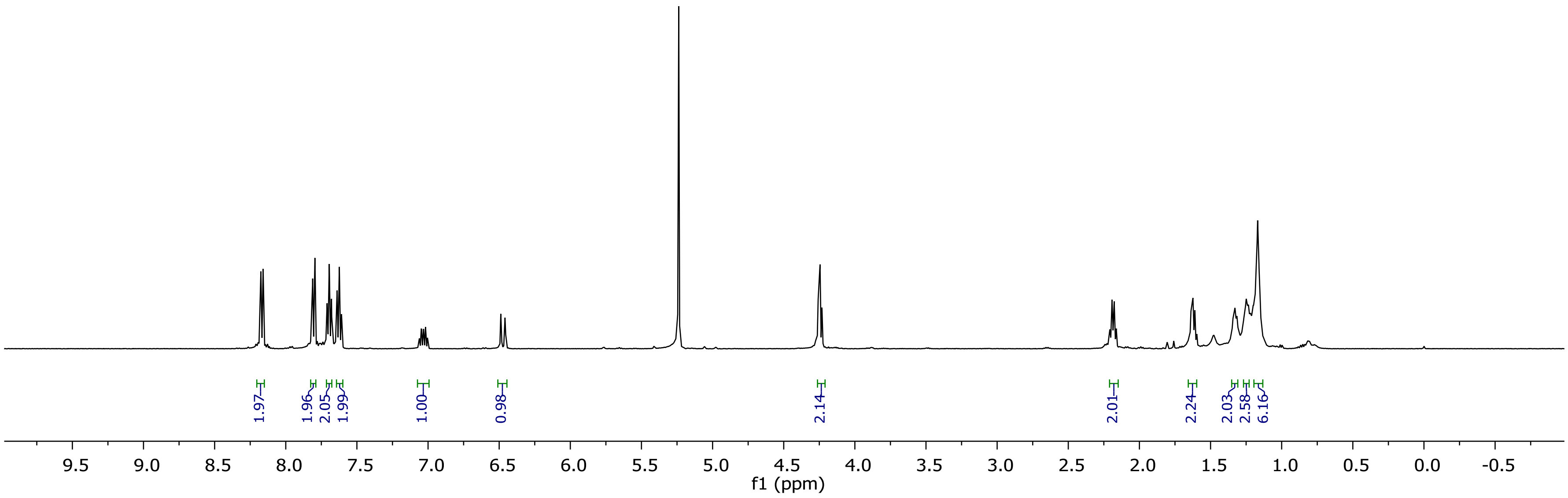

^{13}C NMR of 10-undecen-1-ol-derived thianthrenium salts 9-TT(OTFA)

CD_2Cl_2, 298 K

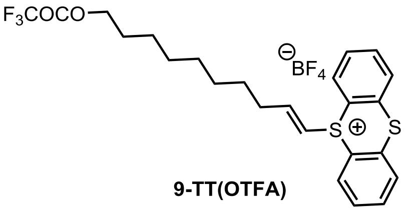

9-TT(OTFA)

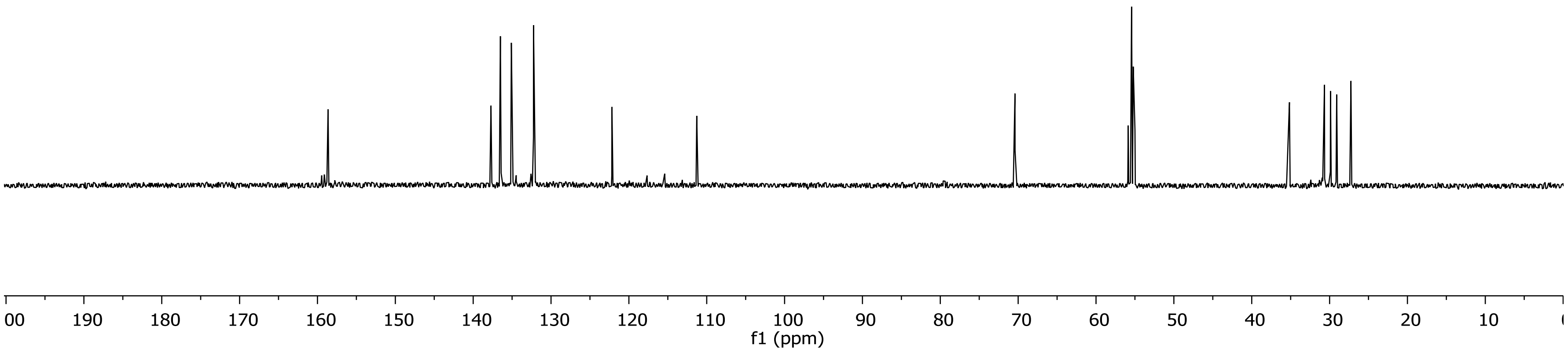

^{19}F NMR of 10-undecen-1-ol-derived thianthrenium salts 9-TT(OTFA)

CD_2Cl_2, 298 K

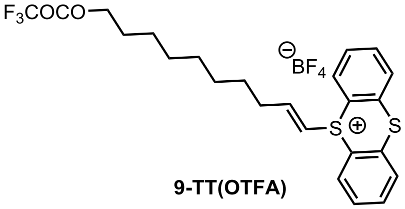

9-TT(OTFA)

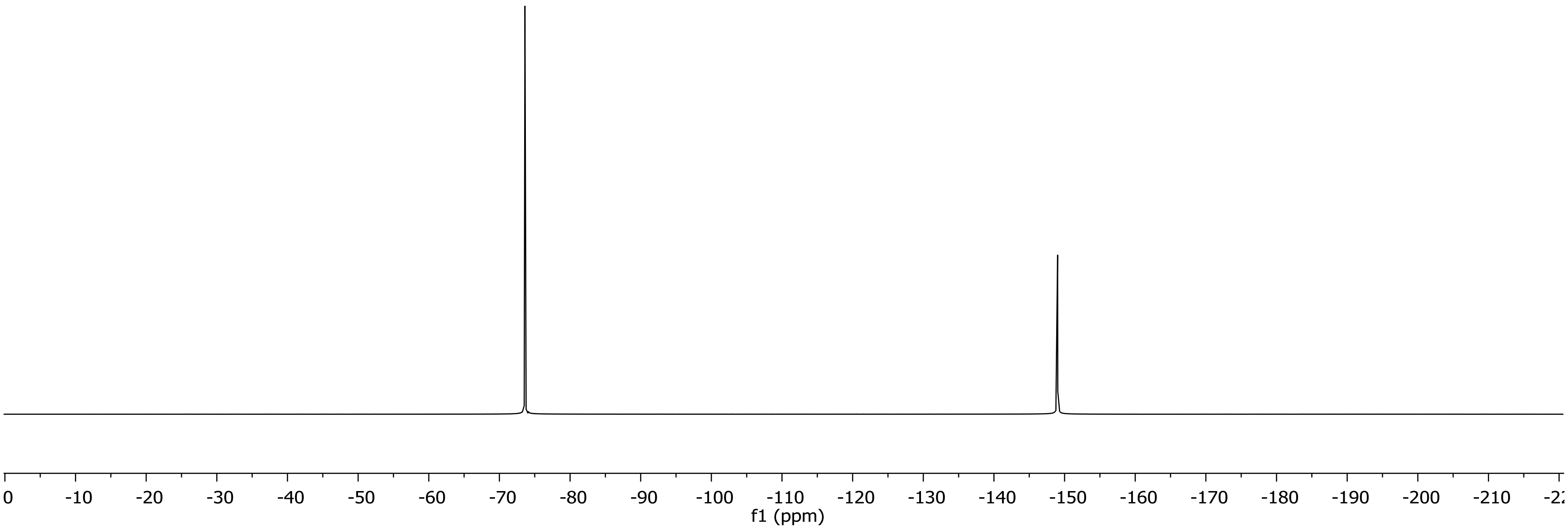

10-Undecen-1-ol-derived thianthrenium salts 9-TT(OH)

^{1}H NMR of 10-undecen-1-ol-derived thianthrenium salts 9-TT(OH)

CD_2Cl_2, 298 K

Mixture of *E*/*Z* isomers (*E*/*Z* ≈ 10/1)

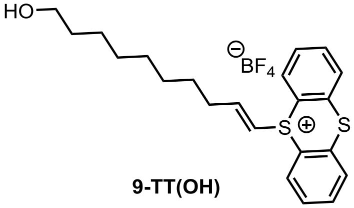

9-TT(OH)

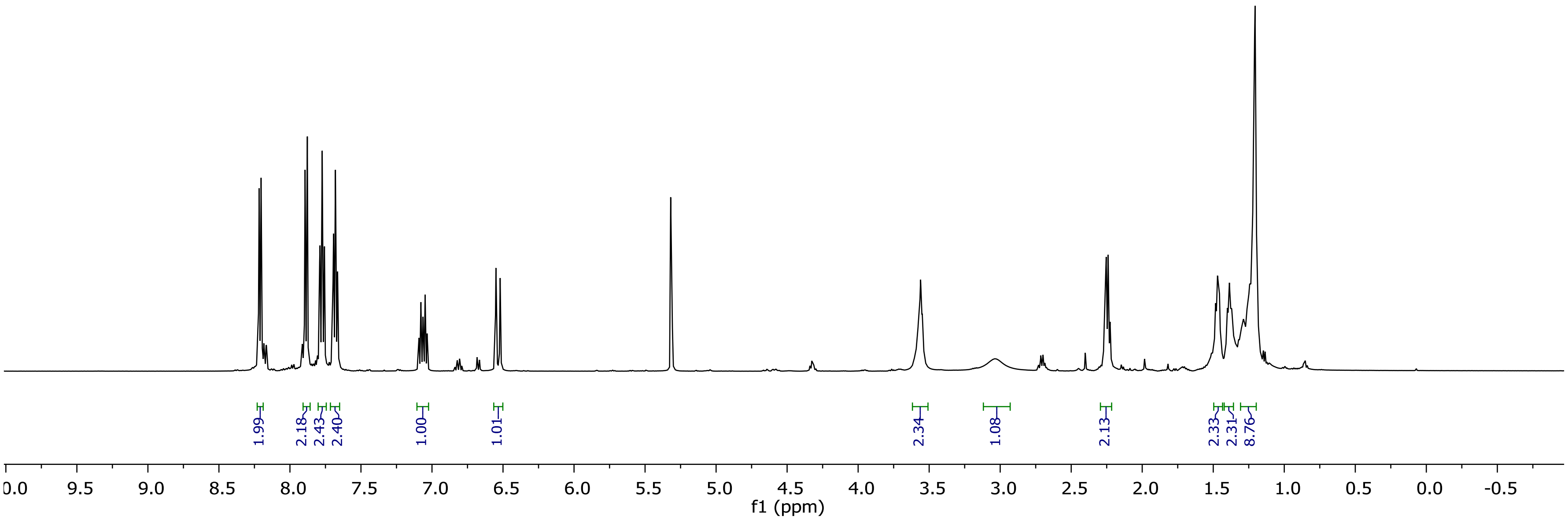

^{13}C NMR of 10-undecen-1-ol-derived thianthrenium salts 9-TT(OH)

CD_2Cl_2, 298 K

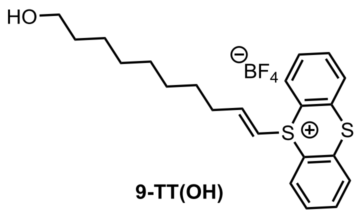

9-TT(OH)

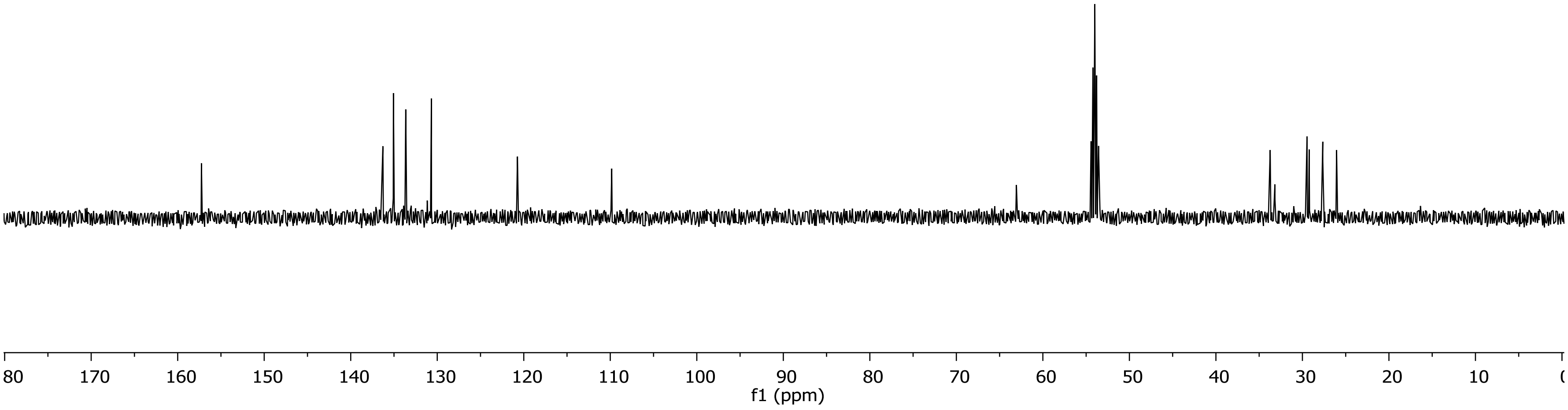

^{19}F NMR of 10-undecen-1-ol-derived thianthrenium salts 9-TT(OH)

CD_2Cl_2, 298 K

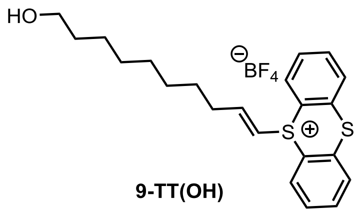

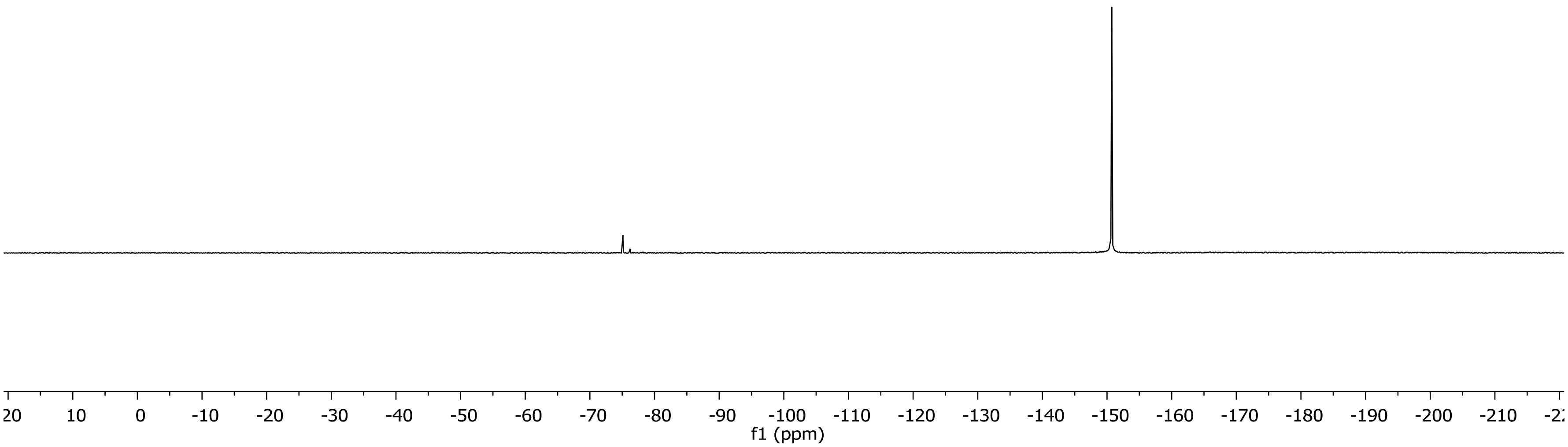

4-Phenyl-1-butene-derived thianthrenium salt 10-TT

^{1}H NMR of 4-phenyl-1-butene-derived thianthrenium salt 10-TT

CD_2Cl_2, 298 K

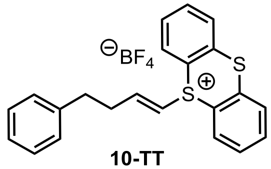

10-TT

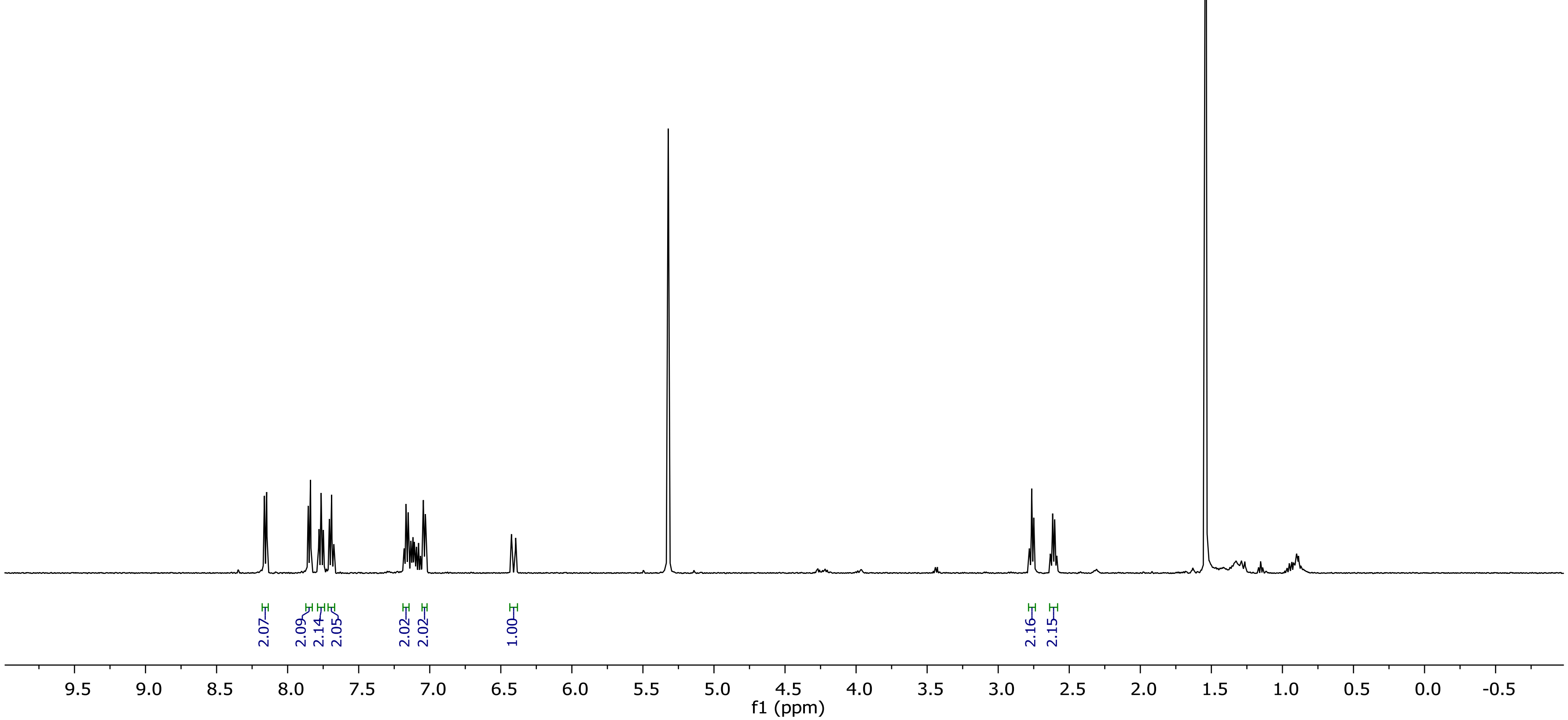

^{13}C NMR of 4-phenyl-1-butene-derived thianthrenium salt 10-TT

$CDCl_3$, 298 K

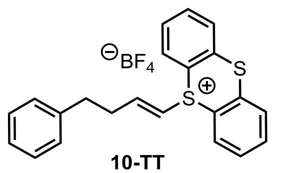

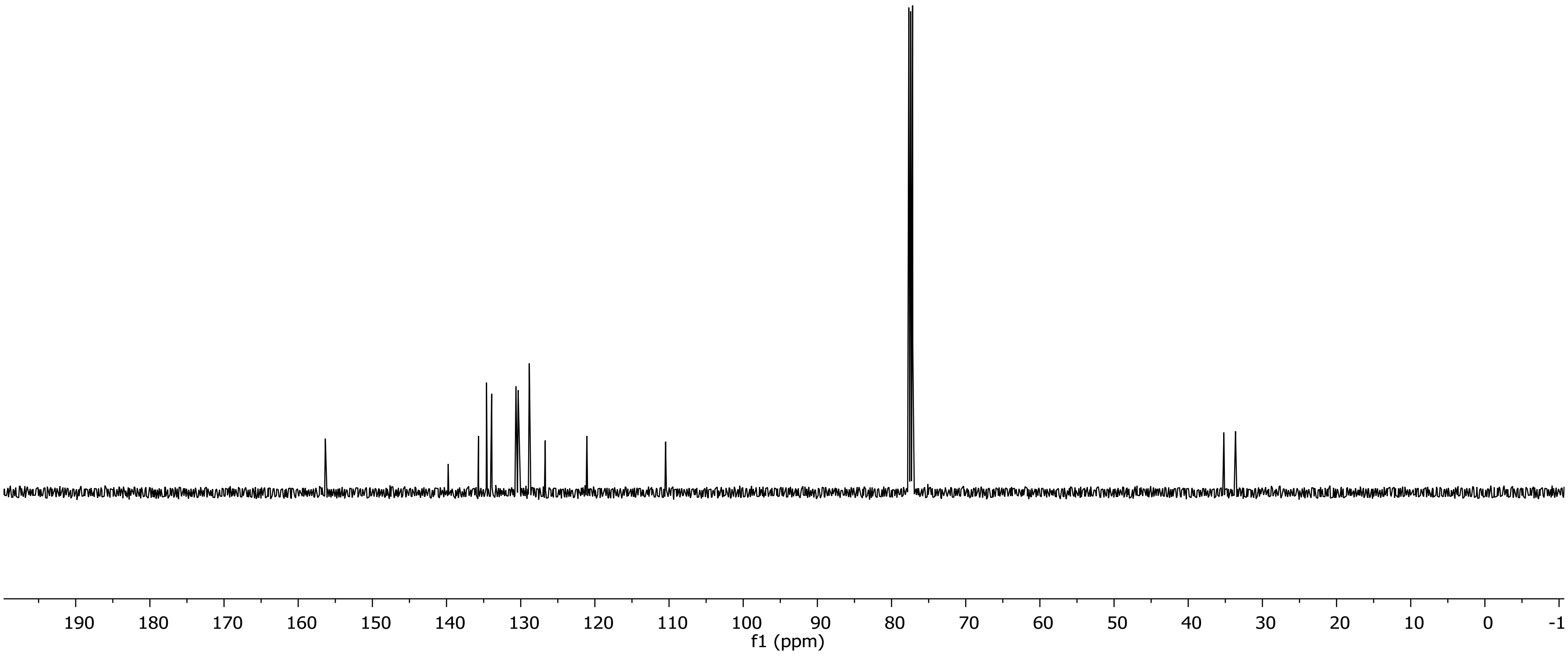

^{19}F NMR of 4-phenyl-1-butene-derived thianthrenium salt 10-TT

CDCl$_3$, 298 K

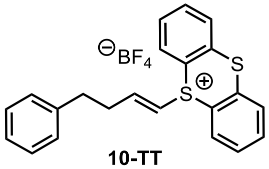

10-TT

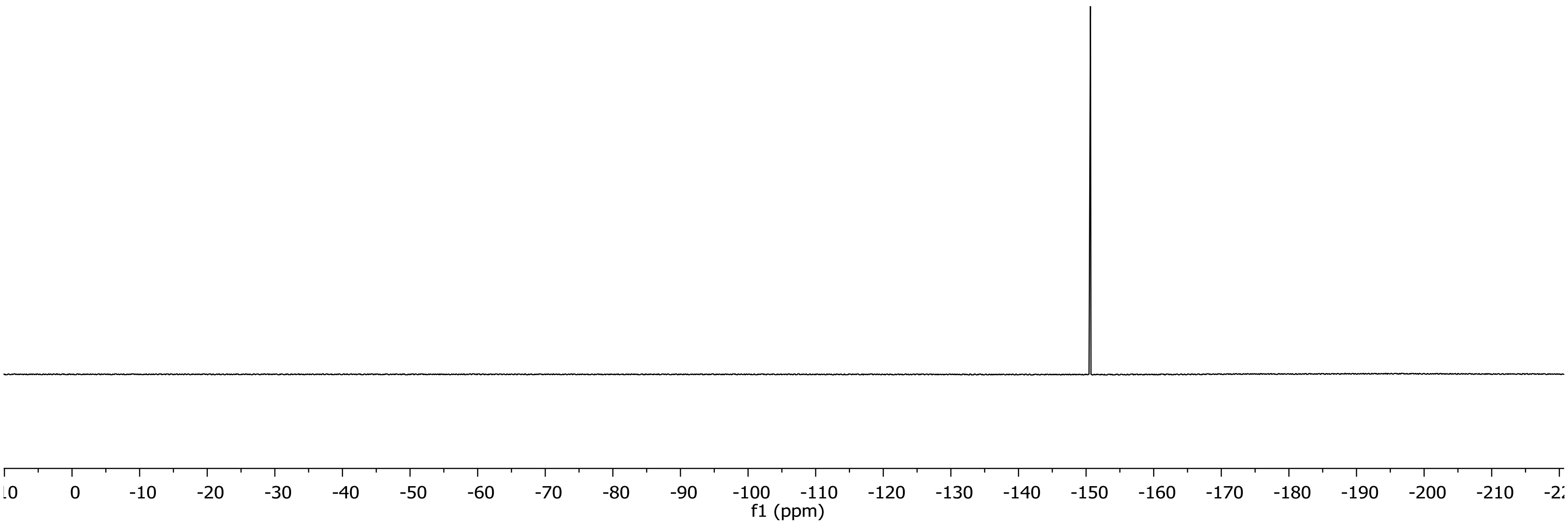

Pent-4-en-1-yl-phthalimide-derived thianthrenium salt 11-TT

^{1}H NMR of pent-4-en-1-yl-phthalimide-derived thianthrenium salt 11-TT

CD_2Cl_2, 298 K

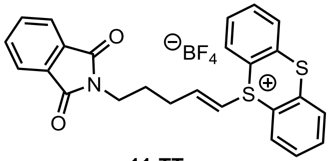

11-TT

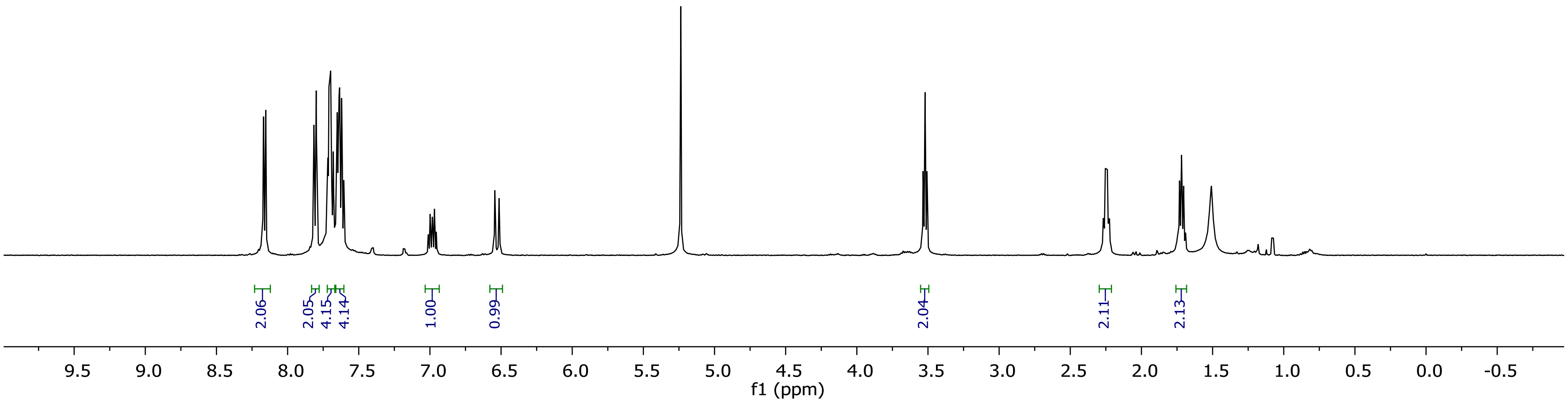

^{13}C NMR of pent-4-en-1-yl-phthalimide-derived thianthrenium salt 11-TT

CD_2Cl_2, 298 K

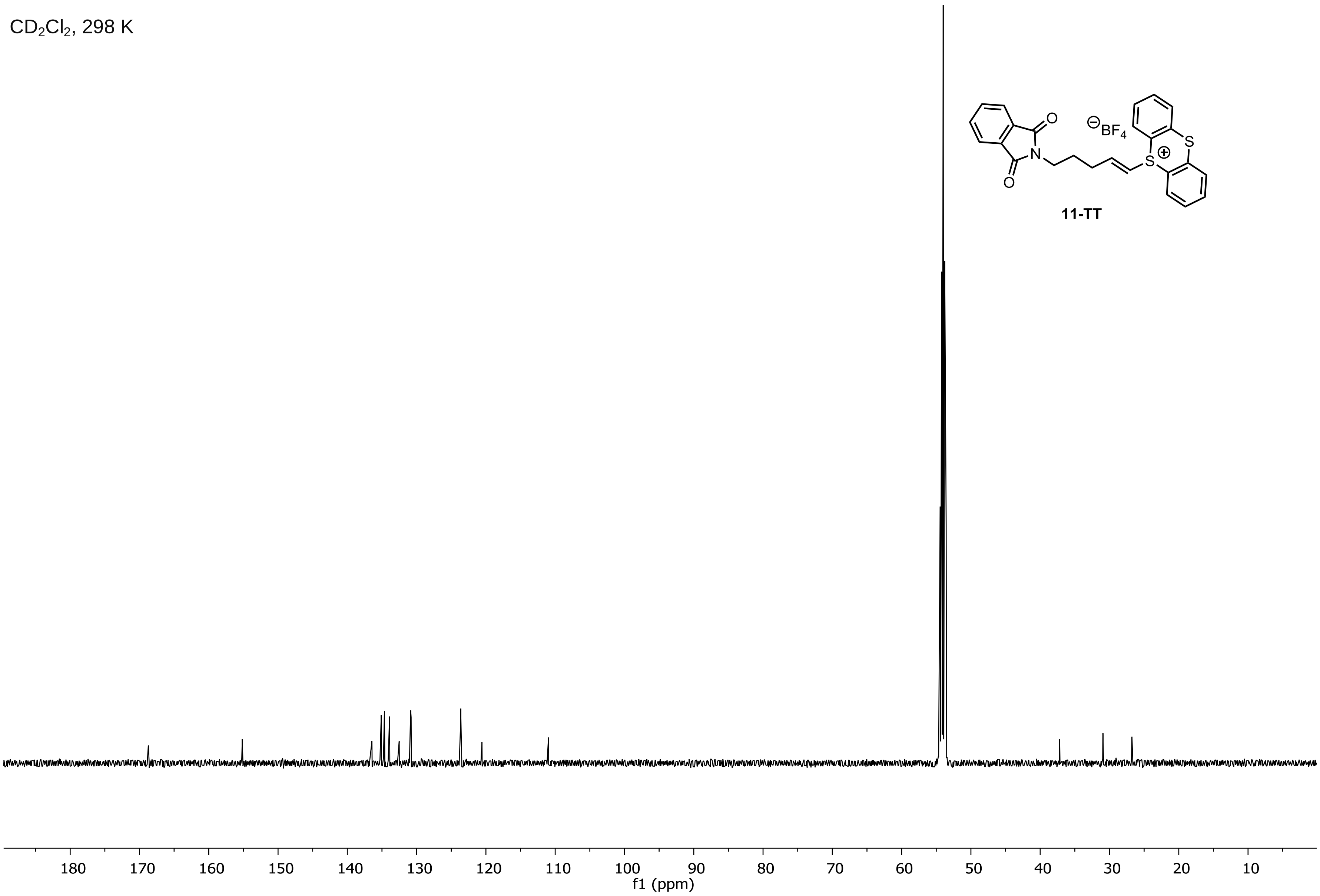

^{19}F NMR of pent-4-en-1-yl-phthalimide-derived thianthrenium salt 11-TT

CD_2Cl_2, 298 K

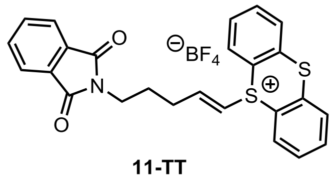

11-TT

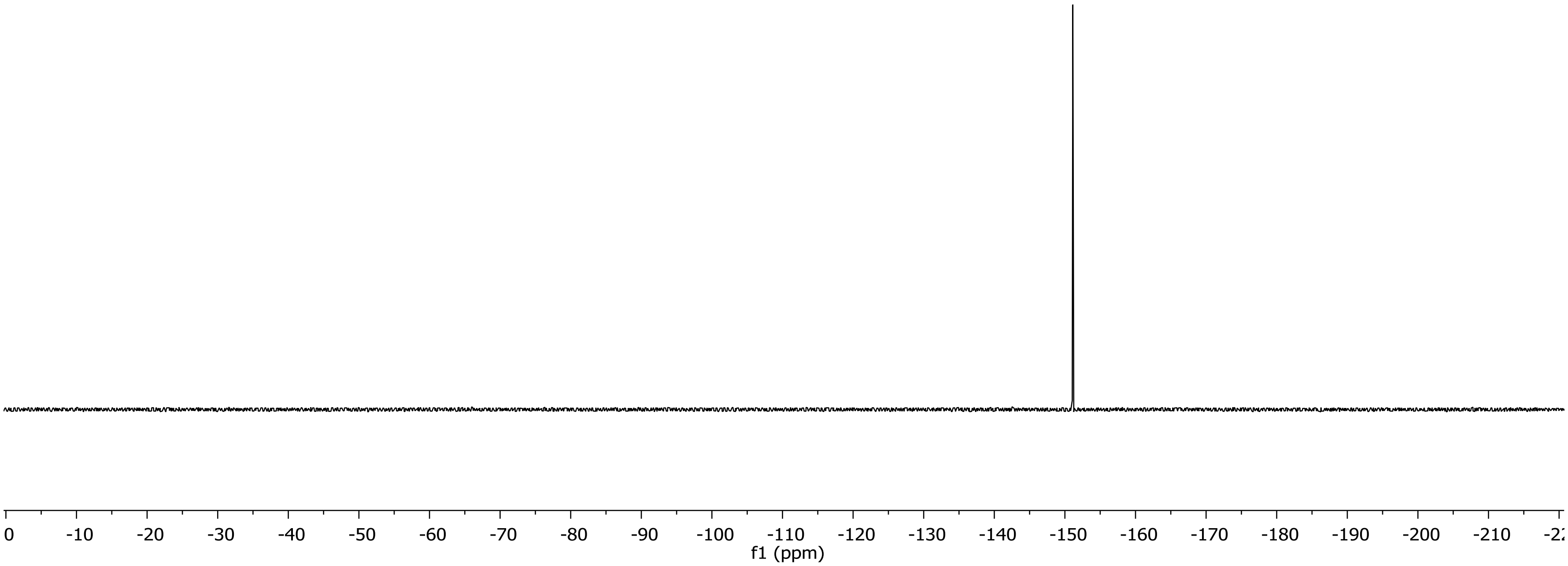

Allylbenzol-derived thianthrenium salt 12-TT

^{1}H NMR of allylbenzol-derived thianthrenium salt 12-TT

CD_2Cl_2, 298 K

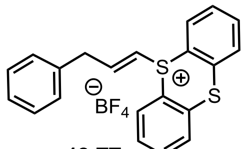

12-TT

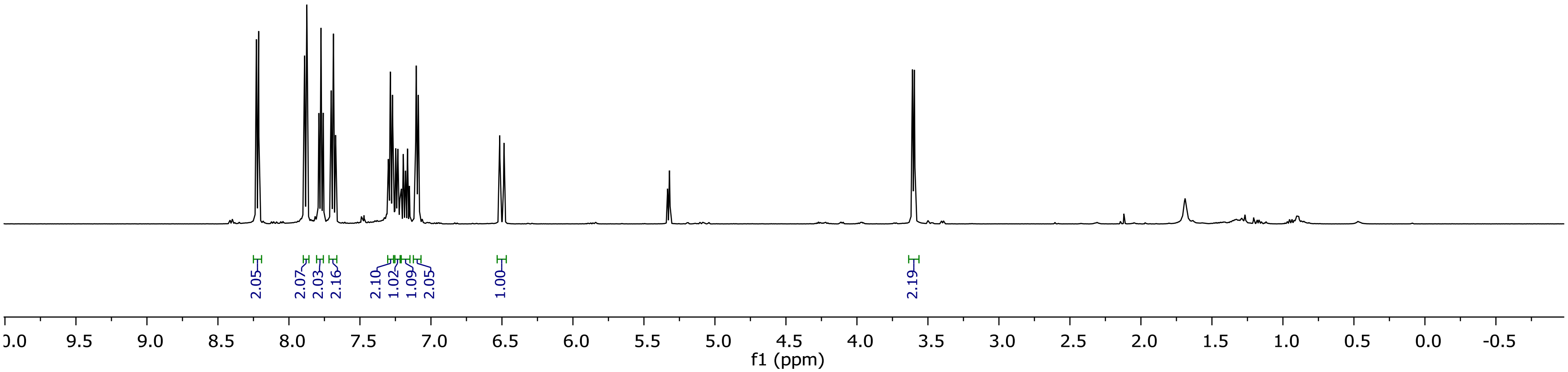

^{13}C NMR of allylbenzol-derived thianthrenium salt 12-TT

CD_2Cl_2, 298 K

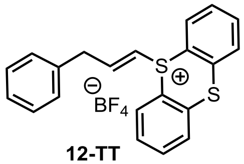

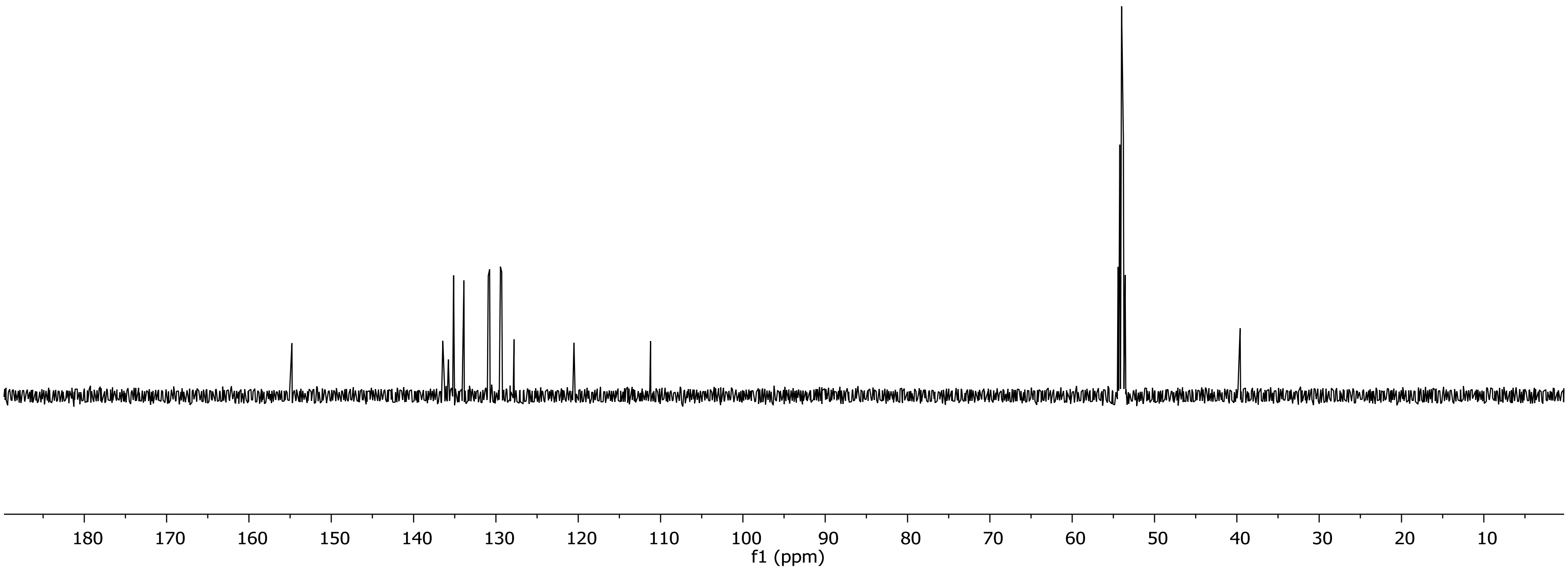

^{19}F NMR of allylbenzol-derived thianthrenium salt 12-TT

CD_2Cl_2, 298 K

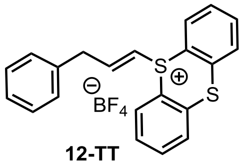

12-TT

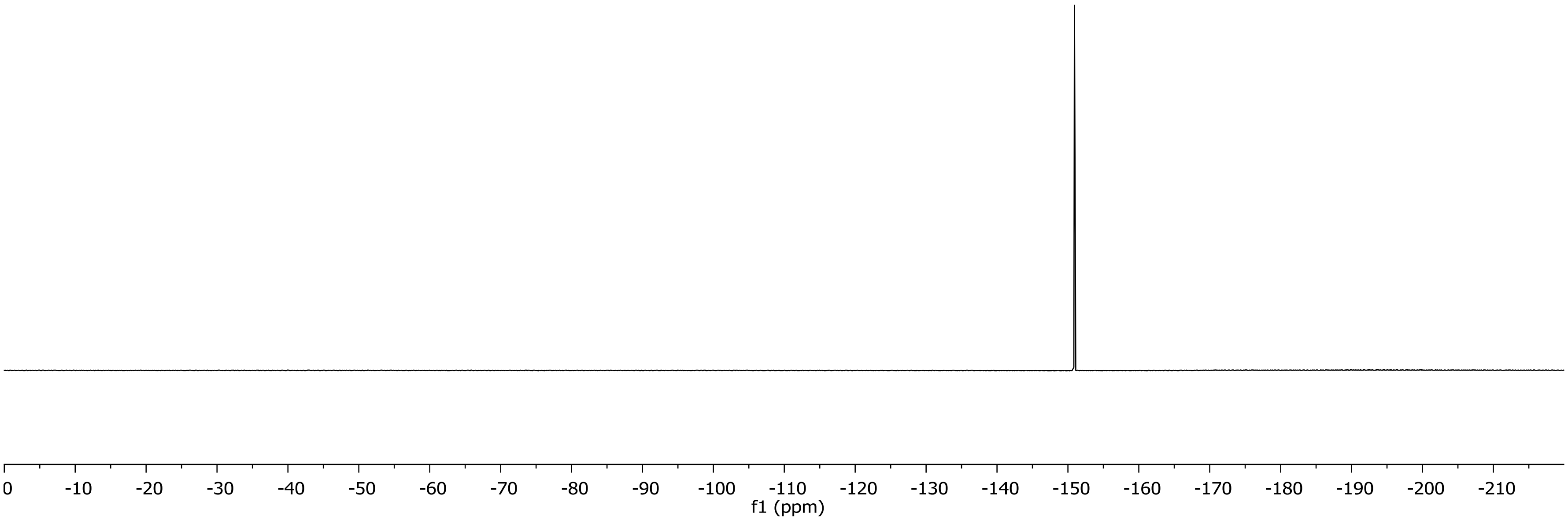

1-Allyl-4-(trifluormethyl)-benzol-derived thianthrenium salt 13-TT

^{1}H NMR of 1-allyl-4-(trifluormethyl)-benzol-derived thianthrenium salt 13-TT

CD_2Cl_2, 298 K

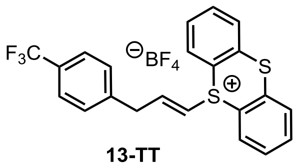

13-TT

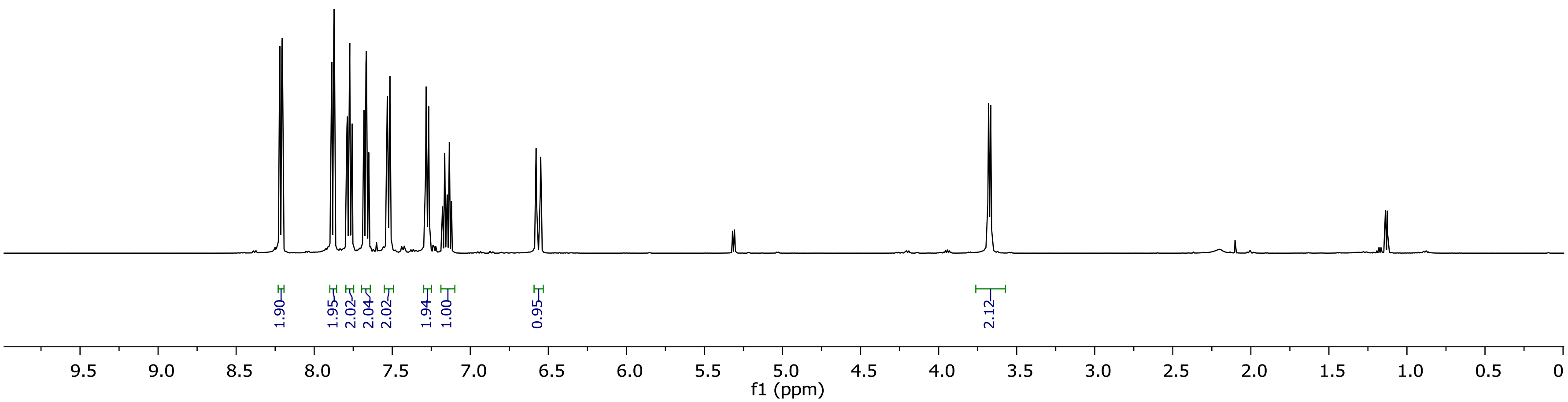

^{13}C NMR of 1-allyl-4-(trifluormethyl)-benzol-derived thianthrenium salt 13-TT

CD_2Cl_2, 298 K

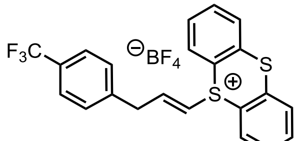

13-TT

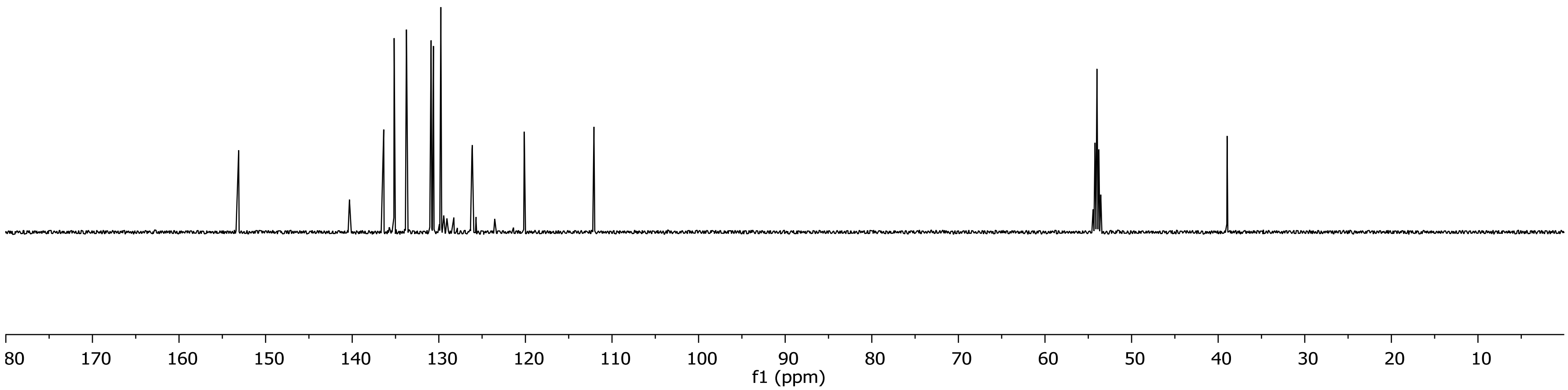

^{19}F NMR of 1-allyl-4-(trifluormethyl)-benzol-derived thianthrenium salt 13-TT

CD_2Cl_2, 298 K

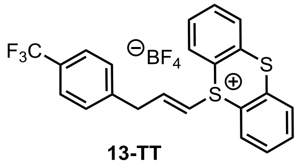

13-TT

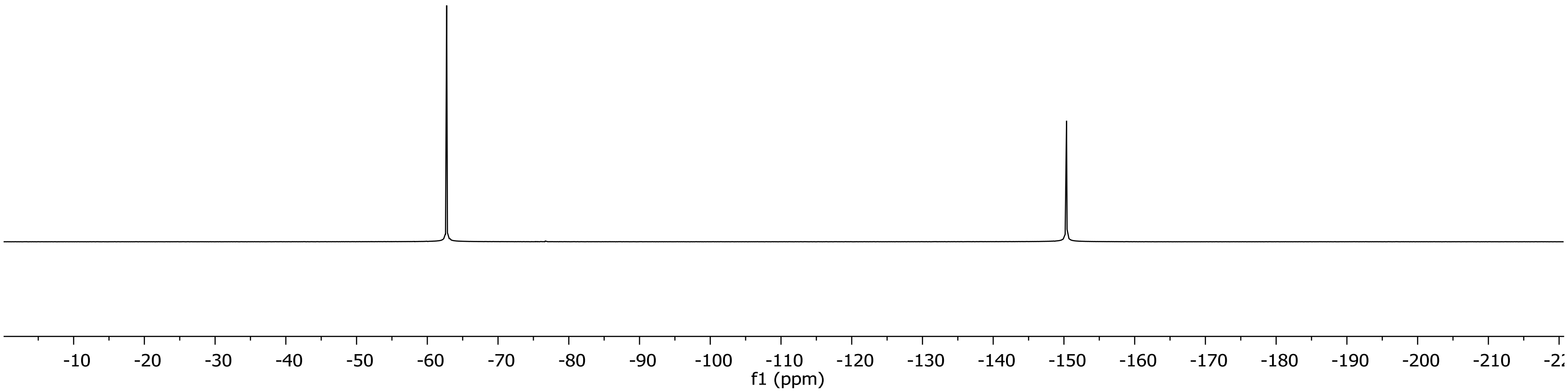

Cyclododecene-derived thianthrenium salt 14-TT

¹H NMR of cyclododecene-derived thianthrenium salt 14-TT

CD_2Cl_2, 298 K

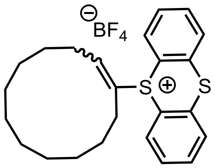

14-TT

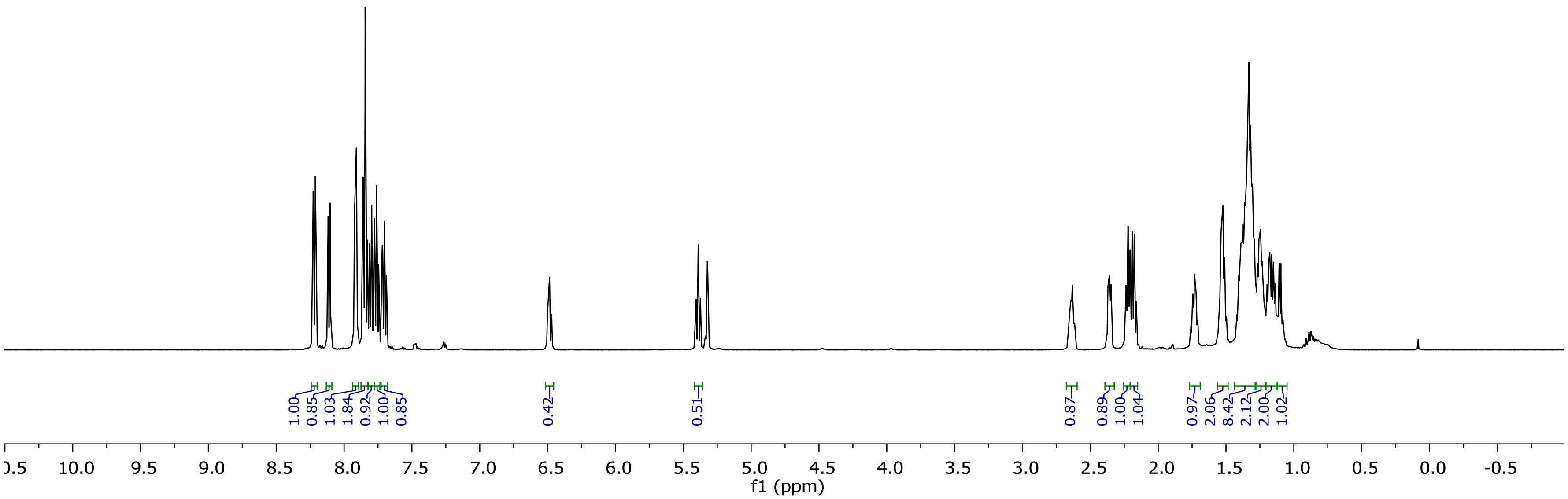

^{13}C NMR of cyclododecene-derived thianthrenium salt 14-TT

CD_2Cl_2, 298 K

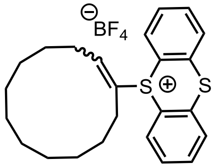

14-TT

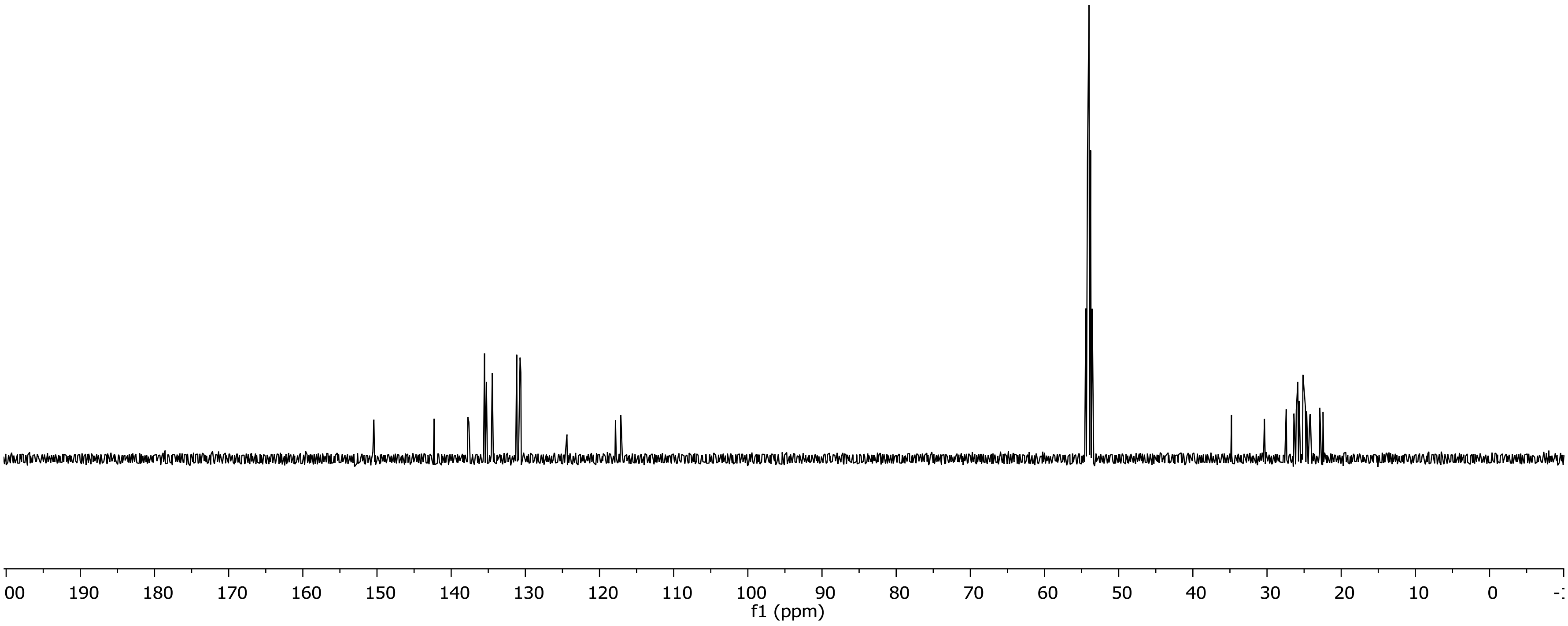

^{19}F NMR of cyclododecene-derived thianthrenium salt 14-TT

CD_2Cl_2, 298 K

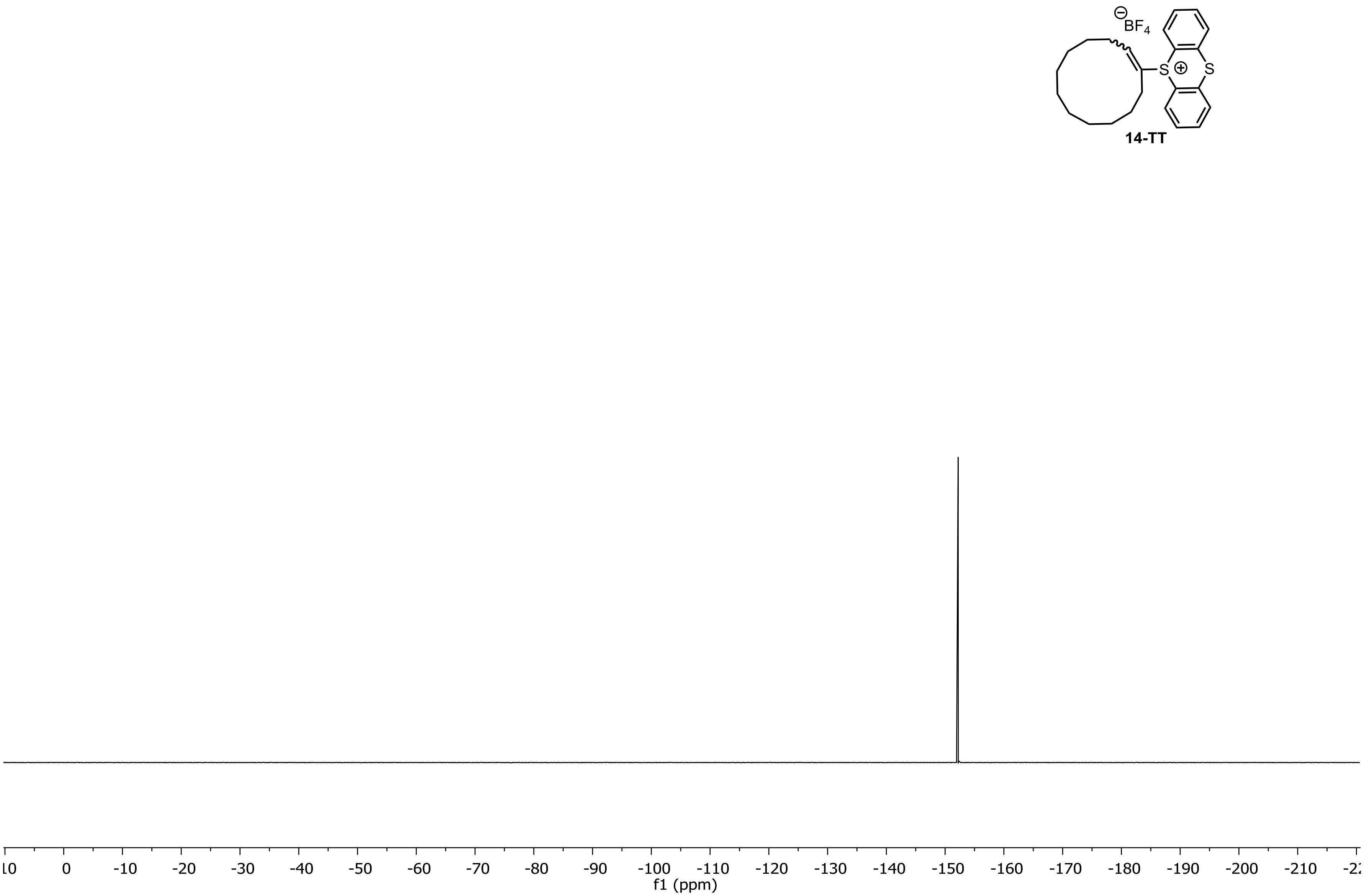

(+)-Rosenoxide-derived thianthrenium salt 15-TT

^{1}H NMR of (+)-rosenoxide-derived thianthrenium salt 15-TT

$CDCl_3$, 298 K

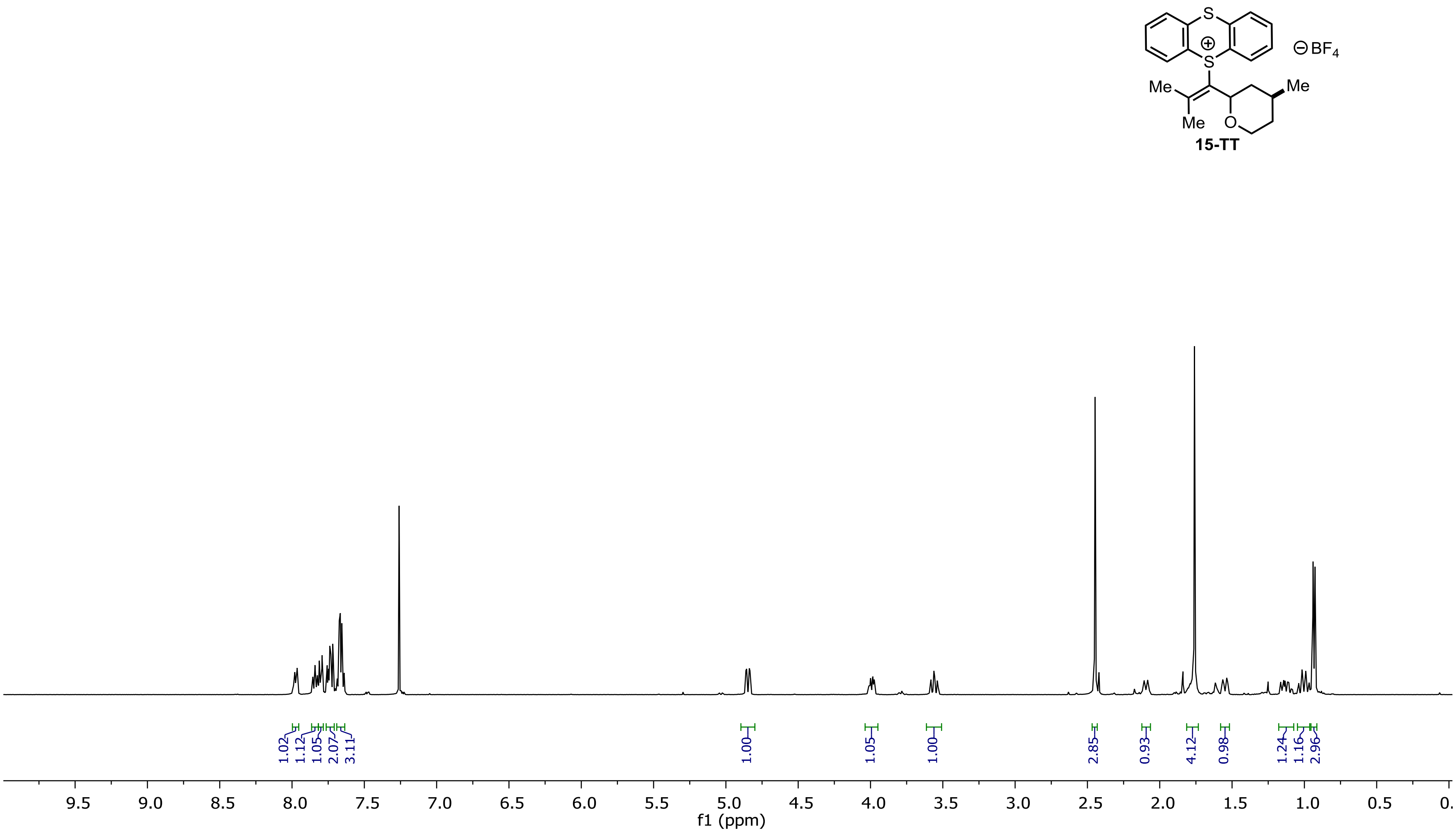

^{13}C NMR of (+)-rosenoxide-derived thianthrenium salt 15-TT

CD_2Cl_2, 298 K

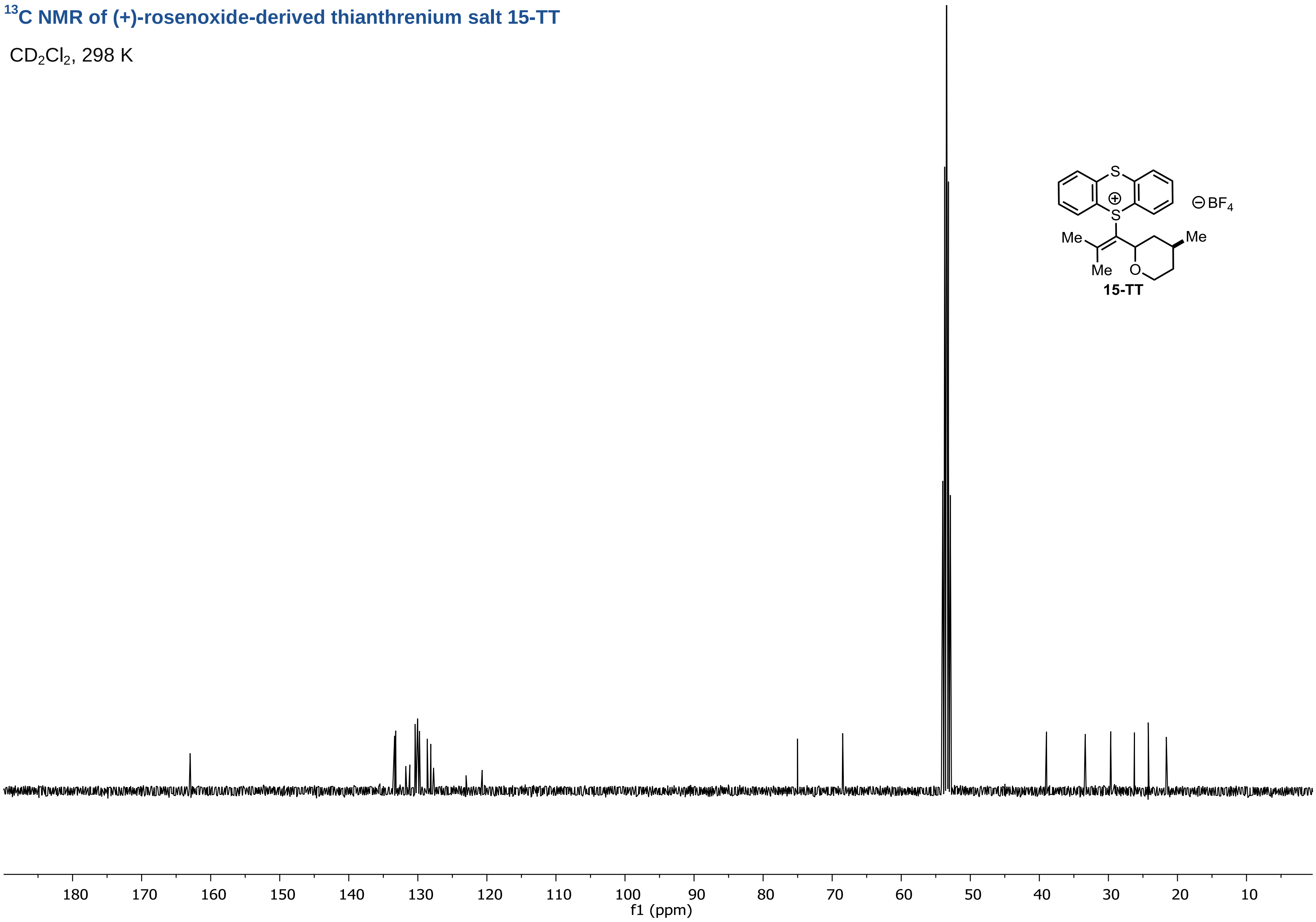

^{19}F NMR of (+)-rosenoxide-derived thianthrenium salt 15-TT

CD_2Cl_2, 298 K

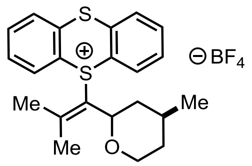

15-TT

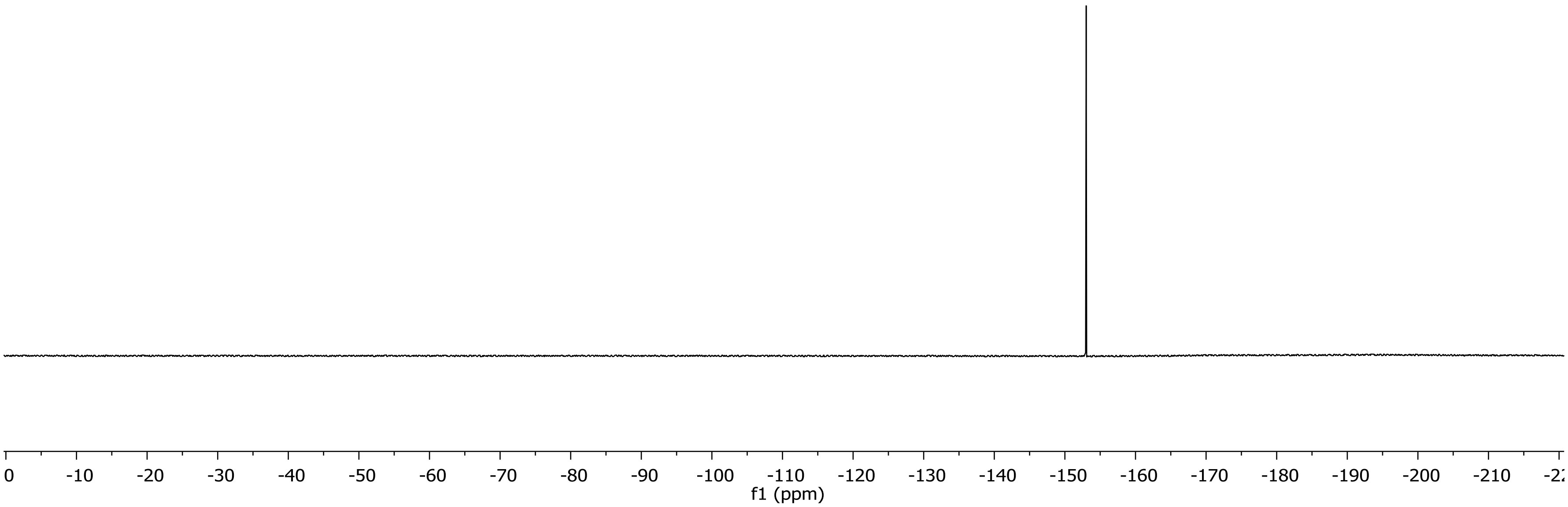

3,4-Dihydro-2*H*-pyrane-derived thianthrenium salt 16-TT

^{1}H NMR of 3,4-dihydro-2*H*-pyrane-derived thianthrenium salt 16-TT

CD_2Cl_2, 298 K

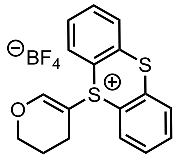

16-TT

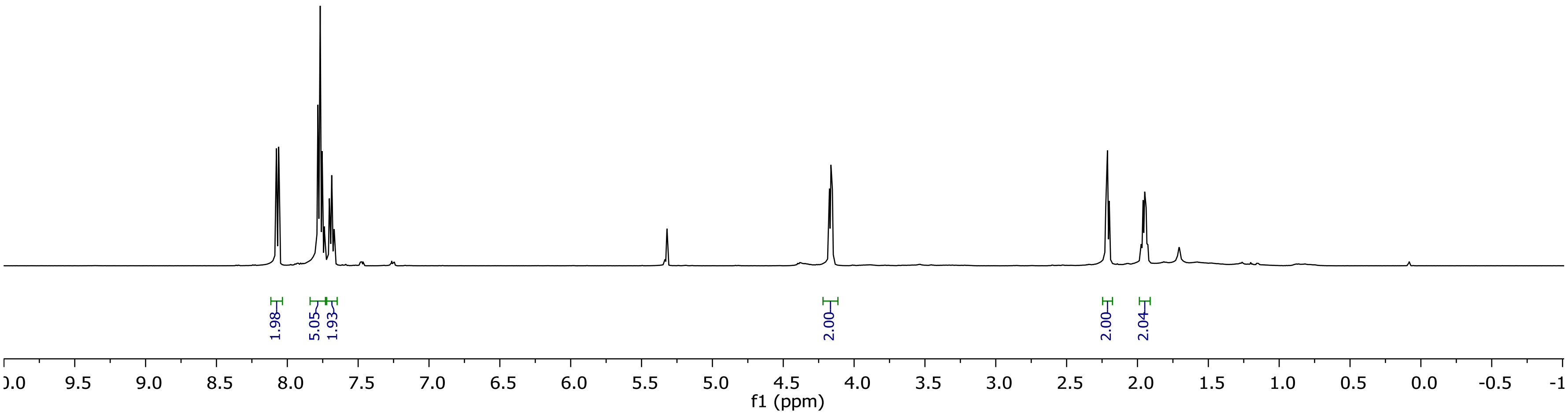

^{13}C NMR of 3,4-dihydro-2*H*-pyrane-derived thianthrenium salt 16-TT

CD_2Cl_2, 298 K

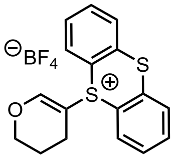

16-TT

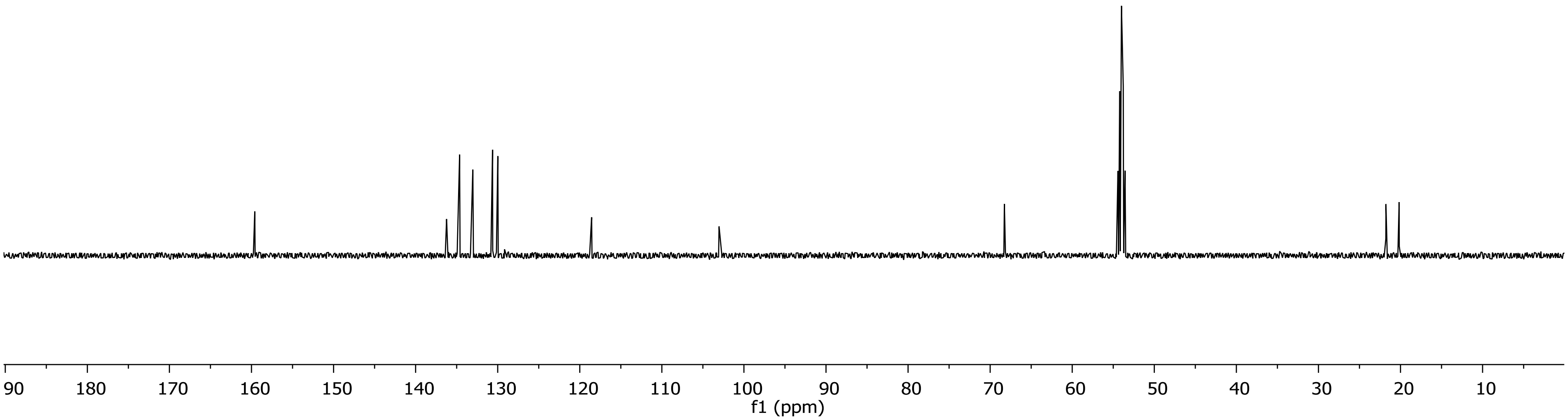

^{19}F NMR of 3,4-dihydro-2*H*-pyrane-derived thianthrenium salt 16-TT

CD_2Cl_2, 298 K

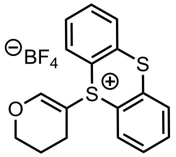

16-TT

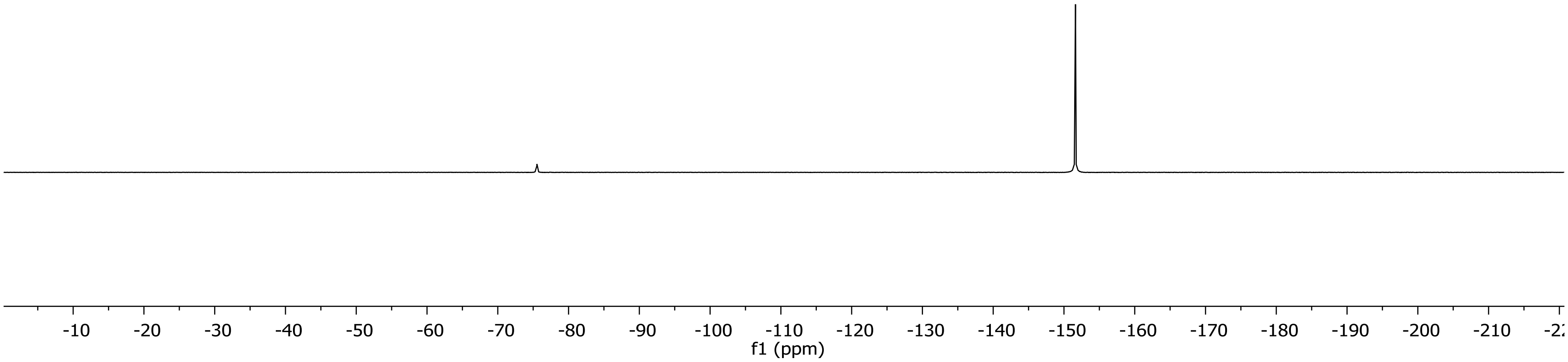

N-(But-3-en-1-yl)benzamide-derived thianthrenium salt 17-TT

^{1}H NMR of *N*-(but-3-en-1-yl)benzamide-derived thianthrenium salt 17-TT

CD_2Cl_2, 298 K

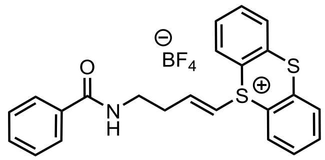

17-TT

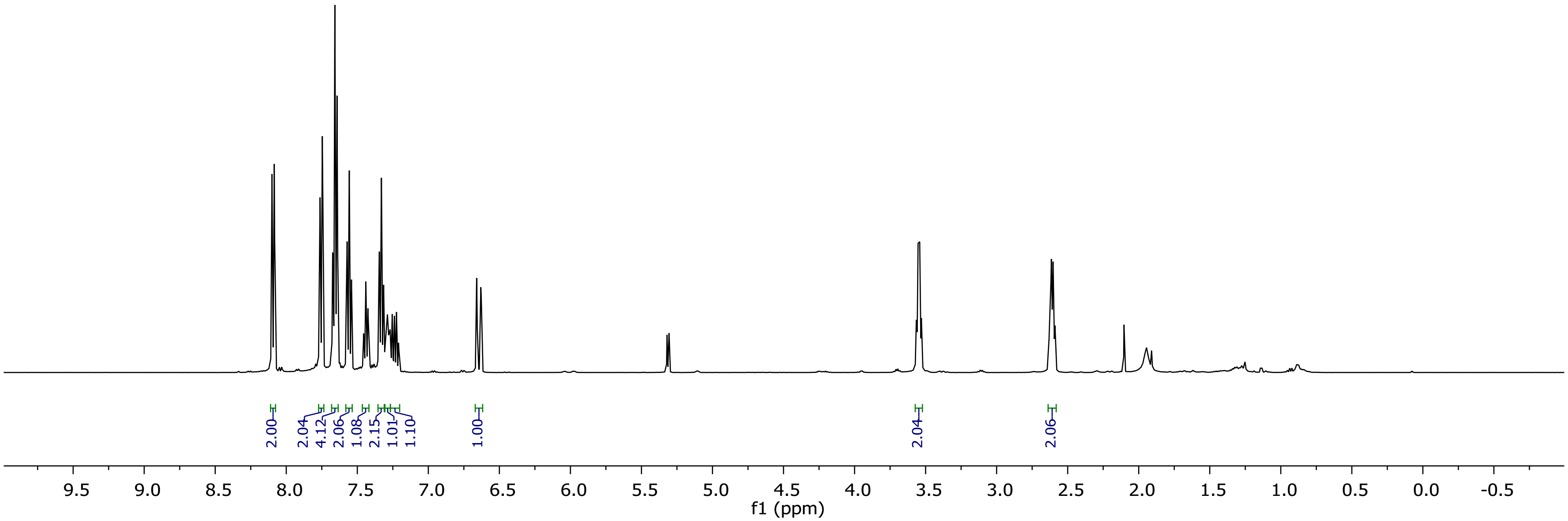

^{13}C NMR of *N*-(but-3-en-1-yl)benzamide-derived thianthrenium salt 17-TT

CD_2Cl_2, 298 K

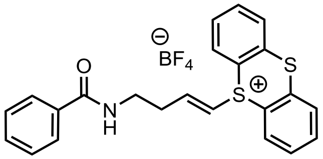

17-TT

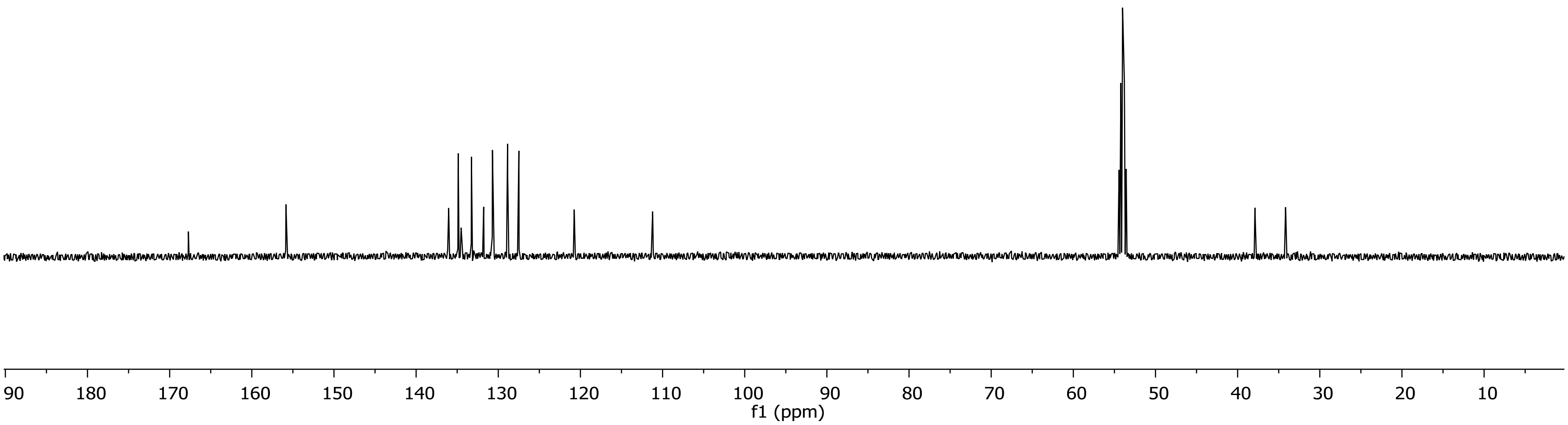

^{19}F NMR of *N*-(but-3-en-1-yl)benzamide-derived thianthrenium salt 17-TT

$CDCl_3$, 298 K

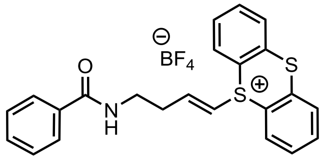

17-TT

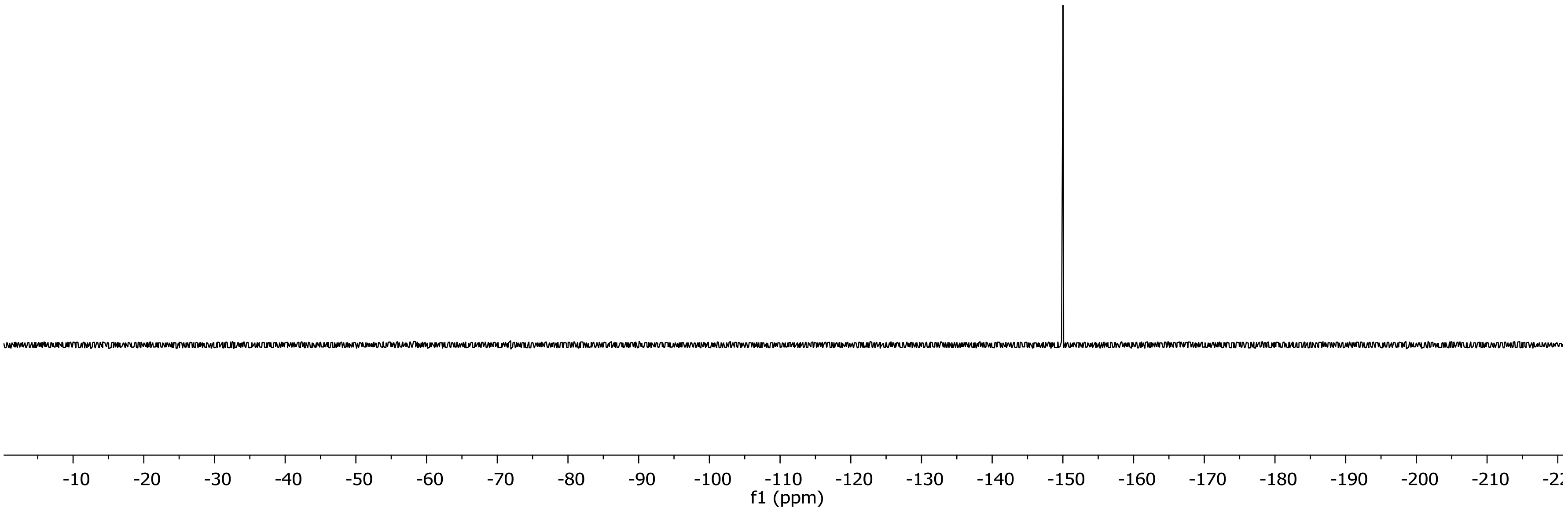

Cyclooctene-derived thianthrenium salt 18-TT

^{1}H NMR of cyclooctene-derived thianthrenium salt 18-TT

CD_2Cl_2, 298 K

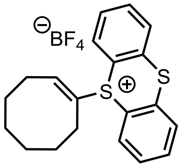

18-TT

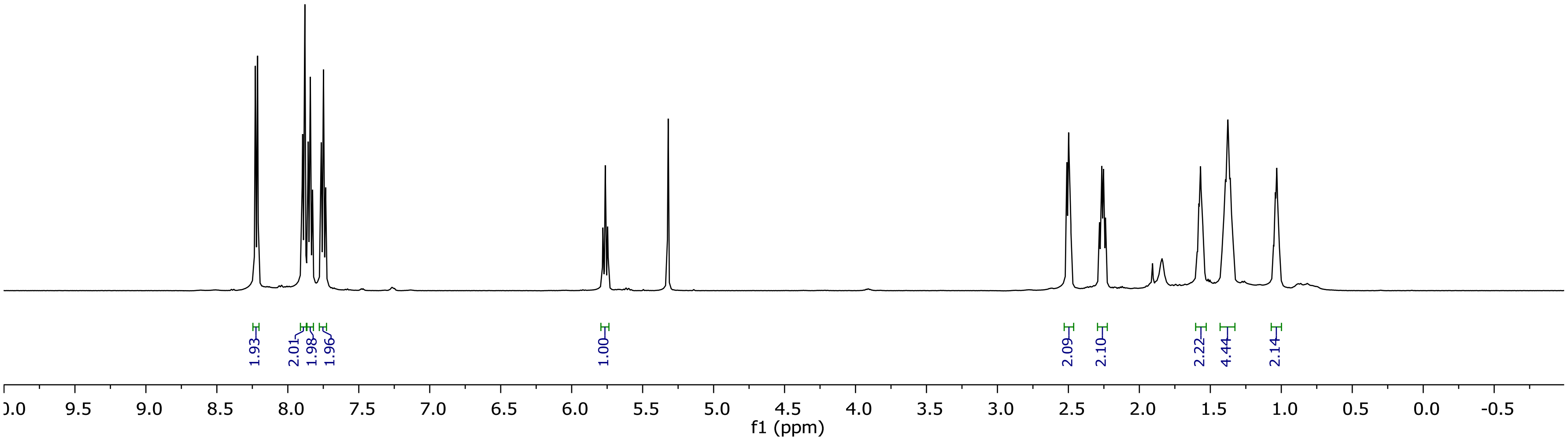

^{13}C NMR of cyclooctene-derived thianthrenium salt 18-TT

CD_2Cl_2, 298 K

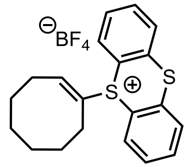

18-TT

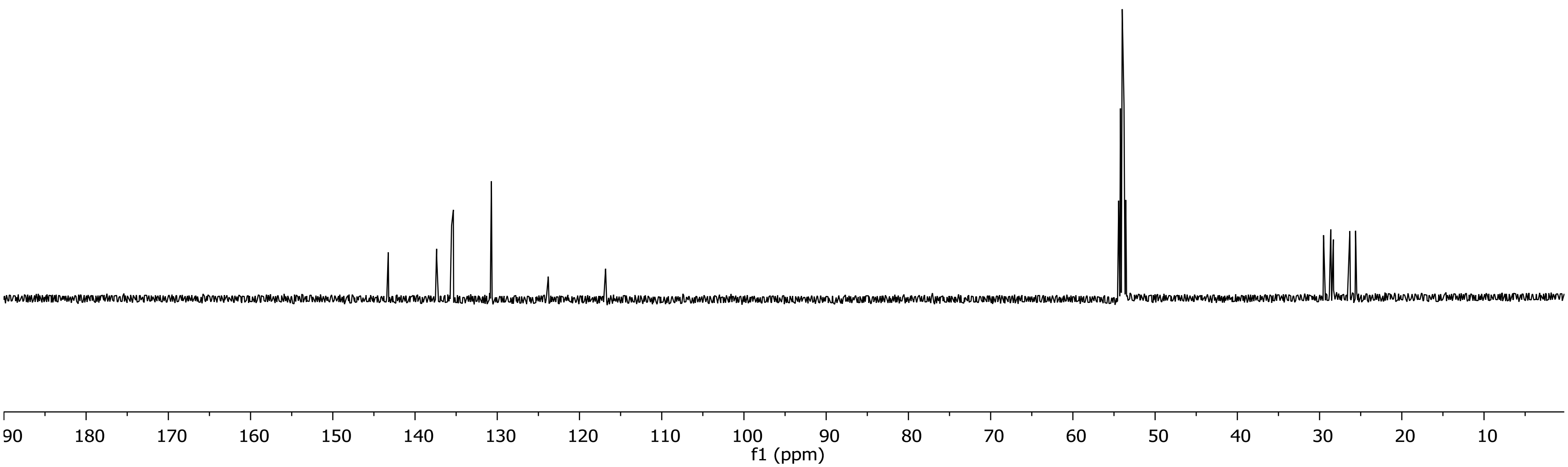

^{19}F NMR of cyclooctene-derived thianthrenium salt 18-TT

CD_2Cl_2, 298 K

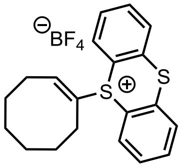

18-TT

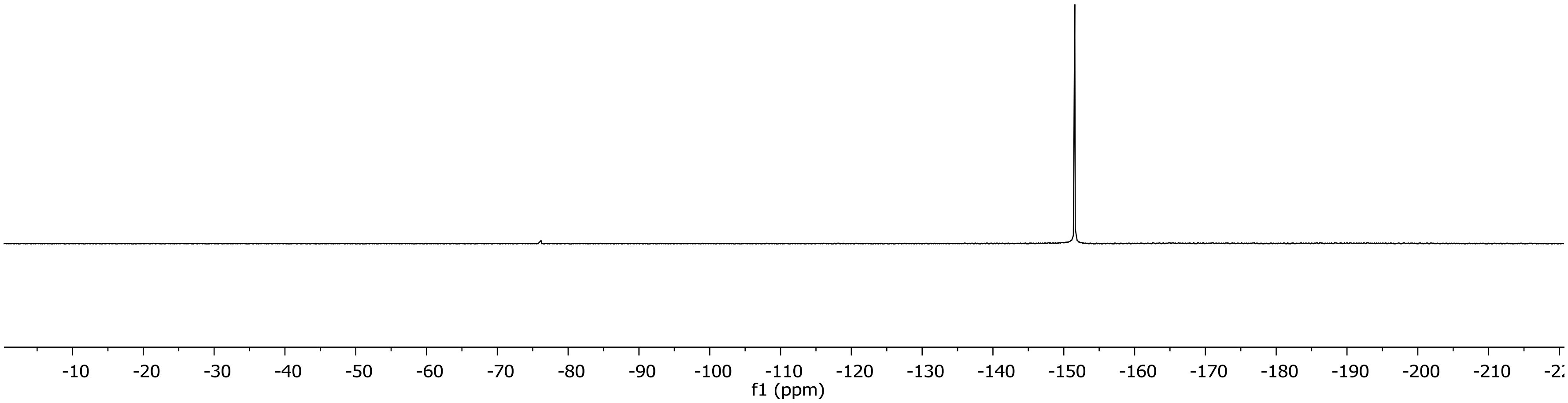

Cycloheptene-derived thianthrenium salt 19-TT

^{1}H NMR of cycloheptene-derived thianthrenium salt 19-TT

CD_2Cl_2, 298 K

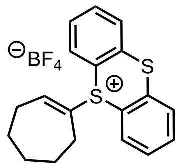

19-TT

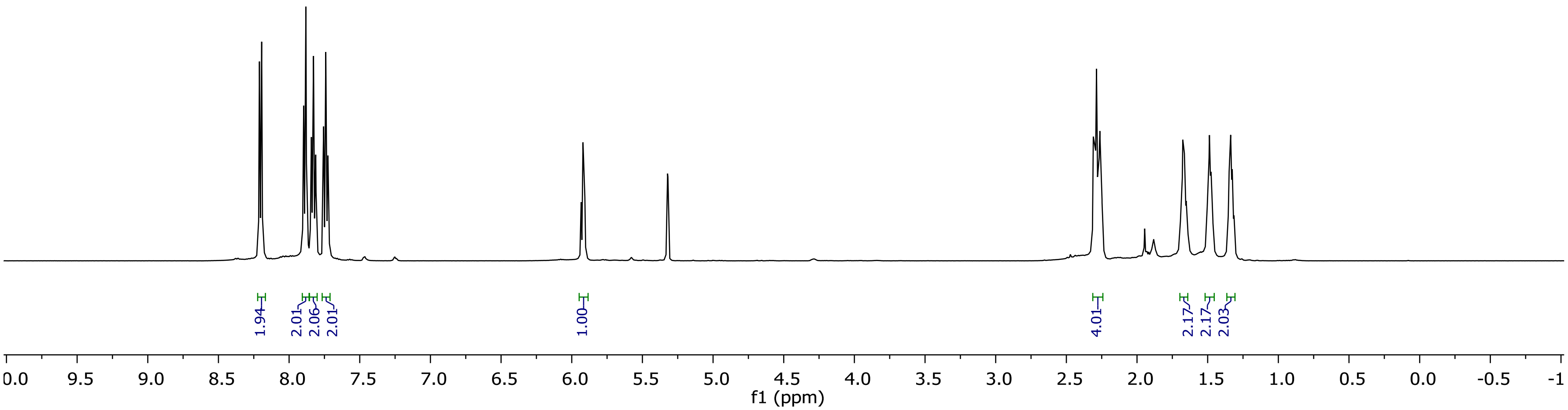

^{13}C NMR of cycloheptene-derived thianthrenium salt 19-TT

CD_2Cl_2, 298 K

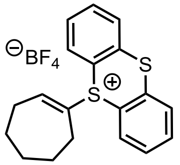

19-TT

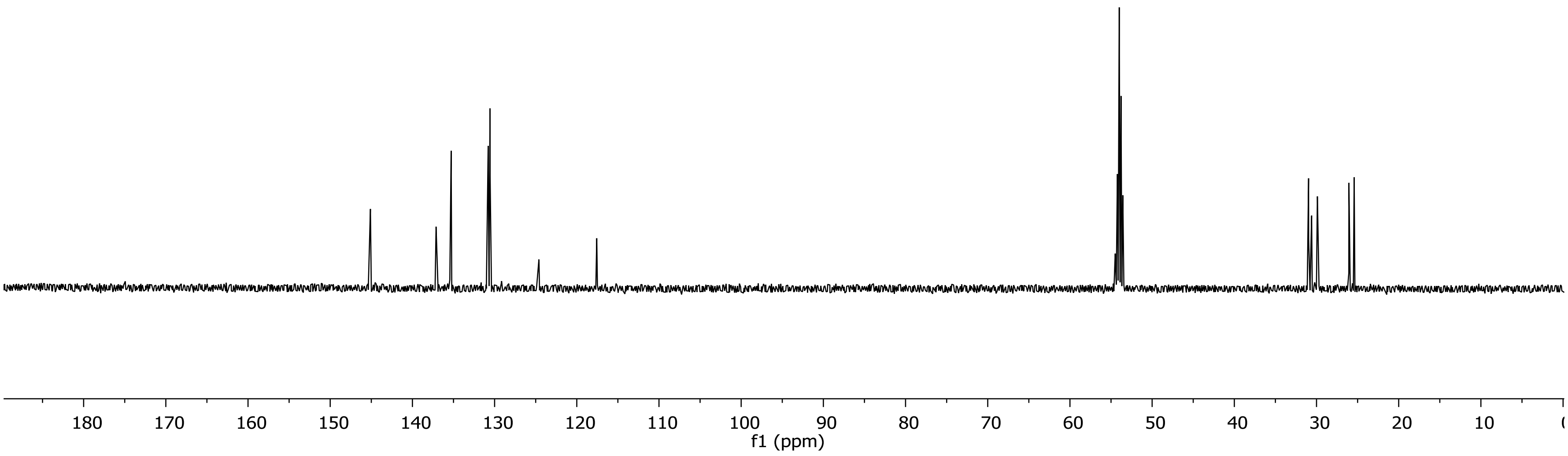

^{19}F NMR of cycloheptene-derived thianthrenium salt 19-TT

CD_2Cl_2, 298 K

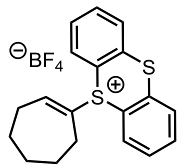

19-TT

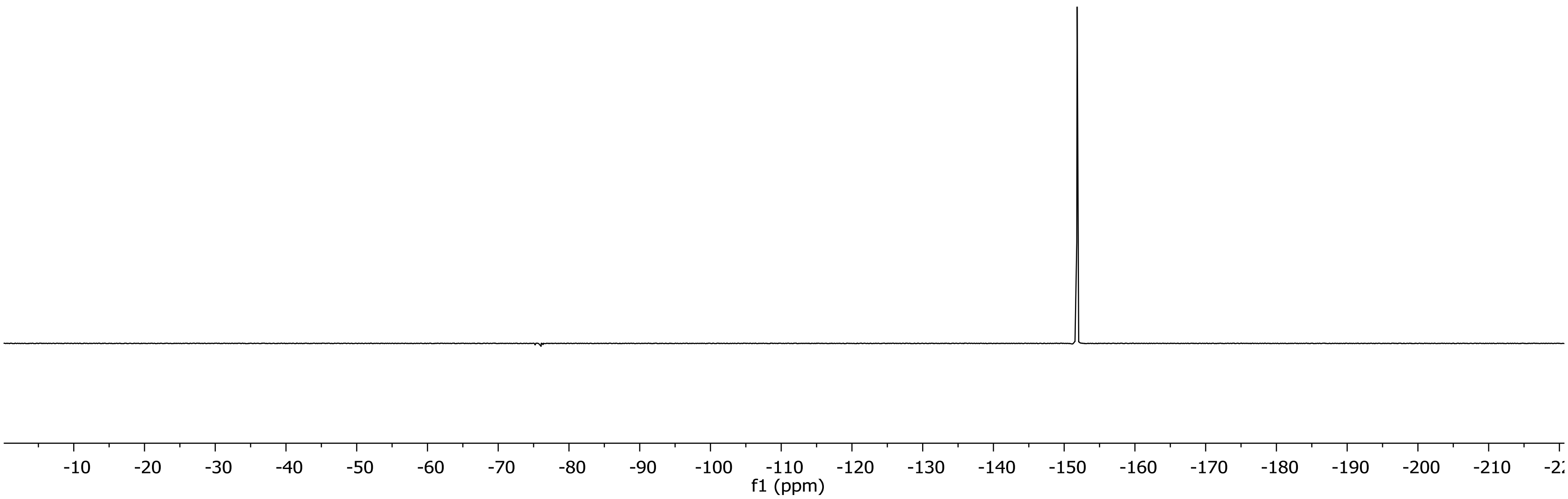

Cyclohexene-derived thianthrenium salt 20-TT

^{1}H NMR of cyclohexene-derived thianthrenium salt 20-TT

CD_2Cl_2, 298 K

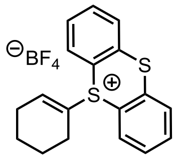

20-TT

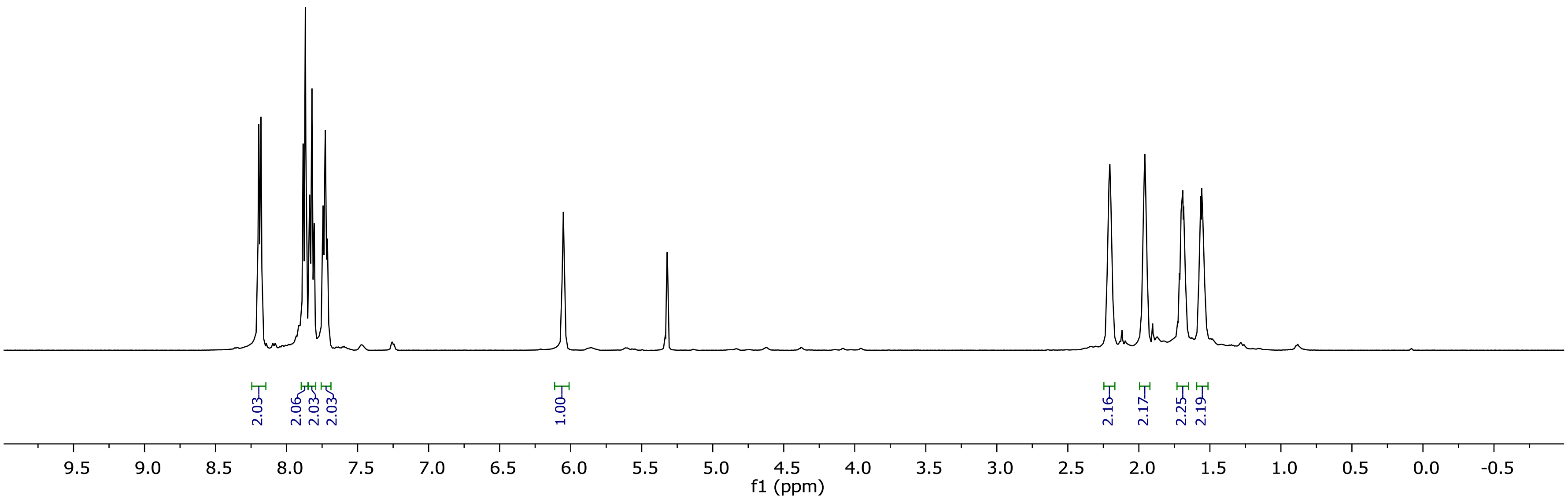

^{13}C NMR of cyclohexene-derived thianthrenium salt 20-TT

CD_2Cl_2, 298 K

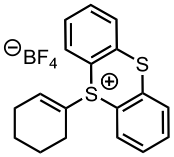

20-TT

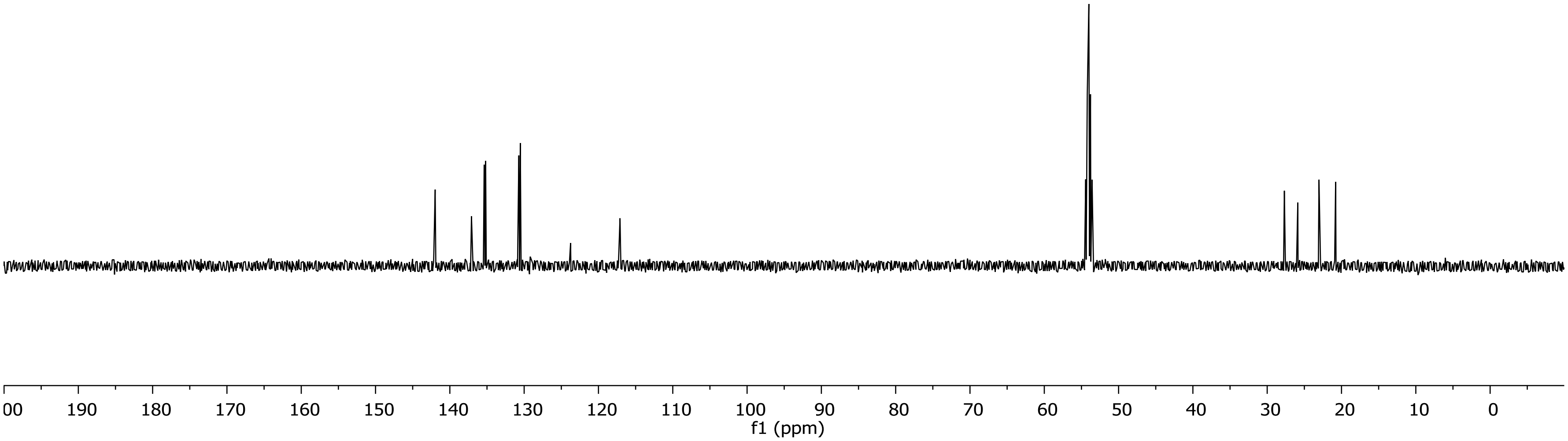

^{19}F NMR of cyclohexene-derived thianthrenium salt 20-TT

CD_2Cl_2, 298 K

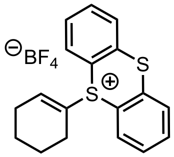

20-TT

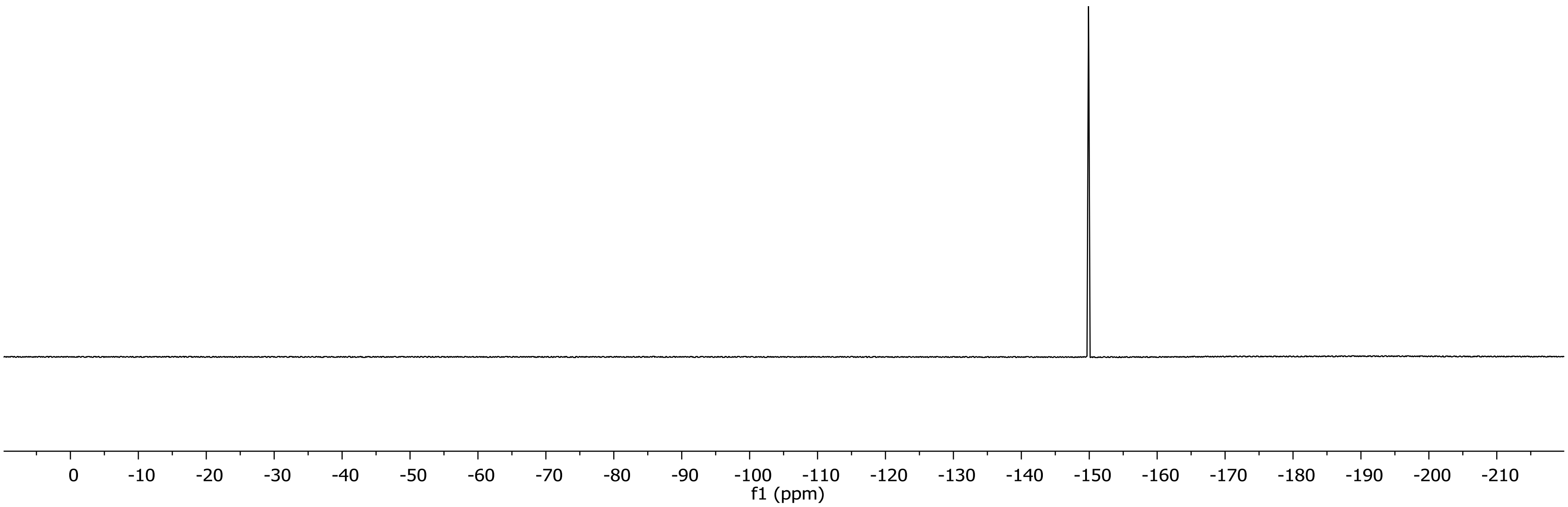

Cyclopentene-derived thianthrenium salt 21-TT

^{1}H NMR of cyclopentene-derived thianthrenium salt 21-TT

CD_2Cl_2, 298 K

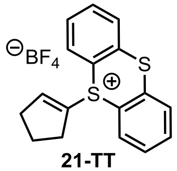

21-TT

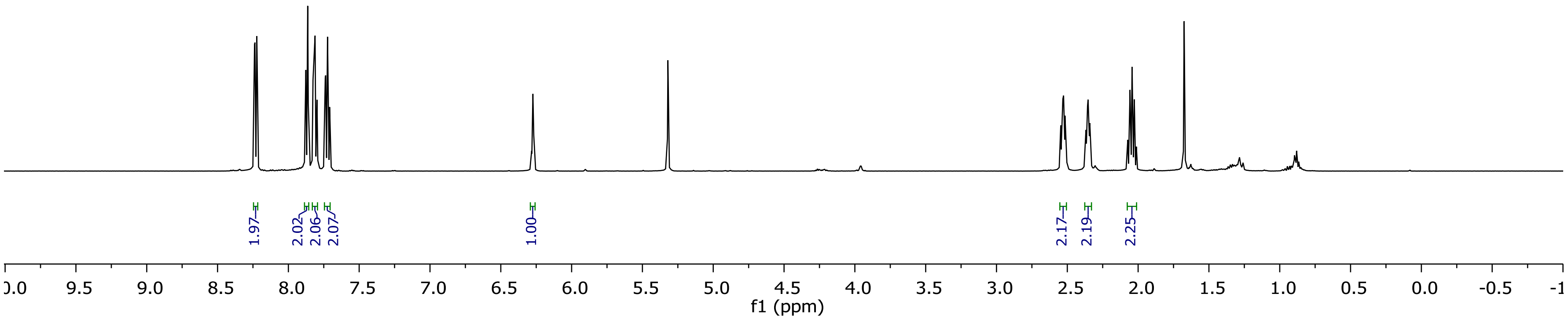

^{13}C NMR of cyclopentene-derived thianthrenium salt 21-TT

CD_2Cl_2, 298 K

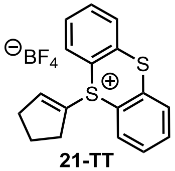

21-TT

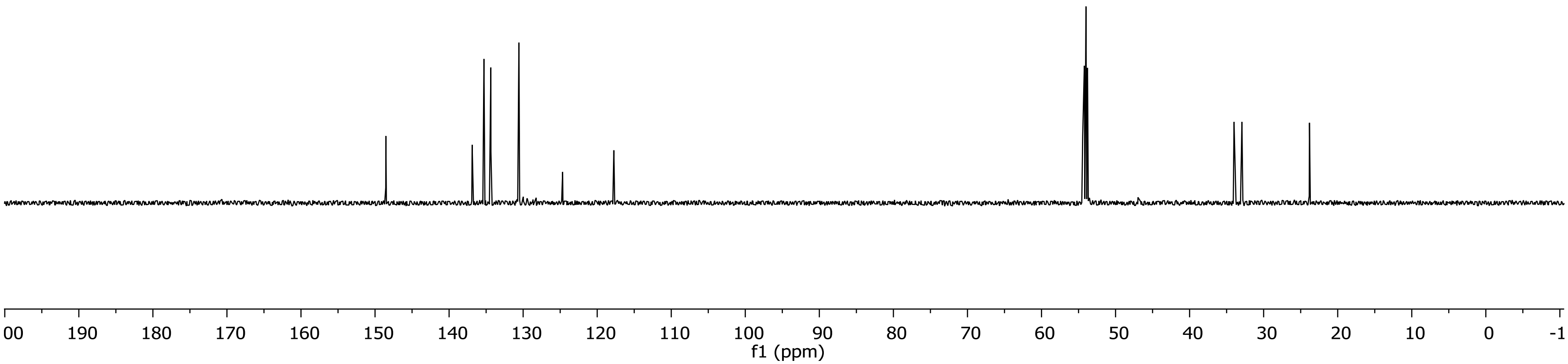

^{19}F NMR of cyclopentene-derived thianthrenium salt 21-TT

CD_2Cl_2, 298 K

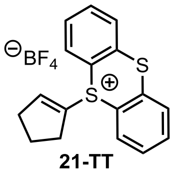

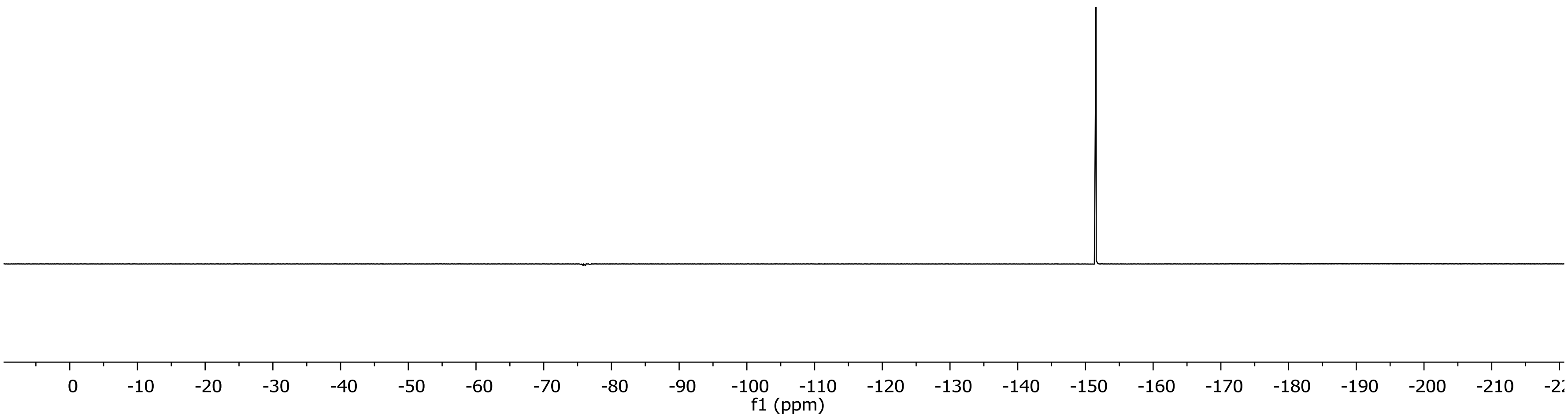

(*E,E,E*)-1,5,9-Cyclododecatriene-derived thianthrenium salt 22-TT

^{1}H NMR of (*E,E,E*)-1,5,9-cyclododecatriene-derived thianthrenium salt 22-TT

CD_2Cl_2, 298 K

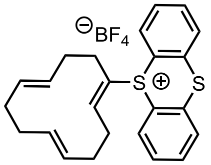

22-TT

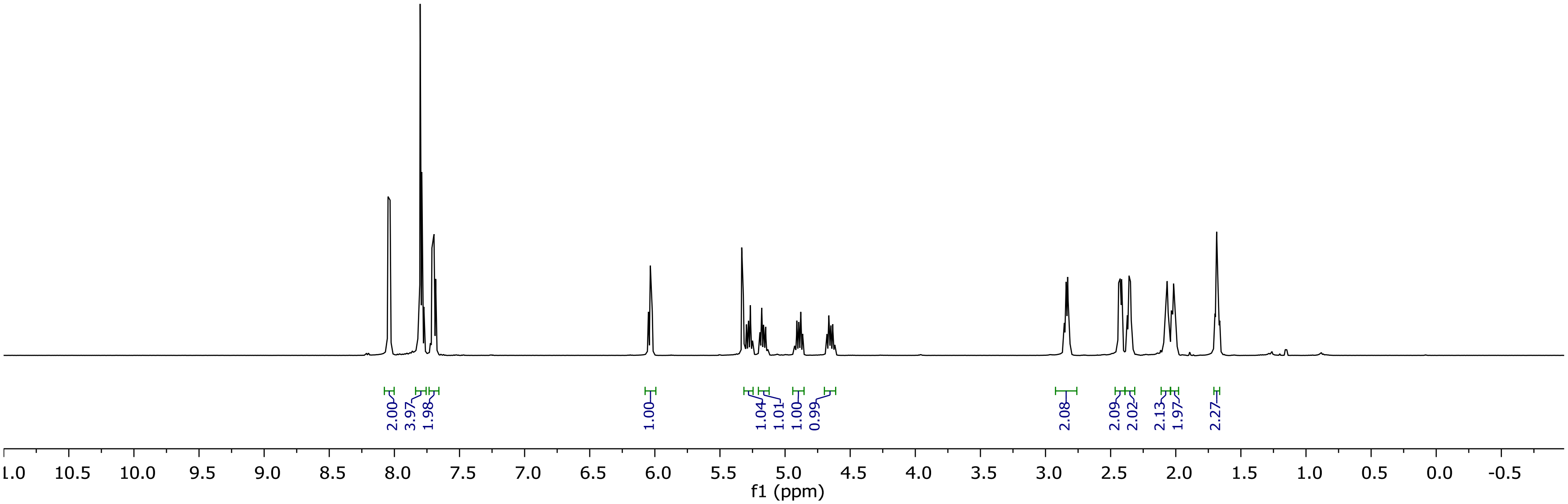

^{13}C NMR of (*E,E,E*)-1,5,9-cyclododecatriene-derived thianthrenium salt 22-TT

CD_2Cl_2, 298 K

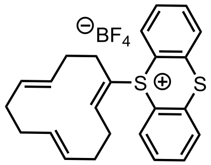

22-TT

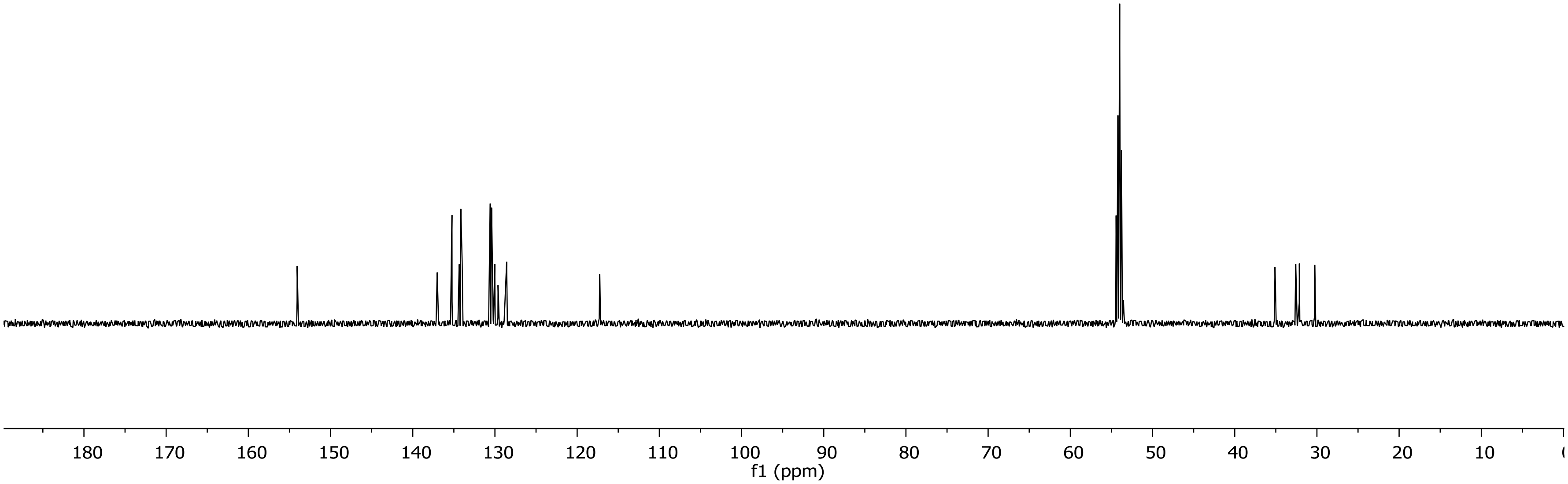

^{19}F NMR of (*E*,*E*,*E*)-1,5,9-cyclododecatriene-derived thianthrenium salt 22-TT

CD_2Cl_2, 298 K

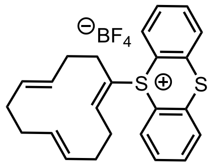

22-TT

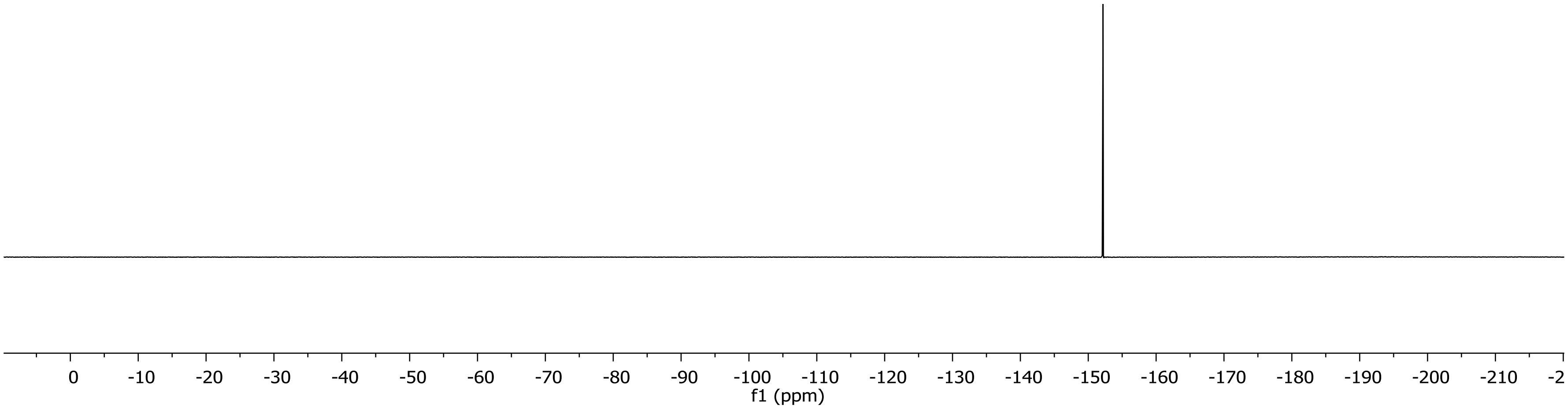

1,9-Cyclohexadecadiene-derived thianthrenium salt 23-TT

^{1}H NMR of 1,9-cyclohexadecadiene-derived thianthrenium salt 23-TT

CD_2Cl_2, 298 K

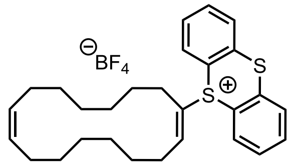

23-TT

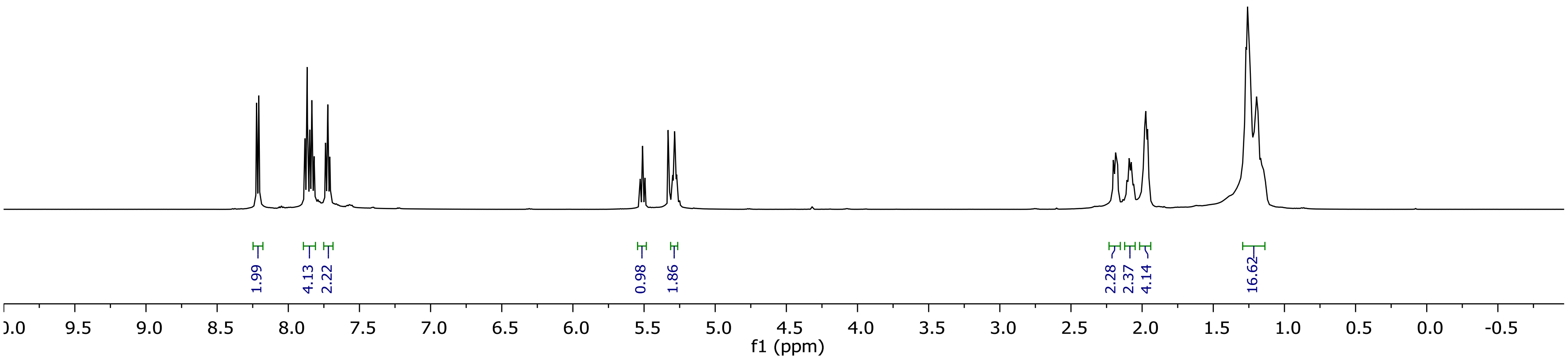

^{13}C NMR of 1,9-cyclohexadecadiene-derived thianthrenium salt 23-TT

CD_2Cl_2, 298 K

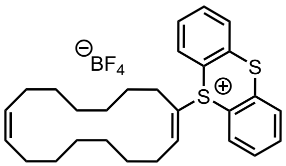

23-TT

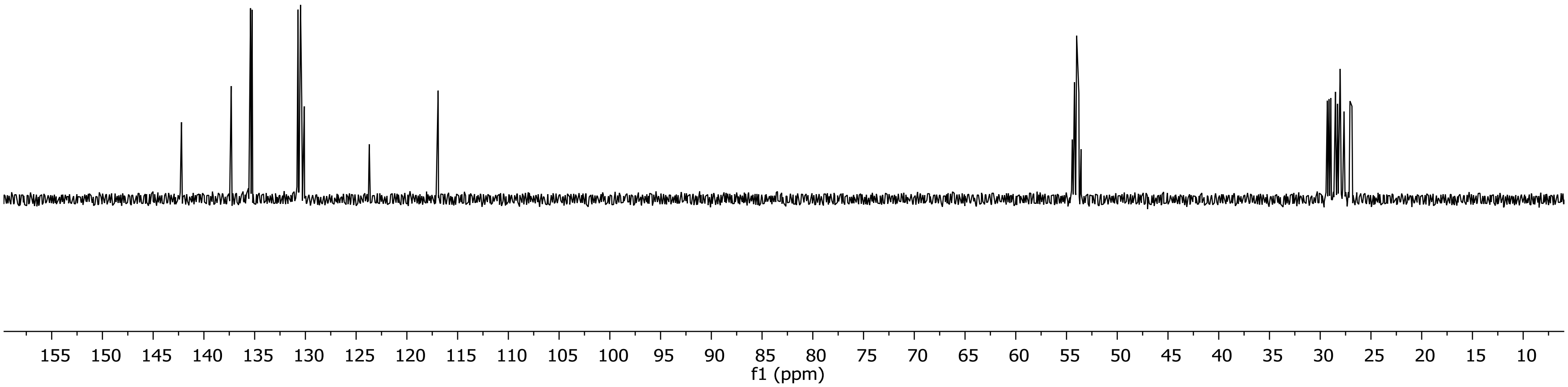

^{19}F NMR of 1,9-cyclohexadecadiene-derived thianthrenium salt 23-TT

CD_2Cl_2, 298 K

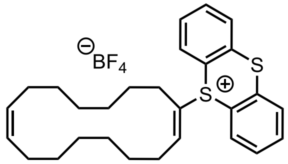

23-TT

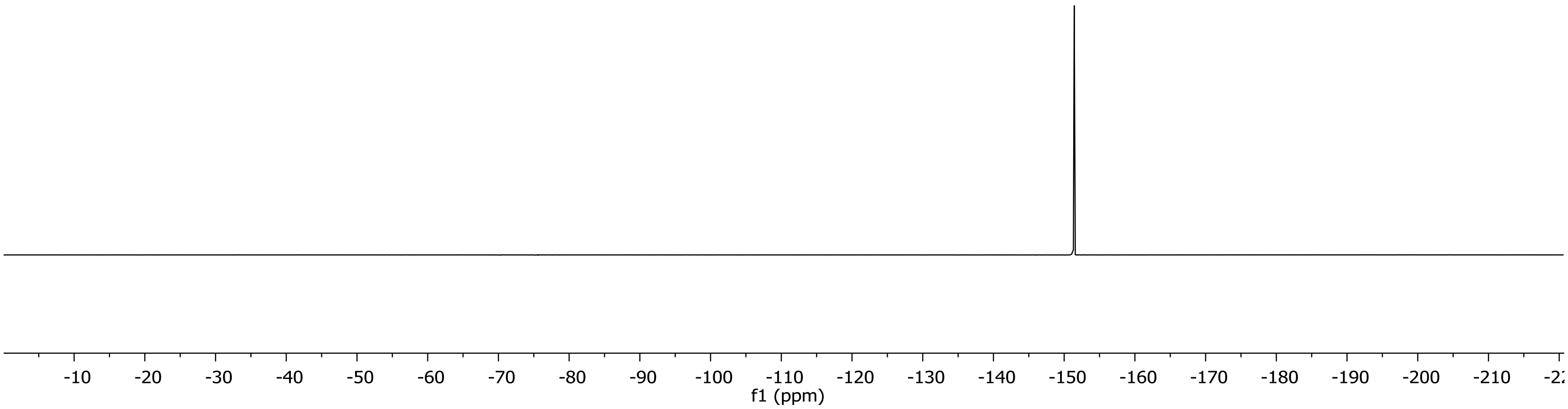

1,5-Cyclooctadiene-derived thianthrenium salt 24-TT

^{1}H NMR of 1,5-cyclooctadiene-derived thianthrenium salt 24-TT

$CDCl_3$, 298 K

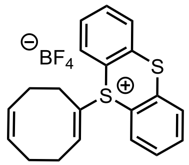

24-TT

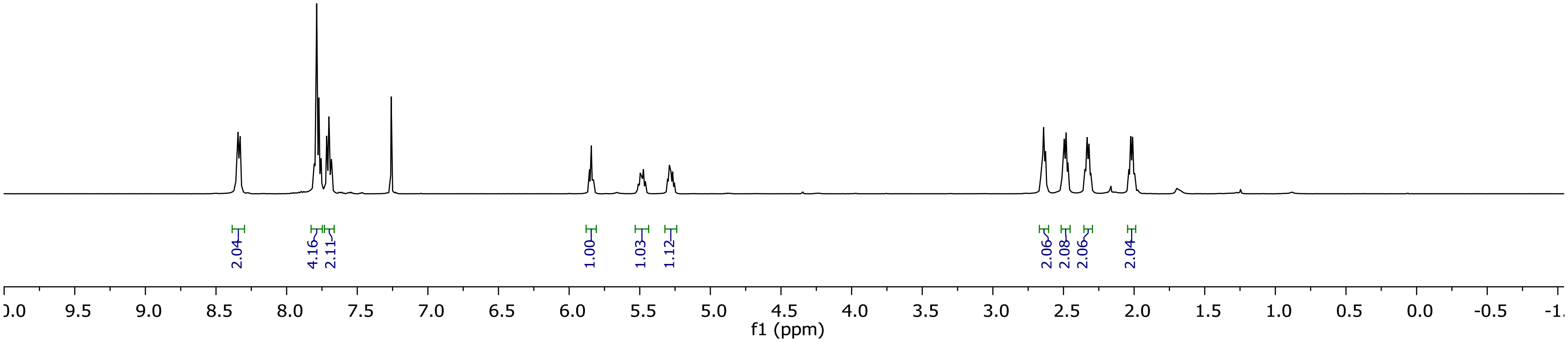

^{13}C NMR of 1,5-cyclooctadiene-derived thianthrenium salt 24-TT

$CDCl_3$, 298 K

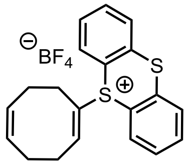

24-TT

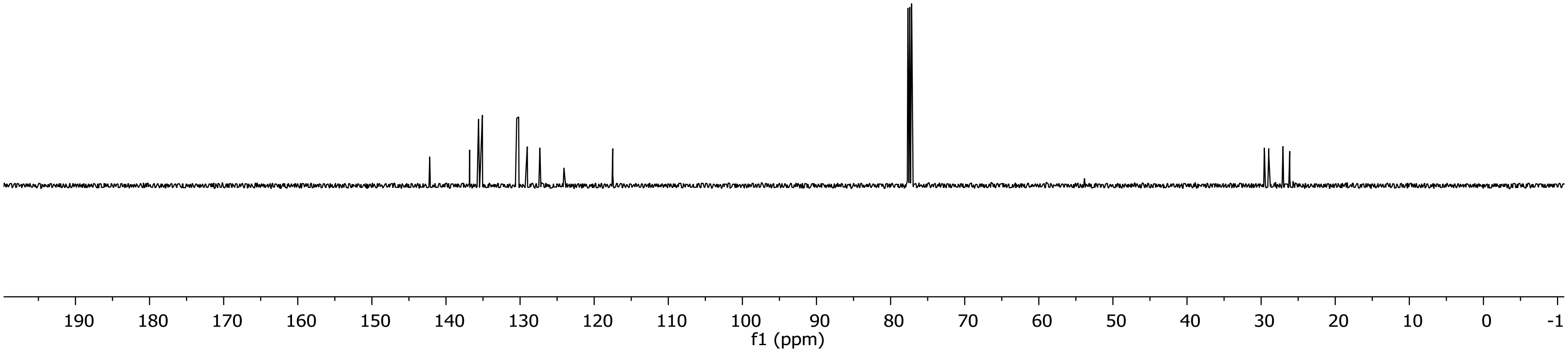

^{19}F NMR of 1,5-cyclooctadiene-derived thianthrenium salt 24-TT

$CDCl_3$, 298 K

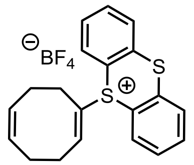

24-TT

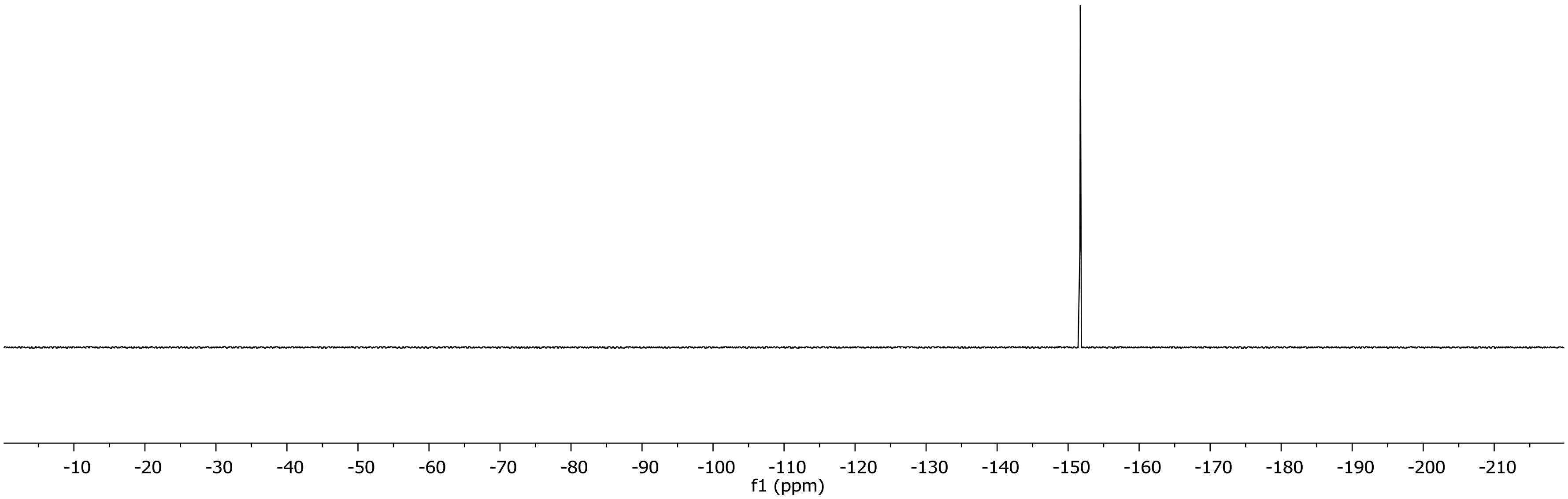

Tricyclo[6.2.1.02,7]undeca-4-ene-derived thianthrenium salt 25-TT

^{1}H NMR of tricyclo[6.2.1.02,7]undeca-4-ene-derived thianthrenium salt 25-TT

CD_2Cl_2, 298 K

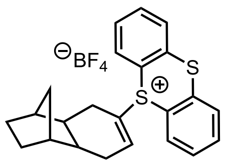

25-TT

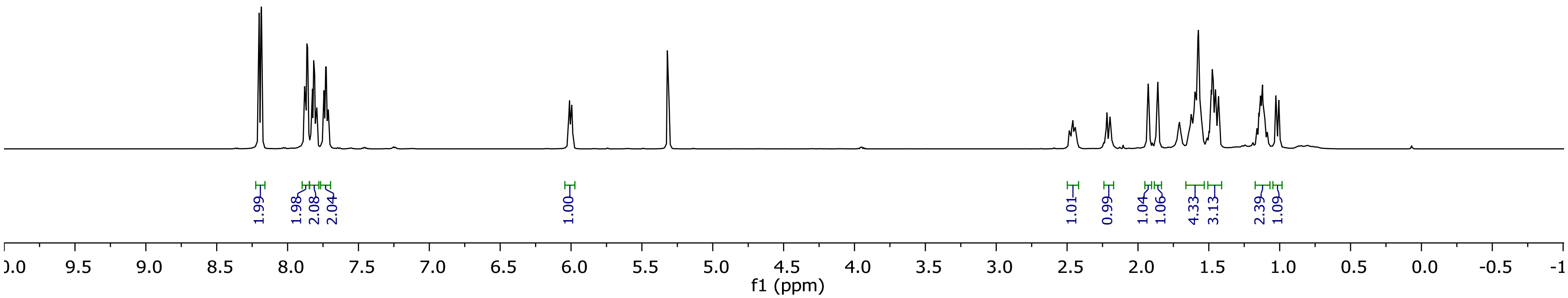

^{13}C NMR of tricyclo[6.2.1.02,7]undeca-4-ene-derived thianthrenium salt 25-TT

CD_2Cl_2, 298 K

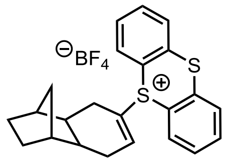

25-TT

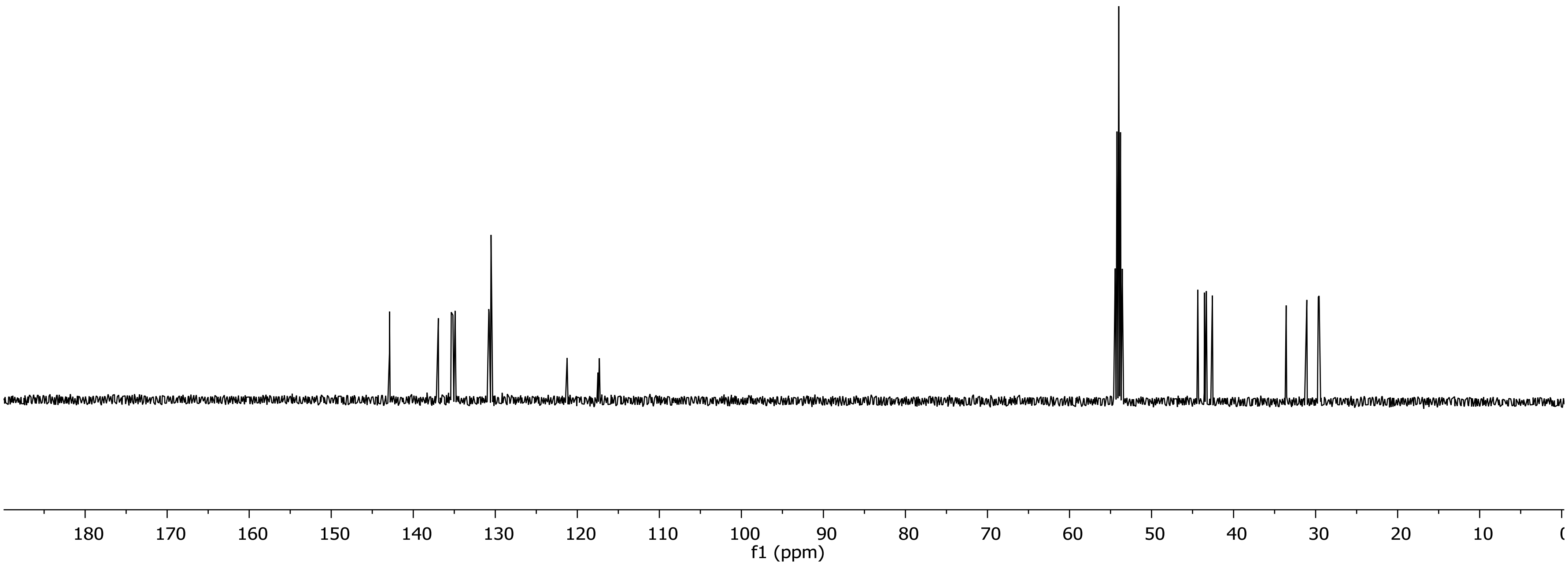

^{19}F NMR of tricyclo[6.2.1.02,7]undeca-4-ene-derived thianthrenium salt 25-TT

CD_2Cl_2, 298 K

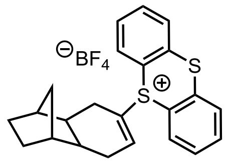

25-TT

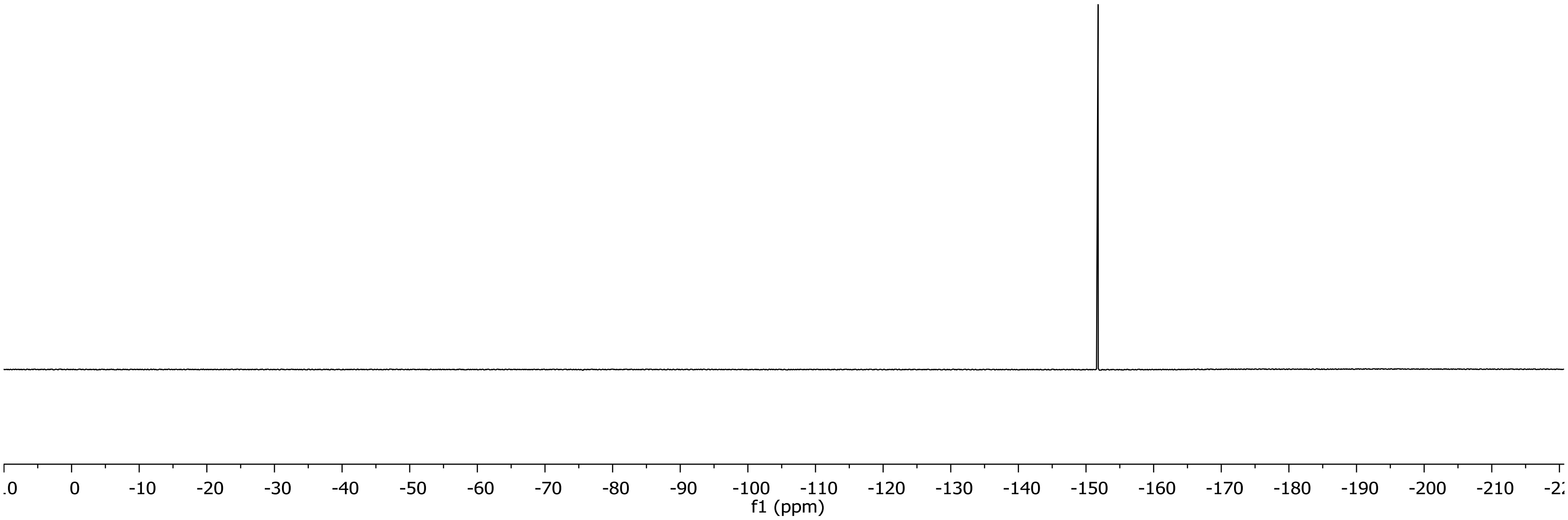

Nicotinic acid-derived thianthrenium salt 26-TT

^{1}H NMR of nicotinic acid-derived thianthrenium salt 26-TT(*E*)

CD_2Cl_2, 298 K

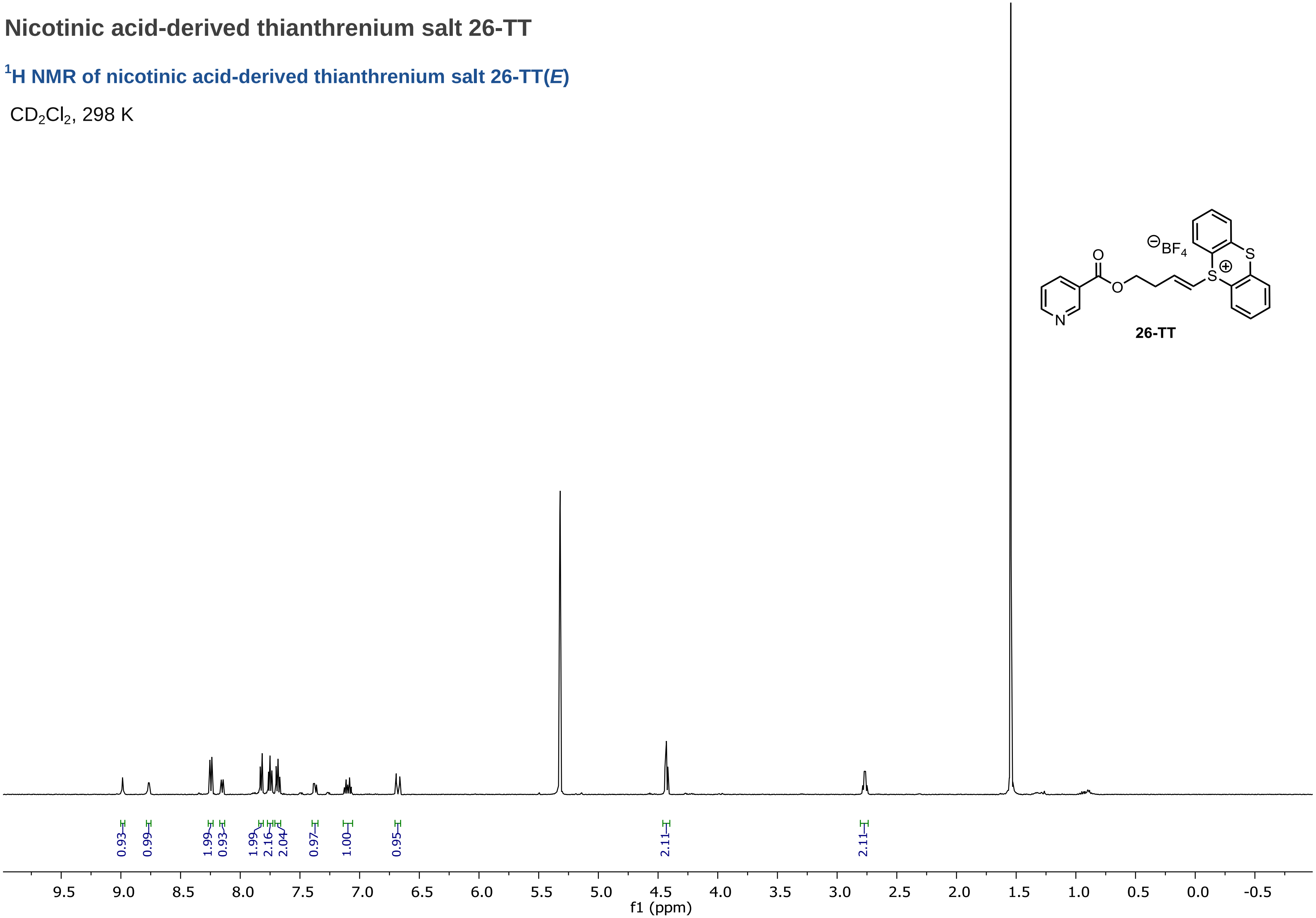

^{13}C NMR of nicotinic acid-derived thianthrenium salt 26-TT

CD_2Cl_2, 298 K

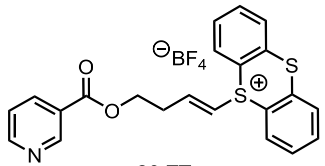

26-TT

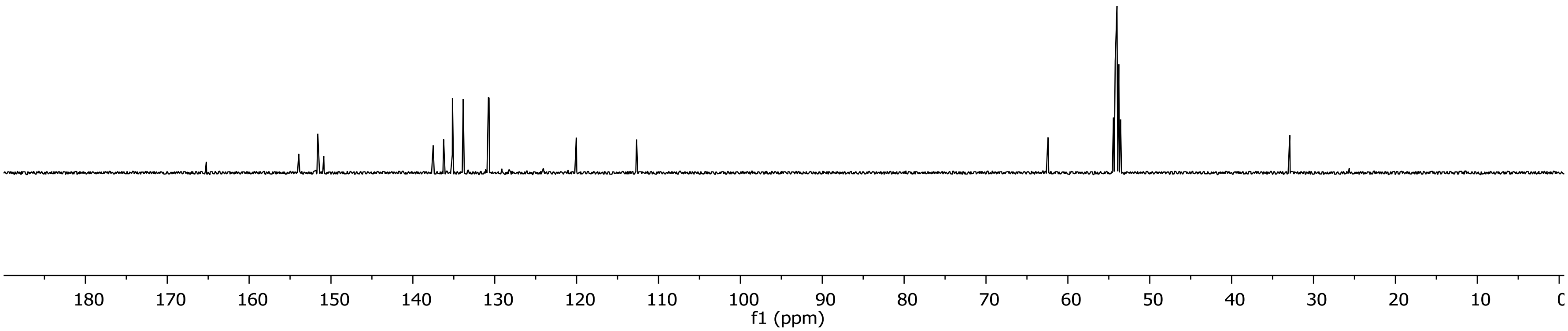

^{19}F NMR of nicotinic acid-derived thianthrenium salt 26-TT

CD_2Cl_2, 298 K

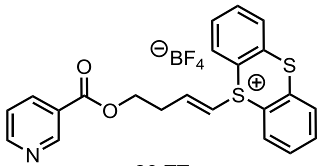

26-TT

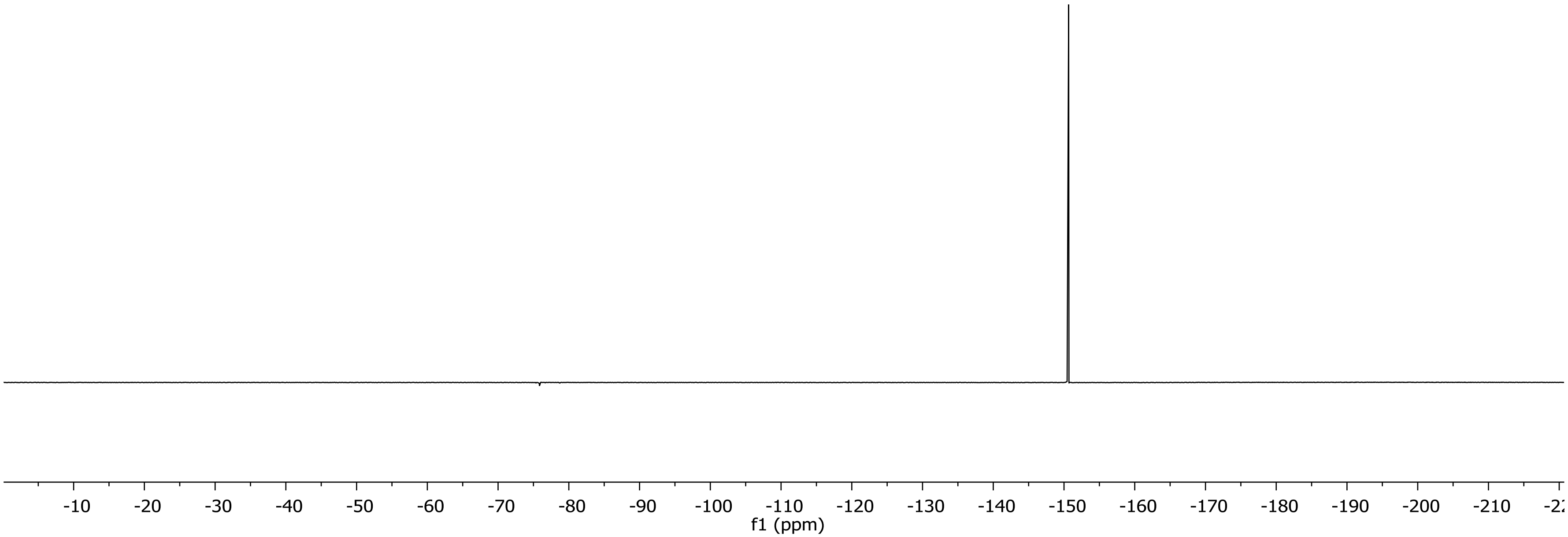

Cinchophene-derived thianthrenium salt 27-TT

^{1}H NMR of cinchophene-derived thianthrenium salt 27-TT

CD_2Cl_2, 298 K

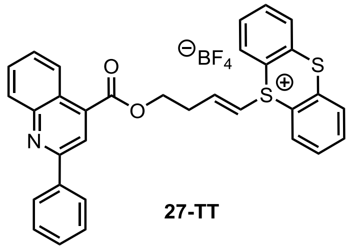

27-TT

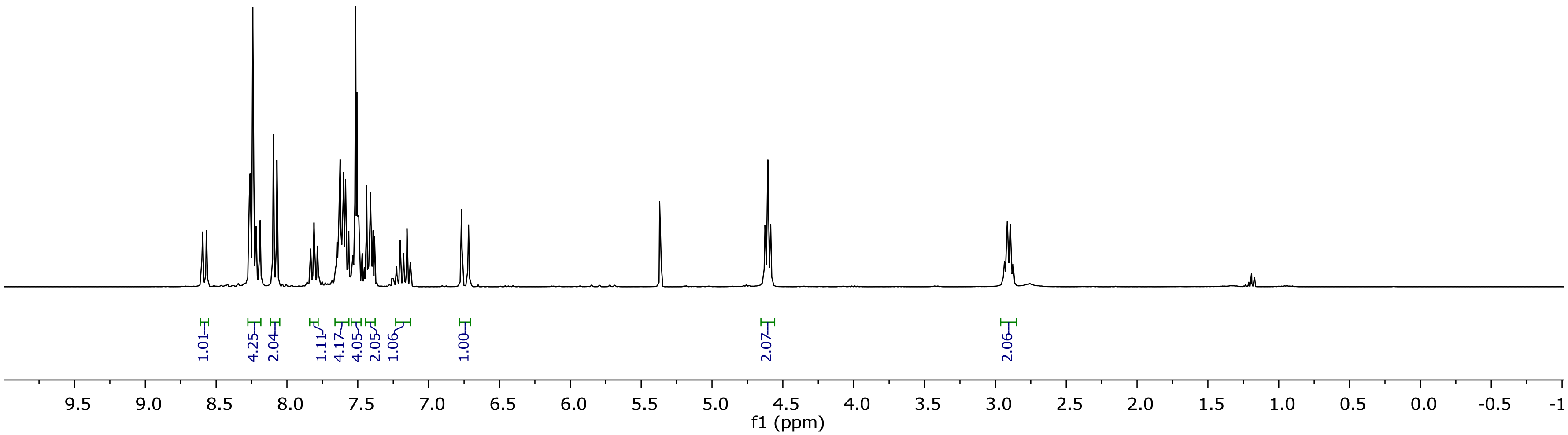

^{13}C NMR of cinchophene-derived thianthrenium salt 27-TT

CD_2Cl_2, 298 K

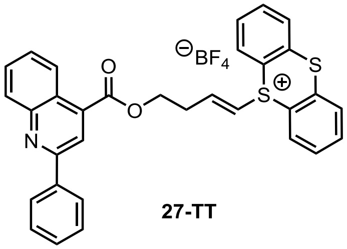

27-TT

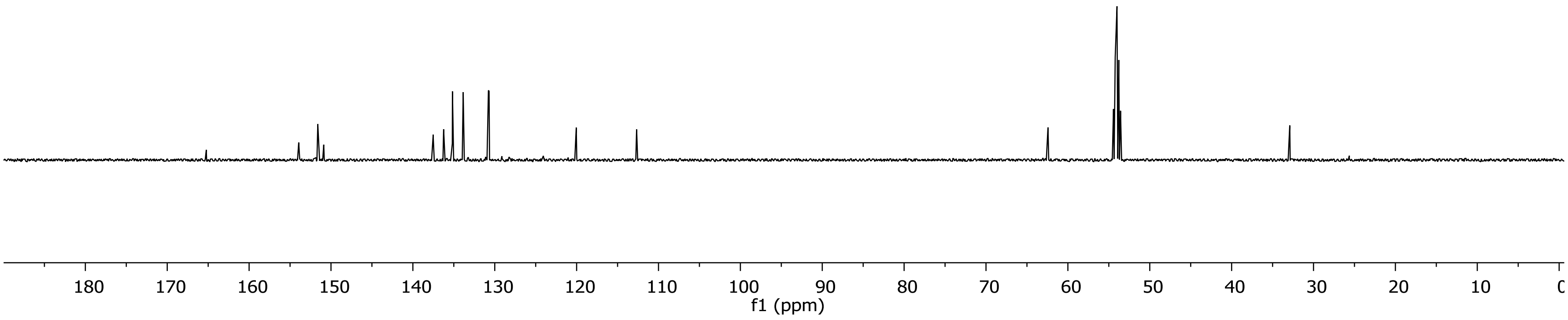

^{19}F NMR of cinchophene-derived thianthrenium salt 27-TT

CD_2Cl_2, 298 K

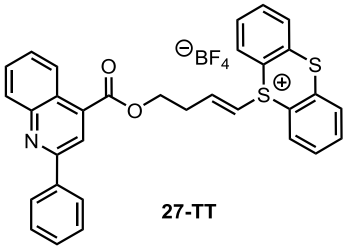

27-TT

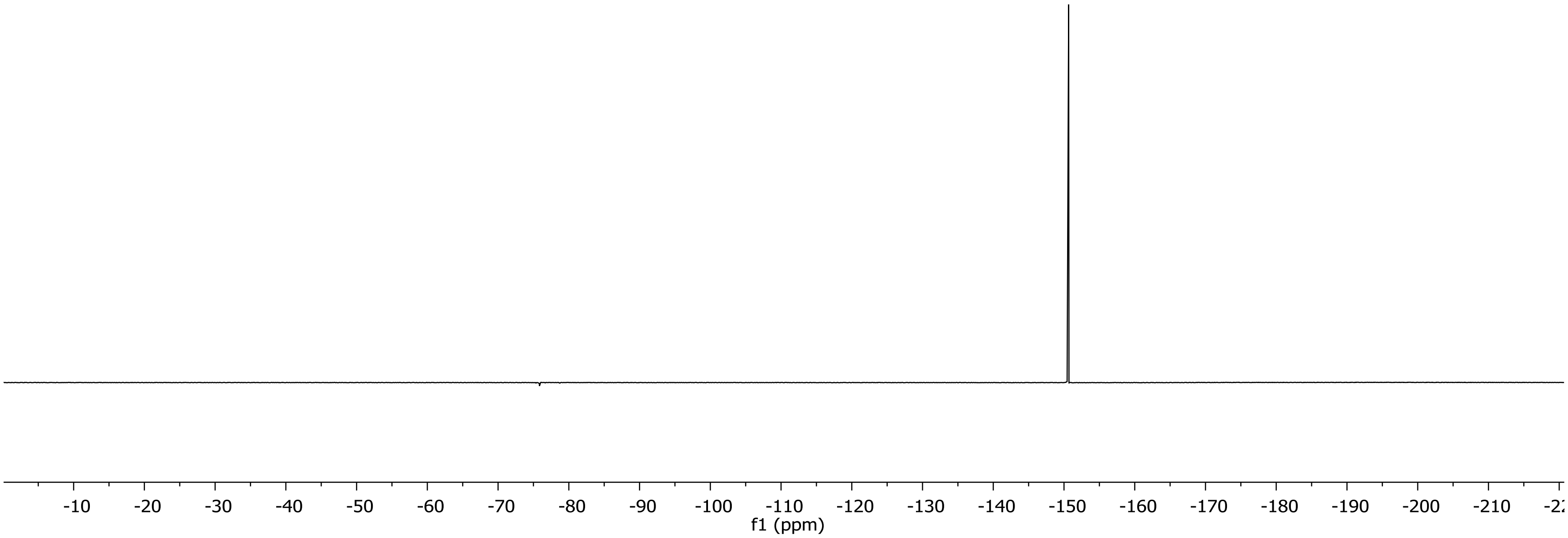

Lithocholic acid-derived thianthrenium salt 28-TT

^{1}H NMR of lithocholic acid-derived thianthrenium salt 28-TT

CD_2Cl_2, 298 K

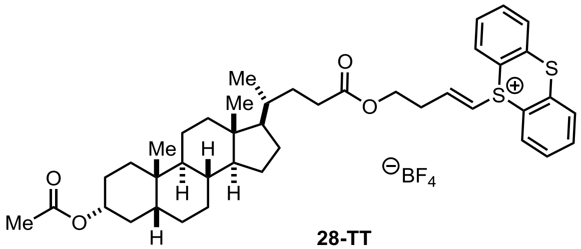

28-TT

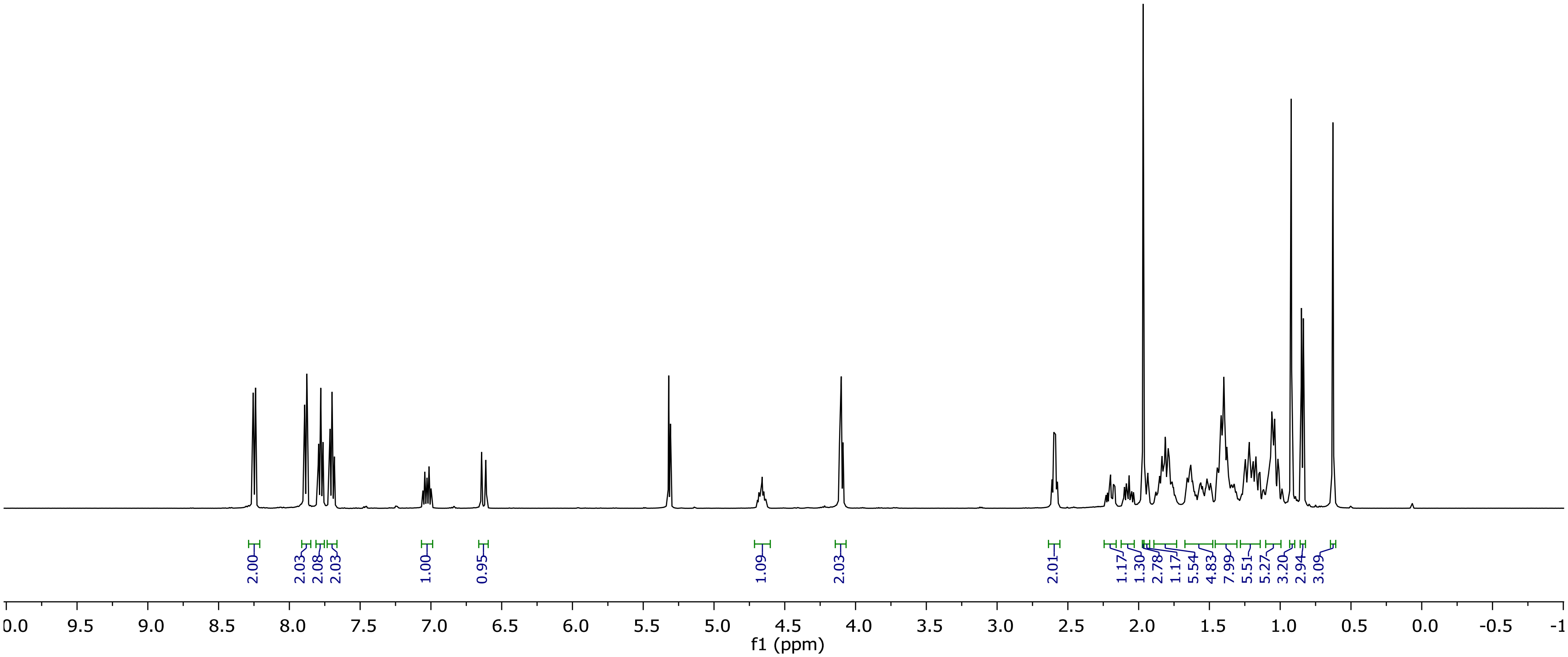

^{13}C NMR of lithocholic acid-derived thianthrenium salt 28-TT

CD_2Cl_2, 298 K

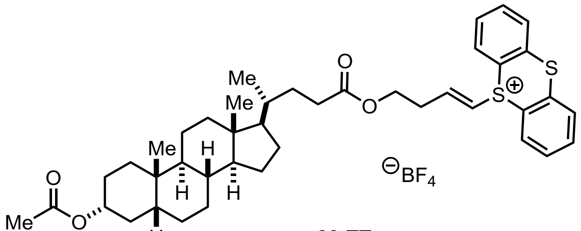

28-TT

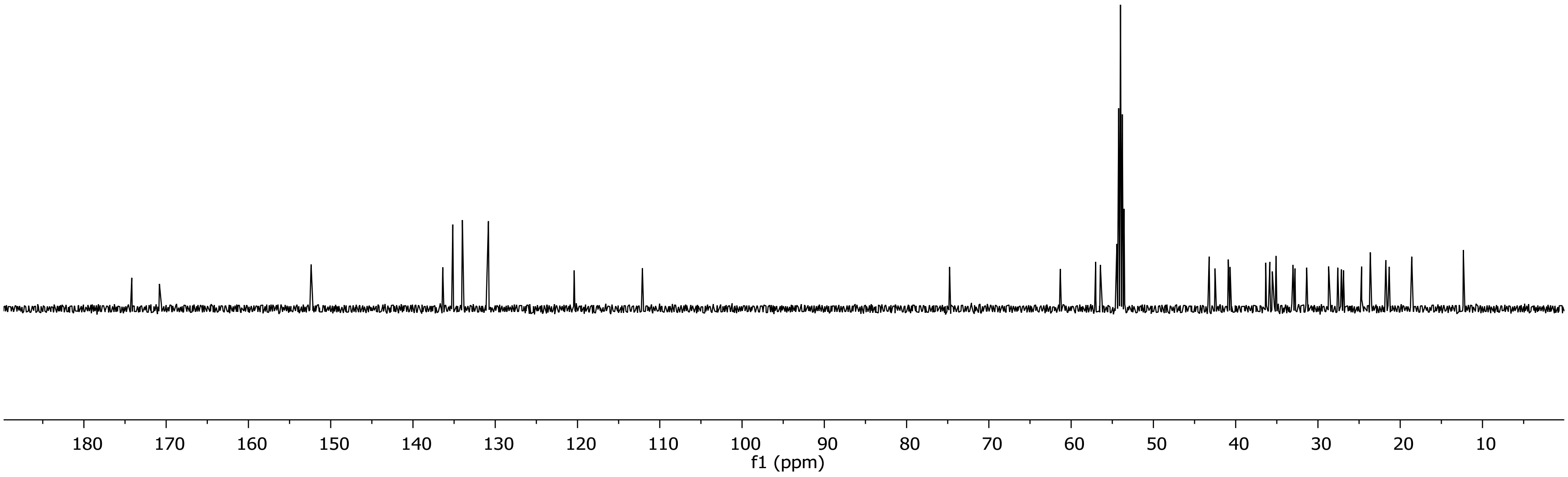

^{19}F NMR of lithocholic acid-derived thianthrenium salt 28-TT

CD_2Cl_2, 298 K

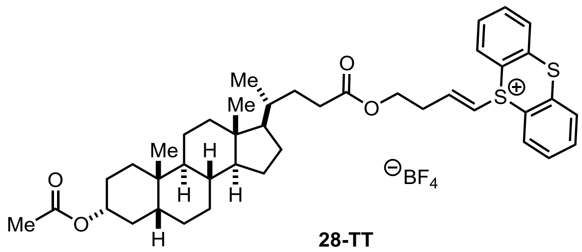

28-TT

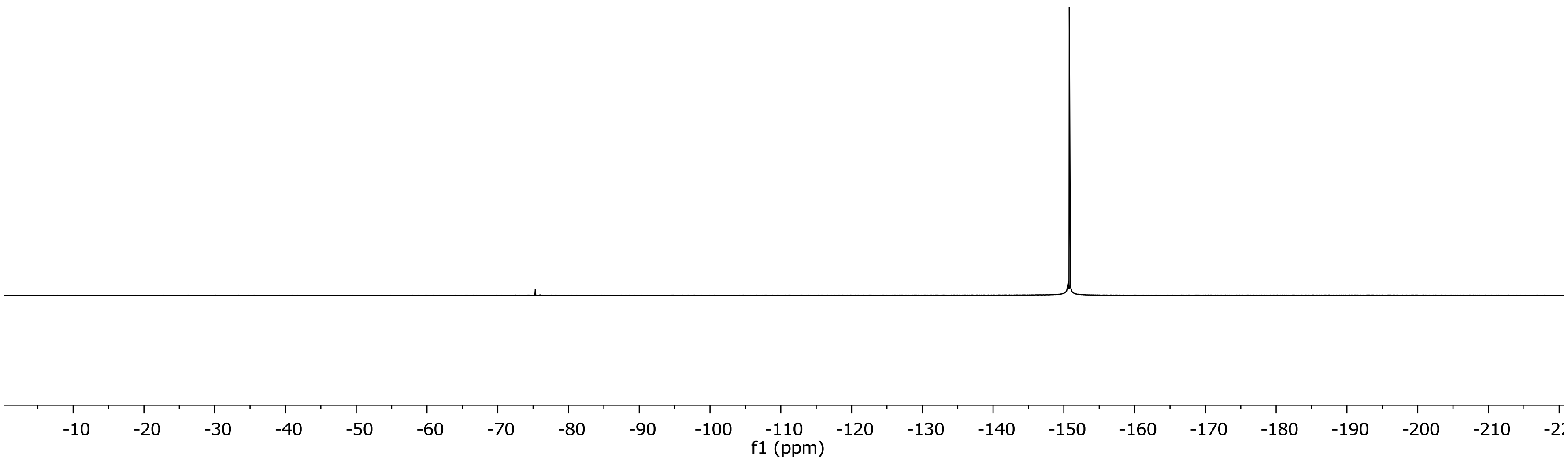

Epiandrosterone-derived thianthrenium salt 29-TT

^{1}H NMR of epiandrosterone-derived thianthrenium salt 29-TT

CD_2Cl_2, 298 K

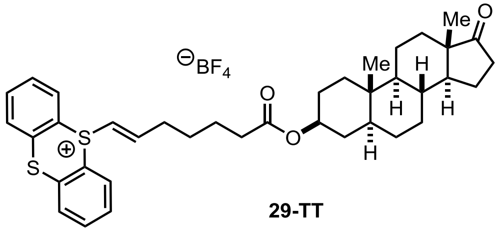

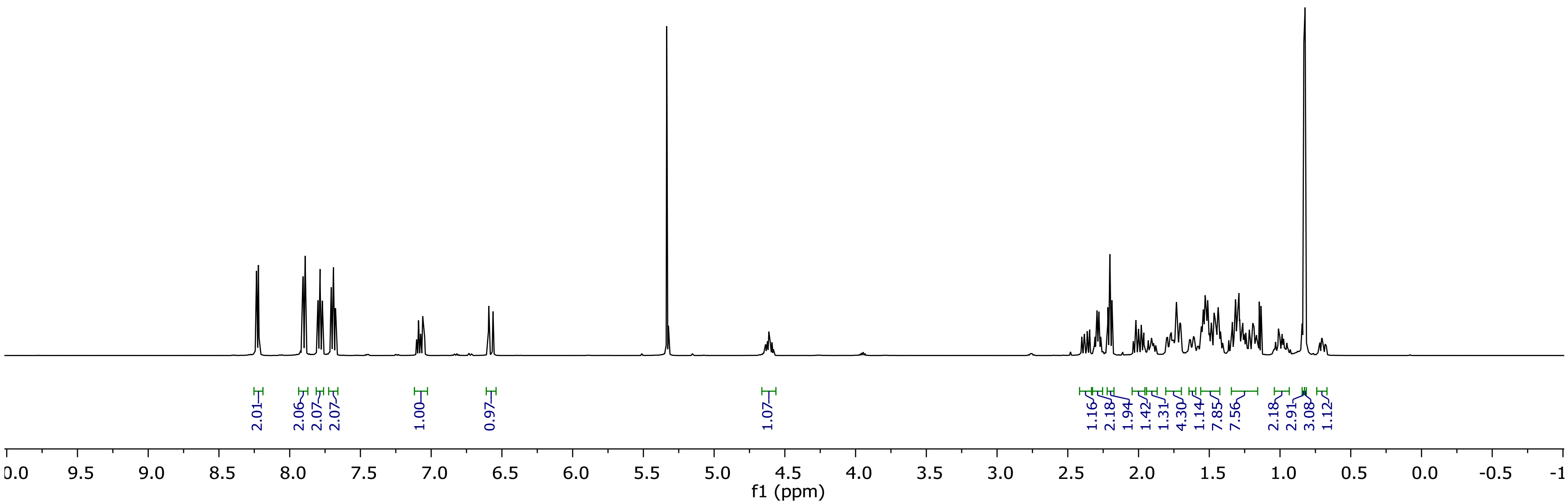

^{13}C NMR of epiandrosterone-derived thianthrenium salt 29-TT

CD_2Cl_2, 298 K

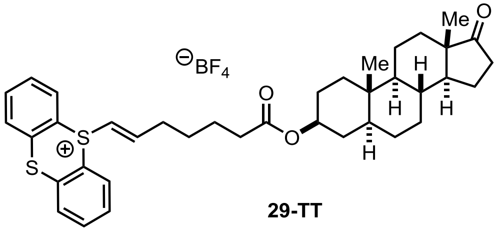

29-TT

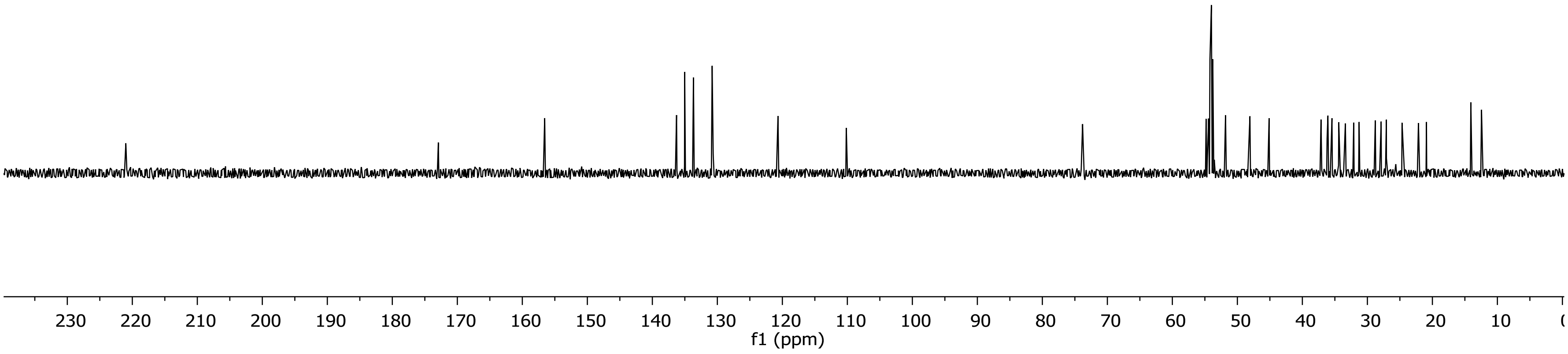

^{19}F NMR of epiandrosterone-derived thianthrenium salt 29-TT

CD_2Cl_2, 298 K

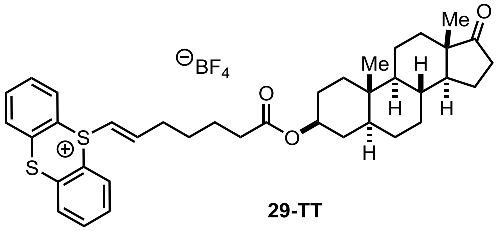

29-TT

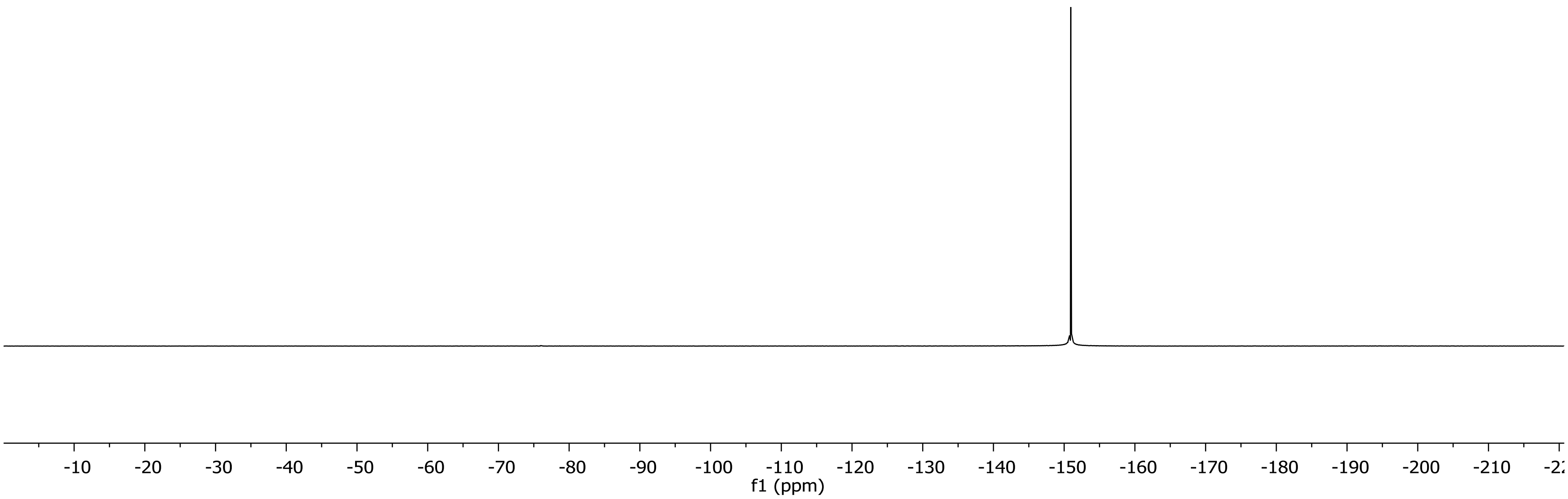

Piperidine-derived thianthrenium salt 30-TT

¹H NMR of piperidine-derived thianthrenium salt 30-TT

$CDCl_3$, 298 K

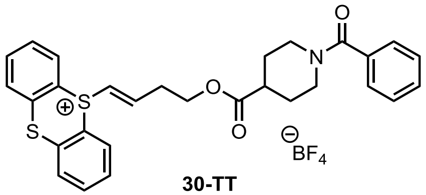

30-TT

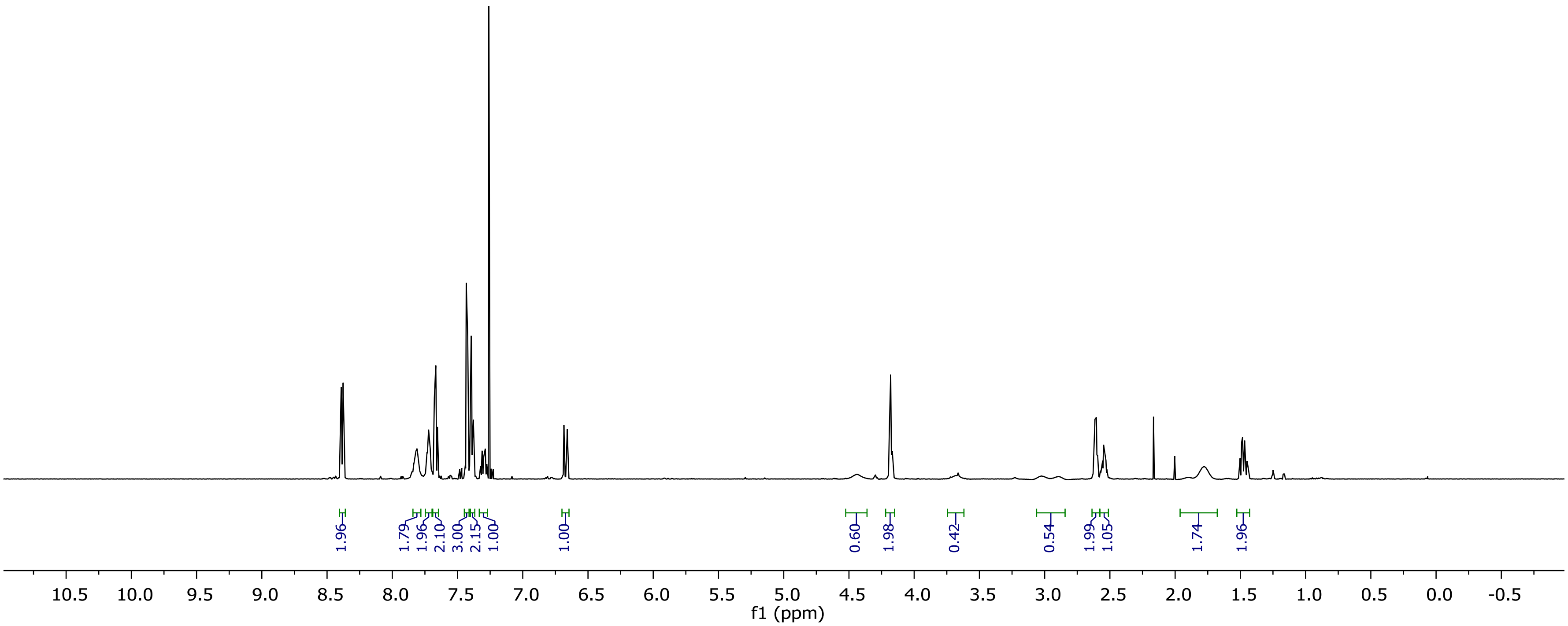

^{13}C NMR of piperidine-derived thianthrenium salt 30-TT

$CDCl_3$, 298 K

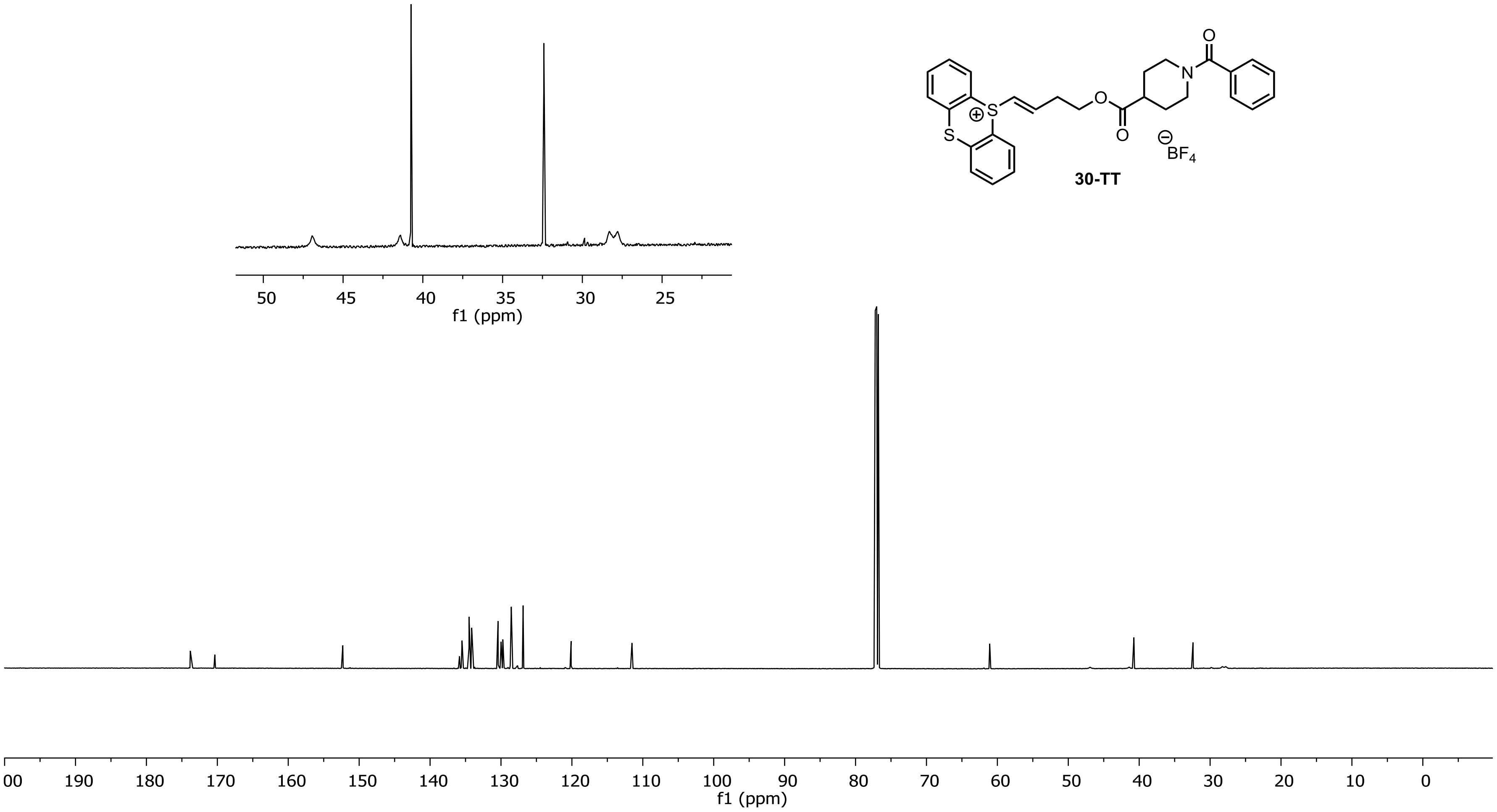

^{19}F NMR of piperidine-derived thianthrenium salt 30-TT

$CDCl_3$, 298 K

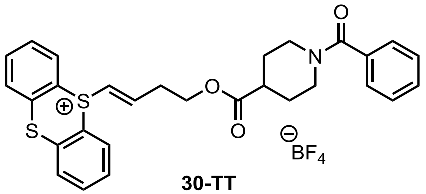

30-TT

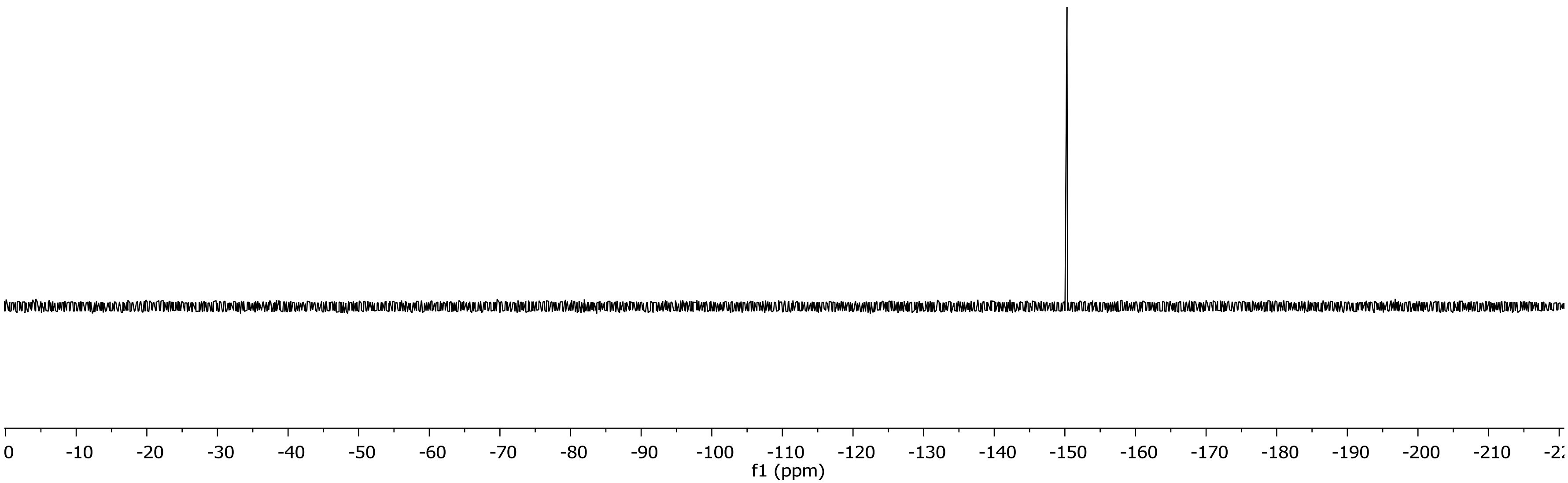

Fluazinam-derived thianthrenium salt 31-TT

^{1}H NMR of Fluazinam-derived thianthrenium salt 31-TT

$CDCl_3$, 298 K

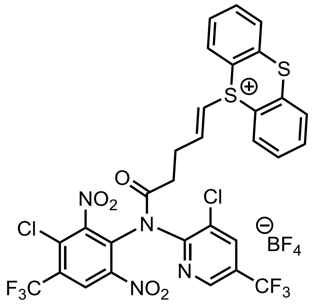

31-TT

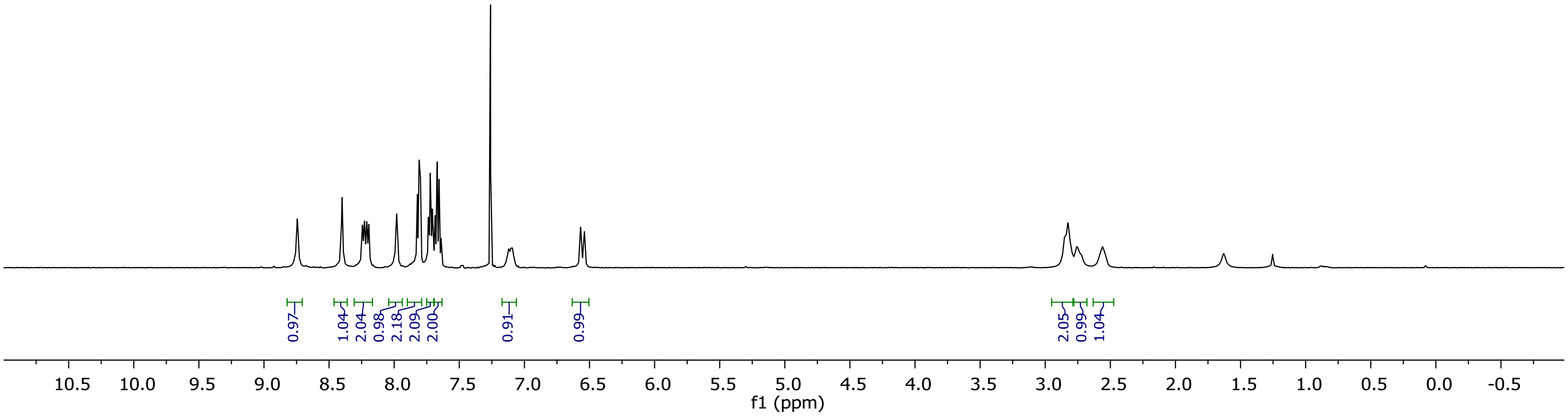

^{13}C NMR of Fluazinam-derived thianthrenium salt 31-TT

$CDCl_3$, 268 K

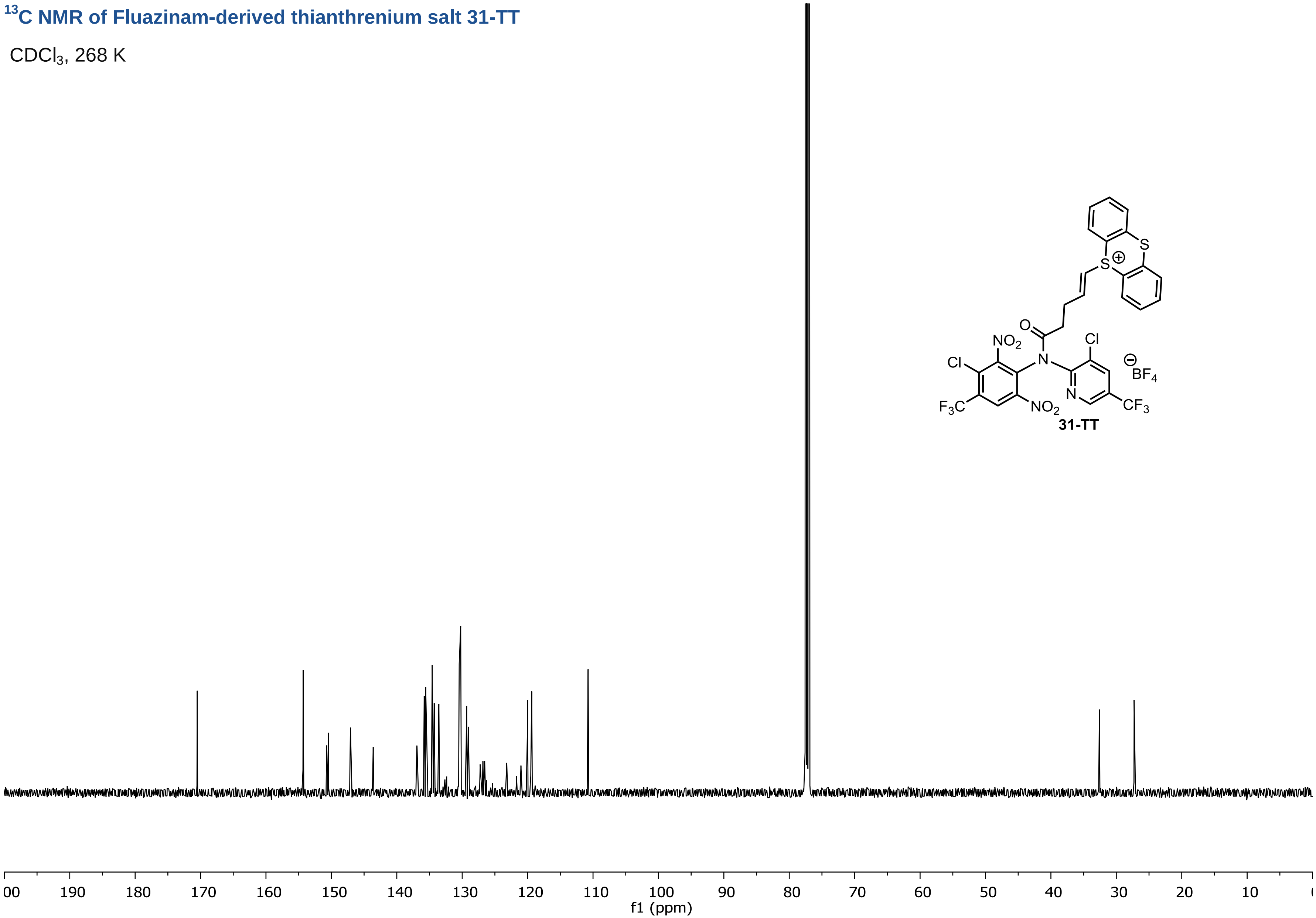

^{19}F NMR of Fluazinam-derived thianthrenium salt 31-TT

$CDCl_3$, 298 K

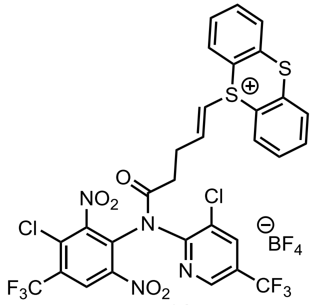

31-TT

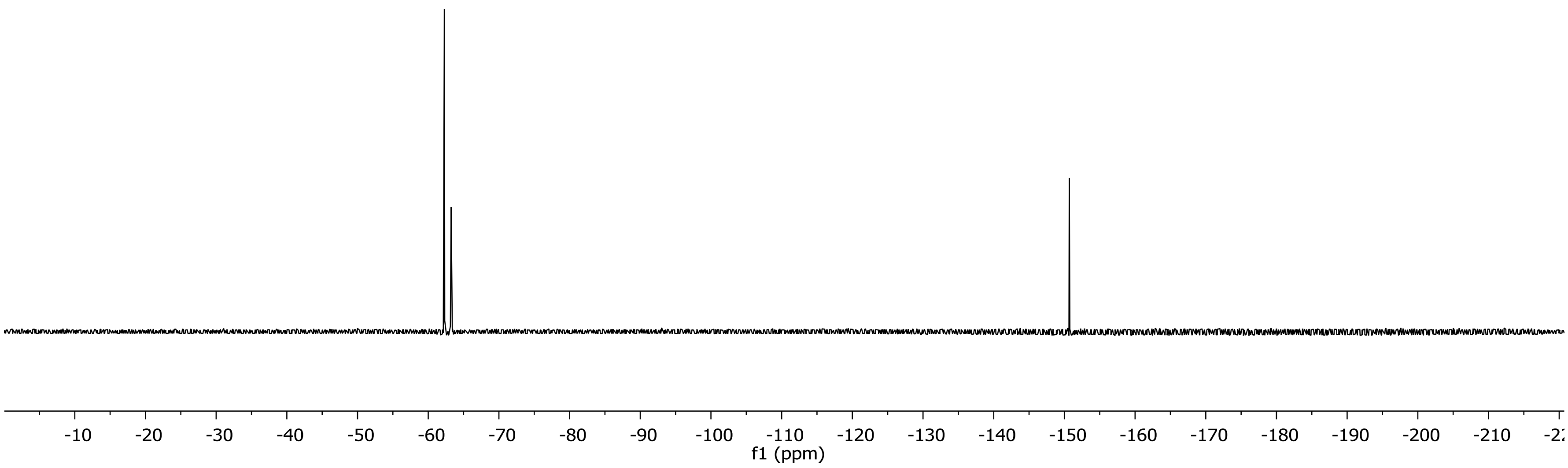

Probenecid-derived thianthrenium salt 32-TT

^{1}H NMR of Probenecid-derived thianthrenium salt 32-TT

$CDCl_3$, 298 K

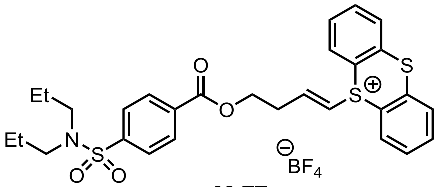

32-TT

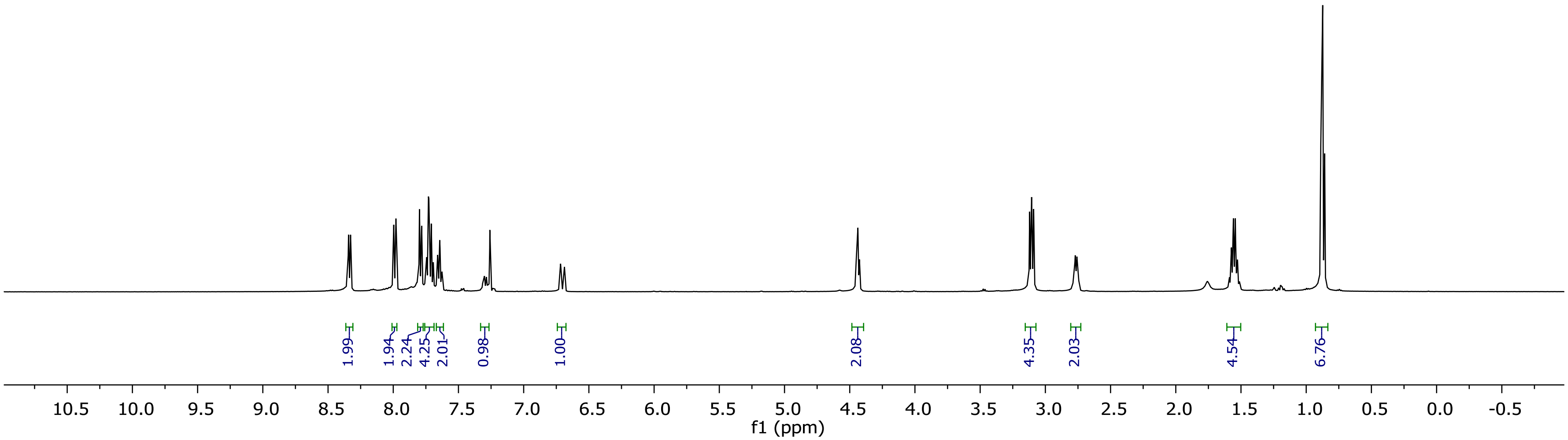

^{13}C NMR of Probenecid-derived thianthrenium salt 32-TT

$CDCl_3$, 298 K

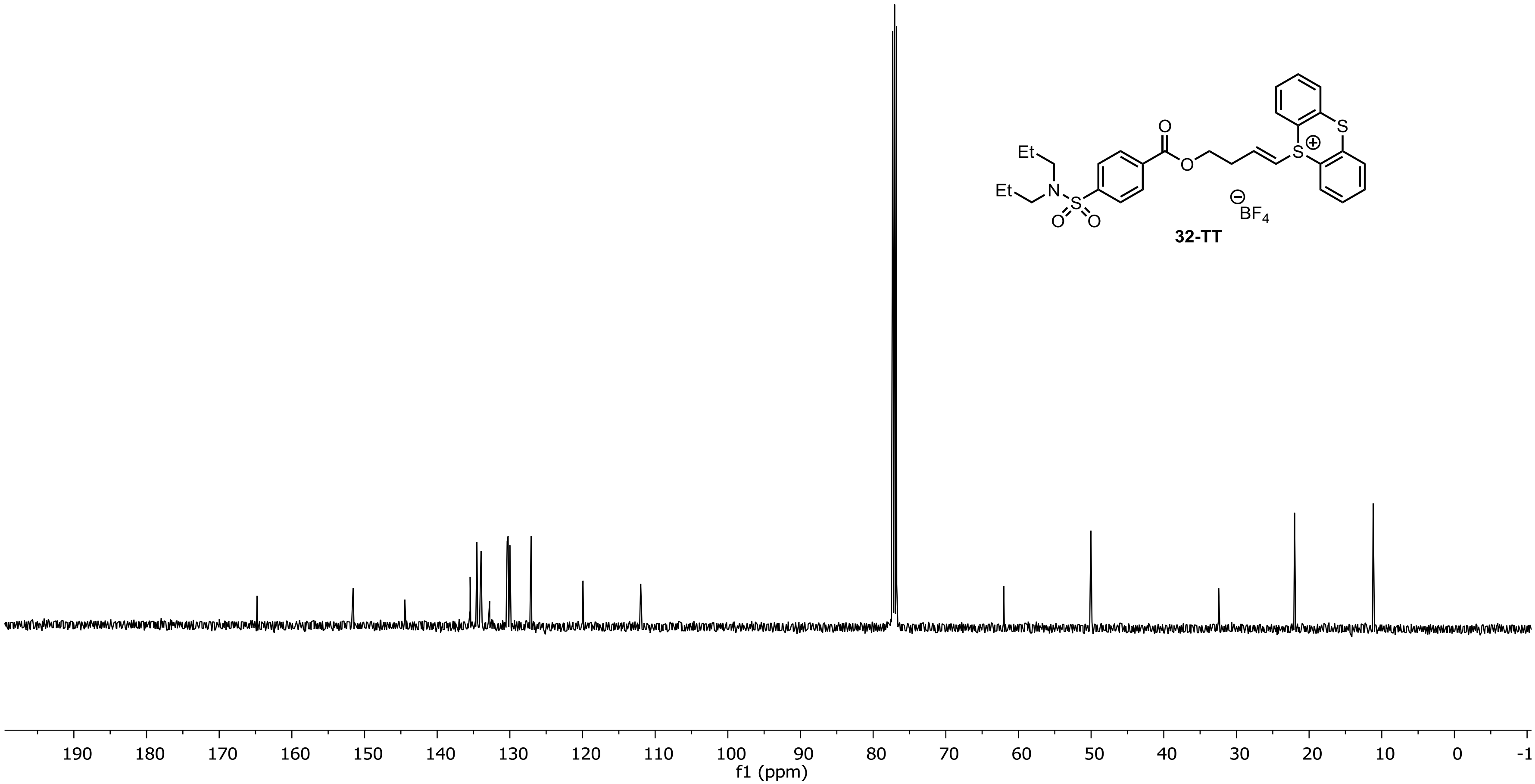

^{19}F NMR of Probenecid-derived thianthrenium salt 32-TT

$CDCl_3$, 298 K

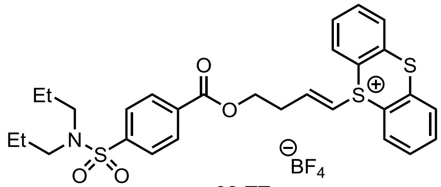

32-TT

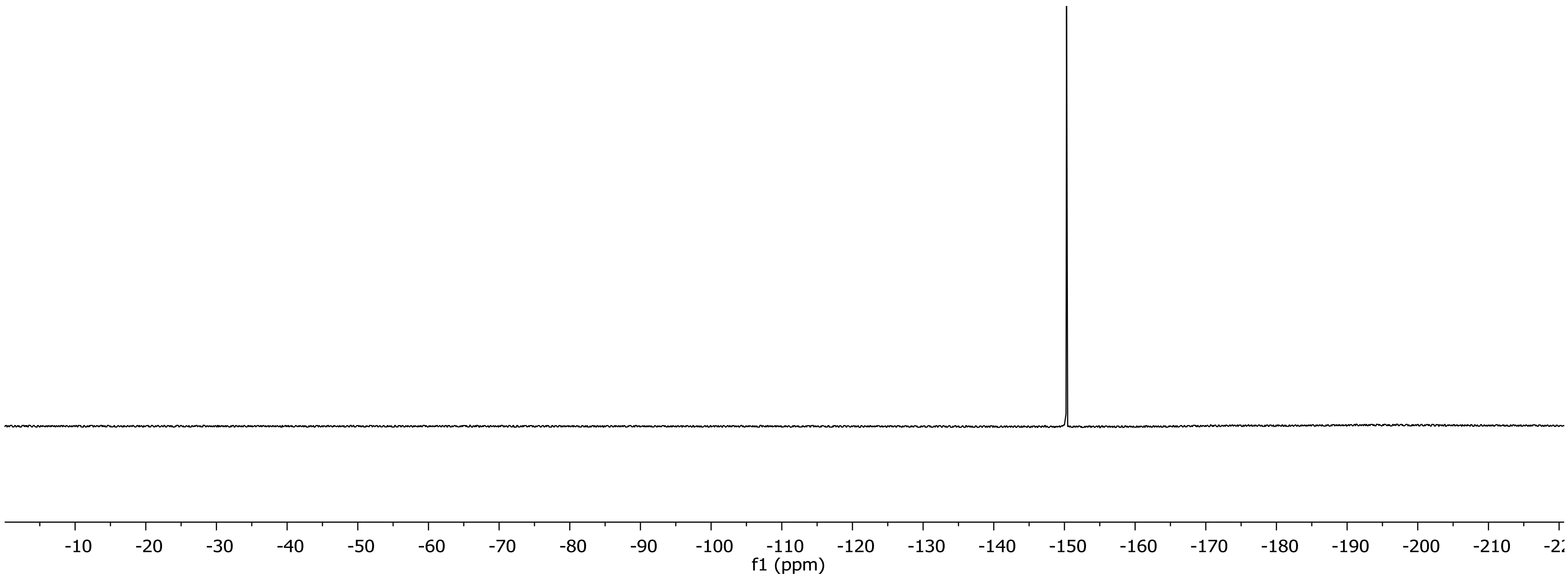

Bicalutamide-derived thianthrenium salt 33-TT

^{1}H NMR of Bicalutamide-derived thianthrenium salt 33-TT

$CDCl_3$, 298 K

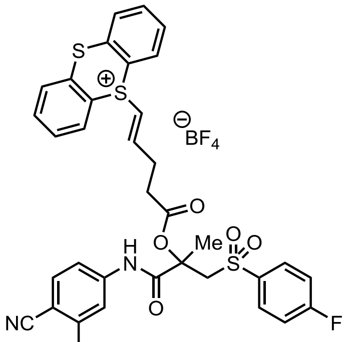

33-TT

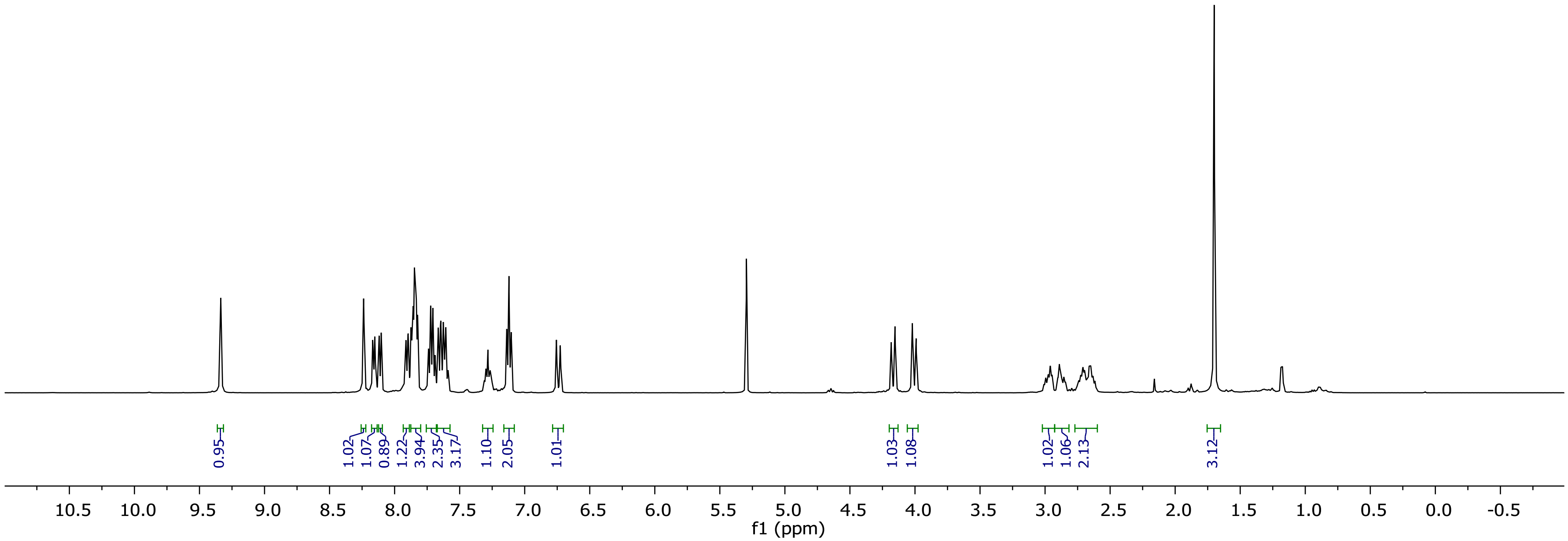

^{13}C NMR of Bicalutamide-derived thianthrenium salt 33-TT

$CDCl_3$, 298 K

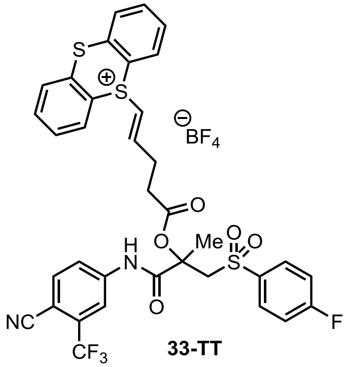

33-TT

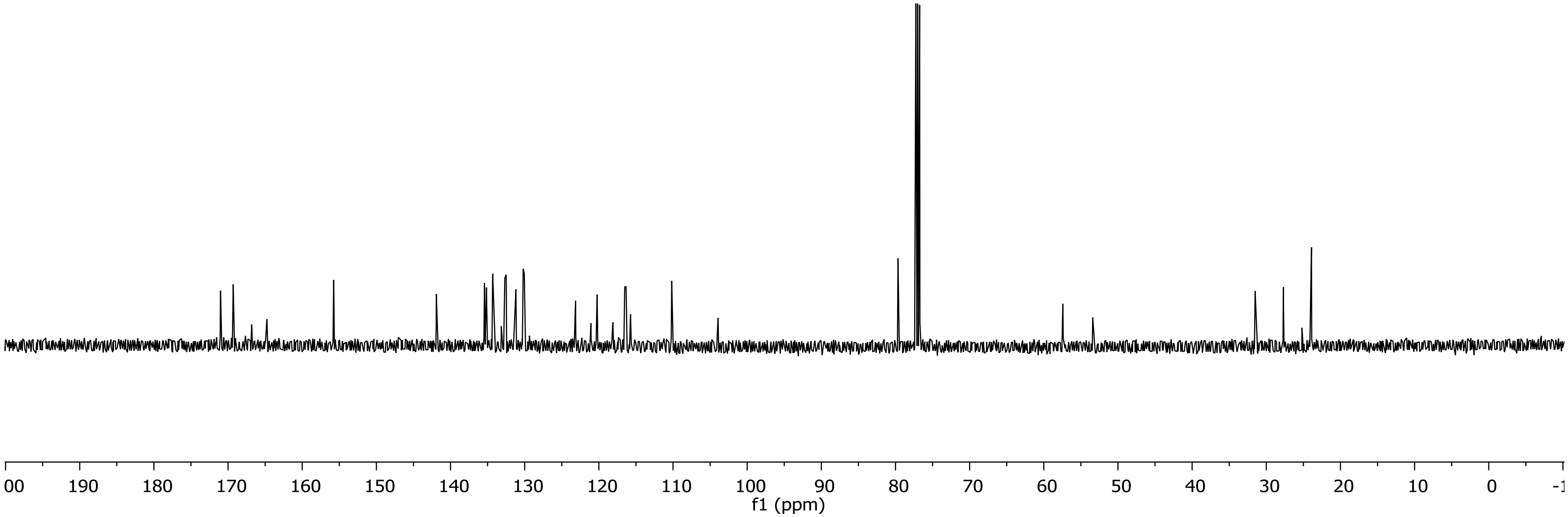

^{19}F NMR of Bicalutamide-derived thianthrenium salt 33-TT

CDCl$_3$, 298 K

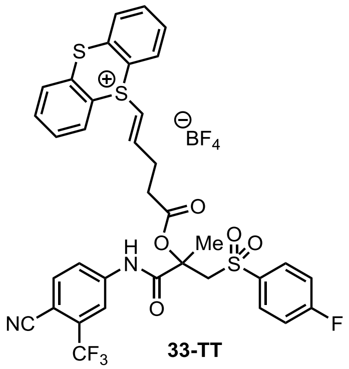

33-TT

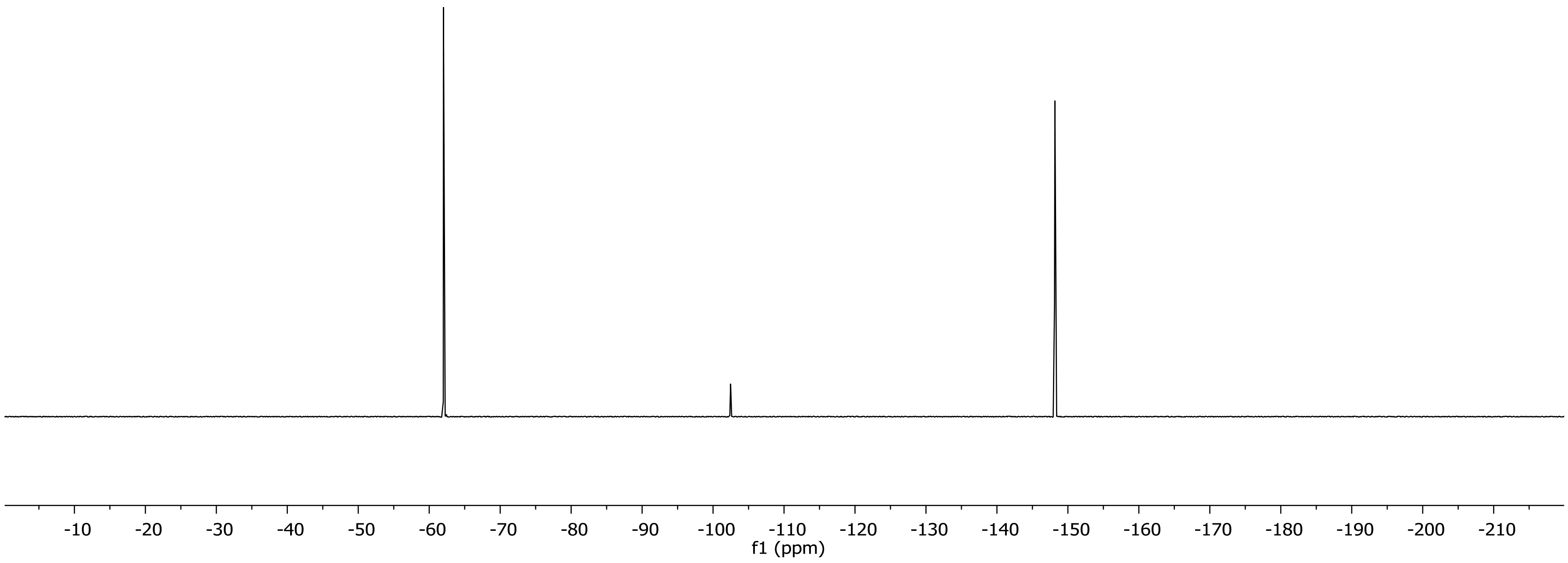

Cyclopropyl-pent-4-en-1-yl-phthalimide (34)

^{1}H NMR of cyclopropyl-pent-4-en-1-yl-phthalimide (34)

$CDCl_3$, 298 K

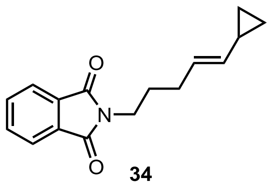

34

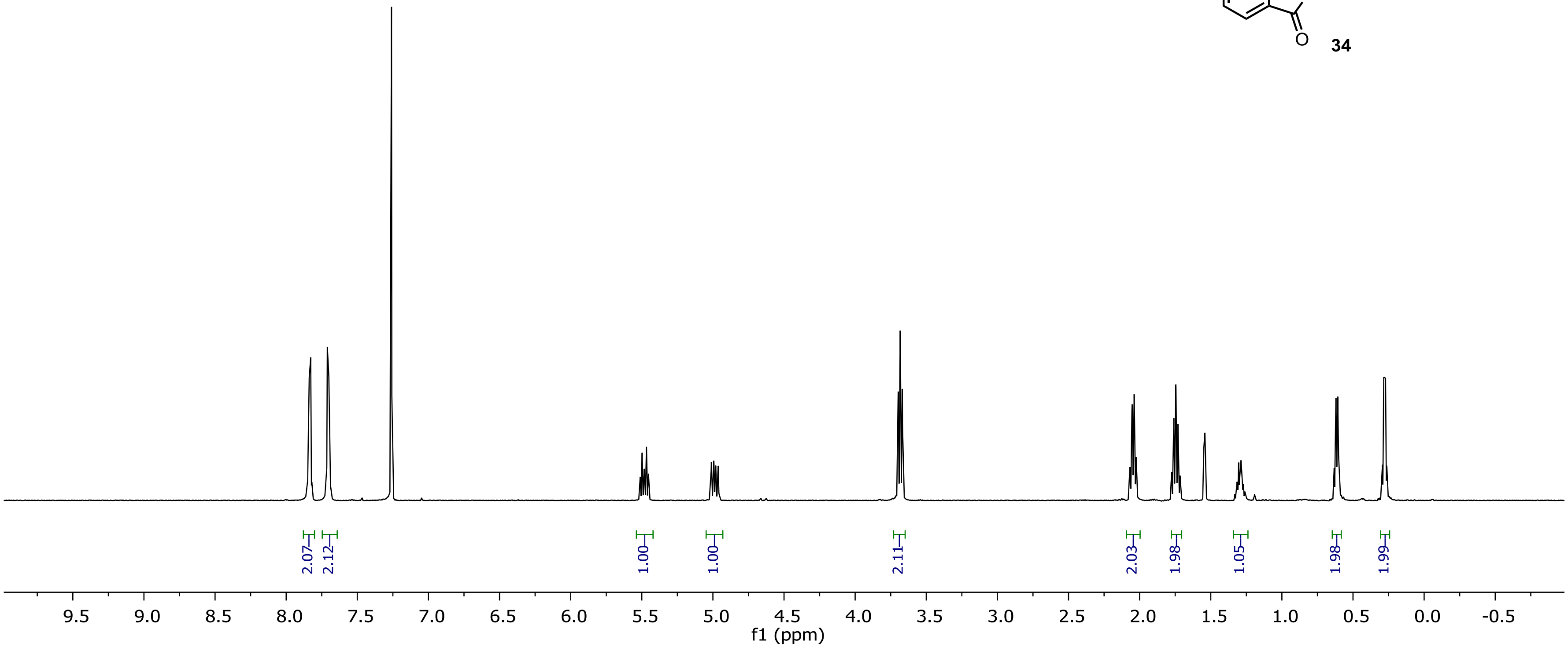

^{13}C NMR of cyclopropyl-pent-4-en-1-yl-phthalimide (34)

$CDCl_3$, 298 K

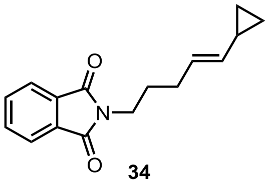

34

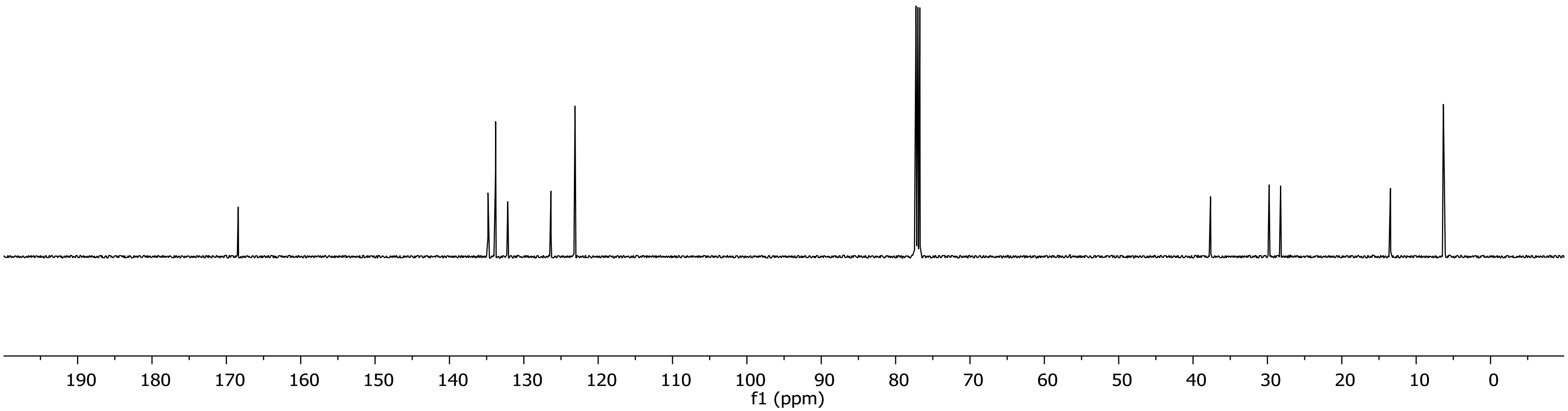

Phenylacetylenyl-pent-4-en-1-yl-phthalimide (35)

^{1}H NMR of phenylacetylenyl-pent-4-en-1-yl-phthalimide (35)

$CDCl_3$, 298 K

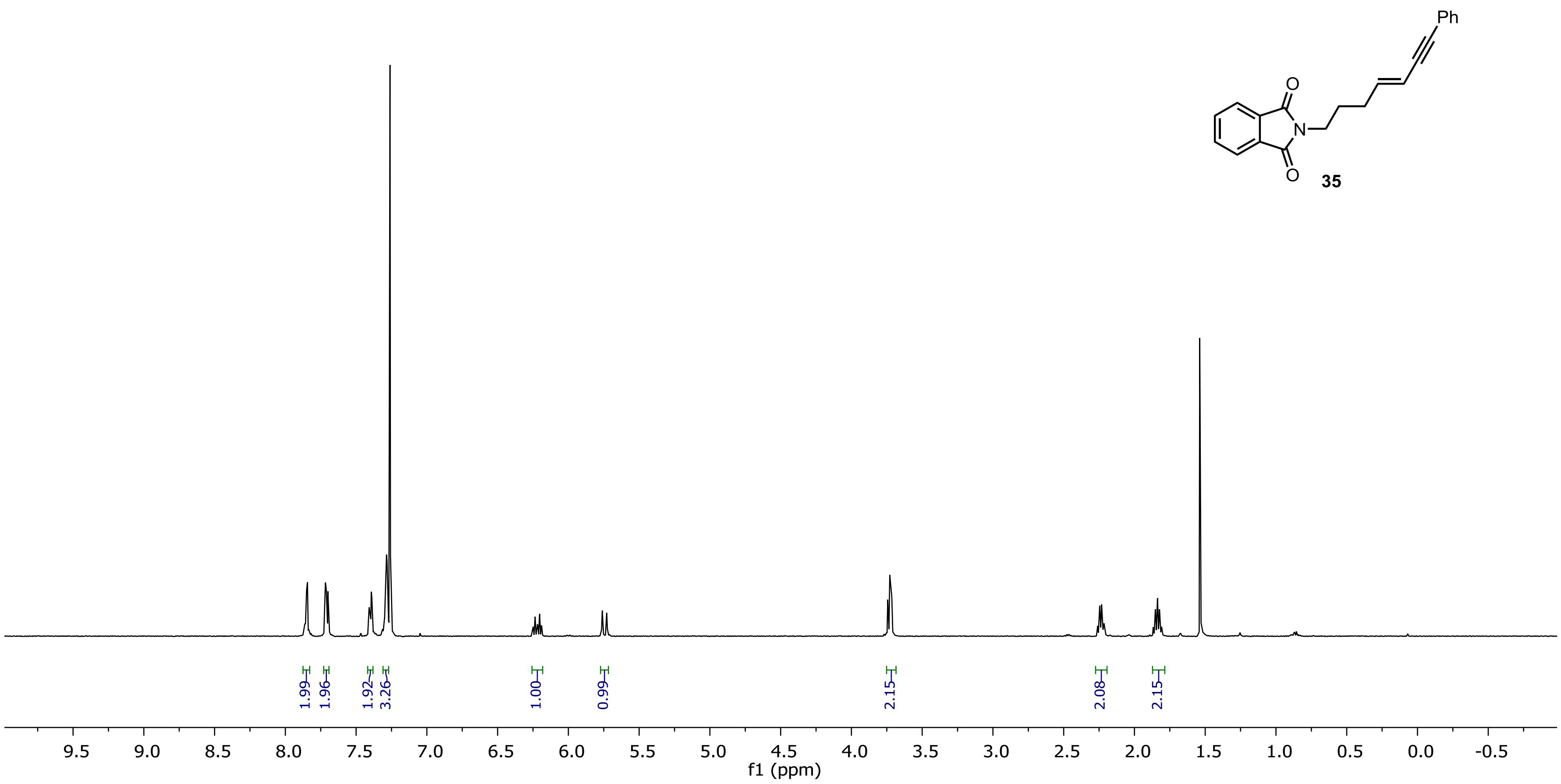

^{13}C NMR of phenylacetylenyl-pent-4-en-1-yl-phthalimide (35)

$CDCl_3$, 298 K

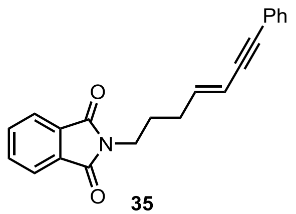

35

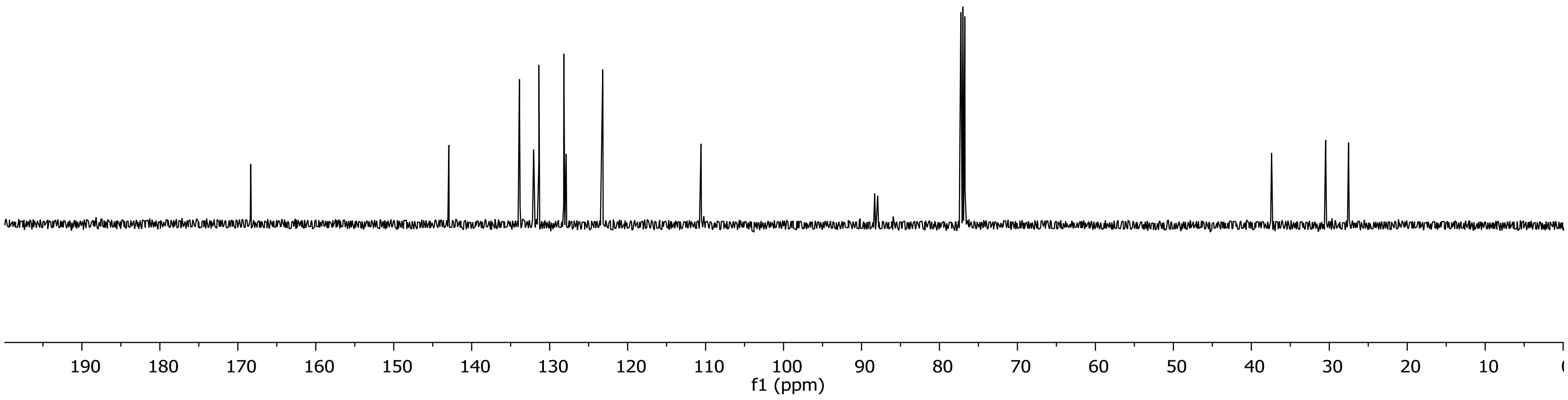

Vinylnaphthalenyl-pent-4-en-1-yl-phthalimide (36)

^{1}H NMR of vinylnaphthalenyl-pent-4-en-1-yl-phthalimide (36)

CD_2Cl_2, 298 K

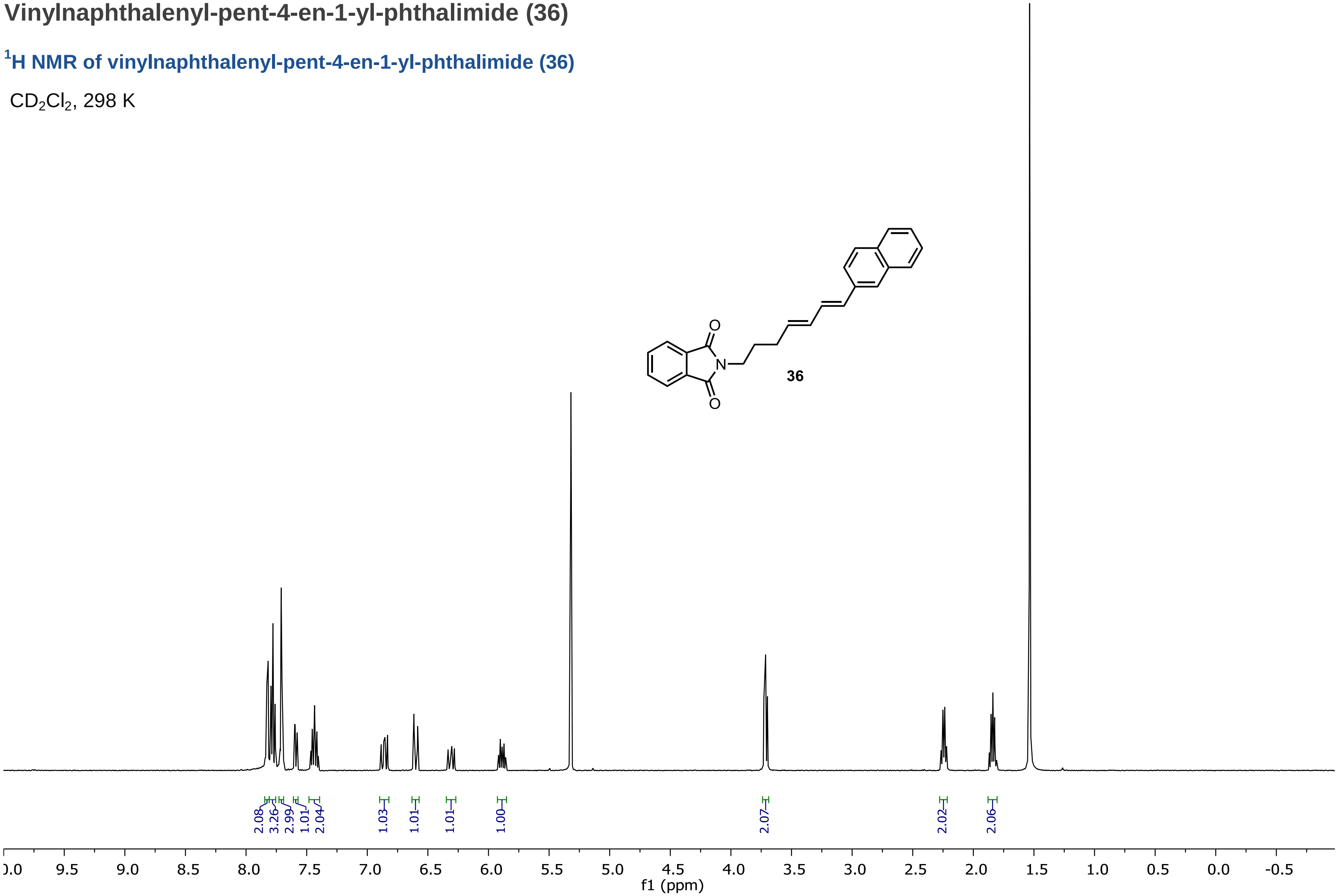

^{13}C NMR of vinylnaphthalenyl-pent-4-en-1-yl-phthalimide (36)

$CDCl_3$, 298 K

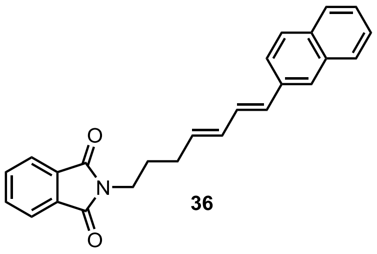

36

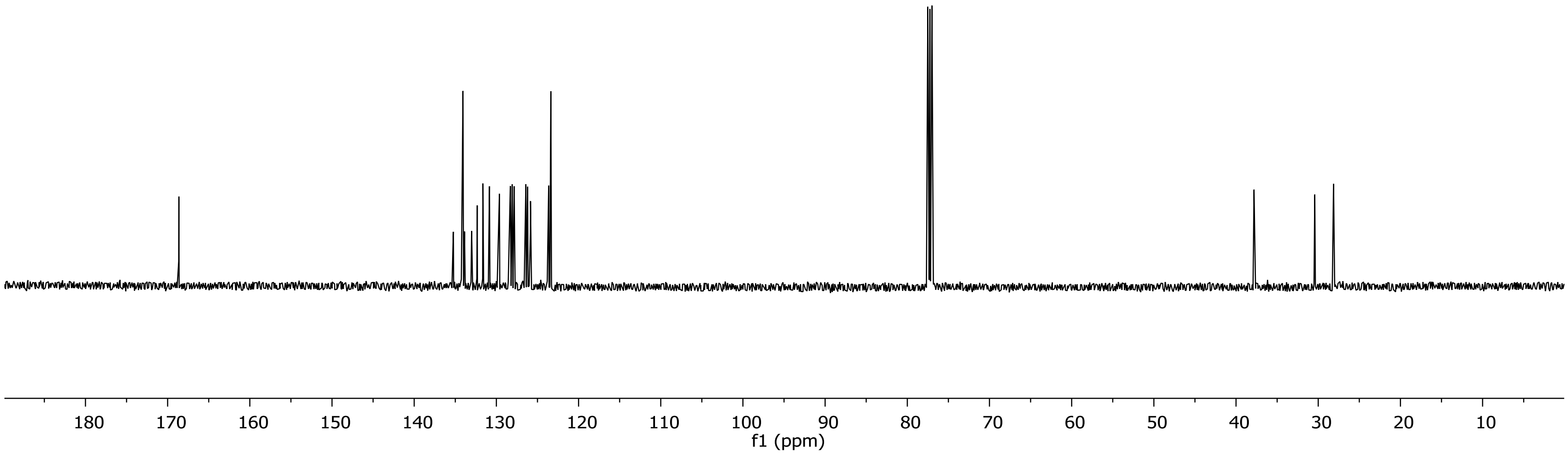

Chloro-pent-4-en-1-yl-phthalimide (37)

^{1}H NMR of chloro-pent-4-en-1-yl-phthalimide (37)

$CDCl_3$, 298 K

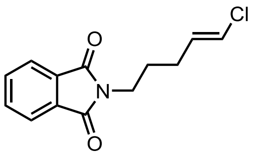

37

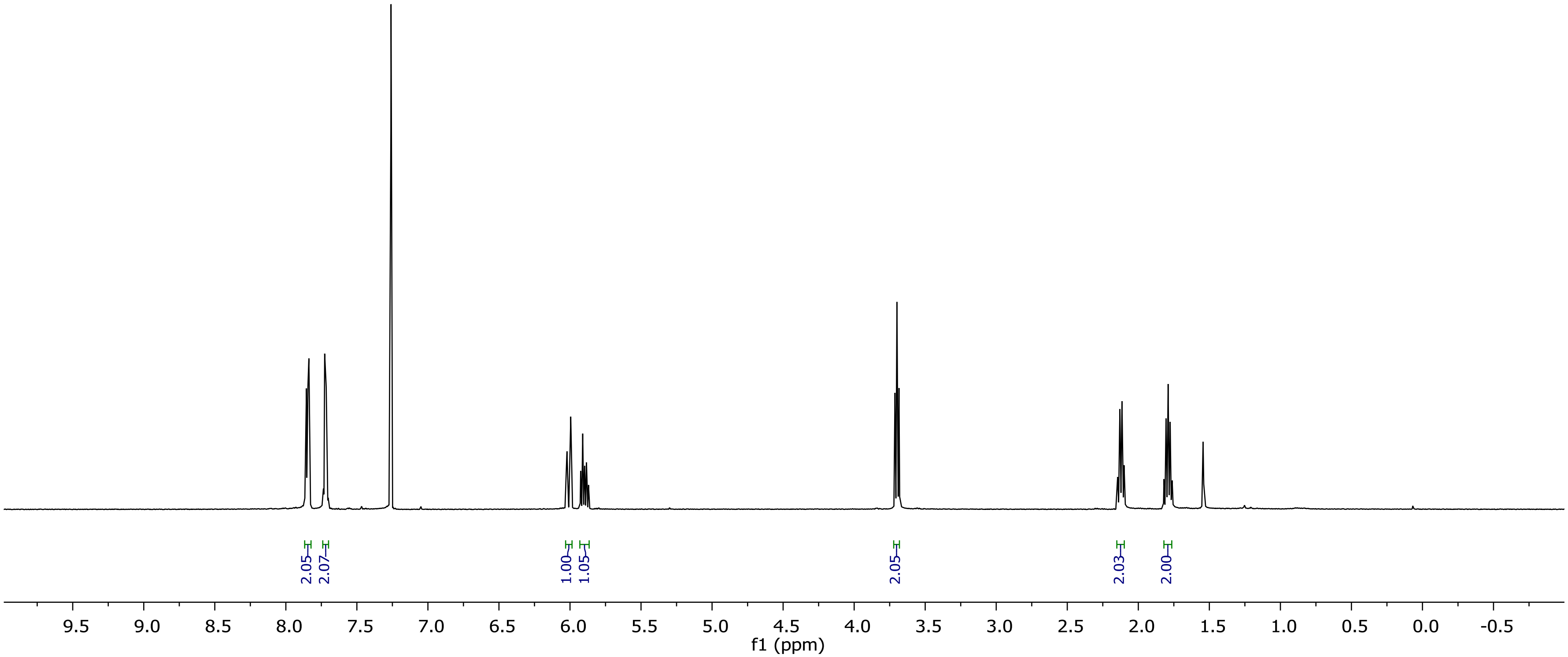

^{13}C NMR of chloro-pent-4-en-1-yl-phthalimide (37)

$CDCl_3$, 298 K

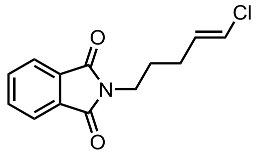

37

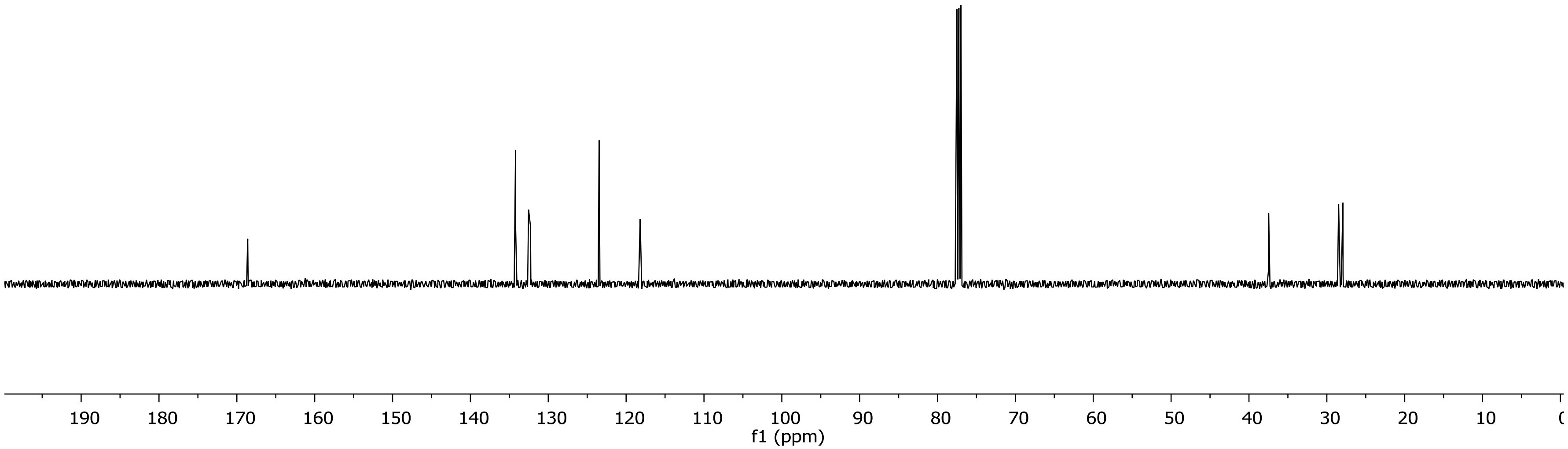

Bromo-pent-4-en-1-yl-phthalimide (38)

^{1}H NMR of bromo-pent-4-en-1-yl-phthalimide (38)

$CDCl_3$, 298 K

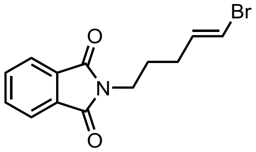

38

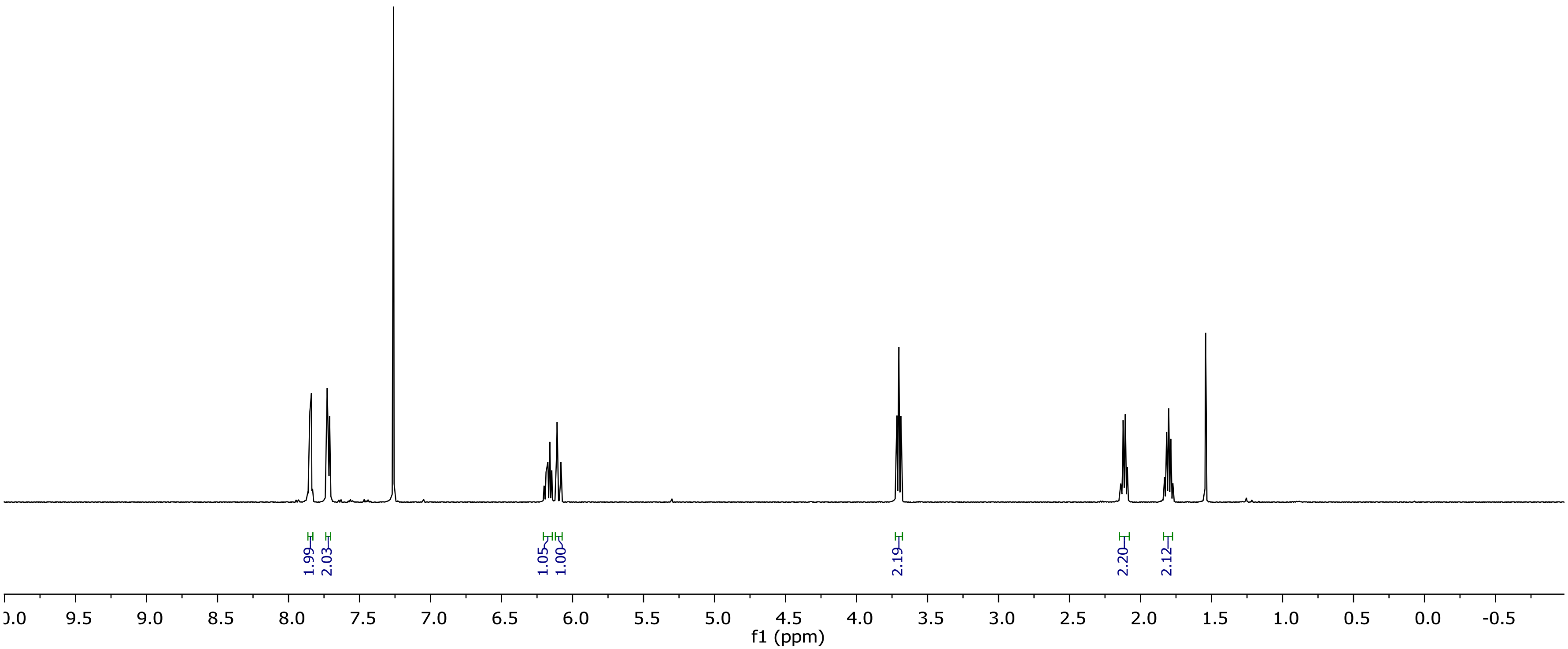

^{13}C NMR of bromo-pent-4-en-1-yl-phthalimide (38)

$CDCl_3$, 298 K

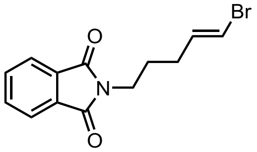

38

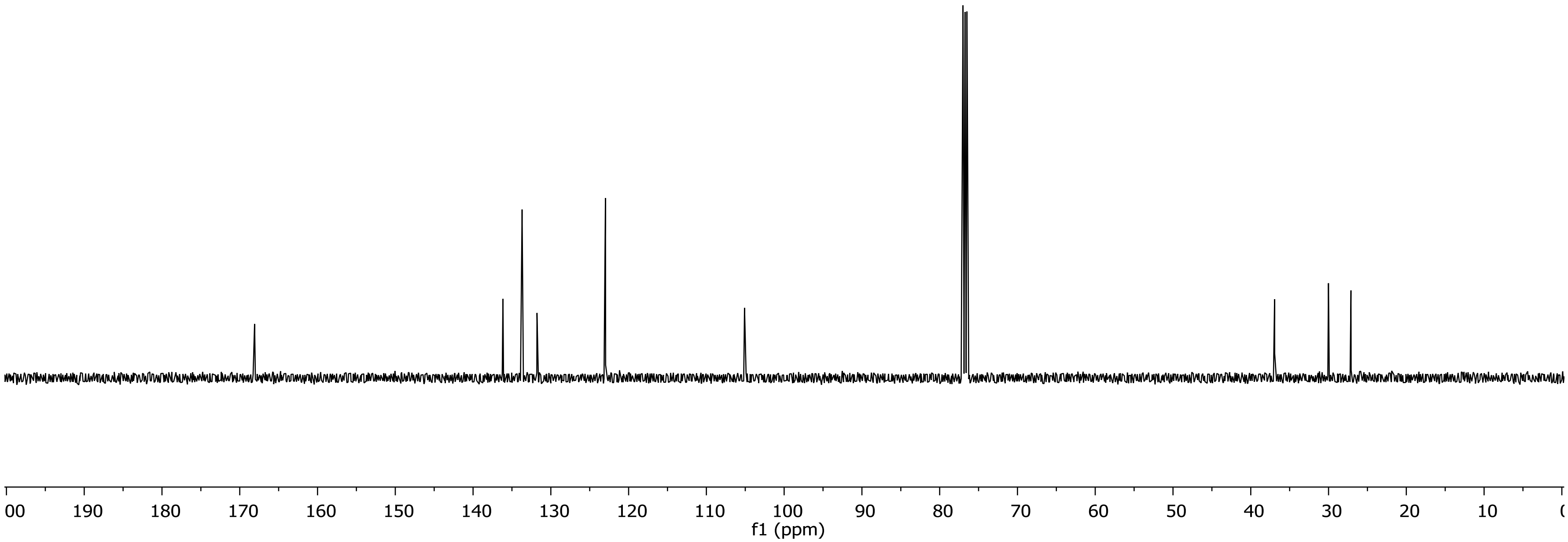

Trifluoromethylsulfuryl-pent-4-en-1-yl-phthalimide (39)

^{1}H NMR of trifluoromethylsulfuryl-pent-4-en-1-yl-phthalimide (39)

$CDCl_3$, 298 K

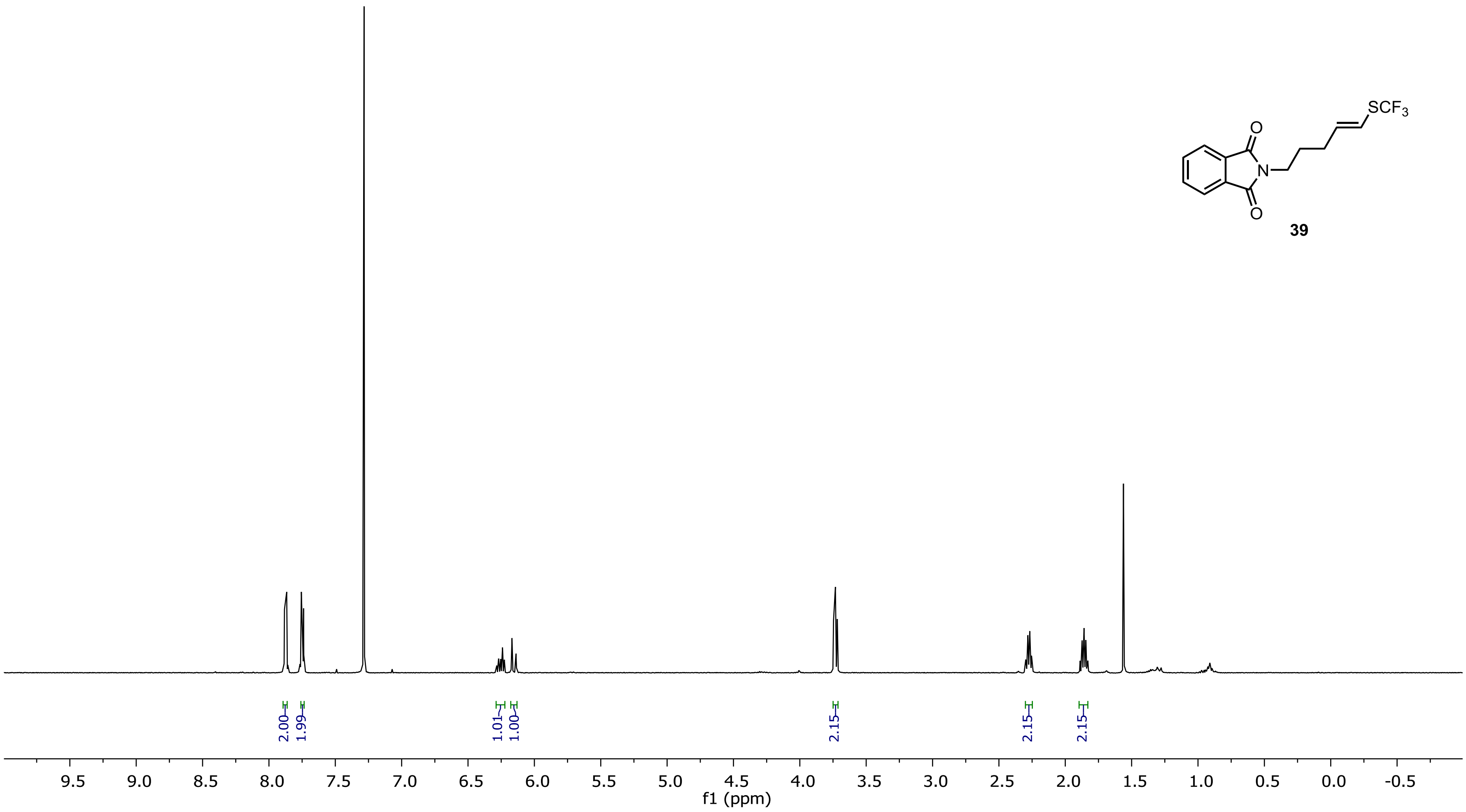

^{13}C NMR of trifluoromethylsulfuryl-pent-4-en-1-yl-phthalimide (39)

$CDCl_3$, 298 K

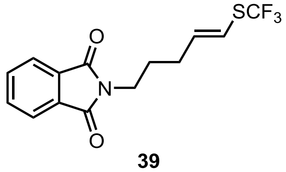

39

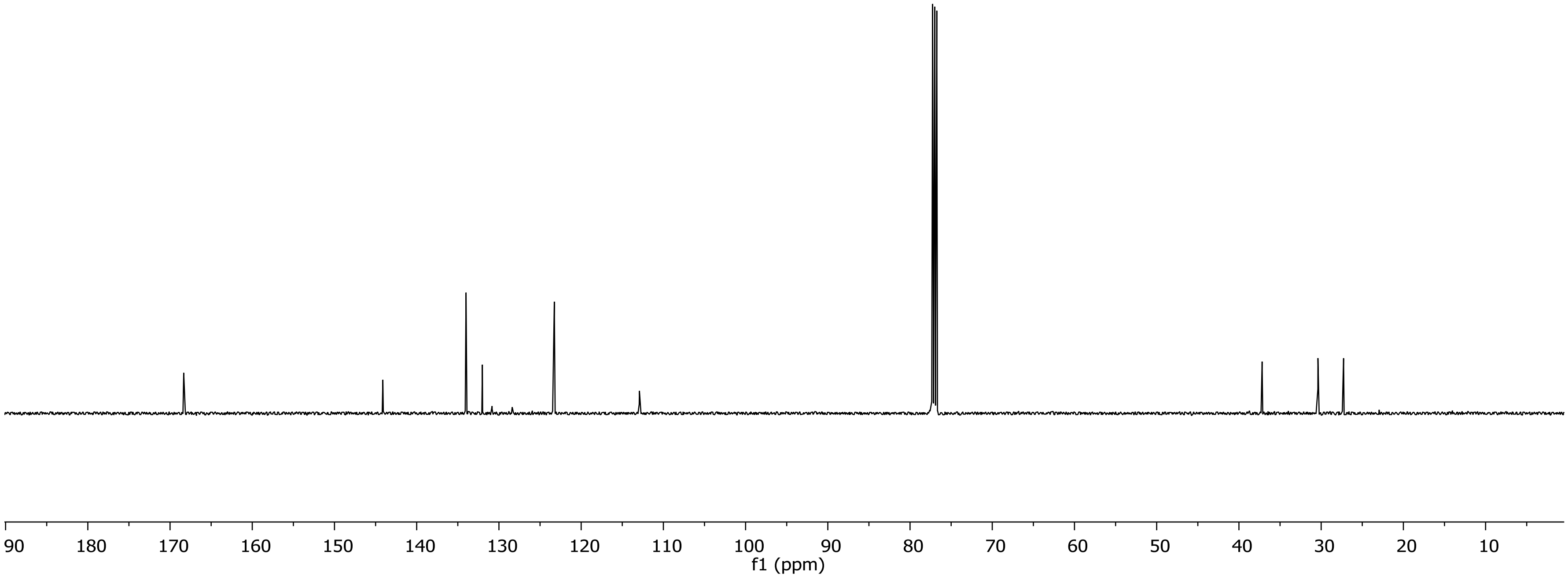

^{19}F NMR of trifluoromethylsulfuryl-pent-4-en-1-yl-phthalimide (39)

$CDCl_3$, 298 K

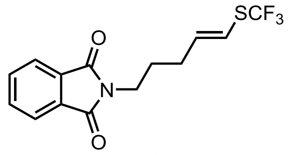

39

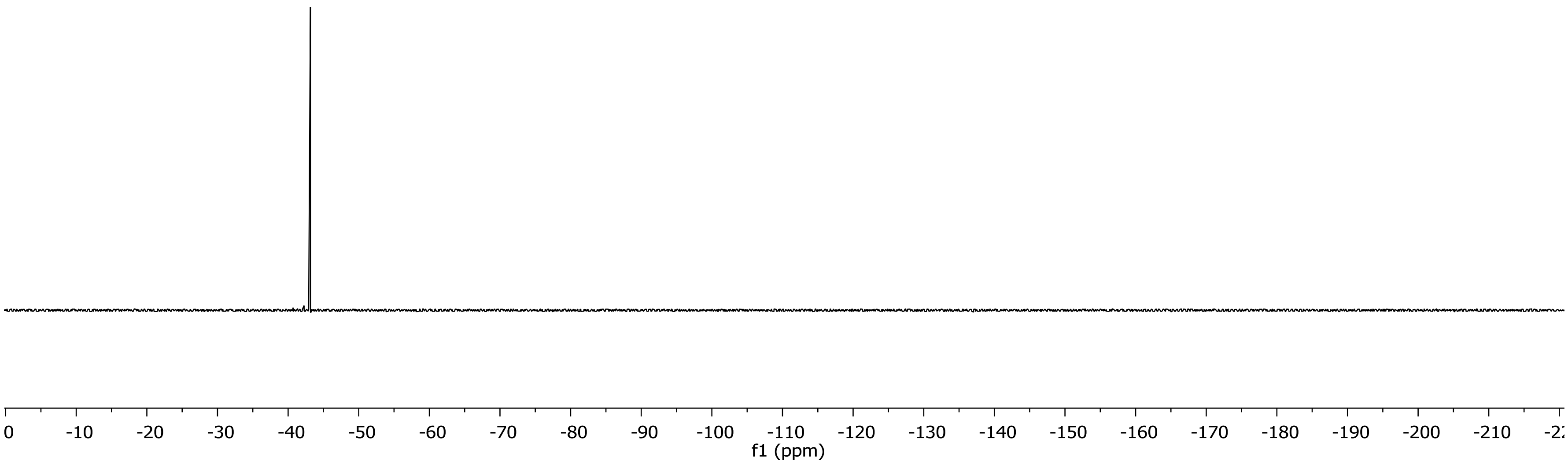

Phenylacetylenyl-4-octene (40-*Z*)

^{1}H NMR of phenylacetylenyl-4-octene (40-*Z*)

$CDCl_3$, 298 K

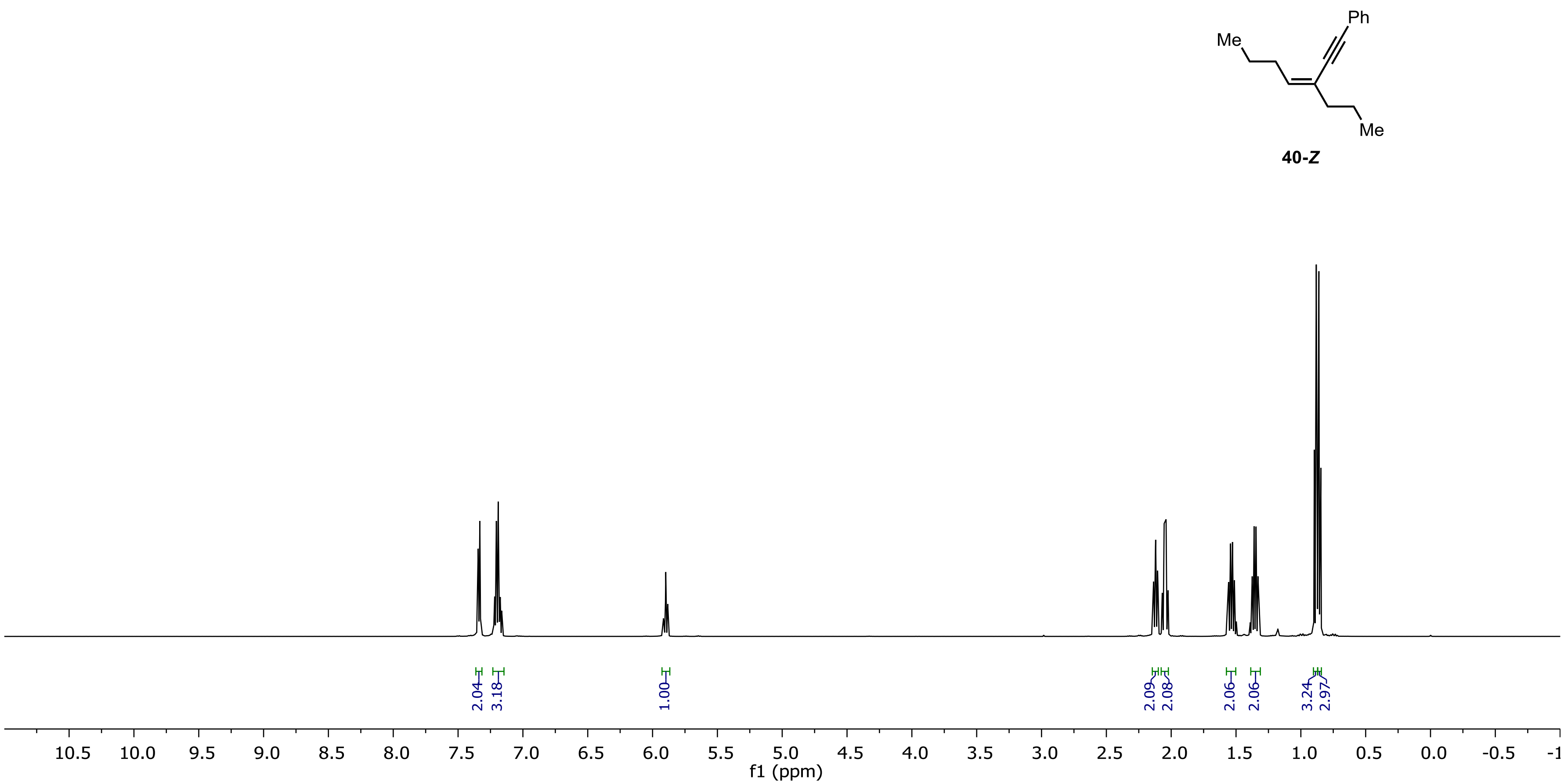

^{13}C NMR of phenylacetylenyl-4-octene (40-*Z*)

CDCl$_3$, 298 K

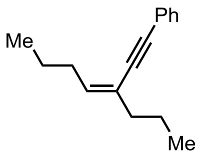

40-*Z*

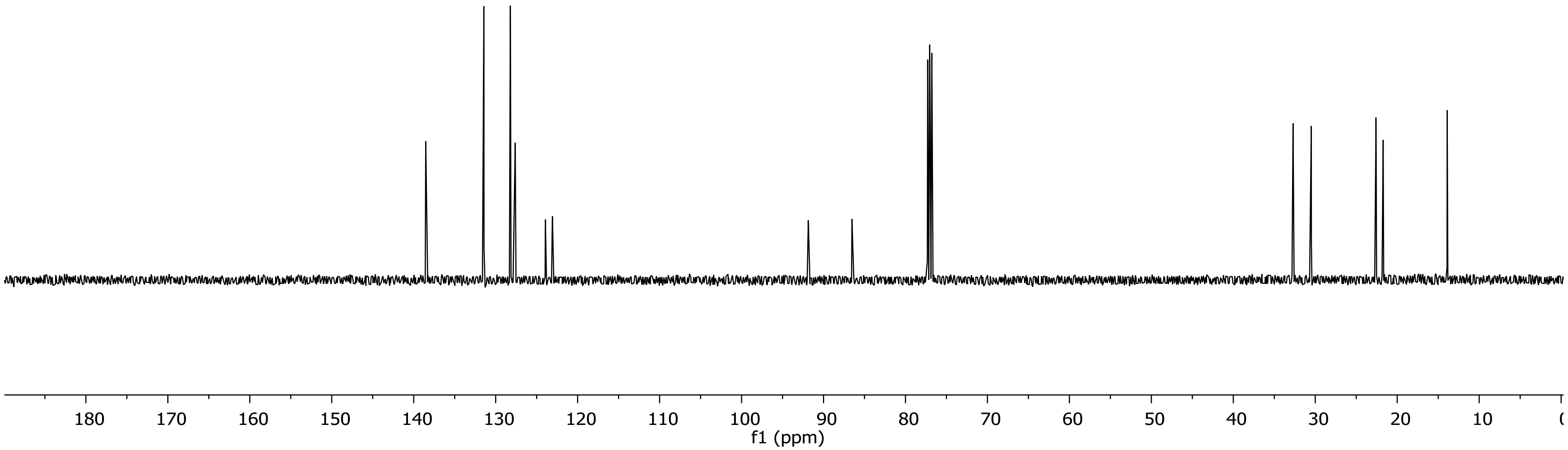

Phenylacetylenyl-4-octene (40-*E*)

^{1}H NMR of phenylacetylenyl-4-octene (40-*E*)

$CDCl_3$, 298 K

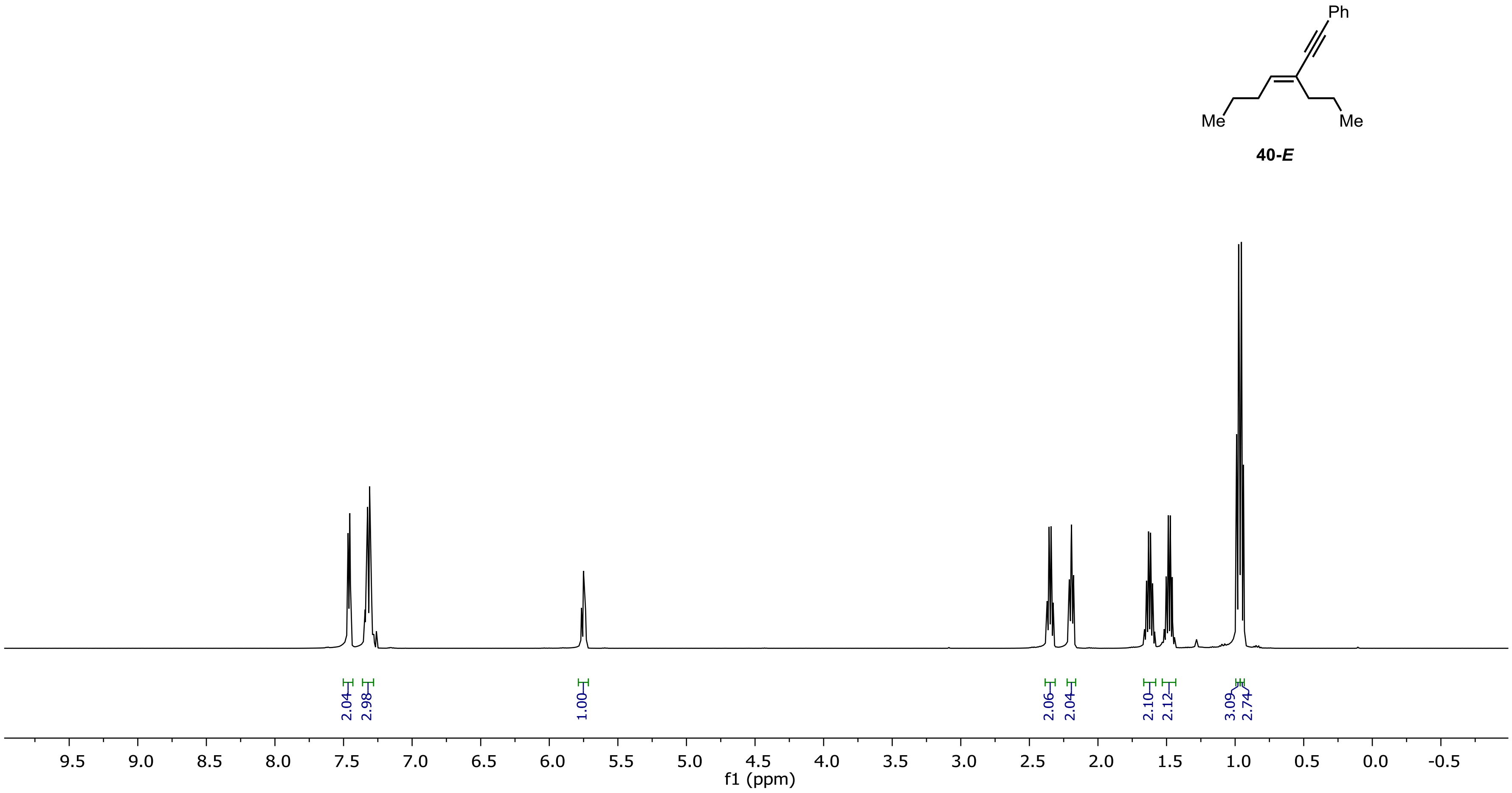

^{13}C NMR of phenylacetylenyl-4-octene (40-*E*)

CDCl$_3$, 298 K

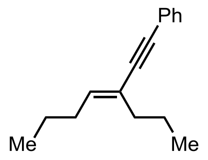

40-*E*

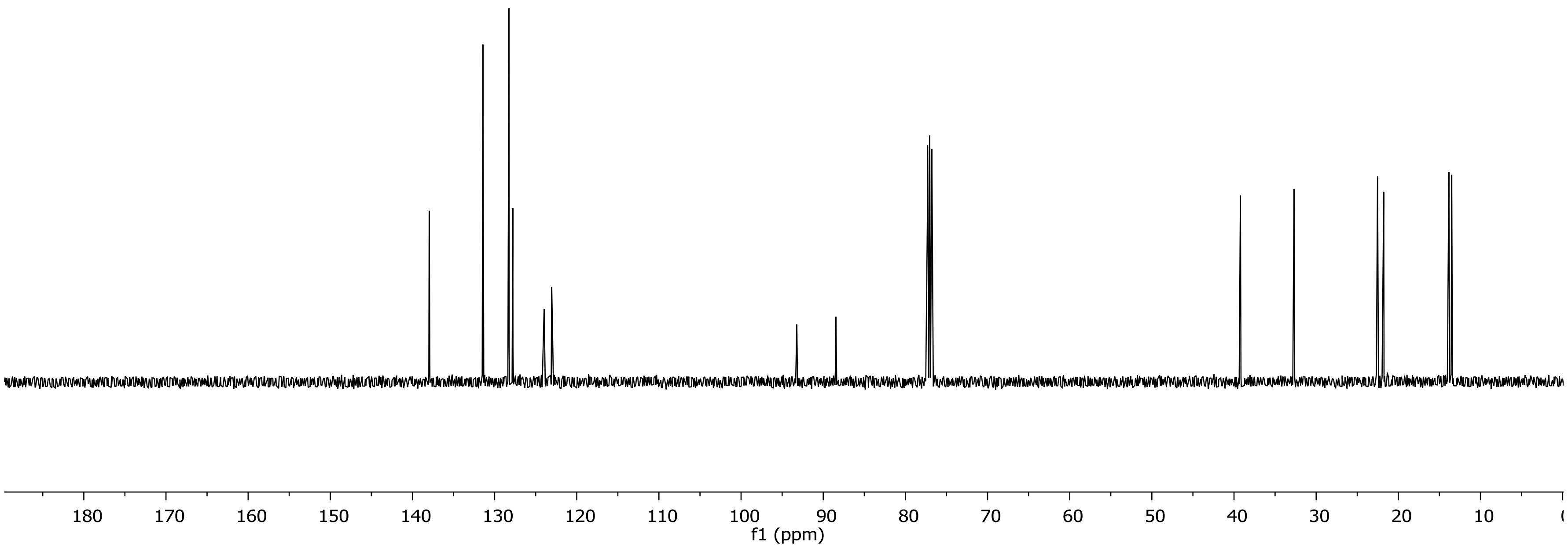

Nicotinic acid-derived alkene 26

^{1}H NMR of Nicotinic acid-derived alkene 26

CDCl$_3$, 298 K

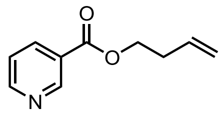

26

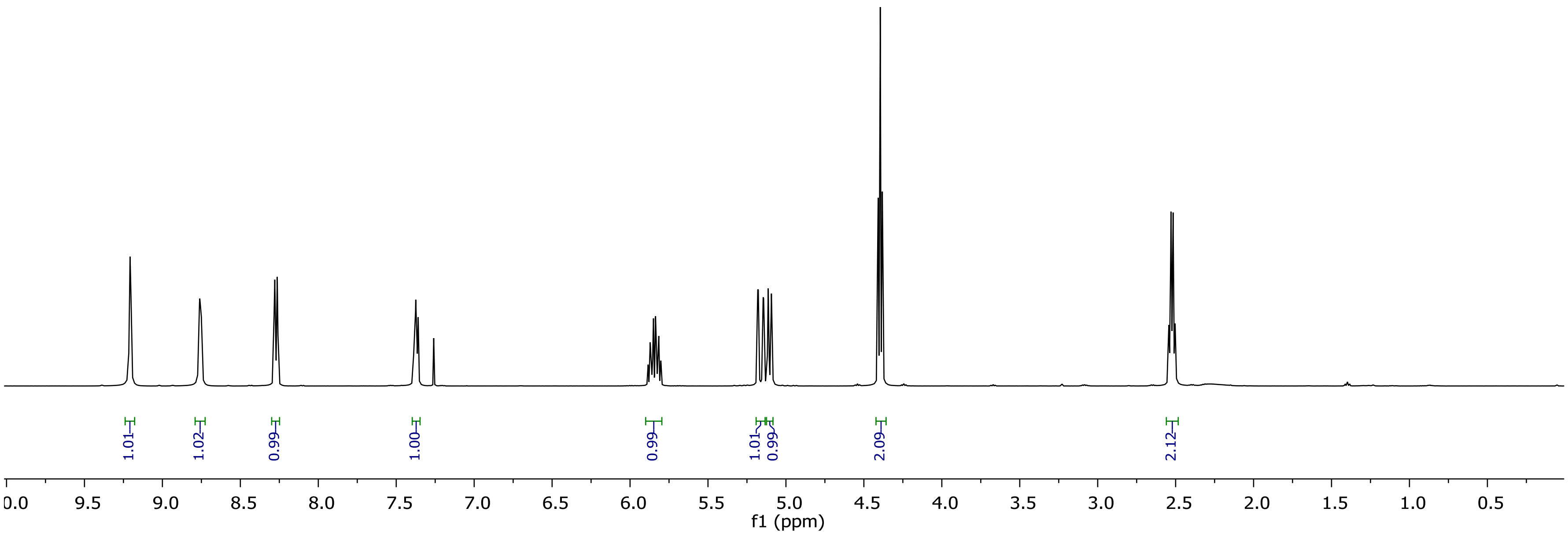

^{13}C NMR of Nicotinic acid-derived alkene 26

$CDCl_3$, 298 K

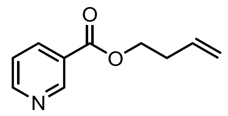

26

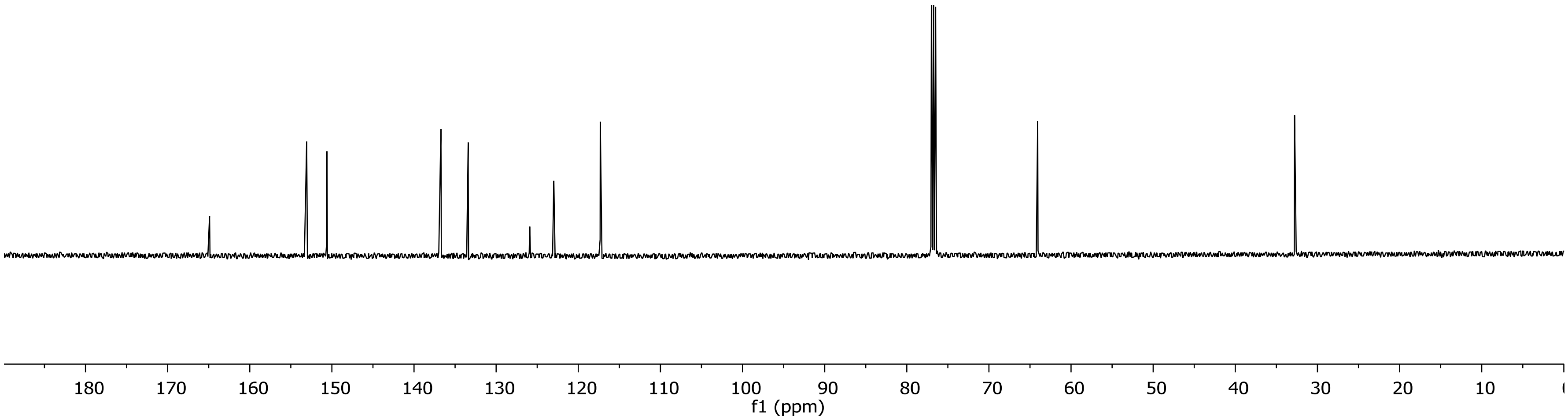

Cinchophene-derived alkene 27

^{1}H NMR of cinchophene-derived alkene 27

$CDCl_3$, 298 K

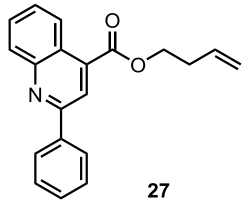

27

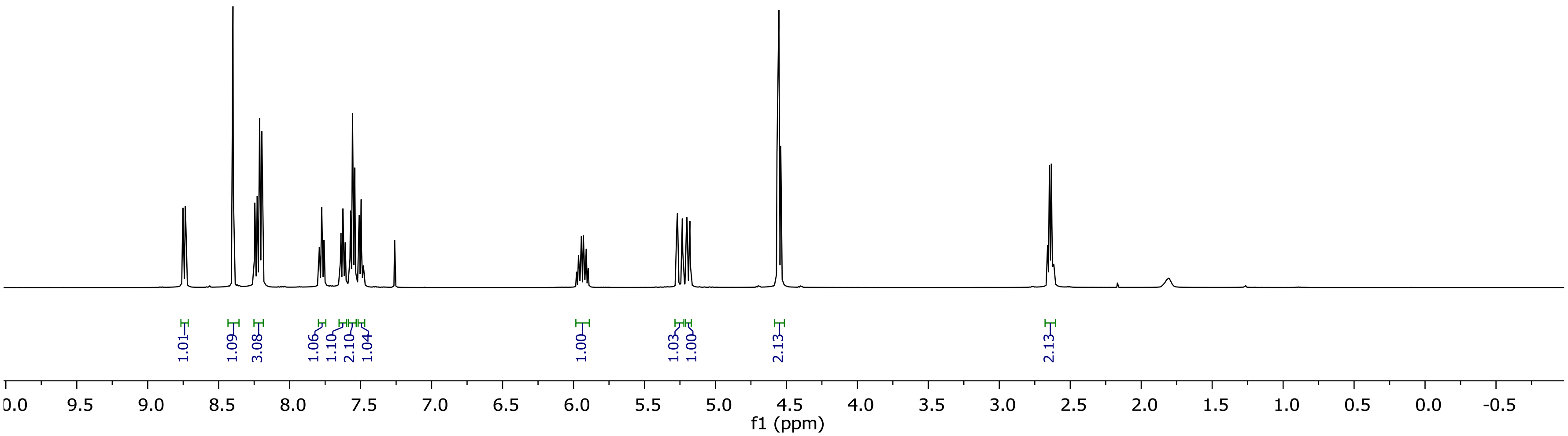

^{13}C NMR of cinchophene-derived alkene 27

$CDCl_3$, 298 K

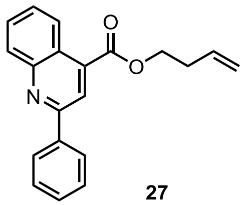

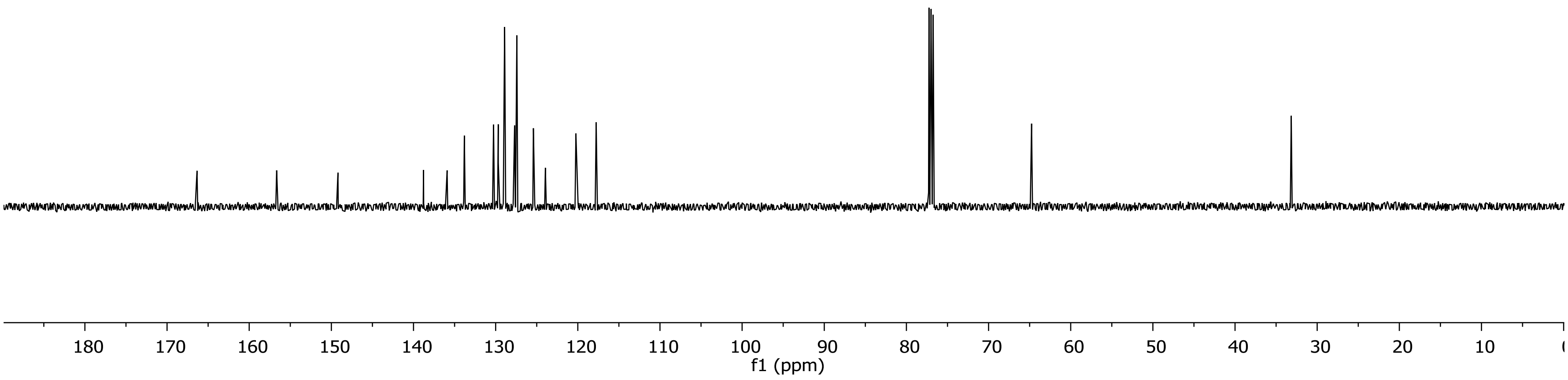

Lithocholic acid-derived alkene 28

^{1}H NMR of lithocholic acid-derived alkene 28

$CDCl_3$, 298 K

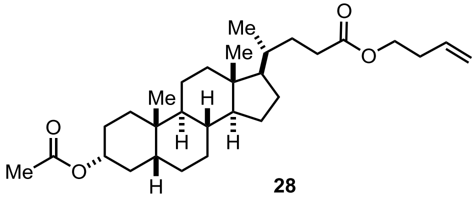

28

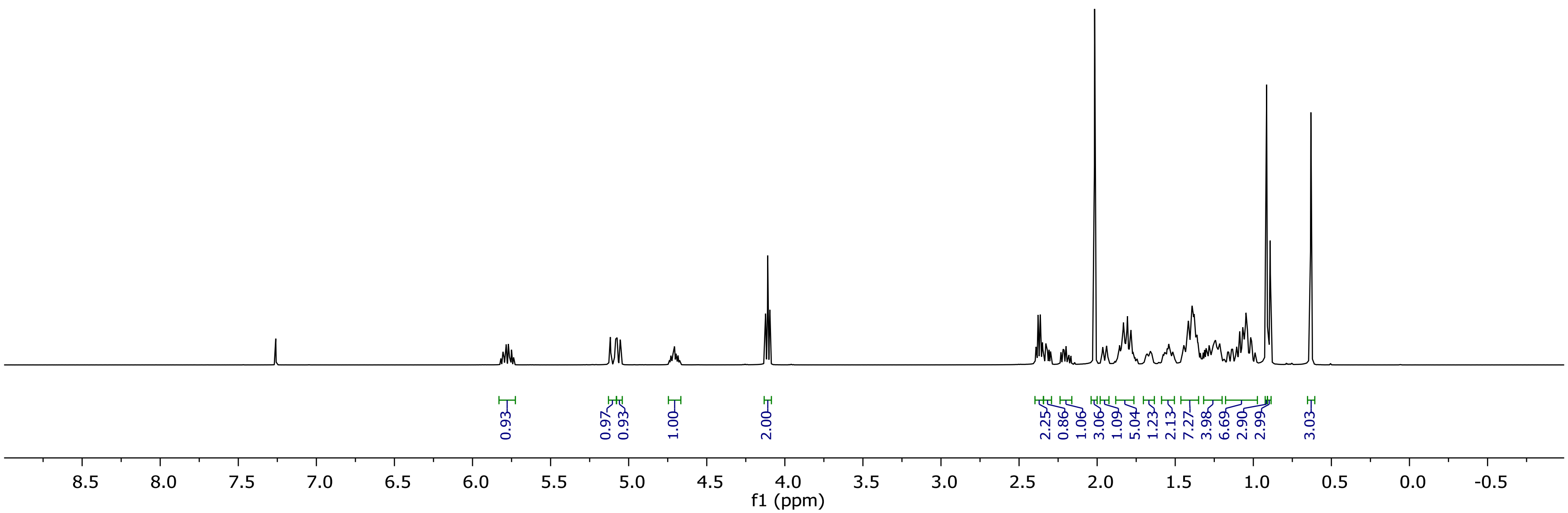

^{13}C NMR of lithocholic acid-derived alkene 28

CDCl$_3$, 298 K

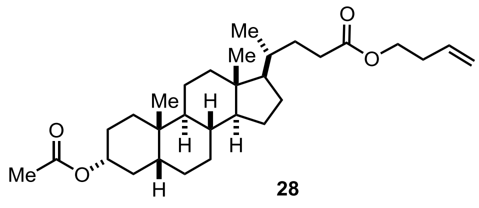

28

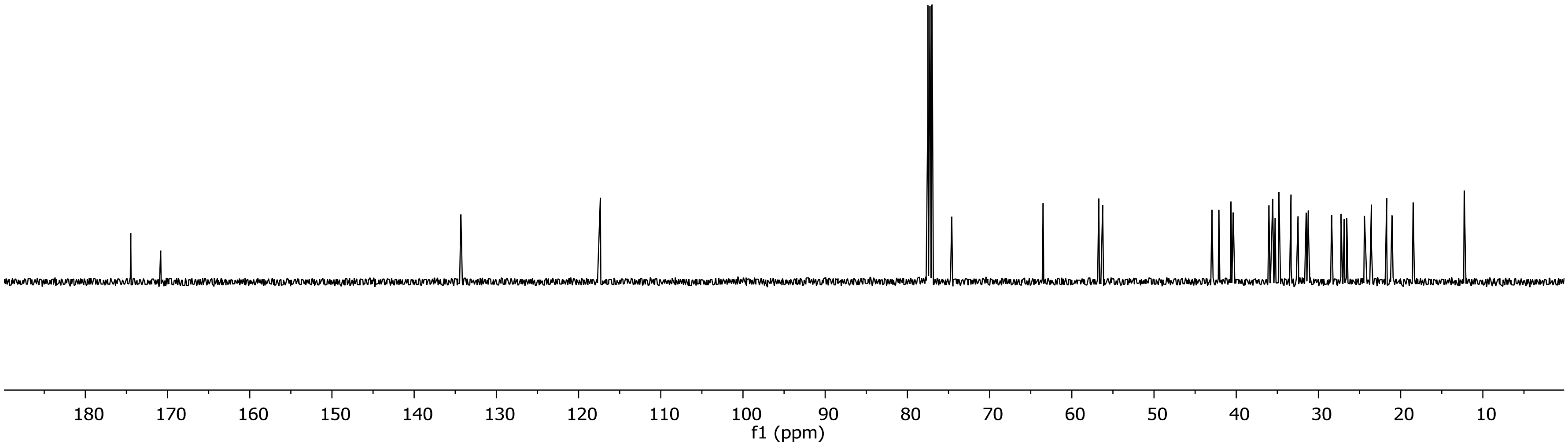

Epiandrosterone-derived alkene 29

^{1}H NMR of epiandrosterone-derived alkene 29

$CDCl_3$, 298 K

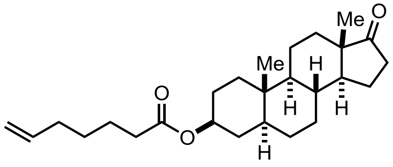

29

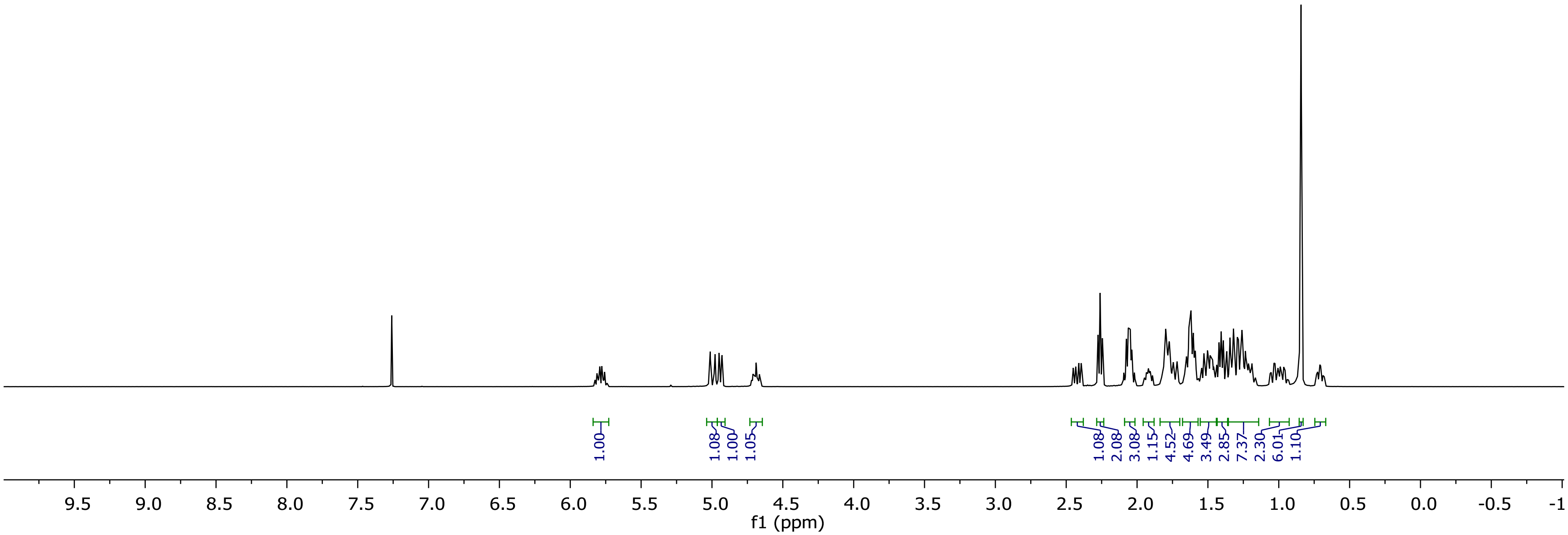

^{13}C NMR of epiandrosterone-derived alkene 29

$CDCl_3$, 298 K

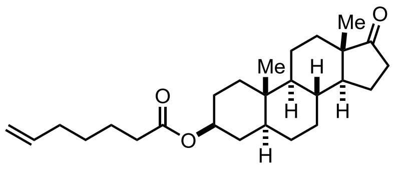

29

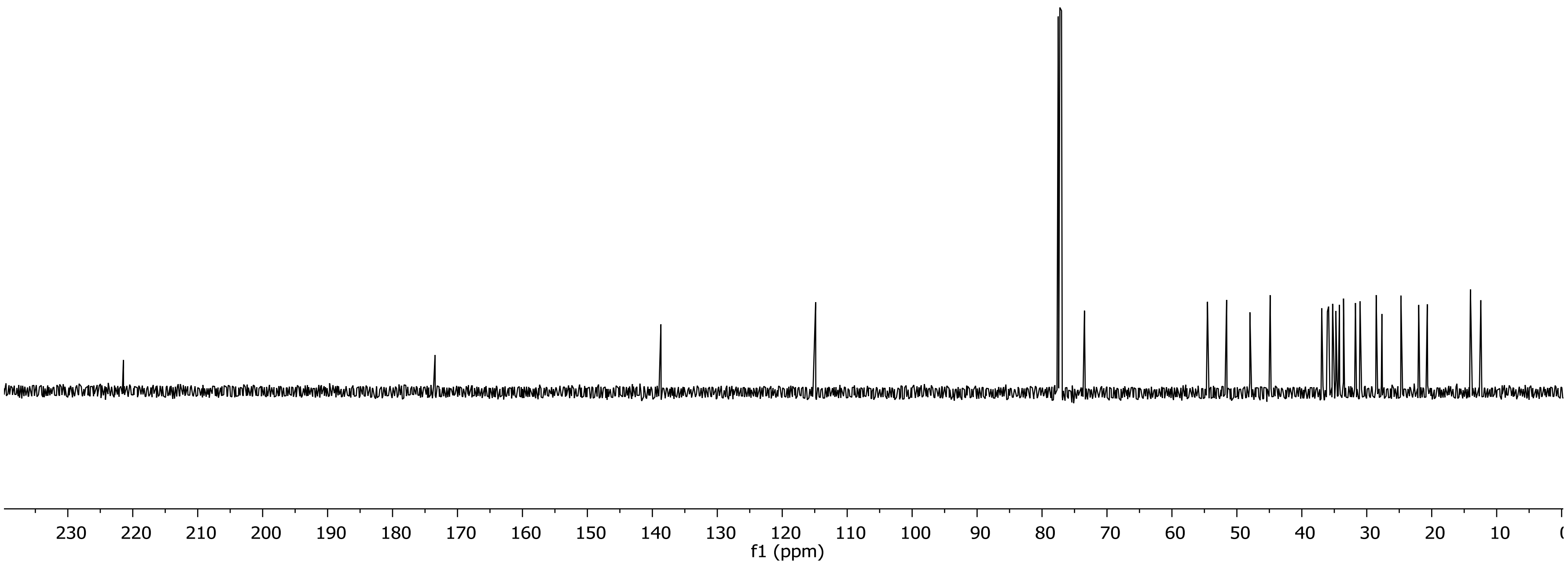

Epiandrosterone-derived alkene 29

^{1}H NMR of epiandrosterone-derived alkene 29

$CDCl_3$, 298 K

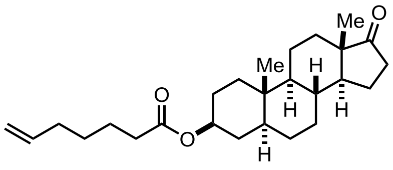

29

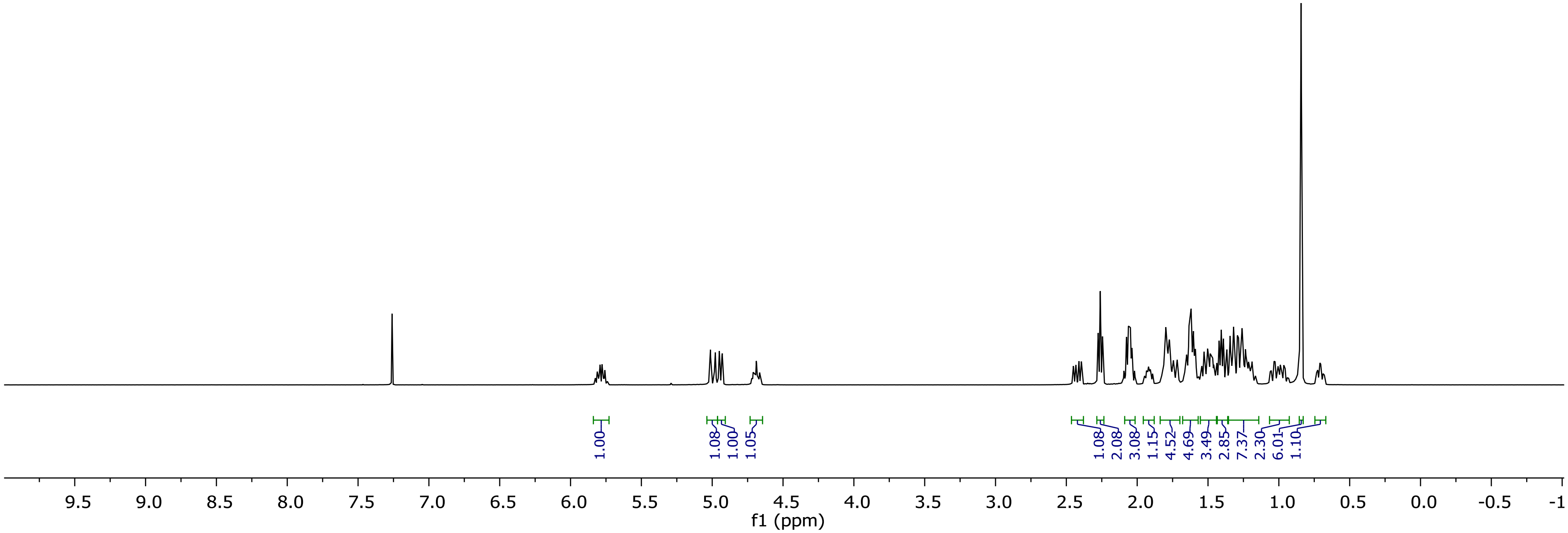

^{13}C NMR of epiandrosterone-derived alkene 29

$CDCl_3$, 298 K

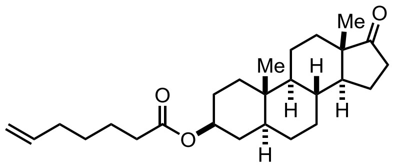

29

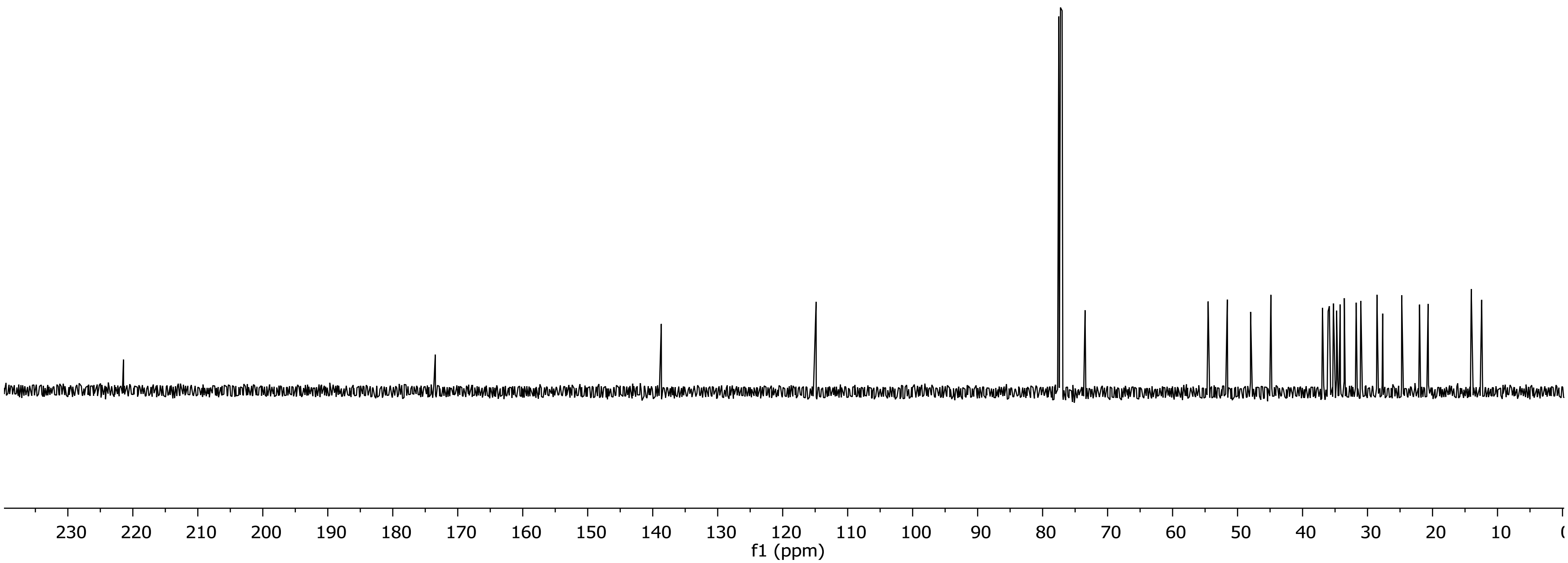

Piperidine-derived alkene 30

^{1}H NMR of piperidine-derived alkene 30

600 MHz, DMSO-d_6, 298 K

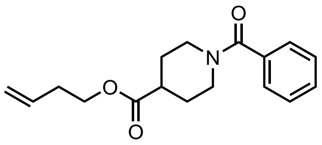

30

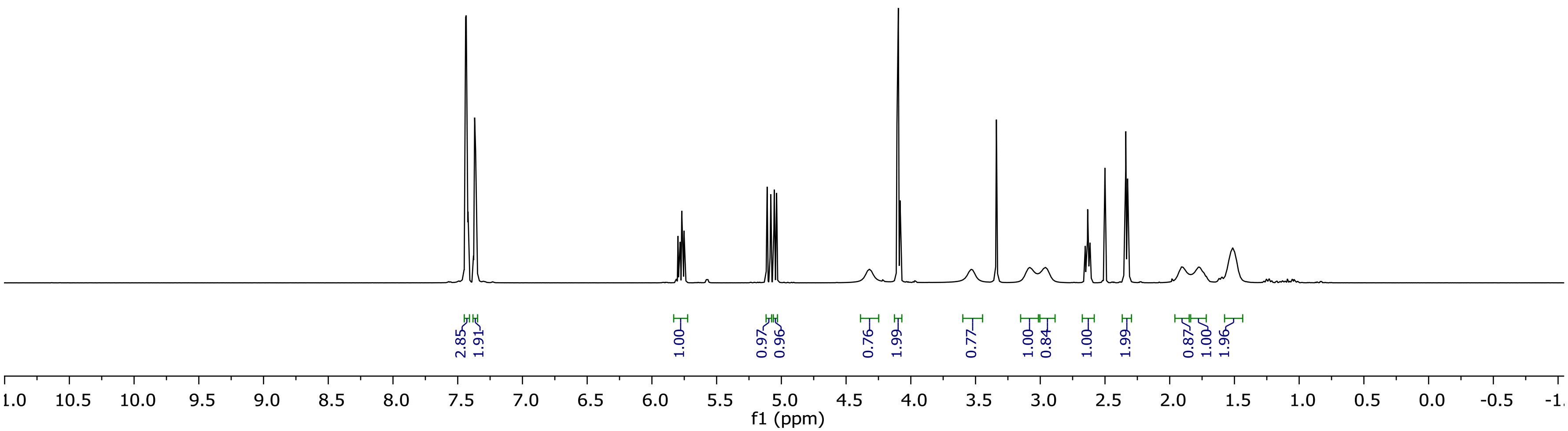

^{13}C NMR of piperidine-derived alkene 30

600 MHz, DMSO-d_6, 298 K

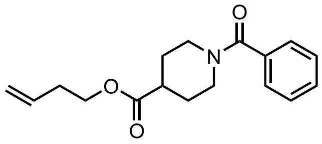

30

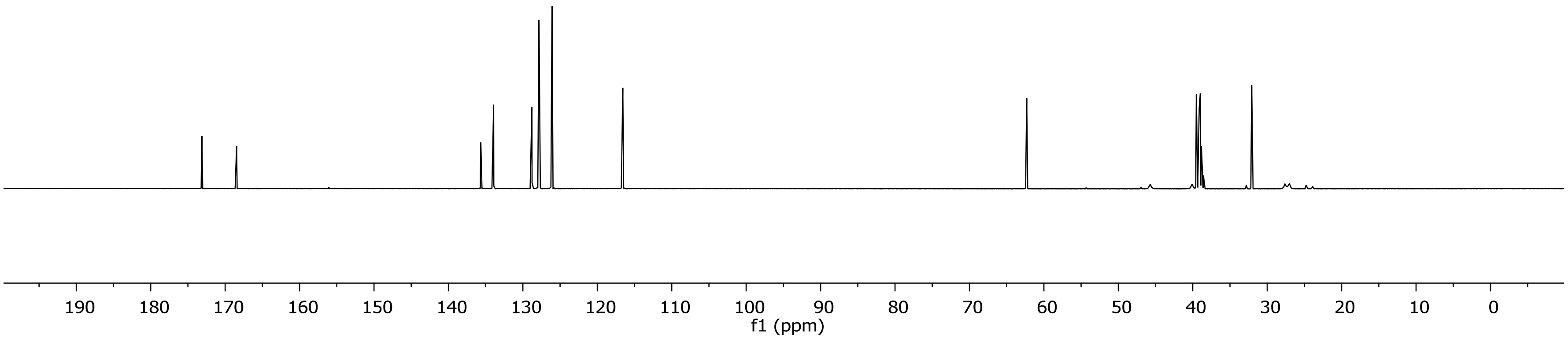

Flazinam-derived alkene 31

^{1}H NMR of Fluazinam-derived alkene 31

$CDCl_3$, 298 K

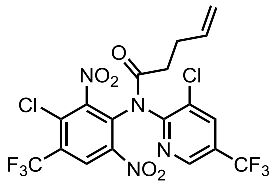

31

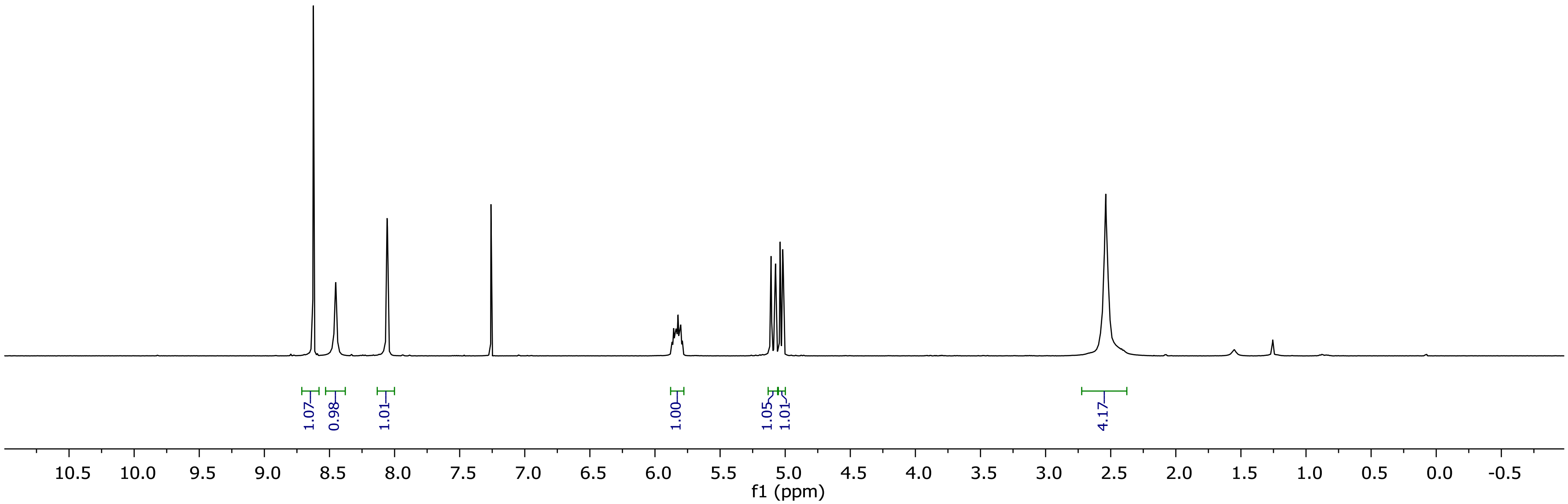

^{13}C NMR of Fluazinam-derived alkene 31

CDCl$_3$, 298 K

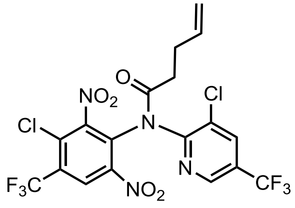

31

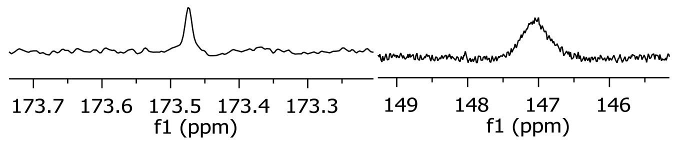

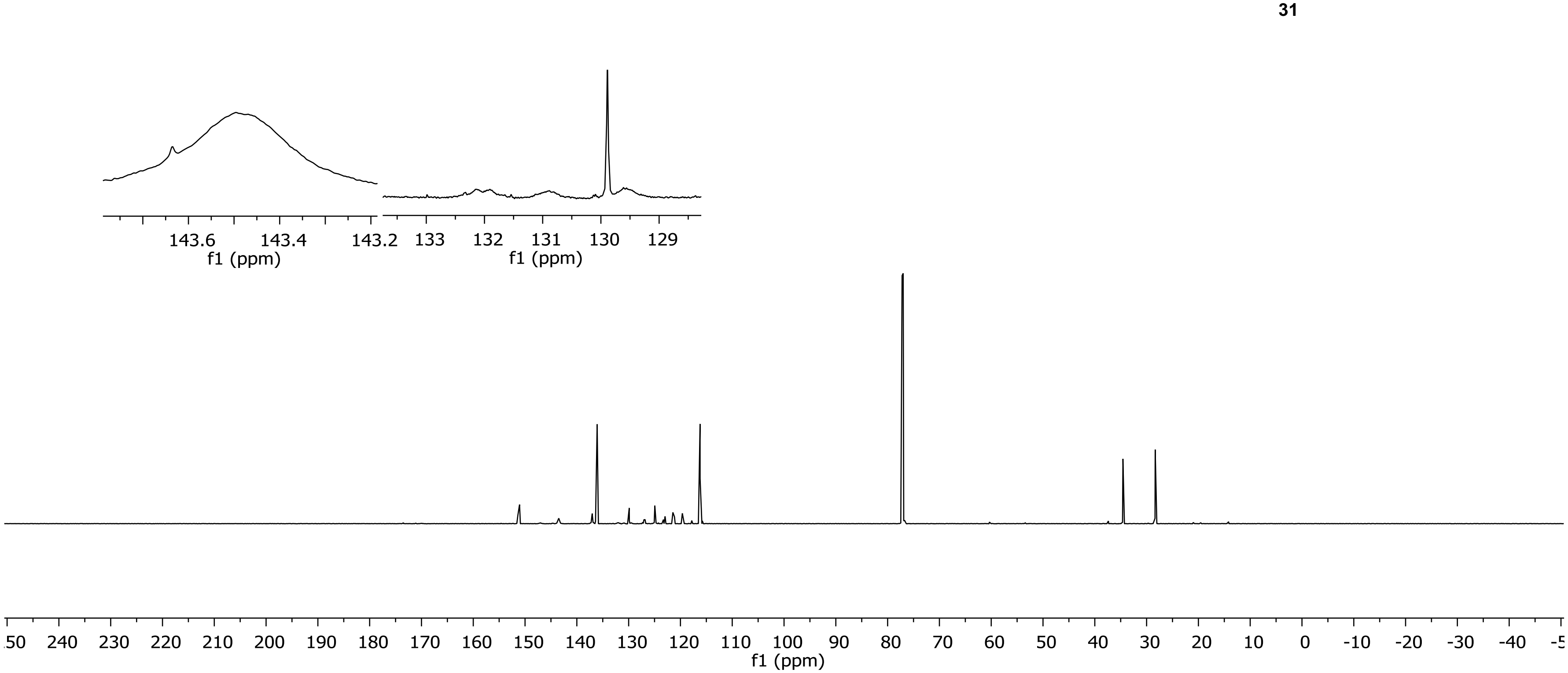

^{19}F NMR of Fluazinam-derived alkene 31

$CDCl_3$, 298 K

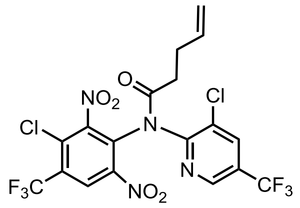

31

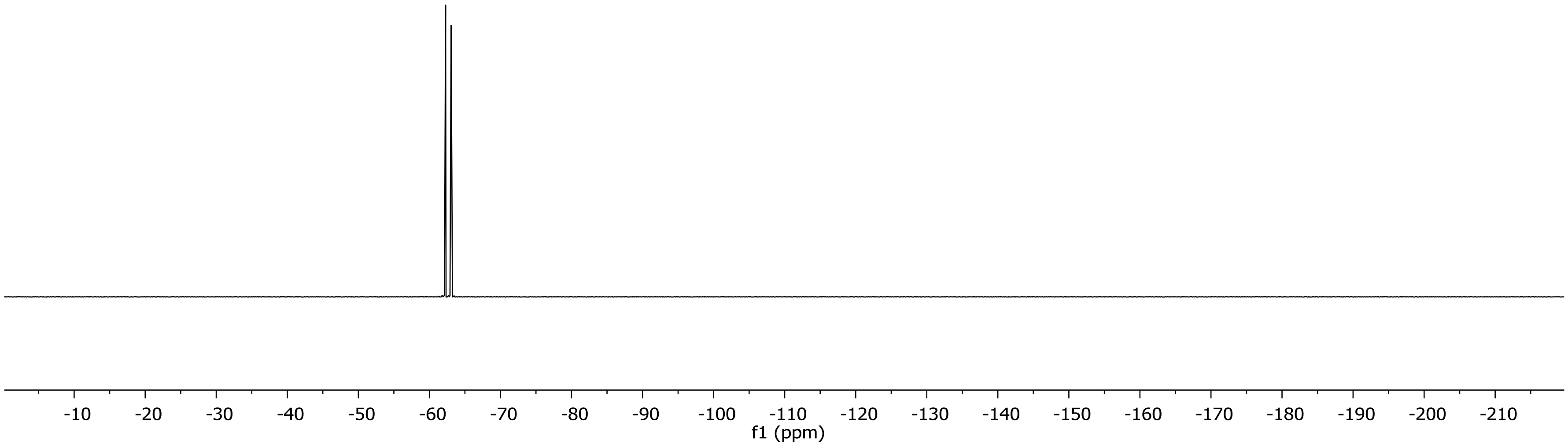

Probenecid-derived alkene 32

^{1}H NMR of Probenecid-derived alkene 32

$CDCl_3$, 298 K

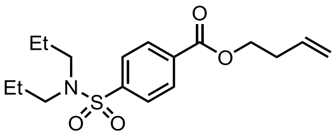

32

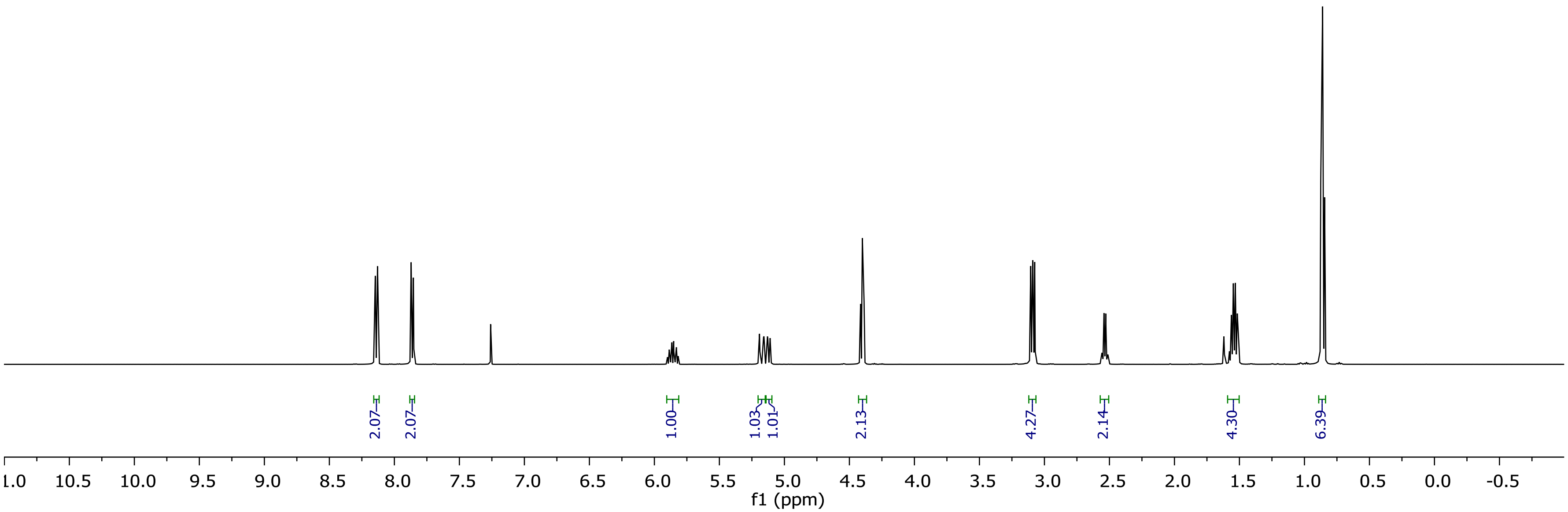

^{13}C NMR of Probenecid-derived alkene 32

$CDCl_3$, 298 K

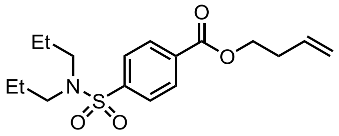

32

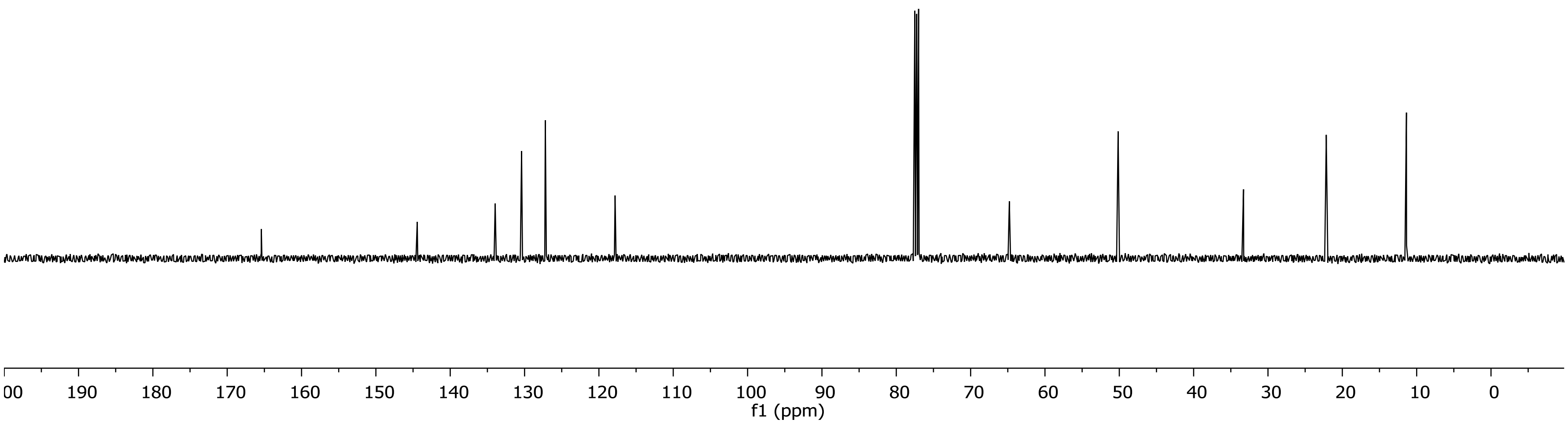

Bicalutamide-derived alkene 33

^{1}H NMR of Bicalutamide-derived alkene 33

$CDCl_3$, 298 K

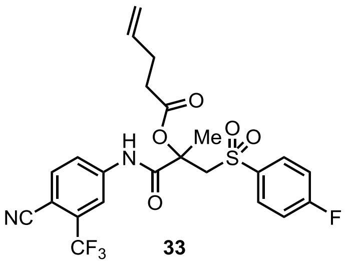

33

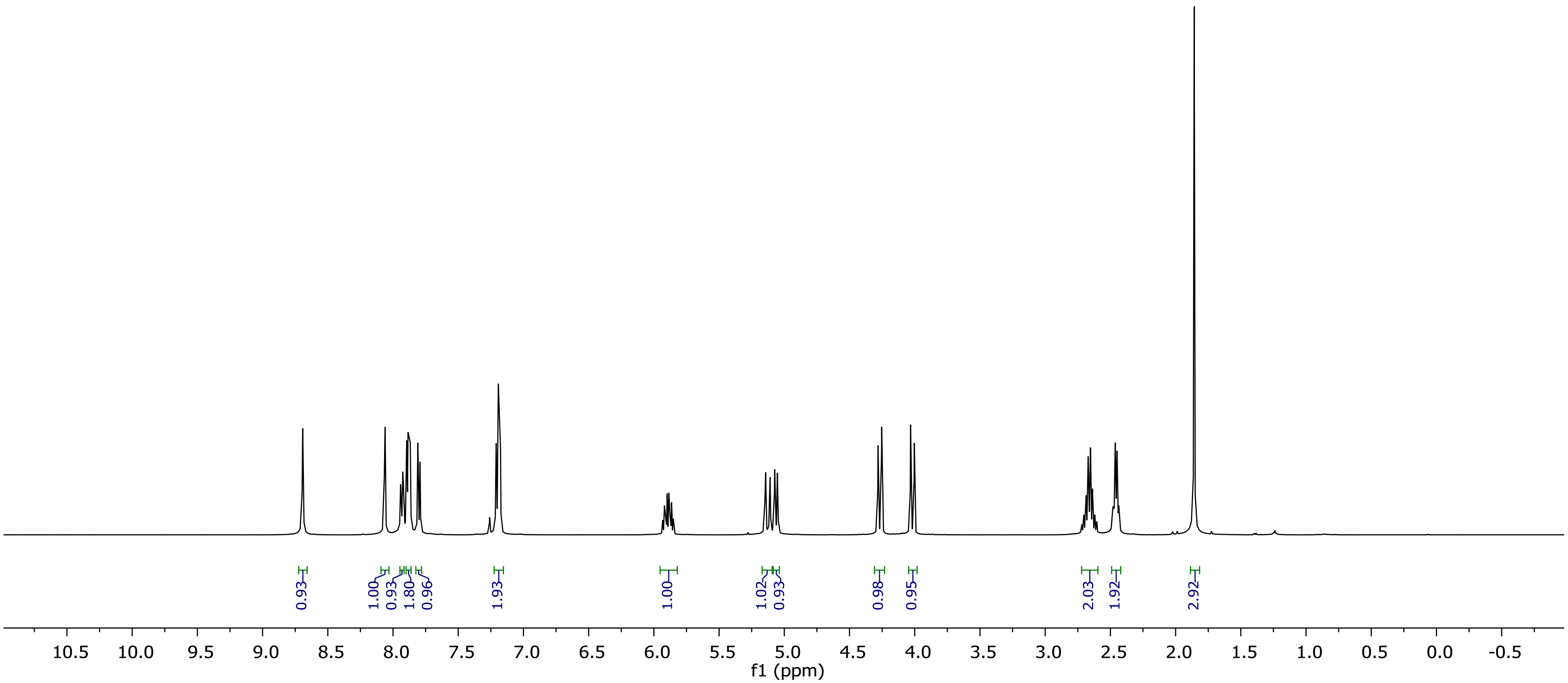

^{13}C NMR of Bicalutamide-derived alkene 33

$CDCl_3$, 298 K

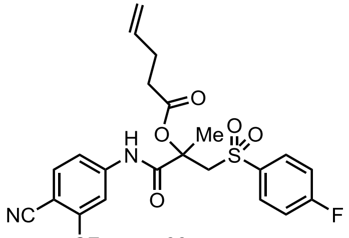

33

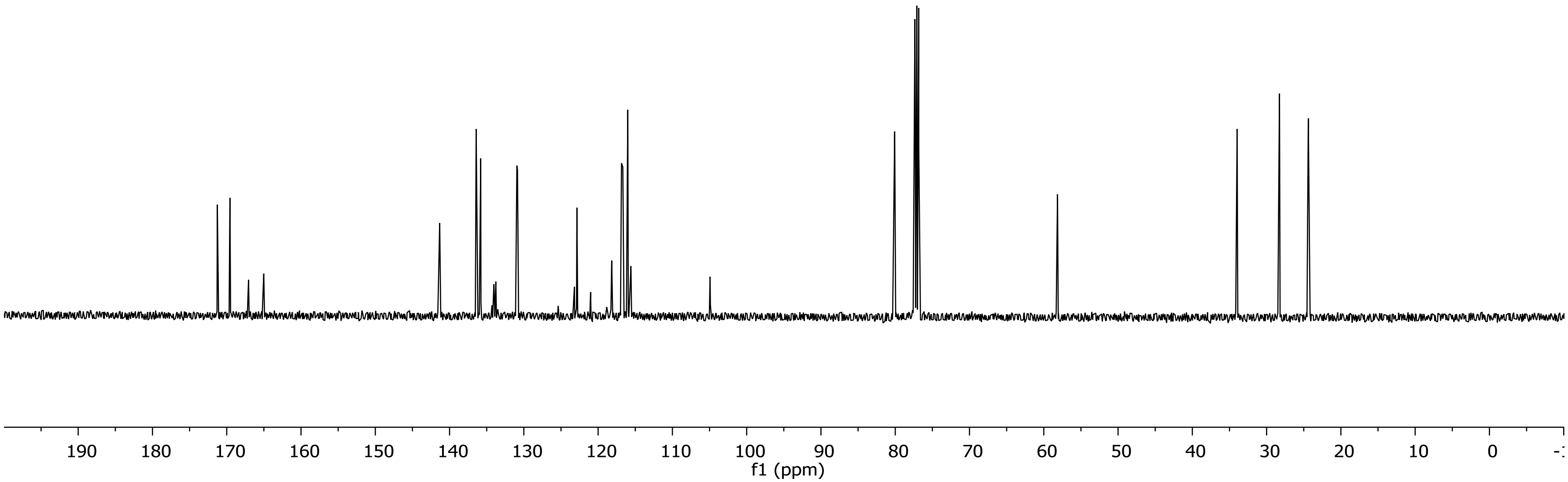

^{19}F NMR of Bicalutamide-derived alkene 33

$CDCl_3$, 298 K

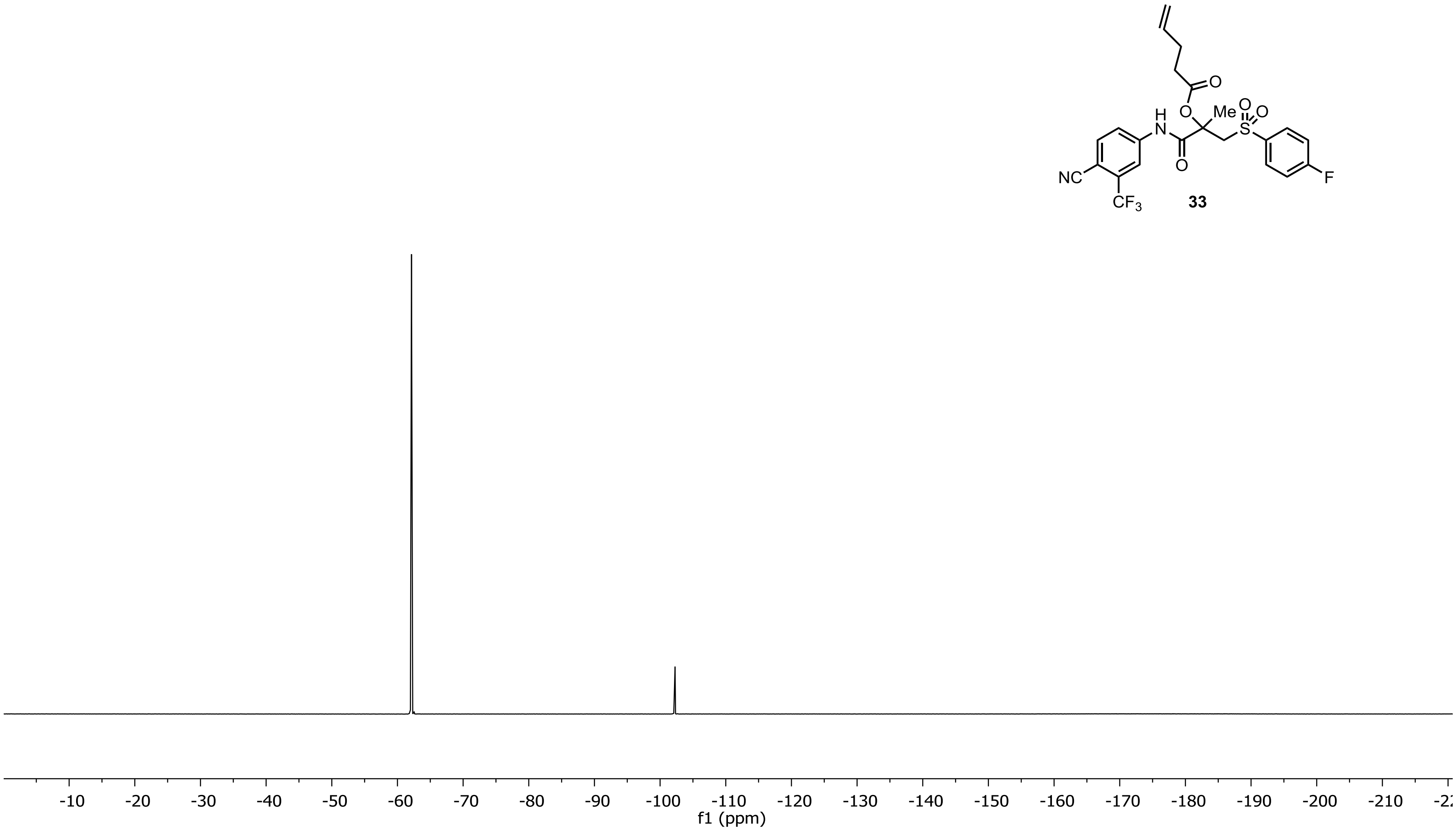

trans-4-Octene-derived thianthrenium dication 2-INT

^{1}H NMR of trans-4-octene-derived thianthrenium dication 2-INT

CD_3CN, 298 K

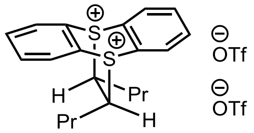

2-INT

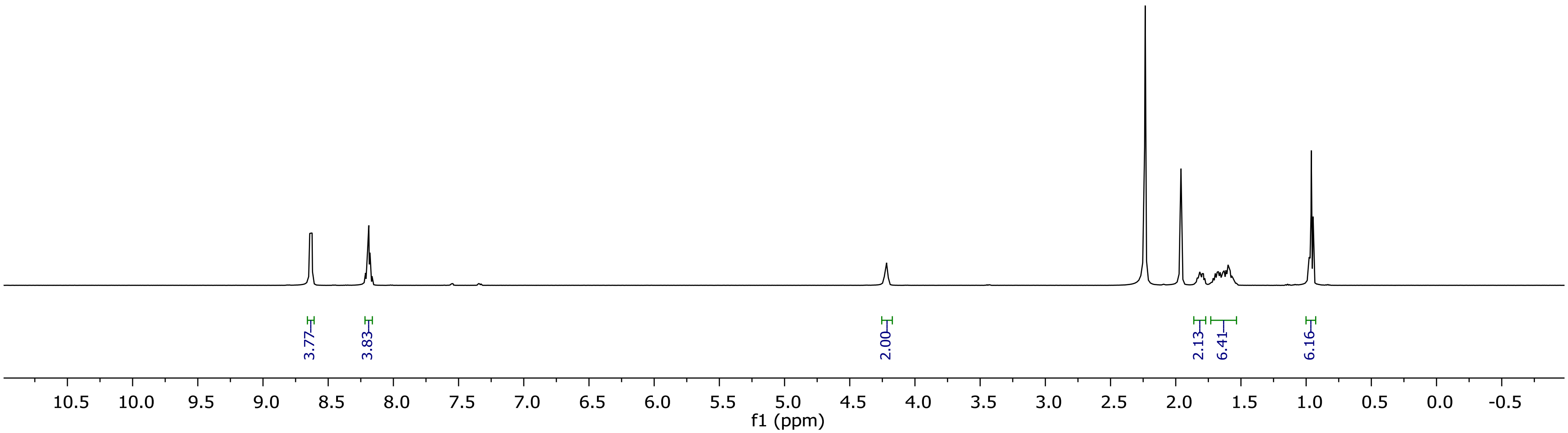

^{13}C NMR of trans-4-octene-derived thianthrenium dication 2-INT

CD_3CN, 298 K

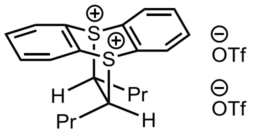

2-INT

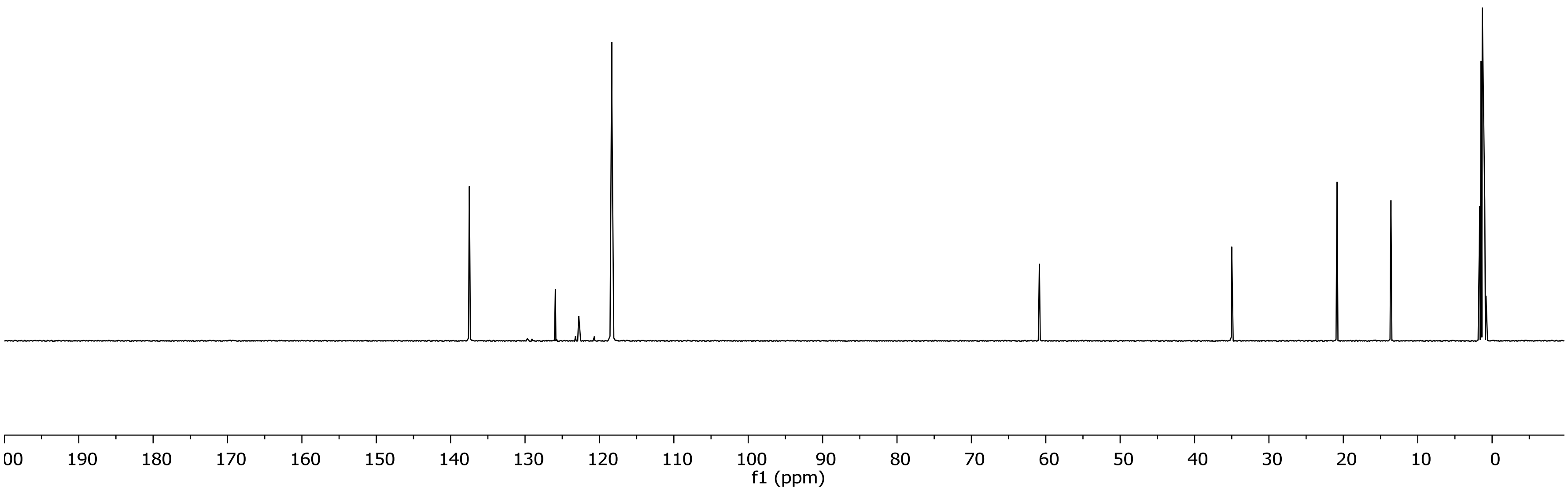

^{19}F NMR of trans-4-octene-derived thianthrenium dication 2-INT

CD_3CN, 298 K

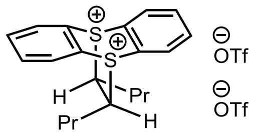

2-INT

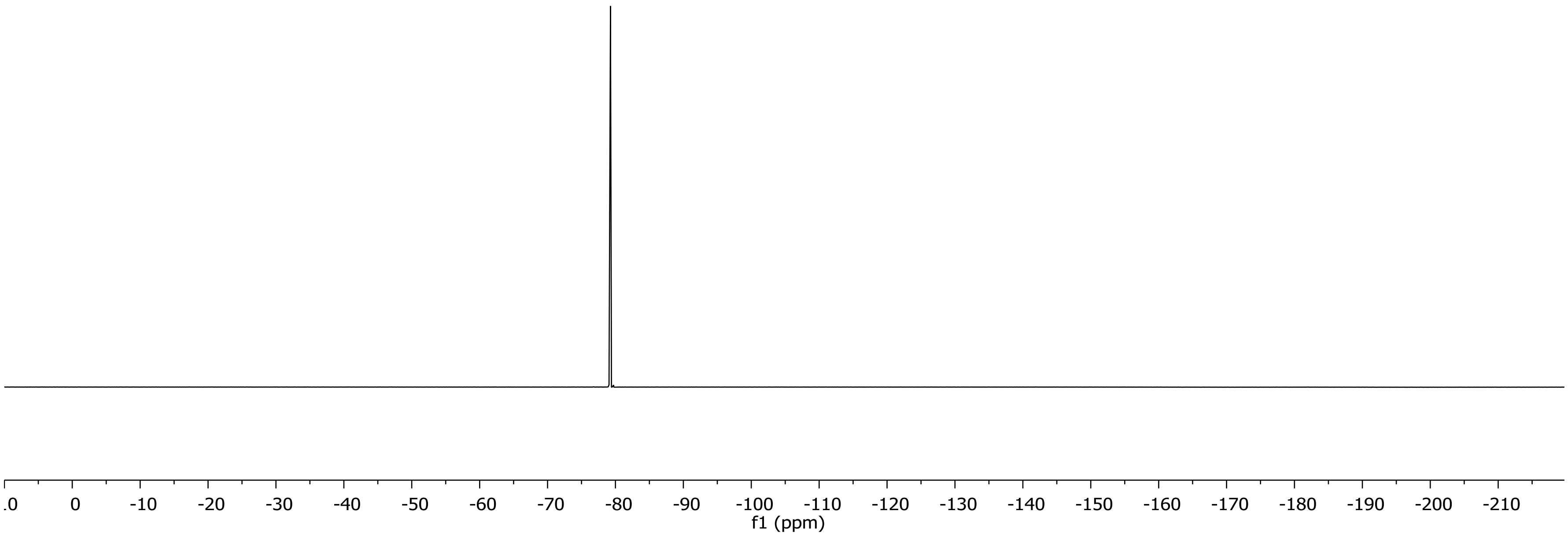

cis-4-Octene-derived thianthrenium dication 3-INT

^{1}H NMR of cis-4-octene-derived thianthrenium dication 3-INT

CD_3CN, 298 K

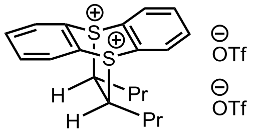

3-INT

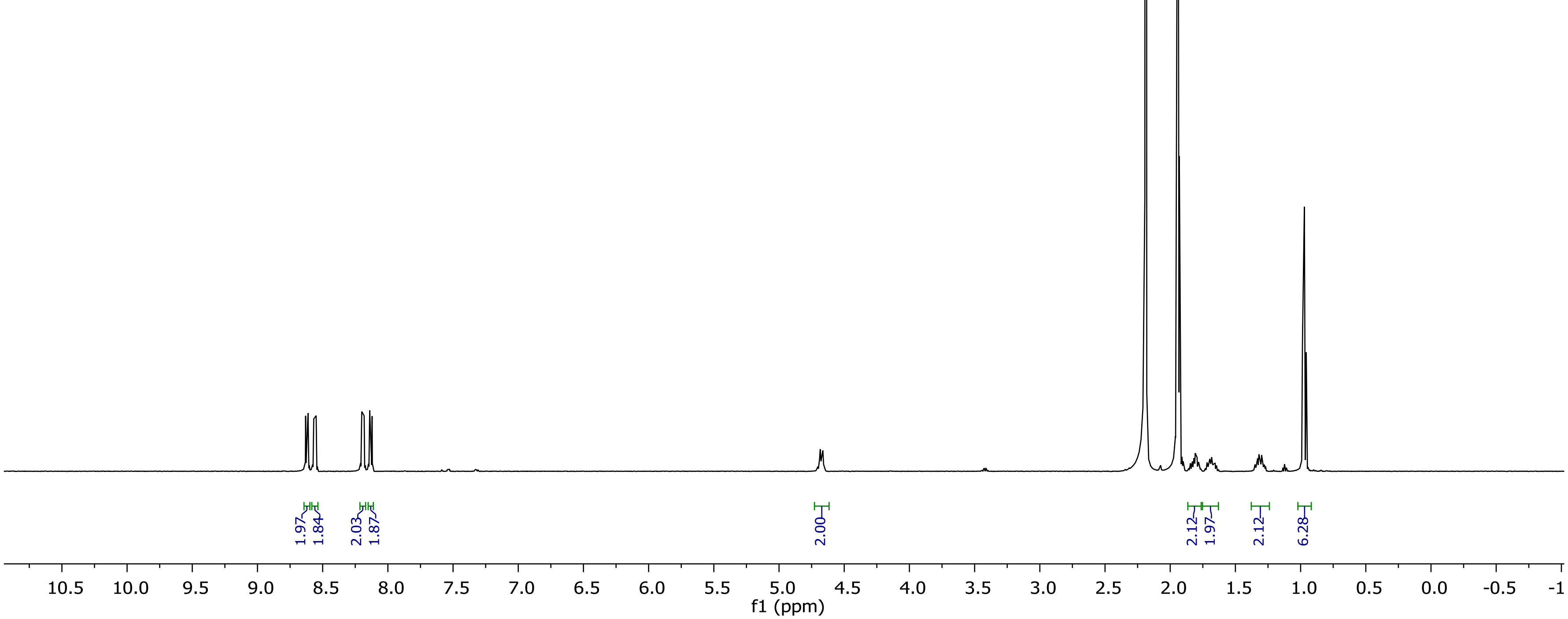

^{13}C NMR of cis-4-octene-derived thianthrenium dication 3-INT

CD_3CN, 298 K (compound decomposed during measurement, and free thianthrene was observed)

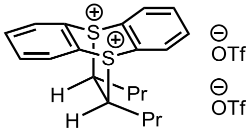

3-INT

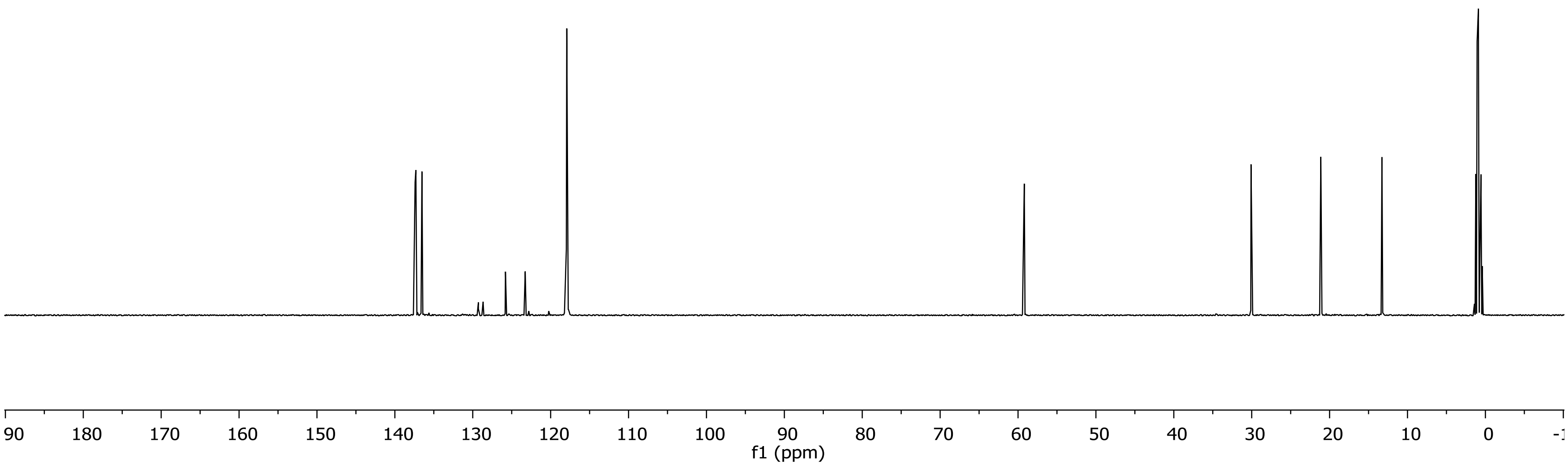

^{19}F NMR of cis-4-octene-derived thianthrenium dication 3-INT

CD_3CN, 298 K

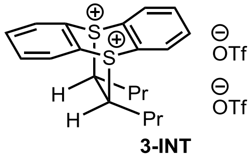

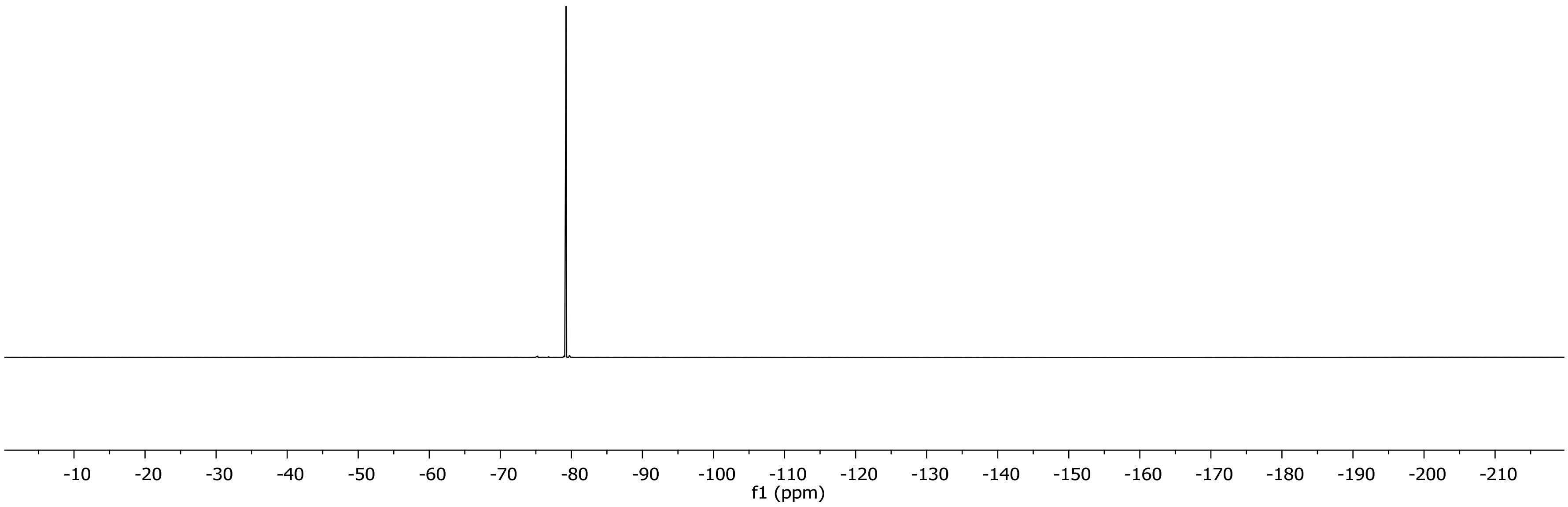

Supplement: Supplementary file 1 — Supplementary [file ANIE-59-5616-s001.pdf]
